# 117th Annual Meeting Medical Library Association, Inc. Seattle, WA May 26–31, 2017

**DOI:** 10.5195/jmla.2018.421

**Published:** 2018-01-02

**Authors:** Nicole Mitchell

## INTRODUCTION

The Medical Library Association (MLA) held its 117th annual meeting in Seattle, Washington, May 26–31, 2017, at the Washington State Convention Center. The meeting theme was “Dream, Dare, Do.” Total attendance for the meeting was 1,584, with 320 participating in continuing education courses. Additional meeting content—including the meeting program and various electronic presentations from the business meetings, plenary sessions, poster sessions, and program sessions can be accessed by all meeting registrants via the MLA ‘17 website.

## WELCOME TO MLA ‘17

### Sunday, May 28, 2017

MLA President Teresa L. Knott, AHIP, welcomed attendees to the 2017 annual meeting, noting that the meeting theme “Dream, Dare, Do” was particularly suited to the meeting location of the Pacific Northwest. She updated the audience on meeting format and “Town Hall” feature of the Business Meeting and encouraged everyone to attend the plenary sessions and visit with vendors throughout the meeting.

President Knott then introduced Laura Zeigen, AHIP, who welcomed attendees on behalf of the Pacific Northwest Chapter. President Knott returned to the podium to introduce the 2017 National Program Committee and the 2017 Local Assistance Committee: Deborah Sibley, cochair, National Program Committee, and director, Libraries, Ische Library, Louisiana State University Health (LSU Health)–New Orleans; Donna R. Berryman, AHIP, cochair, 2017 National Program Committee, and senior associate director, Medical Center Libraries and Technology, Edward G. Miner Library, University of Rochester Medical Center, Rochester, NY; Tania Bardyn, AHIP, cochair, 2017 Local Assistance Committee, and associate dean and director, Library, University of Washington–Seattle; and Catherine M. Burroughs, cochair, 2017 Local Assistance Committee, and associate director, National Network of Libraries of Medicine, Pacific Northwest Region, University of Washington–Seattle. President Knott thanked everyone for their work on planning the meeting.

Deborah Sibley and Donna Berryman, AHIP: On behalf of the 2017 National Program Committee (NPC), welcome to Seattle where we will dream, dare, and do. Just in case you hadn’t figured that out yet. The committee has worked with MLA staff for three years to plan this year’s meeting, and we hope you will enjoy Seattle and your time here.

We are happy to be here in Seattle over Memorial Day weekend and during the Northwest Folklife Festival. We hope you’ll spend some time enjoying Seattle and the festival but also enjoying the things that MLA has to offer. The NPC group, MLA staff, and many volunteers have worked really hard to bring you speakers and programming that will challenge you to think about your local libraries and the changing environment in which we work.

We’d like to recognize the members of the NPC and the Local Assistance Committee. We’ve enjoyed working with everyone in the group. Some of you, we’ve worked with as colleagues in libraries. With others, we’ve had a really great chance to get to know you and to work with you. All of you have made being part of the NPC enjoyable, thought-provoking, and fun. But I have to say, I won’t miss those monthly phone calls.

Again, welcome to Seattle.

Tania Bardyn, AHIP, and Catherine M. Burroughs: Good morning. As cochairs of the Local Assistance Committee, we’re delighted to welcome you to Seattle. At the peak of Copper River salmon season, no less.

The Local Assistance Committee has been busily preparing for your arrival, and both of us agree that we could not have had a more creative and focused team. We ask the local subcommittee chairs and committee members to please stand as your names are called and remain standing. For those in the audience, we ask that you please hold your applause until the end. Lisa LaPointe, *Restaurant Guide* chair; Emily J. Glenn, AHIP, publicity and promotion chair; Molly Montgomery, Local Assistance Committee blog chair; Frances Chu and Andrea Ball, Hospitality Center cochairs; Terry Ann Jankowski, AHIP, FMLA, and Emily Patridge, AHIP, Continuing Education Committee cochairs; Gail Kouame and Carolyn Martin, AHIP, volunteer assistance cochairs, and all of our fantastic volunteers who stuffed bags, led dine-arounds, and will be staffing the Member Resource Room or the Hospitality Center; Patricia Devine, service project chair; and Tania for organizing the cruise to Alaska. So please join us in a round of applause for all these talented and dedicated MLA members. And again, welcome to “Dream, Dare, Do.”

Deborah Sibley then talked about this year’s community service project, Mary’s Place, a shelter for women and families. She encouraged attendees to make a donation online or to stop by the Hospitality Center to learn more about the organization.

President Knott returned to the podium to recognize and thank meeting planners and all the vendors who generously contributed to the meeting’s success.

Deborah Sibley then introduced President Knott who gave her Presidential Address.

## MLA PRESIDENTIAL ADDRESS: TERESA L. KNOTT, AHIP (PLENARY SESSION 1)

Teresa L. Knott, AHIP: Good morning again. So last year, I talked to you about building value and how we go about doing that as an association. This year, I’m going to talk to you about building value in the framework of dreaming, daring, and doing. I’m sorry—by the end of the meeting you’re probably going to be tired of that. But along with that, I’m going to talk about the adventures of being president. From my perspective and the purposes of this speech, goals are articulated achievable dreams. Reaching outside your comfort zone is daring. And achieving goals through action is doing.

I think many of you have heard over time that the Executive Committee and the MLA Board have changed how we do planning, and we adopt strategic goals that roll through time that may last two to five years and then they roll off. Last year, I talked about Dream 1 or Dare 1 or Goal 1, however you want to look at them, about what MLA does.

Some of the things that are already done: We’ve examined our audiences and what we want to do for different audiences. We’ve worked on our annual meeting activities by combining the business meeting and various other tweaks. We actually have ninety more minutes of programming than we previously had, which gives our members more opportunities to share their knowledge and practice with each other. In terms of publications, this year we moved the *MLA News* and *Journal of the Medical Library Association (JMLA)* into electronic platforms, which make them more manageable, hopefully more accessible, and, from a budget standpoint for MLA, about $90,000 less expensive per year. So we really appreciate that time and money saving. In addition this year, we have appointed the editor-in-chief of the *JMLA,* who I will introduce at the Business Meeting, Katherine Goold Akers. I hope you’ll reach out and get to know her. I think she’s a tremendous addition to the *JMLA.*

On the side of doing to done, so in process and almost done, we’ve done some work at headquarters and with governance. At headquarters, I think Kevin Baliozian has tried really hard to identify efficiencies. We’ve looked at space at MLA headquarters in our lease. We can’t get out of our lease, but when we do, Kevin will find us a more reasonably priced place to live as an association. We have instituted new systems at MLA and made things cleaner, more effective. I’ll talk a little more about that under the technology goal. From an organizational standpoint, the most important things we have done as an organization—and I include the entire organization, the people who are sitting here, the people who are unable to attend—is pass new bylaws. That has made a tremendous difference to the organization to be able to move forward more rapidly and in a flexible way. The board approved two new membership categories. One of them is for international members who can join at a lower rate if they’re from a HINARI-eligible country. The other thing we instituted was a new institutional membership pilot. At this point, we have 34 institutions that have taken advantage of the new institutional membership, where, based on the level they join, they can give discount coupons to their staff. At this point, there are 245 certificates out there for discounts to members at eligible institutions. At this point, only 131 of them have been submitted, so if you work for an institution that has an institutional membership, go talk to your director and ask them where your certificate is.

Also, we continue to work on sections and special interest groups (SIGs). We’ve done some housekeeping things like make reporting easier and less tied to MLA’s presidential priorities, where sections and SIGs had to tie what they were doing to those goals, artificially perhaps. And they don’t have to do that anymore. By a show of hands—this is the audience participation part—how many of you read the annual reports for all the committees and sections and chairs? Yay! For those of you who haven’t done that, I would actually encourage you. One of the new features of the annual report is that each section, committee, chapter chair submits an executive summary of the work of their part of the organization. So it’s actually pretty easy to read the *Cliff’s Notes* version, and you might find that people are doing things that you don’t know and that you might like to join them in doing.

Another decision the board made this year was to change our election process. We had a happy accident where we didn’t have enough candidates identified for the Board of Directors, and so we ran two separate elections. And then we said, “Why are we doing something that takes sixteen months to do?” And we said, “Well, we’re doing that because we used to have to mail things out, wait for things to come back, we had to wait for people to mail in profiles or answer questions.” But we live in an electronic age. We don’t have to do that anymore. So for this year only, the schedule on the right is the new schedule. You can see it’s a little compressed. But for this year, Michelle Kraft, AHIP, and I will cochair the Nominating Committee as past presidents, and we think this will be more effective. And it will also deal with the people who are elected in December, then they don’t take their office until May and they’re like, “What do I do, what do I do, what do I do?” Which is great and wonderful, but it’s like, “Wait, you don’t take your office until May, so we need you to chill.” So we think that will resolve that issue, and we will work more efficiently. With all of these actions, the board has decided that we have completed Goal 1, or Dare 1, and the board voted to close this goal.

Last year, I spent a lot of time talking about education because Goal, or Dare, 3 is education. We’re going to talk a lot about that. There are many things that happened in the last year, moving this goal forward. The task force who worked on professional competencies finished their professional competencies. I’m going to spend a fair amount of time talking about that. I particularly want to thank this committee for all of their hard work. What they’ve accomplished is tremendous and allows us to go forward. We had not updated our previous competencies in over ten years. And so this gives us a basis to start and has established a really strong process for us going forward to look at these competencies and come back to them from time to time. So I would especially like to thank Paula Raimondo, AHIP, FMLA, who started chairing the committee, then retired, and Gail G. Hannigan, AHIP, who very graciously and kindly stepped up. And Barry Grant, the director of education at headquarters, did a really remarkable job of steering this committee and these accomplishments.

I’m going to run through the guiding principles that are driving our education work. We launched our new learning management system, MEDLIB-ED. You may have heard of it by now. I hope you have. We are working on a stronger plan to develop content for our education system, and when many of these things come closer to being completed, we’ll have a comprehensive marketing plan to go forward.

Here are the education guiding principles that you saw last year. I’m not going to go through all of them, but essentially we wanted to make sure that whatever we’re doing in education is intentional; is learner-centric, so based on what the learner needs, not what we think they need to learn; member-focused as well, there’s alignment there; as well as striving for excellence in this area. Lastly, we wanted to make sure that it’s resource savvy. We know that our members have full-time jobs and that what we do for MLA is professional development, volunteerism, and we need to be mindful of how precious that resource is. In addition, we don’t have a huge staff at MLA headquarters, so we need to use those resources judiciously.

So, the competencies: And my surprise today is, on each table is a handout on the competencies. But I am going to walk through them one by one. The first competency is on information services. Each of the competencies has a brief statement and then a deeper explanation of what’s going on. So in the case of information services, “a health information professional locates, evaluates, synthesizes, and delivers authoritative information in response to biomedical and health inquiries.” That seems like a good starting place for medical librarians. Each of the competencies has performance indicators attached to it. So in this case, these are the first three indicators for information services. Indicator 1 is “assess information needs.” That’s a pretty fundamental librarian skill. And the basic version of that would be “uses reference interview skills” and expert would be “uses the language of biomedical science,” so adopts that. These are three of six indicators, and each competency has its own indicators, trying to get at a range of behaviors and knowledge.

Competency 2 is on information management, where “a health information professional curates and makes accessible bioscience, clinical, and health information data, information, and knowledge.” Competency 3 focuses on instruction and instructional design. So “a health information professional educates others in the skills of bioscience, clinical, and health information literacy.” Competency 4 is on the dimensions of leadership and management: “A health information professional manages personnel, time, budget, facilities, and technology and leads others to define and meet institutional goals.” Competency 5 focuses on evidence-based practice and research. And basically, “a health information professional evaluates research studies, uses research to improve practice, conducts research, and communicates research results.” The last competency, number 6, is on health information professionalism: “A health information professional promotes the development of the health information professions and collaborates with other professionals to improve health care and access to health care information.”

So here is a preview of MEDLIB-ED. The top page of MEDLIB-ED, at this point, is all about the opportunity for members to go through the competencies and self-assess. You’ll find it on the home page, and I’ve added a shortened uniform resource locator (URL), if you want to use that. We’re very excited that this went out in MLA-FOCUS last week, and at this point, over 400 MLA members have started the process of self-assessing. So thank you. I hope everybody else will do this. I think that it’s also worth thinking about in terms of your work life. It may be something you want to include on your CV. It might be something, as a manager, you want to begin including in performance appraisals, particularly because of the dimensions in performance indicators and the range of behaviors from beginner to expert. Before I move on, one of the things I’d encourage you to do is stop by the MLA booth at the bottom of the escalator and tell your own story. We are recording elevator speeches, and we would love to have people come by and talk about a particular competency you have in this area or other things that you would like to share.

MEDLIB-ED launched last month. It’s the accumulation of a lot of work by a lot of people. It offers personalized learning spaces. It has a catalog of courses that are available that have MLA continuing education (CE) credit and is also home for courses from the National Network of Libraries of Medicine (NNLM). It’s conceivable that we could host content for chapters or sections. It can be linked to a third party. I hope you’ll go explore it. It’s also a place where you can track your CE, you can keep your certificates there in one easy place, where you can just go look it up and say, “Ya know, I took that class on information management last year.” It won’t go back previously, but if you have a collection of CE certificates you can take that and scan that and add that to your MEDLIB-ED portfolio. It’s a nice way to be able to track that.

Another big change that came to education this year is that we have restructured the education enterprise so to speak. We created an Education Steering Committee. I think that over the years, we’ve come to realize that the Continuing Education Committee has done an extraordinary job of creating content throughout the year for courses for the MLA annual meeting, they’re really good at picking up and identifying trends where you might want to have a course on data management or precision medicine. Part of their charge was also to do strategic planning and education. And what we realized is within the parameters of their work scope that was just really difficult to do. So the board voted to change the education structure. There is an Education Steering Committee that is tasked with doing high-level strategic planning for our education enterprise. And, what will happen is, under the Education Steering Committee, you will have curriculum committees. And I’ll show you a diagram on the next slide. The Education Annual Programming Committee will actually be one of the curriculum committees, but they will continue to be tasked with creating CE throughout the year and for the annual meeting and doing webinars. They are really the annual programming committee for education. To begin to address some of the strategic needs, we will develop different curriculum committees. At this point, the board has approved two: one of them is on leadership and management and the other is on research. As we go forward, what will happen is the Education Steering Committee will identify possible areas where we should really focus on growing content for continuing education and make recommendations to the board to form additional curriculum committees. It’s been suggested, but not decided, that, in fact, the competencies are a really good starting place. So there may end up being a curriculum committee for each of the content areas in the competencies. That may or may not happen. That may be a better theory than a reality.

So this is how the organization chart will look for education. On the left, essentially you have the member-driven portion of the curriculum with the support of the staff side. So the director of education is a liaison, an ex officio member, of the Education Steering Committee. And the education director, Barry Grant, is able to assess the kind of resources that need to be invested from the MLA side. So you’ll see that there is the Education Annual Programming Committee, the Leadership Curriculum Committee is there, as well as the Research and Evidence-Based Practice Curriculum Committee.

Goal 4, or Dare 4 if you prefer, is focused on technology. The board had a very lively conversation, and we decided we are going to quit naming goals with numbers because we could be at goal number 23, and somebody could say, “What happened to goal number 20?” and “What happened to goal number 1?” We are going to give them real names in the future. So in this area on the doing-to-done side: Kevin has done remarkable work updating the information systems that headquarters uses. And with the adoption of MLANET using Socious as a platform, we were able to create, we hope, a better user experience, to launch communities where similar groups, whether that’s a committee or a section or a SIG, can gather together and share documents and information within a community. So that’s happened in the last several years. We’ve also worked to improve the integration of content into the new MLANET from the annual meeting. And much to Barry’s delight and to Debra Cavanaugh’s, we launched that new learning management system. So, hooray!

The next goal or dare is in the area of research. We have really exciting news. I’m going to lead with the big news. The Research Imperative Task Force, working with headquarters staff, successfully competed for an Institute of Museum and Library Services (IMLS) grant. It’s a three-year grant to start up an MLA research institute that is focused on building research skills and knowledge for medical librarians. And that’s just a tremendous accomplishment.

Looking at what’s gone from doing to done, the Research Imperative Task Force has been really hard at work, and I really want to call out their work between the IMLS grant and much of what’s accomplished on this slide. Susan Lessick, AHIP, FMLA, is the chair of that committee, and we very much appreciate their hard work. Under the guise of professional development, they worked with the Task Force to Review MLA’s Competencies for Lifelong Learning and Professional Success to make sure that research is represented in the competencies and what we as librarians need to know.

They have created a curriculum, and because of the grant we are going to be able to more rapidly move from a curriculum to a research institute, although the research institute will be part of the curriculum. All that goes together. And they worked to strengthen the research mentorship programs for MLA, trying to really get members to sign up to be mentors, especially when they have research expertise. And I will say as sort of a preview, I think we’re evaluating our mentorship database at this time and we may try to flip it around. Right now it’s very mentor-centric, what does the mentor do or what expertise does the mentor have, and I think we’d like to flip it to “What do I as the member need, where do I need help and how do I go about finding someone to help me in that area?” Barbara A. Epstein, AHIP, FMLA, will talk more about this tomorrow because a lot of this stands up, but I do need to go through more.

The Research Imperative Task Force has worked on consolidating research tools and aids for librarians to use on MLANET. They’ve also worked on gathering information on how we can prove our value and what health sciences librarians bring to the table at our organizations. They have instituted a program, or an award, where we will be able to honor a library that fosters librarian-led research. So for those of you who are at libraries that do a lot of research among the rank and file, this is something to start thinking about and working with your leadership to submit an award application. There’s a lot of exciting things going on in this arena, and that will continue.

Our last goal that I talked about last year was communities. I’m going to defer this to Barbara Epstein in her inaugural address. She has been the board liaison to the communities task force, and she has a lot more in-depth picture of what’s going on in this, so I’m going to punt and let her have that.

And in the last bit of goal-related news, on Friday, the Board of Directors voted to launch a new goal focused on diversity and inclusion within the Medical Library Association. I’m very excited about that. Barbara will talk more about that as well. It’s nice to be able to delegate, she’s my understudy.

Well, that wraps up the sort of stroll through the goals. I really want to talk to you about advocacy. I think, in the last year, we’ve learned that a lot of times members don’t know what the association is doing in advocacy. MLA has a really strong, dynamic history of engaging in advocacy efforts. This year, we’ve responded to the request for information (RFI) from the National Library of Medicine (NLM) for their strategic planning process. I especially thank the people who either willingly volunteered or were willing to say yes to me when I called and said, “Please, can you help us?”, as we submitted a cohesive response from the Medical Library Association. We also responded as an organization to the National Institutes of Health (NIH) request for information on their data management and sharing plans. We supported testimony for NLM’s 2018 funding, and we’ve signed on several public policy statements in that regard.

Earlier, I mentioned that the Research Imperative Task Force has been gathering information. I would encourage you to navigate on over to the Advocacy tab on MLANET and look at the advocacy page, where there is the page on “Aim for Excellence.” This is a great resource for hard data about what librarians contribute and to help you advocate for yourself in your own organization. I think they’re really great numbers, and I hope you’ll take a look. And again, we have the Research Imperative Task Force to thank for that.

Another thing that we did this year, in February, the MLA Board adopted the statement that you see before you, on our core values. We decided that as we were asked to respond to different things going on in the environment, it was really important for the board to be able to look at what our values are as an organization. So that’s page one. That’s page two. And I hope that this will resonate with you. It’s based on going through our vision, our mission, our values, other public statements that we have made. In addition, in March, we signed on to a joint statement with our Canadian colleagues, the Canadian Health Libraries Association/Association des bibliothèques de la santé du Canada, where we affirmed our cooperation and our interest in being able to travel across borders to work together and to share our knowledge and research with each other.

This is this year’s Joint MLA/Association of Academic Health Sciences Libraries (AAHSL) Legislative Task Force. This year, we made two trips to Washington, DC. The first trip is the picture of Michelle, Barbara, and myself in front of the South Carolina flag. We visited Capitol Hill last June, and we decided that June is a really rotten time to traipse around Washington, DC. We decided that spring would be a much lovelier time, so this year we went in April, and the picture on the left is of this year’s joint legislative task force before we headed off to the Hill. It’s a tremendous opportunity, where we sit down to prepare by talking to the governmental relations person from NLM and the governmental relations person from the Association of American Medical Colleges. In this particular case, we were advocating this spring that Congress pass fiscal year (FY) 2017 and FY2018 budgets. At that point, we were two weeks away from a continuing resolution that kept the government from running. And we were talking about how it’s difficult for federal agencies to do their work if all they know is they have funding to the end of April or to the end of May, rather than an entire year.

We, of course, advocated for an increased budget for NIH. We also always talked to the legislative aides about the products of NLM and how the databases and many other things that NLM does touch so many people. We talked about how the small grants from the National Network of Libraries of Medicine can make a tremendous difference in a small community and really advance people’s access to health information. In addition this year, we very strongly encouraged them to preserve funding for IMLS. And it’s always educational for us as well, seeing the different perspectives from different legislative aides.

This afternoon, I hope that you will join us, as I mentioned earlier, for the “Activism in a Time of Turbulence” open forum. I think it’s a great opportunity to learn about how you can work to affect policy locally and nationally. One of my takeaways from the joint legislative task force is what we say as a constituent has a lot more impact than what MLA says as an organization. And so I think it’s something that we all need to consider.

Being president is a grand adventure. We are part of a very robust community. It’s been a privilege to learn and grow with you. I thought before I move on to the thank you part that I want to share some of the adventures it was my privilege to do this year as president. One of the most exciting things was to be able to attend the installation of Patricia Flatley Brennan as the director of NLM. That was awesome. Dr. Brennan will be here at this meeting. I hope you’ll go talk to her. Don’t call her Dr. Brennan. The first thing she said when I called her Dr. Brennan, she’s like “No, it’s Patty.” So I hope that you will join us Wednesday morning, when she will deliver the Joseph Leiter NLM/MLA Lecture. I think it will be a great opportunity to look at what NLM is considering in the future. So I hope that you will be there for that.

I got to attend the gala for the Friends of the National Library of Medicine, where I got to see Shari Clifton, AHIP, awarded the Michael DeBakey Outreach Award from the Friends of the National Library of Medicine.

I got to go to four chapter meetings. Michelle Kraft and I have talked about this, and I think we have similar goals of attending every single chapter meeting because each chapter is unique and special. And it’s a great place to meet people, learn about different things, see what’s going on in the chapters, see what’s going on in different regions. I actually would encourage you if you have the opportunity to go to a different chapter meeting, you should think about doing it.

In Philadelphia, we took a field trip from the [Librarians, Leadership and Learning] 3L chapter meeting to the national historic marker, or the Pennsylvania marker, that marks the spot of the first Medical Library Association meeting. You may notice that Squatch went, too. Squatch went a lot of places. Squatch made some friends. Squatch also went to the South Central Chapter, where Squatch met Scammy, the South Central Chapter mascot. And back to the joint legislative task force. On our trip in April, Michelle and I found out that the senator from Ohio, Sherrod Brown, is actually in Barrack Obama’s former office. We found the Senate Library. Who knew there was a Senate Library, and a House Library, as well as the Library of Congress? And we found this awesome poster in the dining hall encouraging staffers to seek out help from the Senate Library. We like those promotions. I learned that if you go into any Ohio senator or representative’s office, you will find Dum-Dum suckers because Dum-Dum suckers are made in Ohio. You learn something new all the time.

So now we come to the thank you portion, or what I like to say is the yearbook portion of the speech. First, I would like to thank the members for this privilege. And I realize that there are no pictures of, like, audiences, so this was at the exhibit opening last year. It has really been amazing, and I appreciate it. I also want to thank the MLA headquarters staff. They are extraordinary. They provide us continuity. They keep us in line. Sometimes we nudge them; sometimes they nudge us. It all works. And I think what I especially appreciate is their deep commitment to advancing our profession. Serving with this Board of Directors has been amazing. It’s a group of very talented, committed, amazing people. I count them as friends. Thank you.

So here’s where it becomes the yearbook. I think as we walk through life, there are many people who touch us. And these are a few, and I know I’ve left people out despite the crowdedness of it, but thank you. I am fortunate to work with a very strong administrative team that’s given me the freedom and the time to commit to MLA, and I appreciate them. And I work with an amazing group of librarians who picked up the slack, who tolerated their director being gone, ignoring their emails. It’s true. It’s like, “I’ll get to that later. Please remind me five times, and then I’ll do that for you.” And I really appreciate them. These are the librarians of the Tompkins McCaw Library, and there are many staff that keep us going, too. This is a circle of friends who are like family, who have been on this journey with me. And last, but not least, is my family who keeps me grounded and has helped me get here. And with that, thank you.

So I hope you’ll go forth and dream, dare, and do. I really appreciate your time and your attention.

During the past year, we were saddened to learn that several valued members of our association have died. Their counsel and friendship will be deeply missed. We have produced a short video to honor these MLA members: June Bandemer, AHIP, October 24, 2016; Susan J. Barnes, January 3, 2017; Francis (Fran) Brahmi, AHIP, December 25, 2016; William S. Budington, AHIP, FMLA, April 19, 2016; Joanne Callard, January 21, 2017; Julie Lenore Dobbins, June 24, 2016; Katherine Agnes East, March 24, 2016; Eugene Garfield, AHIP, FMLA, February 26, 2017; Velora Avis Jernigan-Pedrick, November 8, 2016; Wendy Lehar, December 26, 2016; Judith Robinson Mercer, July 18, 2016; Ruth C. Morris, November 28, 2016; Gerald (Gerry) J. Oppenheimer, AHIP, FMLA, August 23, 2016; Billy Triplett, November 1, 2016; and Faith A. VanToll Ross, February 12, 2016.

This almost concludes the opening session. At 10:30 a.m., we will gather in this room for the eagerly anticipated John P. McGovern Award Lecture that will be delivered by Julie Angus, an accomplished adventurer, best-selling author, and scientist. She is the first woman to row across the Atlantic Ocean from mainland to mainland and a recipient of the *National Geographic* Adventurer of the Year Award. After her presentation, from noon to 1:00 p.m., if you have her book, she will be available at the EBSCO Health booth, number 301, in the Hall of Exhibits for questions and photos. In the meantime, we have a drawing, and I’m going to make Ray Naegele, MLA’s business manager, come up on stage with me. Julie has graciously donated three of her books for a raffle. Business cards were collected at the Hospitality Center, and I’m going to pull a name from the bowl to see who wins the books. Winners should go to the Hospitality Center to pick up their books. The first book goes to Rebecca Carlson McCall, AHIP, from the University of North Carolina–Chapel Hill. Book two goes to Lisa Blackwell from Chamberlain College of Nursing. Book three goes to Valeria Long from Grand Valley State University. I hope you have a great meeting. Thanks, everybody.

## OTHER PLENARY SESSIONS

All plenary session videos and slides are available online to MLA ‘18 registrants from the MLA ‘18 website. See www.eventscribe.com/2017/MLA for more information about all speakers and sessions.

### Plenary Session 2. Sunday, May 28: The John P. McGovern Award Lecture

Introduction: Kelly Thormodson, member, 2017 National Program Committee, and assistant director, Harley E. French Library of the Health Sciences, University of North Dakota–Grand Forks

*Rowing Across the Atlantic: Strategies to Reach Your Goals*: **Julie Angus,** adventurer, author, and scientist

### Plenary Session 3. Monday, May 29: The Janet Doe Lecture

Introduction: M.J. Tooey, AHIP, FMLA, associate vice president, Academic Affairs; executive director, Health Sciences and Human Services Library; and director, National Network of Libraries of Medicine, Southeastern/Atlantic Region, University of Maryland–Baltimore

*Looking Inside Ourselves: A Culture of Kindness*: **Julia Sollenberger, AHIP, FMLA,** associate vice president and director, Medical Center Libraries and Technologies, University of Rochester Medical Center, Rochester, NY

### Plenary Session 4. Wednesday, May 31: The Joseph Leiter NLM/MLA Lecture

Introduction: Lindsay Blake, AHIP, chair, Joseph Leiter NLM/MLA Lectureship Committee, and clinical services librarian, UAMS Library, University of Arkansas for the Medical Sciences–Little Rock

**Patricia Flatley Brennan,** director, National Library of Medicine, Bethesda, MD

### Plenary Session 5. Wednesday, May 31: Hope Jahren

Introduction: Donna R. Berryman, AHIP, cochair, 2017 National Program Committee, and senior associate director, Medical Center Libraries and Technology, Edward G. Miner Library, University of Rochester Medical Center, Rochester, NY

Moderator: Deborah Sibley, cochair, 2017 National Program Committee, and director, Libraries, Ische Library, Louisiana State University Health (LSU Health)–New Orleans

**Hope Jahren,** professor, University of Hawaii–Manoa, Honolulu, HI

## PRESIDENT’S AWARDS DINNER

The President’s Awards Dinner was held on Tuesday, May 30, 2017, from 6:30 p.m.–10:00 p.m. President Teresa L. Knott, AHIP, welcomed attendees and award recipients.

Teresa L. Knott, AHIP: Good evening! Greetings to you all and welcome to the MLA ‘18 President’s Awards Dinner. Today, we honor our colleagues who have made outstanding contributions to the profession. The names of this year’s recipients are listed in the awards program for your reference. Please note that a few of the recipients are unable to be here this evening.

This was an exceptional year for members of the health information profession. We have members from across the country, and world, who have excelled in every facet of librarianship, and we are pleased to present them and recognize their accomplishments.

Tonight, I would like to thank Shelly Burns, AHIP, chair of the Awards Committee, and Rozalynd McConnaughy, AHIP, chair of the Grants and Scholarships Committee, and all jury members for your time and effort. Shelly, Roz, and members of the juries seated in the audience, would you please stand? Thank you.

I would like to take this opportunity to acknowledge the 2017 MLA section and chapter award winners, honoring their members for outstanding work within their sections or chapters. Would all section and chapter award winners in the audience today please stand to be recognized? Thank you.

The Majors/MLA Chapter Project of the Year Award recognizes excellence, innovation, and contributions to the profession of health sciences librarianship. The Midwest Chapter was selected to receive this award for “A Pilot Mentoring Program for Library Students and New Graduates to Better Prepare for Interviewing for Health Sciences Librarian Professional Positions.”

The MLA Section Project of the Year Award is awarded to an MLA section that demonstrates creativity, ingenuity, cooperation, and leadership within the framework of the mandate of the section. The 2017 winner is the Medical Informatics Section for its project special content session at MLA ‘16, “Data Discovery Bootcamp!”

Sponsored by EBSCO Health, the 4 EBSCO/MLA Annual Meeting Grants provide up to $1,000 each to enable medical librarians new to the profession and working in health sciences libraries to attend MLA annual meetings. This year’s winners are: Toluwase Victor Asubiaro, E. Latunde Odeku Medical Library, University of Western Ontario–London, Canada; Jill Barr-Walker, ZSFG Library, University of California–San Francisco; Annie Cloud Nickum, AHIP, Library Resources, University of North Dakota–Grand Forks; and Jahala Simuel, Louis Stokes Health Sciences Library, Howard University, Washington, DC.

The Ysabel Bertolucci MLA Annual Meeting Grant recognizes a health sciences librarian who is involved in nursing, allied health, consumer health, or international librarianship. We are pleased to present this year’s grant to Robin O’Hanlon, Levy Library, Icahn School of Medicine at Mount Sinai, New York, NY.

The MLA Continuing Education Grant provides monetary awards to MLA members to develop knowledge of the theoretical, administrative, or technical aspects of librarianship. We are pleased to acknowledge Shirley Zhao, Spencer S. Eccles Health Science Library, University of Utah–Salt Lake City. Shirley is unable to be with us tonight.

The Hospital Libraries Section/MLA Professional Development Grant provides librarians working in hospital and similar clinical settings with the support needed for educational or research activities. The 2017 recipient is Sue Espe, AHIP, Merril W. Brown Health Sciences Library, Banner Health-University Medical Center, Phoenix, AZ. Sue is unable to join us.

The Medical Informatics Section awards one individual a grant to support a career development activity that will contribute to the advancement of the field of medical informatics. This year’s recipient is Helene Richards McMurray, Edward G. Miner Library, University of Rochester Medical Center, Rochester, NY.

The MLA Scholarship is granted to a student entering an American Library Association–accredited library school. The 2017 recipient, Christine Remenih, is a student at the University of Alabama–Tuscaloosa.

The 2017 MLA Scholarship for Minority Students recipient is Kelsa Bartley. This scholarship provides funding to a minority student entering an American Library Association–accredited library school. Kelsa is a student at the University of Miami, FL.

The Naomi C. Broering Hispanic Heritage Grant provides a librarian who has an interest in Hispanic/Latino community information services support to pursue a professional activity in the latest medical information services using the latest technical formats. The 2017 recipient is Adela V. Justice, AHIP, Learning Center, University of Texas MD Anderson Cancer Center–Houston.

The MLA Rising Stars program identifies members who will spend a year in a curriculum designed to help emerging association members develop their potential to provide leadership to MLA at a national level. We are pleased to introduce the 2017–2018 Rising Star cohort: Laura Menard, Irwin Library, Butler University, Indianapolis, IN; Hanna Lee Schmillen, Alden Library, Ohio University–Athens; Rachel Keiko Stark, AHIP, University Library, California State University–Sacramento; and Nicole Theis-Mahon, School of Dentistry and Health Sciences Libraries, University of Minnesota–Minneapolis.

The 2016–2017 cohort of MLA Rising Stars spent an active year working on an MLA sections community project, attending learning sessions, and engaging with MLA leaders: Phill Jo, Robert M. Bird Health Sciences Library, University of Oklahoma Health Sciences Center–Oklahoma City; Rachel Charlotte Lerner, Health Sciences Library, Quinnipiac University, Hamden, CT; Tony Nguyen, AHIP, National Network of Libraries of Medicine Southeastern/Atlantic Region, Health Sciences & Human Services Library, University of Maryland–Baltimore; and Gregg A. Stevens, AHIP, Health Sciences Library, Stony Brook University, Yaphank, NY.

The Janet Doe Lectureship is awarded to an active member of the profession for their unique perspectives on the history or philosophy of medical librarianship. This year’s presenter, Julia Sollenberger, AHIP, FMLA, is associate vice president and director, Medical Center Libraries and Technologies, and associate professor, Public Health Sciences, at the University of Rochester Medical Center, Rochester, NY. Sollenberger delivered this year’s lecture, “Looking Inside Ourselves: A Culture of Kindness,” on Monday. As expected, it was indeed a great lecture.

The Joseph Leiter NLM/MLA Lectureship was established in 1983 to stimulate intellectual liaison between MLA and the National Library of Medicine. This year, Patricia Flatley Brennan, director of the National Library of Medicine, will present the Leiter lecture tomorrow at 9:00 a.m. I know MLA members are looking forward to what is sure to be an interesting and engaging lecture.

Adventurer and author Julie Angus delivered the John P. McGovern Award Lecture yesterday. I am sure you will agree that it was a very enlightening lecture. She is unable to attend tonight.

The Erich Meyerhoff Prize was established to recognize and stimulate health sciences librarians’ interest in the history of medicine. The 2017 Meyerhoff prize is awarded to Bart Ragon, Claude Moore Health Science Library, University of Virginia–Charlottesville, for his paper, “The Coming of the Progressive Era to Mr. Jefferson’s Medical School: Violating the Principles upon Which the University Was Founded.”

The Virginia L. and William K. Beatty MLA Volunteer Service Award recognizes a medical librarian who has demonstrated outstanding, sustained service to the Medical Library Association and the health sciences library profession. The 2017 recipient is Marie Tomlinson Ascher, Health Sciences Library, New York Medical College–Valhalla.

The Estelle Brodman Award for the Academic Medical Librarian of the Year is given to a member who has made outstanding contributions to academic medical librarianship. This year’s award is presented to Diana Delgado, AHIP, Samuel J. Wood Library and C.V. Starr Biomedical Center, Weill Cornell Medicine, New York, NY.

The Lois Ann Colaianni Award for Excellence and Achievement in Hospital Librarianship is given to a member who has made significant contributions to the profession through overall distinction in hospital librarianship. This year’s award is bestowed on Marian T. Simonson, AHIP, Floyd D. Loop Alumni Library, Cleveland Clinic, Cleveland, OH.

The Carla J. Funk Governmental Relations Award recognizes a medical librarian who has demonstrated outstanding leadership in the area of governmental relations at the federal, state, or local level and who has furthered the goal of providing quality information for improved health. For over forty-four years, Betsy L. Humphreys, FMLA, National Library of Medicine, Bethesda, MD, has shown extraordinary leadership and service to the profession of health sciences librarianship. She is truly an inspiration to us all. It is my great honor to present her the 2017 Funk award.

Named for one of the profession’s most revered leaders, the Lucretia W. McClure Excellence in Education Award recognizes outstanding practicing librarians or library educators in the field of health sciences librarianship and informatics. Julia Esparza, AHIP, Medical Center Library, Louisiana State University Health–Shreveport, is this year’s recipient.

The Louise Darling Medal for Distinguished Achievement in Collection Development in the Health Sciences recognizes accomplishment in collection development for the health sciences. The 2017 winner is Nancy Lombardo, AHIP, Spencer S. Eccles Health Sciences Library, University of Utah–Salt Lake City.

The Thomson Reuters/Frank Bradway Rogers Information Advancement Award is sponsored by Thomson Reuters and is presented in recognition of outstanding contributions in the use of technology to deliver health sciences information, in the science of information, or in facilitation of the delivery of health sciences information. This year’s recipient is the Health Sciences Library, University of Washington–Seattle.

The MLA Research, Development, and Demonstration Project Grant promotes excellence in health sciences librarianship and information science. This year’s grant is awarded to Amanda Ross-White, AHIP, Bracken Health Sciences Library, Queens University, Kingston, ON, Canada.

The Librarians without Borders® Ursula Poland International Scholarship helps fund an international project by a US or Canadian health sciences librarian. The 2017 recipient is Estelle Hu, AHIP, Library of the Health Sciences, University of Illinois–Chicago. Estelle is unable to attend tonight.

The T. Mark Hodges International Service Award honors an outstanding individual achievement in promoting, enabling, and/or delivering improvements in the quality of health information internationally. The 2017 award is presented to Gurpreet Kaur Rana, Taubman Health Sciences Library, University of Michigan–Ann Arbor.

The MLA HINARI/Elsevier Foundation/Research4Life (R4L) Grants are an expansion of the Elsevier Foundation–funded Librarians without Borders®/E-library Training Initiative that has conducted HINARI/R4L training activities that promote the use of the scientific research resources in emerging or low income countries. We would like to acknowledge 2016 grant recipients tonight as their grants were awarded after MLA ‘16 in Toronto: Megan von Isenburg, AHIP, Duke University Medical Center, NC; Alemayehu Bisrat, Center for eHealth, College of Health Sciences, Tikur Anbessa Specialized Teaching and Referral Hospital, Addis Ababa University, Ethiopia; Martha Cecilia Garcia, National Library of Medicine, Honduras, Facultad de Ciencias Medicas, Universidad Nacional Autonoma de Honduras, Tegucigalpa, Honduras; Karin Saric, Norris Medical Library, University of Southern California–Los Angeles; and Dativa Tibyampansha, Kilimanjaro Christian Medical University College, Tanzania.

We are pleased to honor the 2017 recipients: Irena G. Dryankova-Bond, Blais Family Library, Massachusetts College of Pharmacy & Health Science–Worcester; Sarah Young, Mellon Institute Library, Carnegie Mellon University, Pittsburgh, PA; Israel Mbekezeli Dabengwa, Library, National University of Science and Technology, Bulawayo, Zimbabwe; Lydia Hull Witman, Claude Moore Health Sciences Library, University of Virginia–Charlottesville; and Nguyen Hai Ha, Library and Information Center, Hanoi University of Public Health, Hanoi, Vietnam.

The purpose of the Cunningham Memorial International Fellowship is to assist in the education and training of health sciences librarians from countries outside the United States and Canada. The 2017 recipient is Tam Ha, Vinmec Medical University Project, Vinmec International Hospital SJC, Vingroup, Hanoi, Vietnam. She is unable to be with us tonight.

The Eugene Garfield Research Fellowship was established to promote and support research in the history of information science in the medical or health sciences. This year’s recipient is Nicole Dalmer, University of Western Ontario–London, Canada. Nicole is unable attend the annual meeting this year.

The David A. Kronick Traveling Scholarship is awarded annually to cover expenses involved in traveling to medical libraries in the United States or Canada for the purpose of studying a specific aspect of health information management. This year’s recipient is Catherine Pepper, Medical Sciences Library, Texas A&M University–Austin.

The 2016/17 Board of Directors has named five association members as Fellows of the Medical Library Association. Fellows are chosen for their outstanding contributions to health sciences librarianship and to the advancement of the purposes of MLA. Jane Blumenthal, AHIP, FMLA, Taubman Health Sciences Library, University of Michigan–Ann Arbor, has provided exemplary leadership to the members of MLA through many years of participation in the association, including as a member of the Board of Directors and as the 2012/13 MLA president. Vicki F. Croft, AHIP, FMLA, professor emeritus, Animal Health Library, Washington State University–Pullman, is a well-respected leader in medical librarianship with great passion and commitment to MLA for over forty years at the national, section, and chapter level. Sandra G. Franklin, AHIP, FMLA, Woodruff Health Sciences Center Library, Emory University, Atlanta, GA, has served in leadership roles on numerous committees and as an MLA Board member from 2013 to 2016. Sandra is a strong advocate for librarians and is known as an honest, genuine, and passionate leader. Brenda Faye Green, FMLA, Biomedical Libraries, Dartmouth College, Hanover, NH, has demonstrated durable achievement by a sustained level of commitment to the goals of the association. Brenda has provided leadership, service, and scholarship to MLA at both the national and regional level. Paula Raimondo, AHIP, FMLA, retired, University of Maryland–Baltimore, Catonsville, MD, is a well-respected leader in medical librarianship with a great passion for mentoring. Paula is an active and committed MLA leader who also served on the MLA Board of Directors.

From time to time, the MLA Board of Directors sees that an exceptional contribution has been made to the profession and the goals of the association. This year the President’s Award honors several MLA members. First, I would like to recognize Jodi L. Philbrick, AHIP, Department of Library and Information Sciences, University of North Texas–Denton. Jodi stepped up to become the chair of Section Council and a member of the Board of Directors when former member Ysabel Bertolucci died suddenly in 2012. Jodi served on the Board of Directors, and at the same time, she served as president of the South Central Chapter. While active on two recent task forces, Jodi was also deeply engaged with the development of the *MLA Competencies for Lifelong Learning and Professional Success,* with her role as board liaison. Additionally, she has been strongly involved with the 2016–2017 MLA Rising Stars program. Over the past five years, Jodi has worn many different hats on behalf of MLA. With grace and intelligence, she has worked to connect people, ensuring that mutual understanding is achieved and that progress is made.

This past year, MLA members adopted and voted on amendments to the association’s bylaws. Throughout the 2015/16 year, the Bylaws Committee held regular meetings, read an incalculable amount of emails, and debated and reviewed countless versions of the amendments. The committee openly communicated its progress in the Full Speed Ahead blog and in the *MLA News.* They sought feedback from the membership by creating the Bylaws Discussion Forum and responded to inquiries about the possible impact changes would have on various members and MLA communities. Their considerable efforts showed when the bylaws amendments were adopted with 97% member approval and became effective on January 1, 2017. I would like to recognize the 2015/16 Bylaws Committee members: Ellen Brassil, AHIP, Baystate Health Sciences Library, Baystate Health, West Hartford, CT; Mary E. Helms, Strategic Initiatives, McGoogan Library of Medicine, University of Nebraska Medical Center–Omaha; Andrea C. Kepsel, AHIP, MSU Libraries, Michigan State University–East Lansing; Suzanne Ferimer, AHIP, Weston A. Pettey Library, University of Houston, Houston, TX; Chris Shaffer, AHIP, OHSU Library, Oregon Health & Science University–Portland; and Patricia Thibodeau, AHIP, FMLA, retired, Duke University, Chapel Hill, NC.

The highest honor that the Medical Library Association confers on any individual is the Marcia C. Noyes Award. President Knott asked Margaret Bandy, AHIP, FMLA, recipient of the 2016 Noyes award, to introduce this year’s recipient, Jean P. Shipman, AHIP, FMLA.

President Knott concluded the awards ceremony with the following remarks: “Each year, the awards ceremony and luncheon reminds us of the outstanding accomplishments our peers have made to the profession of health sciences librarianship. It simultaneously provides the encouragement to continue striving to new levels of achievements. In recognizing these individuals, we applaud the ‘best and brightest’ in the field.”

## BUSINESS MEETING

The Business Meeting was held on Tuesday, May 30, 9:00 a.m.–10:25 a.m. President Teresa L. Knott, AHIP, welcomed everyone to the meeting and asked Betsy L. Humphreys, FMLA, to join her at the podium.

President Teresa L. Knott, AHIP: As many of you know, Betsy L. Humphreys, FMLA, will retire on June 30 after more than forty-four years of service at the National Library of Medicine (NLM). The MLA Board of Directors has adopted a resolution honoring Betsy for her many years of outstanding leadership to health sciences librarianship, NLM, the nation, and the world. It is my privilege to read the resolution:

*Whereas,* Betsy L. Humphreys, Fellow of the Medical Library Association, has served as deputy director of the National Library of Medicine since 2005 and as acting director from April 2015, to August 2016;

*Whereas,* Betsy L. Humphreys has furthered the mission and the goals of the Medical Library Association to foster excellence in the professional practice and leadership of health sciences library information professionals in order to enhance the quality of health care, education, and research throughout the world;

*Whereas,* Betsy L. Humphreys has furthered MLA’s goal to support and advance health information research and evidence-based practices in health care by sharing her extensive knowledge and expertise while serving as a member of the research task force and through dedicated support of the Donald A. B. Lindberg Research Fellowship;

*Whereas,* Betsy L. Humphreys has advanced MLA’s goal to position itself as an open access organization through oversight of the implementation of the National Institutes of Health (NIH) public access policy and the successful transfer of MLA’s scholarly publication, the *Bulletin of the Medical Library Association,* launched in 1911 and now known as the *Journal of the Medical Library Association*, into digital format, for which she received MLA’s 2004 President’s Award;

*Whereas,* Betsy L. Humphreys has tirelessly, through the National Network of Libraries of Medicine (NNLM), advanced the progress of medicine and the improvement of the public health, provided leadership and advocated for funding to health sciences libraries and their efforts to bring health information to all who need it, including all types of libraries, health professionals, consumers, and community-based organizations across the United States and rural, inner city, suburban, and underserved areas;

*Whereas,* Betsy L. Humphreys has helped pioneer the development of health data standards, serving as the US member and founding chair of the General Assembly of the International Health Terminology Development Standards Association;

*Whereas,* Betsy L. Humphreys has diligently and authoritatively led the development of the Unified Medical Language System project, which provides tools to manage resources that improve the recall and precision of library resources for searchers throughout the world, empowers health professionals to make better decisions, and improves efficiency in health care processes by producing knowledge sources that support advanced processing, retrieval, and integration of information from disparate electronic information sources;

*Whereas,* Betsy L. Humphreys has steadfastly advocated and exemplified throughout her career her belief in the enduring value of the health sciences library profession through her support in developing highly effective leaders both at NLM and in our libraries by fostering and strengthening a role for NLM through its informatics scholarships, NLM associate fellowship program, NLM/Association of Academic Health Science Libraries (AAHSL) leadership fellows program, minority recruitment, and career development programs for health sciences librarians and information support;

*Whereas,* Betsy L. Humphreys has demonstrated vision and leadership that ensures the relevance of libraries and librarians into the future by building bridges with colleagues serving in librarianship, informatics, medicine, and public health fields;

*Whereas,* Betsy L. Humphreys has elevated MLA with masterful and insightful delivery of her 2001 Janet Doe Lecture, “Adjusting to Progress: Interactions between NLM and Health Sciences Librarians, 1961–2001,” and has received MLA’s highest honor, the Marcia C. Noyes Award, in 2007;

*Whereas,* Betsy L. Humphreys has generously motivated, warmly encouraged, and graciously inspired health sciences librarians at NLM and across the vast network of medical libraries to effectively achieve their own goals and aspirations;

*Whereas,* Betsy L. Humphreys has provided outstanding leadership while serving as acting director of NLM, continuing to promote NLM’s mission by ensuring that health professionals and the public have continued support and access to vital information resources, resulting in a flawless transition between NLM directors;

*Therefore,* be it resolved, that the Medical Library Association declares Betsy L. Humphreys an inspiration to her profession and a national treasure;

And further resolved, that the Medical Library Association commends Betsy L. Humphreys for over forty-four years having extraordinary leadership, service, and championing the profession of health sciences librarianship.

Congratulations.

Betsy L. Humphreys, FMLA: Thank you all very, very much. I think I will be extremely brief, given this long introduction to my career. It has been a terrific pleasure to work with all of you. Everything that I have accomplished has been accomplished through eons with many, many different people in many different disciplines, but none have been more supportive and better colleagues and worked any more effectively with me than the members of the Medical Library Association and everyone in our profession.

I admire you, and I feel exactly the same way Mary Lindberg did when she first started coming to MLA meetings, when Donald A. B. Lindberg became director of NLM, and she said to me, “Betsy, it’s much better meeting with librarians than with pathologists.” And allow me to say that when I negotiated the SNOMED deal, I came to know what she meant.

Anyway, thank you very much. It has been a great pleasure being part of this group, and it is a group I’m very proud to be a member of. Thank you.

President Knott then asked Kevin Baliozian, executive director, to make introductions and announcements. Mr. Baliozian presented the members of MLA’s 2016/17 Board of Directors: President Teresa Knott; President-Elect Barbara A. Epstein, AHIP, FMLA; Immediate Past President Michelle Kraft, AHIP; Treasurer Chris Shaffer, AHIP; Secretary Lisa K. Traditi, AHIP; Chapter Council Chair Melissa Ratajeski; Section Council Chair Jodi L. Philbrick, AHIP; and Directors Amy Blevins; Keith W. Cogdill, AHIP; Melissa De Santis, AHIP; Heidi Heilemann, AHIP; and Melissa L. Rethlefson, AHIP. He went on to introduce appointed officers, editors, and coordinators: incoming Parliamentarian Chris Shaffer, filling in for Patricia L. Thibodeau, AHIP, FMLA, who was unable to attend; Sergeant-at-Arms Linné Girouard, AHIP; *MLA News* Editor Cheryl Rowan; *Journal of the Medical Library Association (JMLA)* Editor-in-Chief Katherine Goold Akers; MEDLIB-L Coordinator Richard James; and Oral History Project Director Carolyn E. Lipscomb, AHIP, FMLA

Mr. Baliozian then recognized the vital role the thirteen MLA chapters play in bringing the benefits and services of the association to members at the regional, state, and local levels. He also recognized the 21 sections and special interest groups (SIGs) of MLA that provide significant networking and professional development opportunities for MLA members in their areas of specialization, allowing specific needs and issues to be addressed. He also acknowledged committees, task forces, and representatives to allied organizations for their crucial role in the success of MLA’s programs and services. He then remarked that more than 310 new members have joined MLA since the 2016 annual meeting.

President Knott then called to order the Business Meeting of the 2017 MLA annual meeting and asked if a quorum of 250 voting members required for transaction of business was present. Sergeant-at-Arms Linné Girouard confirmed the quorum, and President Knott called on Secretary Traditi to move adoption of the Rules of Assembly. Secretary Traditi explained that the Rules of Assembly include information on addressing the chair, presenting motions, debating, and voting. At the direction of the Board of Directors, she moved that the Rules of Assembly as they appear on MLANET be adopted. Voting paddles were raised, and there being a majority in the affirmative, the rules were adopted. Secretary Traditi then announced that each meeting registrant had a printed copy of the *Official Program,* and that the agenda for the 2017 Business Meeting was on page 35. She moved that the agendas be adopted. The vote was affirmative and the agendas were adopted.

President Knott announced that the ballots for the 2 MLA elections for 2017/18 president-elect, Board of Directors, and Nominating Committee were sent electronically or by postal service to all 2,733 eligible voting members of the association. For the first election, 877 valid ballots were returned, with a participation rate of 32%. Election results were announced in the December 2016 issue of MLA-FOCUS. For the 2nd election, 748 valid ballots were returned, with a participation rate of 27%. Complete election results, including vote totals, are published in the 2016/17 Annual Report, which is available on MLANET.

Election results: President-Elect: Beverly Murphy, AHIP, FMLA; Nominating Committee: Heidi Sue Adams, AHIP; Kristine Alpi, AHIP; James Bothmer, AHIP; Diana Delgado, AHIP; Ayaba Logan, AHIP; Fatima M. Mncube-Barnes; Katie Prentiss, AHIP; Peace Ossom Williamson; and Michael A. Wood; Board of Directors: Marie Tomlinson Asher; Stephanie Fulton, AHIP; and Sandra I. Martin, AHIP.

President Knott announced that earlier this year, the Board of Directors revised the election calendar. Going forward, elections for the president-elect, directors of the MLA Board, and members of the Nominating Committee will be held in February instead of the prior November, with all terms rotating at the Business Meeting. This will shorten the work of the Nominating Committee from sixteen months down to twelve within the same association year from June to May. Elected individuals will be notified by the end of February in time to plan for the annual meeting, but they won’t have to wait as long for the start of their terms.

President Knott then called on Treasurer Chris Shaffer to present the treasurer’s report.

Chris Shaffer, AHIP: So this is my final treasurer’s report. I am happy to announce that in 2016, we had revenues of $2,763,213 and expenses of $2,748,535, resulting in a net profit of $14,678, which exceeded our original budget projections.

Our 2016 revenues were affected by soft revenues and weakening of our institutional dues and continued reductions in print ad sales—actually, a precipitous decline in print ad sales for the *MLA News* and *JMLA.* But through careful stewardship by the board and in particular by the MLA staff—Ray Naegele, Kevin Baliozian, and others—we were able to fully offset those expenses. We had significant savings in payroll and office expenses, and reduced board travel by having various members of the board present at chapter meetings in place of the president; and by using the new social platform for the MLA ballots, saved us considerable money, which allowed us to end in the black.

In November of 2016, we approved our new board business plan with a new institutional membership model, which has been extremely successful. Ten percent of our members were able to take advantage of the discounts provided by institutional memberships, which far exceeded our expectations and will help with our finances going forward.

We shifted to a digital *MLA News* and *JMLA* with a print opt-in for an additional fee. A surprising number of you took that print opt-in, more than we expected. But at any rate, this also resulted in savings. And this allowed us to make significant new investments in education programs, including the new MEDLIB-ED—which I hope many of you have checked out; if you haven’t, please do so—and continued webinars, which have been very popular.

Our 2017 budget was adopted this past fall, with revenues of $2,811,850 and expenses of $2,805,895, with a net revenue projected of $5,955. I have every expectation that the staff will do a great job and meet that budget again.

In 2016, we accepted an unrestricted donation from the Health Web Board of Trustees. Health Web was one of the very first projects I ever worked on in the Medical Library Association and in the Midwest Chapter back in the 1990s, when we were trying to do a Yahoo for the web. Times have changed. But at any rate, they were very gracious in donating their remaining funds to the Medical Library Association.

We also received an unrestricted donation from the Estate of Gertrude Lamb, which we very much appreciate.

And we established an awards and grants endowment task force, which is evaluating how we manage our grant funds and our strategic priorities for those. That task force continues its work through this coming year, and we expect some recommendations from them.

We also approved the recommendation from Consumer and Patient Health Information Section (CAPHIS) to elevate the Consumer Health Librarian of the Year to an MLA award. We are still seeking funds. If you stop by the scholarship booth, we’re seeking funds to fully endow that award, and I strongly encourage anyone who is in support of consumer health, which should be all of us, to consider making a donation so that we can begin giving that award as a national award.

And we also accepted a fundraising request from the Hospital Libraries Section.

And I’d like to thank you very much. It has been a privilege to serve as your treasurer for these past four years. But I’m not going away. I’ll be sticking around as MLA parliamentarian. I just love bylaws so much. And Amy Blevins, who is behind me here, will be your new MLA treasurer as of about thirty minutes from now.

For more information, we do publish our budget in the *MLA News.* The annual audit report, which we expect to receive this week, will be published on MLANET; log in is required. You can contact me or Ray Naegele with any questions. And even though I’m leaving, I’ll be happy to answer questions going forward. But as of June first, you can also contact Amy Blevins, and her contact information is here.

Next, President Knott called on Executive Director Kevin Baliozian to give the executive director’s report.

Kevin Baliozian: Thank you, Theresa. So, as I mentioned earlier, this is my third meeting, and I’m recognizing a lot of faces, which is a good feeling. I’m starting to feel part of the family. And I fondly remember the first time I came on this podium. I was a little clueless. And then I came the second year, last year, my one-year anniversary, and I had a general sense of panic of just the enormity of the task. And now we’re in 2017. So the question, of course, on all of your minds, is, what is he going to show us in 2017?

Well, job security is a big part of what I’m seeking, and so you’ve seen me walk around as a photographer. I’m doubling up, apparently, also as an entertainer, so please enjoy.

So, absolutely—please congratulate your MLA Board of Directors. Your incoming Board of Directors was also part of the mischievous singing here.

All right, so a reminder: This is the MLA executive director’s report, so I’m going to switch it up a little bit and provide you with some research and do an evidence-based presentation in the next few minutes with facts. We provide the data. You can actually check the facts and reproduce the results.

So, a couple contrasting things: I think membership has actually gone down—and I’ll give you a few stats here—and it continues to go down. But the part I’m really going to be focusing on is the really magic and increasing engagement in all of you in MLA activities. So even though we’re seeing a small attrition—maybe fifty members net per year—we’re seeing a really significant increase in engagement on many different metrics, which I’ll share with you.

We’ll just spend one minute on the not-so-good news of membership easing down, which really reflects what’s happening in your own institutions and the fact that some of you—and maybe some of you not at this meeting now—have lost their jobs or their retirement positions aren’t being filled. So we’re a mirror of that.

As Chris mentioned, we have done very well in stopping the decrease of institutional memberships and making membership more affordable in several ways, and one of them is for people who are working for institutions that took on that institutional membership program and getting the $50 discounts. The board also decided to expand some of the programs for reduced membership as well. So it’s always a balance between that and MLA needing to get funds. Membership revenues represent 22% of our revenues only, so we have to find the remainder, of course.

Academy of Health Information Professionals (AHIP) credentialing: These are impressive increases year-to-year. Really, we’re talking about 70 or so increase just in little over a year. And 35% of members are academy members, which is actually quite high. I mean, I know we’d all love it to be 100% and I suggest that the remaining 65% be engaged in the program. But that’s a very high engagement number.

How many members are at least in 1 SIG or 1 section? Any guesses? 50%? Well, 75%. We ask people to participate. I know at the new member, we certainly suggest that that happens, and it is happening. Seventy-five percent of academy participation is very high. Let’s make it 100%. But 75% is good. And when you do participate, you participate in between 2 to 3 communities—2 and 1/2 communities. You can’t join a half-community; that’s an average. That’s my attempt at humor.

Members can volunteer in at least one committee or task force as section leaders, SIG conveners, or allied representatives. So we’re not counting in here people who participated at the chapter level. We’re just looking at the MLA headquarters level and the programs we have. That is a staggering 25%. Having been involved in other associations, those are amazing numbers.

That a quarter of you actually are actively volunteering for the organization—and that’s not counting chapters—is huge. That’s 642 of you. There were 227 applications this year, because some of your appointments are multiyear. There are about 8 to 10 individuals for whom we haven’t yet found appointments. Trust me; we will. That’s coming. There are openings all the time. So anyone, frankly, who wants to participate, we find a spot. It’s a 30% increase in number of applicants this year. That’s about 60 more people who chose to participate in juries or committees or task forces this year, which is just phenomenal.

Members who include their expertise area in their profile. This is 45%. And it was less than half of that last year. And so you’re making it easier for other people to find you, and that’s great. It would be great if it were higher, but close to 50 is—people spending time doing that, that’s great.

Members who are available as mentors: That’s over 21% of you. So for people who are looking for mentors, there are people out there who clearly are making the time available to support you in whatever your needs may be. And we have a database that allows you to easily find that, so please take advantage of it. And thank you for all of you who are making yourselves available.

So, now, a few metrics on site logs. And Chris mentioned a move to the *MLA News*—going digital—in his treasurer’s report. You can see how that spurred an increased—that’s good news—of people going to blogs. Now the *MLA News* is in blog format. So these are all three-month intervals, so you see that significant increase in the first quarter following the launch of the *MLA News* mid-January, and we expect that to continue.

Just one word on the *MLA News:* It’s now a personalized experienced depending on what you’re a member of, this section or another for example. Or if you’re a member or a nonmember, your experience in the *MLA News* is going to be different; it’s going to give you the content that you have access to. So that’s why it’s an interactive, personalized experience.

MLANET access we launched in 2015. We have the last third of 2015 showing a slight increase, significant increase in 2016. And you can see with a third of the year, we’re above half of the accesses we had last year. That’s an over 50% increase year-to-year. That’s kind of a geometric increase. We’ll see what it ends up being. But that’s very good news. People are coming to our content; you’re participating and using MLANET; and the numbers show that.

This is very exciting because we’re moving to education, which, if you hadn’t realized, is my favorite topic in the world. And this is just one metric on the average attendance for life webinars. So this is the life webinar part. And we extrapolate, because we sell site licenses, we also sell individual licenses to come up with comparable numbers.

And this is a pretty clear graph. Over a two-year period, the number of you attending those webinars has increased. And just a lot of people to salute on this: Obviously, the Continuing Education Committee (CEC), which plans those, is doing a masterful job in increasing the interest that there is out there. All of the instructors participating, the MLA staff in what we call the education department. That’s two people. That would be Debra Cavanaugh and Barry Grant.

We’ve also increased our marketing efforts as well. And this is all pre-MEDLIB-ED. Just imagine how we’re ramping up our success before we have our learning management system (LMS). This is before the LMS and before we launched our other content. So this is very exciting—that we have that increase. The percentage signs apparently didn’t make it from Mac to PC, so we’ll just have to extrapolate those.

Continuing education (CE) revenue, which is one way of looking at general increases, you can see that we were hovering between $150,000 and $200,000 in 2013, 2014, 2015. In 2016, we really had a very good increase of our revenues. And that shows, of course, usage. So it’s kind of aggregate usage. Already in April, you can see where we’re at. We’re going to blow through our conservative budget in 2017. And this is, again, before we launch all our new initiatives.

Our goal here is for education to go from 6% of MLA revenues to 15% and even 20% of MLA revenues, so that it will be really a 5th of our activities over the 5 years of the strategic goal. And we’re here entering Year 3, and we’re right on track. We were performing great programs, but there were a lot of gaps in our education programming. What we were doing was always excellent and continues to be excellent, but now we want to reach more audiences and be much more intentional in how we go about it, and really make education a core program that we offer to as many people as we can. So that’s very exciting.

A few stats about this meeting, if you’re curious. We have 38% of our members attending the meeting. That’s actually very high. I know we would all like 100% of our members to come to the annual meeting, but 38%, close to 40% of members attend, that’s great. We have a 1,031 paid attendees and about 1,500 to 1,600 actual total attendees, if you count vendors and invited speakers and such.

We actually have more people in the United States this year, a few are from Canada and international. I remind you that last year we had a triple joint meeting with our Canadian association colleagues, as well as with international organizations, so that’s not unexpected. Probably, if we hadn’t scheduled this meeting on Memorial Day, we probably would have had higher numbers as well. And I promise this is not happening in the foreseeable future. You’ll be able to be with your families.

Four hundred ninety-three abstract submissions. Last year, we had 540 or so, but last year was also a joint meeting. There were 150 or so from Canadians. So this is actually an increase, frankly, of our submissions, if you compare comparables. Seventy-three percent of them were accepted, and you can see the detail between papers, posters, lightning talks, and special content sessions. There continues to be a very, very robust interest in presenting, and thank you very much.

I’ve noticed a lot of smiles, and people happy to be here, going around. And I wanted to share a few of those smiles with you, so a couple slides here of people accepting me taking pictures of them. This is my favorite. I think I’m going to start a band. Here you see the board. So if you want to see all the slides, come tonight to the party, because we have a couple hundred photos that will be rotating as well, so that should be fun.

And I just want to leave you on a note of, of course, dream, dare, and do. And the last thing I want to say is, make sure you record your personal story about how you can make a difference in specific competencies, and take those competencies home and at the office and make great use of them. Thank you.

President Knott returned to the podium and announced that, in late-breaking news from Chris Shaffer, “We’re happy to say that the Consumer Health Librarian of the Year award has been fully funded through your generous donations.”

She then moved on to the annual report. In the interest of time, annual reports were received in a block. The informational reports of the appointed officials, the councils, committees, task forces, representatives, sections, and chapters are found in the 2016/17 Annual Report of the Medical Library Association. These reports are available on MLANET and will remain there throughout the year. They are also available in paper copy from the executive director’s office by request. There being no corrections or objections from the members, the reports were filed as presented.

She then recognized and thanked retiring MLA Board Members Melissa De Santis, Heidi Heilemann, Jodi Philbrick, and Chris Shaffer and presented them with certificates as a token of respect and gratitude for work well done. President Knott also expressed her gratitude to Michelle Kraft, MLA president during the 2015/16 association year. Highlighting some of Kraft’s initiatives, Past President Kraft was presented a crystal gavel for a job well done.

President Knott then introduced the new members of the MLA Board of Directors: Beverly Murphy, president-elect; Marie Ascher, director; Stephanie Fulton, director; Sandra I. Martin, director; and Elizabeth R. Lorbeer, AHIP, director.

Barbara Epstein then presented outgoing President Knott with the Presidential Cup and congratulated her for a year when under her leadership MLA broadened its opportunities to build its future.

President Knott next introduced 2017/18 MLA President Barbara Epstein who delivered her inaugural address.

## INAUGURAL ADDRESS

Barbara A. Epstein, AHIP, FMLA: I feel like the cheese stands alone up here. Thank you for those kind words, Teresa. And I want to thank you again for your mentoring and support during the past year while I served as your understudy.

One of the favorite quotes that I have stuck up on my refrigerator is by a woman named Betty Bender. And she says, “Anything I’ve ever done that ultimately was worthwhile initially scared me to death.” And that has been true for me in most of the things I’ve undertaken, and that was very true when I became your president-elect last year in Toronto.

But then I have another quote that inspires me that says, “Don’t be afraid of your fears. They’re not there to scare you. They’re there to let you know that something is worth it.” And this past year has been an eye-opening experience for me. The energy and enthusiasm of the Board of Directors and our hardworking executive director and headquarters staff was so exciting. I was impressed by the sense of teamwork and focus on the ambitious goals that we set.

We are an association of volunteers, and most of us have demanding day jobs. And I am so grateful for the literally hundreds of members who find the time to serve on our committees, task forces, juries, panels, editorial boards, chapters, sections, and special interest groups (SIGs), and to tweet and to blog and to email. Without you, nothing would happen.

So before I talk about what we hope to accomplish before we meet again next year in Atlanta, I want to take just a moment to review how our organization works and how things get done in MLA, because we think we know it, but sometimes we forget, so this is a little review.

Our organization starts with you, the 3,200 members. That’s probably a little optimistic, but we’ll get it up there. You join chapters and sections and SIGs. You may be appointed to a committee, a task force, or jury by the president-elect. You elect a Nominating Committee each year that develops a slate of candidates for board members and a president-elect.

The twelve-person board hires and evaluates the executive director, who in turn manages the headquarters staff. The board sets policy and makes sure our resources are well managed. Each board member is assigned as a liaison to several committees or task forces. The chairs of Section Council and Chapter Council serve on the board and ensure that the concerns of these groups are reflected.

The board has two in-person meetings and two virtual meetings each year. The Executive Committee includes the president, president-elect, and immediate past president, the treasurer, and the executive director. And we have a video conference every other week to make sure that the organization’s business is running smoothly.

But I want to stress that the board’s most important job is to develop a strategic direction for the association. If you’ll allow me a sports analogy, I will quote Wayne Gretzky, one of greatest hockey players of all time, even though he wasn’t from Pittsburgh. And he was asked, “Why are you so good? What’s the secret to your success?” And he answered, “It’s really not that complicated. I just skate to where the puck will be.”

So if we’re to be successful, our job is to figure out where the puck is going and to be ready for it when it gets there. It sounds easy, but it’s not.

On Sunday, Theresa outlined the many accomplishments achieved during her presidency in the past year, and they are indeed impressive. In the rest of my talk today, I will outline our most important goals for the coming year that will hopefully bring us to where the puck will be when we meet again in Atlanta next year to adapt, to transform, and to lead.

These include initiatives in diversity and inclusion, education, research, and a new program called Corporate Partners. And I’ll talk about that last, so you’ll have to stay. Last week, the board approved a new strategic goal to ensure that diversity and inclusion are reflected as core values in our activities, our programs, our publications, and our statements.

Why are we doing this? What do we hope to accomplish? Well, we want to attract a diverse community of members who feel comfortable and welcome in our programming and our activities, and we want to provide tools for our members to enhance their skills in this area and to embed these values in their professional activities in their home institutions.

You will note that diversity and inclusion are detailed in our newly adopted professional competencies as an indicator under leadership and management. At the basic level, we should be able to describe our own cultural backgrounds and to recognize biases; and to value cultural norms, experiences of others, and expressions of diverse viewpoints; and to recognize power dynamics in relationships at an expert level, which I hope we will all become. We should be able to develop and implement practices that foster diversity and equality, contribute to correcting inequalities, and participate in external collaborations.

This summer, I will appoint a task force to lead the initiative and coordinate activities throughout MLA. The task force will reach out to our membership via community sections, SIGs, open forums, and other means as they recommend action items.

As an association, we have already started along this path. Several chapters have task forces, conduct surveys, have committees, and sponsor programming, and this meeting includes several highly relevant programs. The diversity initiative task force will be appointed for two years, but as I said in my *MLA News* column, it is my hope that this will not be a one-and-done activity; that instead, we will continue to consciously embed these values into our programs and activities as an ongoing effort.

Next, I want to talk about research, and especially to commend the Research Imperative Task Force for their impressive accomplishments this year. It is chaired by Susan Lessick, AHIP, FMLA, and the rest of the members are also listed on my slide.

First, and this is a first, they submitted a successful Institute of Museum and Library Services (IMLS) grant proposal, and they were awarded $239,000 for MLA to create a research training institute. Their goal is to ensure that an increasing number of health sciences librarians will have the research skills required for their work in the 21st century. The institute will be a yearlong experience for a cohort of 20 participants a year. It will have a 1-week face-to-face workshop and then follow-up with individual mentoring, a research project, post-workshop support, and membership in an online research community of practice. And I’m happy to say that there will be many scholarship opportunities for participants.

Second, on the task force recommendation, MLA will offer a new reward for research advancement in health sciences librarianship, beginning with the 2017/18 award cycle. And the purpose is to recognize organizations that foster librarian-led research. I will appoint an award jury early this summer, and criteria for this award will be widely distributed.

And third, new web pages on MLANET are in the final stages of development. The first of these, shown here, will highlight research, resources, tools, and aids to facilitate research engagement for health sciences librarians at all levels of experience. Another will identify impact studies that demonstrate the value and benefits of health sciences librarians. And there are a lot of these studies available, and I urge you to take a look at them and incorporate them into your activities.

You’ve heard about the work that we’ve done to advance our focus on education and professional development and to position MLA as the go-to education resource for health information professionals. In the coming year, we will implement our new committee structure to guide educational programming. Instead of one overworked Continuing Education Committee as in the past, we have an integrated structure with the Education Steering Committee as the coordinating body, the Education Annual Programming Committee to select and plan course offerings for the annual meeting, and webcasts and webinars throughout the year. The Education Leadership Curriculum Committee is the first of what will be several operational committees to develop curricula and oversee course development in specific subject areas. The next committee, to be appointed soon, will be the Research and Evidence-Based Practice Curriculum Committee—they’ll have to come up with an acronym—whose first job will be to develop the curriculum for our IMLS-funded research institute.

The Communities Strategic Goal Task Force, under the leadership of Rikke Sarah Ogawa, AHIP, has been working diligently through the past year. Their charge is to strengthen our member communities, our sections and SIGs, and other affinity groups by analyzing and recognizing changes in the community architecture and roles. Their aim is to increase the relevance and effectiveness of these groups to maximize the value and visibility of member content and to reduce administrative complexity and thereby attract and involve more members.

After a great deal of thoughtful discussion, the task force came up with a draft of guiding principles to serve as an overarching set of common ideas of what MLA communities of interest are supposed to be and do. These guiding principles specify that communities should provide a valuable organizational home to their members and that they’re organized to embody the MLA mission. They should be organized efficiently, they should be judicious in their use of finite member and staff resources, and they should be organized to retain existing members and attract new ones.

If you would like to learn more about this initiative and to share your ideas, you are invited to attend an open meeting tomorrow morning, and the time and place are listed on the slide. And the task force expects to submit their report to the board sometime during the coming year.

So the last program I want to discuss is the Corporate Partners program, which is another new venture for MLA. It started with an exploratory meeting last year in Toronto and has continued with a small working group that came up with an outline that was refined through conversations with several of MLA’s corporate supporters.

Their aim is to provide a forum for MLA experts and MLA corporate partners to engage in high-level, high-value dialogue that furthers our opportunities to learn, inform, come together, and influence each other on issues of common interest that impact the profession. And the ultimate goal is to strengthen MLA’s position as the thought leader for our profession.

Several of these publishers have already expressed strong interest in participating and have indicated willingness to commit funding to support the program. On Thursday, the board approved a two-year pilot that will be led by a corporate partners task force. The program format will be to support two in-person summits each year for about thirty to fifty invited participants, equally divided between library experts and publisher representatives.

Discussion at these meetings will center around issues and topics that impact our professional communities; not dealing with things like pricing models and subscription costs, but really trying to look at the fundamental issues that we’re all wrestling with. While discussion among the partners at the meeting may be confidential, the agenda, the participant lists, and the executive summaries will be public, as will the outcomes of those meetings.

The summit themes, issues, and topics of discussion are not set in stone yet but will be developed consensually by these groups. Several possible themes are listed on this slide, and we appreciate that not every topic will be of interest to every librarian or every corporate partner, but this is a start.

Depending on the topic, possible outcomes might include a white paper about challenges and opportunities, articles in academic or lay literature about the event and its findings; it may be educational strategies for our members or the publisher community; action plans to invest in or cocreate informational content; or a marketing campaign, like public service announcement, press releases, or social media.

And I want to stress that this is a new program and it is very much a pilot. And hopefully, when I report to you next year at this time, the program will have gained speed and taken off.

Before I close, I want to recognize a very special MLA member and fellow, and that’s Lucretia W. McClure, AHIP, FMLA, and I think she has already been recognized in several meetings and forums that have already occurred. As you know, she is the retired director at the Edward G. Miner Library at the University of Rochester. And during her career she won the MLA Triple Crown: She’s a past president, a Doe lecturer, recipient of the Marcia C. Noyes Award, and probably most of the other awards that we have.

Recently, she was honored again in a very special way. When she moved to a senior living environment, she took responsibility for the residents’ library, and of course, it was a mess. But very soon, she helped her residents win a grant for $75,000, and she reorganized the library. And so it’s a well-functioning entity, and it has larger space, more shelving, fresh paint, and a new red carpet. And in her honor, the library was named the Lucretia McClure Library at the Valley Manor Life Community, with a grand opening earlier this month.

But I show this picture to remind us that we all have special contributions to offer throughout our careers and even beyond. And we all have different styles. Whether we’re working on a committee, designing a project, teaching a class, or just picking strawberries in the Georgia sun, we have different ways of getting there. Some of us are meticulous and organized, and others are unfettered and creative. But working together, we can gain speed, take off, and accomplish great things.

I thank you for this honor and the opportunity to serve as your president.

At the conclusion of her inaugural address, President Epstein asked David A. Nolfi, AHIP, and Debra Berlanstein, AHIP, of the 2018 National Program Committee (NPC) to come to the podium to give the official thank you for the 2017 annual meeting.

David A. Nolfi, AHIP: Hello, Debbie.

Debra Berlanstein, AHIP: Hello, David. And hello, MLA. Hasn’t this been a great meeting?

David Nolfi: As Barbara said, we are the cochairs of the 2018 NPC, and we’re here to introduce a resolution to thank all the planners of the 2017 annual meeting.

Debra Berlanstein: Is it just me or is there a lot of alliteration associated with MLA annual meetings?

David Nolfi: Hmmm…dream, dare, do. This year’s NPC cochairs were Debbie and Donna [Deborah Sibley and Donna Berryman, AHIP].

Debra Berlanstein: I doubt they did it deliberately. Doing alliteration by design would be daft, disconcerting, and desultory. Only a couple of dullards would do that.

David Nolfi: You might have a point. Maybe we should move on to the resolution.

Debra Berlanstein: Definitely. *Whereas,* the National Program Committee exhorted us all to dream of a brighter future for our libraries and our profession and help to make those dreams a reality by putting together an array of awesome speakers and awe-inspiring programs;

David Nolfi: And *whereas,* the Local Assistance Committee dared us to experience the energetic, enigmatic, and exciting city of Seattle with its diverse attractions. From the cultural to the gastronomical, to the natural to the arboreal to the geographical to the nocturnal, they dared us to acculturate, told us here to perambulate, masticate, caffeinate until we hallucinate.

Debra Berlanstein: Okay, I’m going to stop you right there.

David Nolfi: And *whereas,* the MLA staff made sure that everyone on the NPC and Local Assistance Committee did what they needed to do and provided their expert, thoughtful, and professional guidance to make this year’s annual meeting run smoothly.

Debra Berlanstein: And *whereas,* the exhibitors and sponsors have not only helped to make the meeting more meaningful, but also partnered with us to provide effective solutions that help us and our clients do our work.

David Nolfi: Therefore, be it *resolved* that the membership of the Medical Library Association extends its profound appreciation and sincere gratitude to the 2017 National Program Committee, Local Assistance Committee, sponsors, exhibitors, MLA staff, and presenters for an excellent meeting in the beautiful city of Seattle.

Debra Berlanstein: And thanks to all of you who dream, dare, and do.

The resolution was adopted by acclamation. President Epstein then recognized Lisa Traditi, 2017/18 secretary of the MLA Board of Directors, who moved to adjourn the meeting. The motion carried, and the business meeting of the 117th annual meeting was officially adjourned.

Next, Sandra G. Franklin, AHIP, FMLA, and Joe Swanson Jr., cochairs of the 2018 Local Assistance Committee, invited members to attend the 2018 annual meeting in Atlanta.

## PROGRAM SESSIONS

Section programs were presented in 4 time slots: Sunday, May 28, 3:00 p.m.–4:25 p.m.; Monday, May 29, 10:30 a.m.–11:55 a.m., and 1:00 p.m.–2:25 p.m.; and Tuesday, May 30, 3:00 p.m.–4:25 p.m. Paper abstracts that were scheduled to be presented are available on the MLA ‘18 website. The final version of the abstracts reflecting only those presented at the meeting is included as an online-only supplemental file to the January 2018 issue of the *Journal of the Medical Library Association.*

## POSTER SESSIONS

Poster sessions were presented in 4 time slots: Sunday, May 28, 2:00 p.m.–2:55 p.m.; Monday, May 29, 2:30 p.m.–3:25 p.m.; Tuesday, May 30, 1:00 p.m.–1:55 p.m.; and Tuesday, May 30, 2:00 p.m.–2:55 p.m. Poster abstracts that were scheduled to be presented are available on the MLA ‘18 meeting website. The final version of the abstracts reflecting only those posters presented at the meeting is included as an online-only supplemental file to the January 2018 issue of the *Journal of the Medical Library Association.* The actual posters are available online in the MLA ‘18 meeting website.

## OTHER MEETINGS AND EVENTS

### Pre-meeting activities

On Thursday, May 25, the MLA Board of Directors met. The MLA Board of Directors and Credentialing Committee met on Friday, May 26. On Saturday, May 27, the following groups met: 2018 National Program Committee; 2018 program planners; Chapter Council; Communities Strategic Goal Task Force; Joint Section Council/Chapter Council; National Network of Libraries of Medicine (NNLM) Steering Committee; Nominating Committee; and Section Council.

### Sunday, May 28, 2017

On Sunday, May 28, these groups, sections, and SIGs met: Ad Hoc Committee to Review Core Clinical Journals; African American Medical Librarians Alliance SIG Business Meeting; chapter treasurers orientation meeting; Complementary and Alternative Medicine SIG Business Meeting; Consumer and Patient Health Information Section (CAPHIS) Executive Committee Meeting; Education Steering Committee; Federal Libraries Section Business Meeting; Fellows of MLA; Gaming in Adult Learning SIG Business Meeting; Health Disparities SIG Lunch; Information Literacy in Medical Education (ILME) SIG Business Meeting; International Cooperation Section Business Meeting; *JMLA* Editorial Board; Leadership Curriculum Committee; Librarians without Borders®/Elsevier Foundation Research4Life Grants: Sharing Best Practices 2016–2017; MLA-Philadelphia Merger Discussion Update; Outreach and Marketing SIG Business Meeting; Pediatric Librarians SIG Business Meeting; Public Health/Health Administration Section Business Meeting; Research Section Research Award Judging Meeting; Resource Sharing SIG Business Meeting; Sewell Fund Informational and Networking Meeting; and Translational Sciences Collaboration SIG Business Meeting. Exhibitor meetings included: EBSCO Medical Librarian Luncheon and the *JoVE* Library Advisory Board.

### Monday, May 29, 2017

On Monday, May 29, the following groups, sections, and SIGs met: 2019 National Program Committee; Association of Academic Health Sciences Libraries (AAHSL) Scholarly Communications Committee; Awards and Grants Endowment Task Force; Awards Committee; Books Panel; Clinical Librarians and Evidence-Based Health Care SIG Business Meeting; Consumer and Patient Health Information Section (CAPHIS) Business Meeting; Data SIG Business Meeting; Dental Section Business Meeting; Department of Veterans Affairs Librarians SIG Business Meeting; Education Steering Committee, Education Annual Programming Committee, and Leadership Curriculum Committee Joint Meeting; Governmental Relations Committee; Health Association and Corporate Libraries Section (HACLS) Business Meeting; History of the Health Sciences Business Meeting; Hospital Libraries Section (HLS) Executive Board Meeting; Hospital Libraries Section Member Reception and Business Meeting; Institutional Animal Care and Use SIG Business Meeting; Interprofessional Education (IPE) SIG Business Meeting; Latino SIG Business Meeting; LGBTQ Health Science Librarians SIG Business Meeting; Libraries in Curriculum SIG Business Meeting; Medical Library Education Section (MLES) Business Meeting; Medical Library Group of Southern California and Arizona Business Meeting; Molecular Biology and Genomics SIG Business Meeting; New Members SIG Business Meeting; New York-New Jersey Chapter Business Meeting; Nursing and Allied Health Resources Section (NAHRS) Business Meeting; Nursing and Allied Health Resources Section (NAHRS) Executive Board Meeting; Osteopathic Libraries SIG Business Meeting; Pacific Northwest Chapter Business Meeting; Pharmacy and Drug Information (PDI) Section Business Meeting; Professional Recruitment and Retention Committee; Public Services Section Business Meeting; Relevant Issues Section Business Meeting; Revitalizing the Research Imperative Task Force; Scholarly Communications Committee; Southern Chapter Executive Committee; Systematic Reviews SIG Business Meeting; Technical Services Section Business Meeting; Veterinary Medical Libraries Section Informal Meeting; Vision Sciences SIG Business Meeting. Exhibitor meetings included: Health Sciences Libraries to the Rescue: Bolstering the Library’s Role with Repository and Publishing Services.

### Tuesday, May 30, 2017

On Tuesday, May 30, the following groups, sections, and SIGs met: AAHSL Program and Education Committee; AAHSL Research Services Committee; Bylaws Committee; Cancer Librarians Section Business Meeting; Collection Development Section Business Meeting; Department of Veterans Affairs Librarians SIG Business Meeting; Educational Media and Technologies Section (EMTS) Business Meeting; Information Literacy in Medical Education (ILME) SIG Business Meeting; Joseph Leiter NLM/MLA Lectureship Committee; Leadership and Management Section Business Meeting; Librarians without Borders® Committee; Medical Informatics Section Business Meeting; Medical Library Group of Northern California and Nevada Business Meeting; Membership Committee; MLA community managers and webmasters; *MLA News* Editorial Board; Research Section Business Meeting; Rising Stars Program; section treasurers orientation; Systematic Reviews SIG: Competencies for Librarians Working on Systematic Reviews; Task Force to Review MLA’s Competencies for Lifelong Learning and Professional Success; Technical Services Section Social; and Veterinary Medical Libraries Section Business Meeting.

### Wednesday, May 31, 2017

On Wednesday, May 31, the following groups, sections, and SIGs met: Communities Strategic Goal Task Force; Continuing Education Committee; Grants and Scholarships Committee; MLA Board of Directors; and Oral History Committee.

## OPEN FORUM

One open forum was held on Sunday, May 28, from 4:30 p.m.–5:25 p.m.:

- Activism in a Time of Turbulence

## NATIONAL LIBRARY OF MEDICINE UPDATE

The National Library of Medicine (NLM) Update took place on Tuesday, May 30, 11:00 a.m.–11:55 a.m. Joyce E.B. Backus, associate director for library operations, began the session by introducing Patricia Flatley Brennan, the new director for the NLM and interim National Institutes of Health (NIH) associate director for data science.

Dr. Brennan talked about NLM’s core mission and the key elements of that mission: management, acquisition, and dissemination of literature, including grey literature and the latest things coming out; data resources such as the National Center for Biotechnology Information (NCBI) and GeneBank, a substrate of discovery; researchers and intramural research program that seek to discover the best ways to inquire, investigate, and disseminate literature, data, and the like; and outreach, to make sure that NLM’s resources are available around the world. Dr. Brennan reported that the NLM budget was very good and received an increase of almost $15 million in 2017 over the 2016 budget. NLM is also the hub for data science, an assignment from the advisory committee to the director of NIH, which will provide value-added services on top of the data, such as adding metadata and constructing pathways to link data.

Daniel R. Masys, cochair, NLM Board of Regents Strategic Planning Committee, and affiliate professor, Biomedical and Health Informatics, University of Washington School of Medicine, provided a brief overview of the strategic planning timeline that began in September 2016 and the four major topic areas: advancing biomedical discovery and translational science; supporting the public’s health; building collections to support discovery and health in the twenty-first century; and advancing data science, open science, and biomedical informatics. The guideline will be published in fall 2017, and implementation will begin in October 2018 with the 2019 budget year.

Amanda J. Wilson, head, National Network Coordinating Office of the National Network of Libraries of Medicine (NNLM), was formerly the director of the National Transportation Library at the Department of Transportation. She talked about new offices in NNLM that focus on DOCLINE, evaluation, public health coordination, training, and web services as well as the National Network Steering Committee and subcommittees. National initiatives include the launch of NNLM RD3: Resources for Data-Driven Discovery and work with the Public Library Association to get high-quality medical information to public librarians and get them engaged.

Ms. Backus reported on the Inaugural Michael E. DeBakey Lecture in the History of Medicine, which is archived on the NLM website; the growing digital collections; the upcoming book *Images of America: US National Library of Medicine,* an illustrated history of NLM; NLM grants; and NLM-driven data management curriculum resources. She concluded the NLM Update with a Q&A session.

## LEGISLATIVE UPDATE

The Legislative Update was held on Tuesday, May 30, from 1:00 p.m.–1:55 p.m., and was moderated by Cristina Pope, AHIP, chair, Governmental Relations Committee. Committee members provided an overview of health funding, public access, and other relevant issues.

## OTHER SPECIAL EVENTS AND RECEPTIONS

### Saturday, May 27 2017

- Welcome Reception and Opening of the Hall of Exhibits, 5:30 p.m.–7:30 p.m.

### Sunday, May 28, 2017

- MLA New Members/First-Time Attendees Program, 7:00 a.m.–8:55 a.m.
- Sunrise Yoga, 7:30 a.m.–8:30 a.m.
- Chapter Council Presents Chapter Sharing Roundtables Luncheon, noon–1:55 p.m.
- International Visitors Reception, 6:00 p.m.–7:00 p.m.

### Monday, May 29, 2017

- Academy of Health Information Professionals Q&A Session, 2:30 p.m.–3:25 p.m.
- Library School Reunion, 6:30 p.m.–7:30 p.m.

### Tuesday, May 30, 2017

- MLA Book Authors and Prospective Authors Gathering, 2:00 p.m.–2:55 p.m.
- President’s Awards Dinner, 6:30 p.m.–10:00 p.m.

## SUNRISE SEMINARS

Exhibitors held Sunrise Seminars to provide information and to introduce new products and services. The following seminars were held:

### Sunday, May 28, 2017

- DynaMed Plus Updates and Case Studies
- Using Evidence to Inform Practice: The Solution at Point of Care, Point of Reference, and Point of Learning

### Monday, May 29, 2017

- American Psychological Association (APA)
- Evidence Synthesis and Systematic Review: Premier for Medical Librarians
- An “Inside/Outside” Look at Cochrane: Update from Carol Lefebvre
- Nature Masterclasses: Supporting Researcher Development
- Nursing Reference Center Plus and Learning Management Systems
- The Value of Visualization: See VisualDx in Action

### Tuesday, May 30, 2017

- What’s New and Unique in EndNote

## TECHNOLOGY SHOWCASES

Twelve Technology Showcases were held throughout Sunday, Monday, and Tuesday:

- Approaches to Authentication
- CABI: Science, Products, and Platforms
- Clinical Discovery and the Continuing Education Evolution
- Covidence: Using Technology to Streamline Systematic Review Production
- DistillerSR and CuratorCR: A Systematic Review and Reference Management Primer
- EBSCO eBooks
- EBSCOHealth Interdisiplinary Care Content Package
- The Evolving Role of the Library in Institutional and Faculty Assessment: A Discussion of Research Metrics
- Medical Full-Text Databases
- Medical Research and the Future of Discovery
- The R2 Digital Library, a Health Sciences E-Book Database
- Welcome to the New HTML5 User Interface of Primal Pictures and Anatomy.tv

## CONTINUING EDUCATION COURSES

The 2016/17 Continuing Education Committee offered the following courses to 320 attendees on May 26 and 27, 2017.

### Friday, May 26, 2017

CE100, Engaging Assessment to Show Value and Make Decisions: Making the Case for Your Library, **Instructor:** Lisa Janicke Hinchliffe, professor and coordinator, Information Literacy Services and Instruction, University Library, University of Illinois–Urbana

CE200, Maintain Momentum in Marketing, **Instructors:** Patricia Baker, professor, Lister Hill Library of the Health Sciences, University of Alabama–Birmingham, and Valerie St. Pierre Gordon, AHIP, retired, Birmingham, AL

CE201, Leading the Way: Preparing for and Enhancing Your Leadership Role, **Instructors:** Lindsay Alcock, head, Public Services, Health Sciences Library, Memorial University of Newfoundland–St. John’s, Canada; Kelly Thormodson, interim director, Harley E. French Library of the Health Sciences, School of Medicine and Health Sciences, University of North Dakota–Grand Forks; Diana Delgado, AHIP, associate director, User Support, Research and Education, Samuel J. Wood Library, Weill Cornell Medicine, New York, NY; and Martin Wood, AHIP, assistant director, Maguire Medical Library, Florida State University College of Medicine–Tallahassee

CE300, Advanced Searching Techniques and Advanced Strategy Design, **Instructors:** Carol Lefebvre, HonFCLIP, independent information consultant, Lefebvre Associates, Oxford, United Kingdom, and Mick Arber, senior information specialist, York Health Economics Consortium, Enterprise House, University of York, York, United Kingdom

CE700, Research Design and Data Analysis, **Instructors:** Jin Wu, emerging technologies librarian, Norris Medical Library, University of Southern California–Los Angeles, and Lili Luo, associate professor, School of Information, San Jose State University, San Jose, CA

CE702, Introduction to Systematic Reviews for Librarians, **Instructors:** Margaret J. Foster, AHIP, associate professor and systematic reviews and research coordinator, Medical Sciences Library, Texas A&M University–College Station, and Susan Fowler, coordinator, Systematic Reviews, Becker Medical Library, Washington University, St. Louis, MO

### Saturday, May 27, 2017

CE101, Accelerating Innovation through Information Tools and Expertise, **Instructors:** Tallie Casucci, innovation librarian, Spencer S. Eccles Health Sciences Library, University of Utah–Salt Lake City, and Jean P. Shipman, AHIP, FMLA, librarian and director, Spencer S. Eccles Health Sciences Library, Clifford C. Snyder Endowed Chair and director, MidContinental Region and National Training Office, National Network of Libraries of Medicine, director, Information Transfer, Center for Medical Innovation, and adjunct professor, Department of Biomedical Informatics, School of Medicine, University of Utah–Salt Lake City

CE102, Your Role in Achieving Magnet Status and Continuing Support of Nursing Research, **Instructor:** Helen-Ann Brown Epstein, AHIP, FMLA, informationist, Health Sciences Library, Virtua Center for Learning, Mt. Laurel, NJ

CE202, Creating Influence: How to Get What You Want, **Instructors:** Lisa K. Traditi, AHIP, head, Education and Reference, and associate professor, Health Sciences Library, Anschutz Medical Campus, University of Colorado–Aurora, and Frank Traditi, sales learning manager, XO Communications, Lone Tree, CO

CE301, Becoming an Expert Searcher, **Instructor:** Terry Ann Jankowski, AHIP, FMLA, librarian emerita, Health Sciences Library, University of Washington–Seattle

CE302, Searching ClinicalTrials.gov, the World Health Organization Portal (ICTRP), and Regulatory Agency Websites to Identify Clinical Trials for Systematic Review and Other Clinical and Research Questions, **Instructors:** Carol Lefebvre, HonFCLIP, independent information consultant, Lefebvre Associates, Oxford, United Kingdom, and Mick Arber, senior information specialist, York Health Economics Consortium, Enterprise House, University of York, York, United Kingdom

CE303, Complementary, Alternative, and Integrative Health for the Consumer: A Primer for Librarians, **Instructor:** Kelli Ham, consumer health librarian, National Network of Libraries of Medicine, Pacific Southwest Region, UCLA Louise M. Darling Biomedical Library, Center for the Health Sciences, University of California–Los Angeles

CE304, Providing Bibliometrics Services in Medical Libraries: Why, What, and How?, **Instructors:** Ya-Ling Lu, informationist, and Christopher Belter, bibliometrics informationist, NIH Library, Division of Library Services, Office of Research Services, National Institutes of Health, Bethesda, MD

CE305, Reduce, Refine, Replace: Strategies for Animal Alternative Searches, **Instructors:** Adele Dobry, subject specialist, Carlson Health Sciences Library, University of California–Davis, and Maureen (Molly) Knapp, AHIP, training development specialist, National Training Office, National Network of Libraries of Medicine, Spencer S. Eccles Health Sciences Library, University of Utah–Salt Lake City

CE400, Perspectives in Research Data Management, **Instructors:** Alisa Surkis, translational science librarian and head, Data Services, and Kevin Read, knowledge management librarian, NYU Health Sciences Library, New York University School of Medicine–New York

CE401, Developing a Systematic Review Service, **Instructors:** Amanda Ross-White, AHIP, health sciences librarian (nursing), and Sandra McKeown, health sciences librarian, Bracken Health Sciences Library, Queen’s University, Kingston, ON, Canada

CE402, Coming to TERMS with Electronic Resource Management, **Instructor:** Christy Jarvis, AHIP, head, Information Resources and Digital Initiatives, Spencer S. Eccles Health Sciences Library, University of Utah–Salt Lake City

CE403, Open Access Publishing Models and Predatory Journals in PubMed and Beyond, **Instructors:** Carolann Lee Curry, reference and outreach librarian, and Anna Krampl, AHIP, head, Public Services, Medical Library, Mercer University School of Medicine, Macon, GA

CE500, Take Two Apps and Call Me in the Morning: Mobile Applications for Health and Wellness, **Instructor:** Emily J. Hurst, AHIP, head, Research and Education, Tompkins-McCaw Library for the Health Sciences, Virginia Commonwealth University–Richmond

CE602, How to Have Fun Teaching with Technology, **Instructor:** Molly Higgins, health sciences librarian, Health Sciences Library, Stony Brook University, Stony Brook, NY

CE703, Evidence-Based Medicine (EBM)/Evidence-Based Practice (EBP): The Basics: Study Design and Randomized Controlled Trials, **Instructor:** Connie Schardt, AHIP, FMLA, adjunct faculty, School of Library and Information Science, University of North Carolina–Chapel Hill

CE704, Practical Skills and Tips for Research: From Proposal to Presentation, **Instructors:** Skye Bickett, AHIP, assistant director, Library Services, Philadelphia College of Osteopathic Medicine–Georgia Campus, Suwanee, GA; and Christine Willis, director, Knowledge Management and Learning Resources, Shepherd Center, Atlanta, GA

CE800, Librarian’s Role in Reproducibility of Research: Keynote Speaker: Shona Kirtley, senior research information specialist, EQUATOR Network at the Centre for Statistics in Medicine, University of Oxford, Oxford, United Kingdom; Panel Speakers: Cynthia R. Hudson-Vitale, data services coordinator and research transparency librarian, Data and GIS Services, Libraries, Washington University in St. Louis, St. Louis, MO; Bart Ragon, associate director, Knowledge Integration, Research and Technology, University of Virginia–Charlottesville; Kristi L. Holmes, director, Galter Health Sciences Library, Northwestern University, Chicago, IL; and Moshe Pritsker, cofounder and chief executive officer, *JoVE (Journal of Visualized Experiments),* Cambridge, MA

## RESOURCES AND SERVICES

Sponsored by Wolters Kluwer, the online itinerary planner allowed attendees to peruse programs and events online, while the MLA ‘18 mobile app, which was sponsored by Elsevier-ClinicalKey, provided a way for attendees to save their itinerary schedules, access abstracts, message other app users, and more. Complimentary WiFi was available throughout the convention center courtesy of The JAMA Network. Live streaming conversations were available on Twitter using the hashtag #MLANET17, and volunteer bloggers, the Local Assistance Committee, and the 2016 National Program Committee contributed to the official meeting blog with meeting tips, announcements, and more. For those seeking new jobs and prospective employers, the Job Placement Center was open from Saturday through Wednesday, and the MLA Resume Clinic was available Saturday through Monday. The Hall of Exhibits was open Saturday through Tuesday.

## SUPPLEMENTAL FILES

Appendix MLA ‘17 Program Session Abstracts

Appendix MLA ‘17 Poster Session Abstracts

