# Supplementary material for: 117th Annual Meeting Medical Library Association, Inc. Seattle, WA May 26–31, 2017
Source: J Med Libr Assoc. 2018 Jan 2;106(1):E1–E28. doi: 10.5195/jmla.2018.421 (PMC5764600; doi:10.5195/jmla.2018.421)
Supplement: Appendix [file jmla-106-E1-s001.pdf]

Medical Library Association

MLA ’17 Program Session Abstracts

Any unsolicited abstracts for the annual meeting undergo a process of blind peer review. Abstracts of papers intended for session programs are reviewed by members of a panel of reviewers sponsoring the programs. The final decision on program speakers rests with the program planners.

Sunday, May 28, 2017, 3:00 PM – 4:25 PM
Room: 604

Session: "Engage for Health": A Partnership for Improved Patient-Doctor Communication

Moderator: Elaina Vitale

"Engage for Health": A Partnership for Improved Patient-Doctor Communication

Lydia Collins

Consumer Health Coordinator, National Network of Libraries of Medicine, Middle Atlantic Region, Pittsburgh, Pennsylvania

Jennifer R. Collins

Director, Public Affairs, The Hospital and Healthsystem Association of Pennsylvania, Harrisburg, Pennsylvania

Objectives: Efficient doctor-patient communication is an essential skill for individuals of all ages. Recognizing that many health care systems are moving toward the patient-and family-centered care model, the Hospital & Healthsystem Association of Pennsylvania (HAP) created ‘Engage for Health’ a tool for health literacy community education. HAP, its Hospital Engagement Network (HEN) developed ‘Engage for Health’, a series of tools to conduct a community education program on taking an active role in your health care.

In order to bring the program to the community, the HAP-HEN partnered with the Pennsylvania State Library Association (PaLA) and the National Network of Libraries of Medicine, Middle Atlantic Region (NNLM MAR) to offer this innovative program via a cohort of public libraries across the state. Learn how public libraries partnered with hospitals and health care providers to offer the ‘Engage for Health’ program at their libraries. Learn how to take the materials and replicate this program in your community.
Methods: NNLM MAR and the NNLM Evaluation Office (NEO) trained public library staff and health care providers on how to run the ‘Engage for Health’ program. In addition, cohort members learned how to establish partnerships with local hospitals and health care providers, were trained how to use consumer health resources (MedlinePlus and NIHSeniorHealth) and how to evaluate health information programs.
Results: The cohort librarians reported attendance of 150 adult participants who were part of the user populations for the 18 cohort libraries. They used a pre-post assessment evaluation form that they all helped to develop. Librarians collected evaluation forms from 132 (88%) of participants.
Key findings include:

- The program was well received, with 98% of participants rating it positively.
- The participants did report that they prepared for doctors’ visits. Before the training session, most said they sometimes or almost always researched their health conditions (78%), made lists of their medications (78%), and wrote out their health questions (68%). Almost everyone (95%) asked questions of health care providers when they didn’t understand something and 86% said they were comfortable doing so.

Conclusion: The successful ‘Engage for Health’ program is available for replication and provides an opportunity for hospital and health sciences libraries to provide outreach within their communities focusing on a topic of major importance for health consumers, in collaboration with public libraries.

Keywords: communication, patient engagement, partnerships, community outreach, doctor-patient communication, patient-and-family centered care, healthcare systems

Sunday, May 28, 2017, 3:00 PM – 4:25 PM

Room: 613

Session: #meddatalibs: A Book Group Discussion about the Intersection of Data and Medical Librarianship

Moderator: Michelle B. Bass

Patricia Gogniat

#meddatalibs: A Book Group Discussion about the Intersection of Data and Medical Librarianship

Michelle B. Bass

Population Research Librarian, Stanford University School of Medicine, Stanford, California

Objectives: Medical librarians are no stranger to data nor are the communities in which we work. However, the ways by which data are stewarded, made discoverable and accessible continually evolve with technological innovation and public access policy mandates. Medical librarians, sometimes in collaboration with other information technology partners, have become the go-to-source for information about the best practices for data collection, storage, and preservation, the data life-cycle. We create instructional materials in multiple formats to reach our different audiences, including our colleague who may field questions about data-related issues. There are many forums for information professionals to discuss these issues and share solutions and in this session, we will create such a forum for focused discussion around the ways medical librarians are involved in data management. We will use the MLA publication, “Guide to Data Management for Librarians” edited by Lisa Federer as our starting point to facilitate discussion about the following themes: models of data management instruction; data consultation versus data manipulation services; discoverability challenges; and the open data movement. We encourage all attendees to participate in this discussion and hope this special content sessions serves as a catalyst for the creation of a formal medical data librarians community.

Keywords:

Data

Data Management

Instruction

Preservation

Open Data

Sunday, May 28, 2017, 3:00 PM – 4:25 PM
Room: 608

Session: Call to Action for Diversity and Inclusion: Perspectives for Our Patrons and Out Profession

Call to Action for Diversity and Inclusion: Perspectives for Our Patrons and Our Profession

Megan Threats

Doctoral Student in Information Science, University of North Carolina, Chapel Hill, Chapel Hill, North Carolina

James Anderson

Physician Assistant, Evergreen Treatment Services, Seattle, Washington

Annabelle V. Nunez

Mel & Enid Zuckerman College of Public Health Liaison Librarian, University of Arizona, Tucson, Arizona

Gerald (Jerry) Perry, AHIP

Associate Dean for Health Sciences and Strategic Planning, and Director, University of Arizona Health Sciences Library, University of Arizona Libraries, Tucson, Arizona

Hannah F. Norton, AHIP

Interim Fackler Director, Associate University Librarian, University of Florida, Gainesville, Florida

Objectives: We are in a time of movement making, where matters of diversity and inclusion are brought to the forefront of our conversations. Our profession is broadly impacted by current events as we seek to move toward a more equitable society for all. This session covers a wide range of topics related to the unique cultural, gender, and socioeconomic perspectives of the people in our profession and those that we serve. Areas include promoting inclusivity in outreach by identifying unconscious bias, building a diverse profession, providing access to health information for specific populations, being inclusive in our service models, and recognizing developments in biomedical research related to sex and gender specific analysis and health outcomes.

Members from within our profession and others serve as panelists, and they will give short presentations on their areas of interest. Though the presentation topics are diverse, panelists will reflect upon the role each of us play in striving for equality and justice in our world. Following the presentations there will be an interactive discussion with attendees to further discuss issues of diversity and inclusion. The session will

conclude with a final perspective drawing together the areas discussed and giving ideas for providing leadership, supporting services, and building relationships that are respectful and thoughtful of our diverse world.

Keywords: diversity, inclusion, unconscious bias, implicit bias, equality

Sunday, May 28, 2017, 3:00 PM – 4:25 PM

Room: 612

Session: Lightning Talks 1

Moderator: Jonquil D. Feldman, AHIP

Evaluating and Choosing a Point-of-Care Tool for Your Library

Billie Anne Gebb

Director of Library Services, Frontier Nursing University, Lexington, Kentucky

Objectives: To perform a systematic evaluation of point of care / clinical decision tools to determine which products should be included in the library collection to meet the needs of both clinical and didactic students as well as faculty.

Methods: A committee including clinical, didactic, and librarian faculty was formed to evaluate leading point of care tools for inclusion in a university library collection. Committee members evaluated each product for content, currency, evidence level, and usability. To evaluate content, all members reviewed the same topics and gave feedback on their findings. Additionally, each member reviewed a topic area of her choice. Currency was evaluated by marking the references as generally being more or less than five years old. Evidence level and usability were evaluated using five point Likert scales. Other faculty were offered the opportunity to evaluate the products by identifying most and least favorite features, and giving an overall rating of the product on a scale of one to five. Results of these evaluations were analyzed to make a recommendation regarding point of care tools for the library collection.

Results: The product evaluations showed that UpToDate scored highly for overall usability but that references in DynaMed Plus were more current. There was no significant difference in evidence level in any of the products reviewed. Content reviews showed concerns regarding evidence selection, older references, and reliance on a medical model of treatment, as well as further usability issues. No particular product outperformed the others.

Conclusion: Because the evaluation revealed that the products were somewhat similar and all had concerns, the committee felt it was important to provide more than one point of care tool for the library collection. The committee's recommendation will be to add UpToDate to the collection while maintaining current subscriptions to DynaMed Plus and Essential Evidence Plus. The proposal is pending budgetary approval.

Keywords: clinical decision making

point of care tool

mobile apps

collection development

electronic resources

Discovering Data: A Federated Search Portal of Data Journals

Melissa A. Ratajeski

Coordinator of Data Management Services, Health Sciences Library System, Pittsburgh, Pennsylvania

Andrea Ketchum

Research and Instruction Librarian/Scholarly Communication Liaison, Health Sciences Library System, Pittsburgh, Pennsylvania

Angela Zack

Knowledge Integration Lead, Health Sciences Library System, Pittsburgh, Pennsylvania

Carrie L. Iwema

Information Specialist in Molecular Biology, Health Sciences Library System, Pittsburgh, Pennsylvania

Objectives: Data journals are a means to share datasets and provide detailed information about the methods and instrumentation used to acquire the data. MEDLINE indexes very few data journals and does not provide full-text searching. To facilitate data discovery we created a federated search portal that searches the full-text of select open access data journals.

Methods: We identified seven open access health science data journals and examined their search features. To be included in the federated search portal each journal's search interface had to allow searching across all fields, such as data type, data description, materials, methods, instrumentation, data source location, and data accessibility. Four journals met this criteria: Data in Brief, Genomics Data, GigaScience, and Scientific Data. Using the proprietary software IBM Watson Explorer a federated search portal was developed to search across the data journals with one query. Search results are aggregated and ordered by relevance and can be filtered by journal name or clustered topical categories that are created on the fly based on the textual information of the retrieved records. The portal is available on our data management LibGuide. Usage is tracked and feedback/comments can be submitted to guide further changes.

Keywords: data management; data journals; federated searching

Patient Education Materials: Assessing Compliance with Health Literacy Standards

Erica R. Brody

MSLS Student, School of Information & Library Science, Durham, North Carolina

Objectives: Education materials help patients understand and comply with self-care recommendations, thus avoiding problems that delay recovery or require additional healthcare intervention. This project evaluated the quality of a sample of patient education materials used at a large academic medical center.

Methods: In collaboration with leadership of the patient education committee of a large academic medical center, a library science student obtained educational materials distributed to patients about self-care activities for burns, tracheostomy, and peripherally-inserted central catheters. Data about each item was

abstracted into Libre Office, including items from the Patient Education Materials Assessment Tool (PEMAT). PEMAT understandability and actionability scores were calculated using Microsoft Excel to analyze variations in the quality of patient education materials overall and by the following characteristics: (1) source (health system staff – group process, a national health education vendor, and health system staff – individual post-visit summary), (2) health condition, and (3) care setting where deployed (inpatient, outpatient, home health).

Results: Thirteen patient education materials from inpatient, outpatient, and home health settings were assessed. PEMAT scores for understandability and actionability were associated with the presence of visual aids and document source, variables that were also correlated with each other. Materials created by system staff using a group process and informed by assessment of patient education needs had higher PEMAT scores than those from an external vendor or generated by individual clinicians. Materials created by system staff were also more likely to contain visual aids. The two highest scoring materials were also the longest in length, each more than 10 pages.

Conclusion: Additional research is necessary to tease out the effect of visual aids and source on the quality of patient education materials. The PEMAT is a useful tool for assessing patient education materials. However, the PEMAT does not account for the length of patient education materials and users may not stay engaged with longer documents to fully benefit from the information presented. As health care providers have recently adopted patient education from novel sources to meet requirements of meaningful use, it is important to ensure that these materials effectively inform patients about self-care activities for improvement and maintenance of health. In addition, this project demonstrates how a library student can conduct a pilot project with clinical staff to test out a process for evaluating patient education materials.

Keywords: patient education, health literacy, content analysis, self-care

Keeping up with Demand: Doing National Institutes of Health Biosketch Support

Karen Gutzman

Impact and Evaluation Librarian, Galter Health Sciences Library, Chicago, Illinois

Pamela L. Shaw

Biosciences & Bioinformatics Librarian, Galter Health Sciences Library, Chicago, Illinois

Kristi L. Holmes

Library Director and Associate Professor of Preventive Medicine, Health and Biomedical Informatics Division, Galter Health Sciences Library, Chicago, Illinois

Objectives: The NIH biographical sketch allows scientists to elaborate on their contributions to science and the influence of their research in science. While this is a helpful step towards evidence-based decision making in research funding, it may be difficult for researchers in all stages of their careers to determine how best to describe or quantify their scholarly contribution and research impact.

Methods: This presentation highlights our efforts to assist investigators with the NIH biosketch requirements and to help them communicate the meaningful impact of their work. We briefly review our training materials, frequently asked questions, and practical tips for keeping up with the demand. We also discuss the combination of promotion and outreach that quickly made our biosketch services the most highly sought after training in our library's recent past. To date, we've reviewed and provided comments on over 50 biosketches. Finally, we offer suggestions to those interested in offering this important and highly popular service.

Results: To date, we’ve reviewed and provided comments on over 100 biosketches. We have provided 37 trainings to program managers, researchers, post and pre-doctoral students.

Conclusion: Providing NIH biosketch support has been a highly active area of service for our library. We hope to continue providing this service as our university continues to seek increased funding from the NIH and other funding bodies that require biosketch-like materials.

Keywords: NIH biosketch, research support, research impact, promotion and outreach, writing support

The Library's Small "L" Leadership in Meeting the Mission of an Academic Health System

Elaine Attridge

Clinical Librarian, The Claude Moore Health Sciences Library, Charlottesville, Virginia

Gretchen Arnold

Director, The Claude Moore Health Sciences Library, Charlottesville, Virginia

Daniel Wilson

Associate Director for Collections & Library Services, The Claude Moore Health Sciences Library, Charlottesville, Virginia

Tracey Hoke

Chief of Quality and Performance Improvement, Health System Administration, Charlottesville, Virginia

Objectives: To determine the usability of repurposed space to support the mission of an academic health sciences center.

Methods: The Affordable Care Act has created incentives to change clinical practice in order to foster better coordination and quality care. The University of Virginia Health Sciences Center has developed new processes for merging these goals with the educational mission including daily "huddles" to exchange information and problem-solve. Through the Library's participation in these high level working groups comprised of administration and clinicians, the need for a collaborative space was identified. In this PDCA project, the library rapidly repurposed a central, accessible space with a flexible design allowing for quick information exchange and informal break-out areas. Through prominent branding, this space promotes the hospital's quality initiatives in a non-verbal way further contributing to the institution's mission. A quantitative research questionnaire will be utilized to measure the effectiveness of this space for enhancing communication among participants.

Results: An anonymous, print survey was made available for one week to approximately 70 participants who attend the daily leadership huddle. IRB was consulted and approval not required. 33 surveys were returned and 21 included suggestions for improvement. The room size, visual displays on whiteboards, real-time dashboard data on digital displays, and the location of the room in the library were elements that positively affected communication for this group. 18% found the presence of a librarian at the daily huddle to be of value to their service line. Commenters stated the space is appreciated, provides natural breakout areas for networking, and needs more seating. A quantitative survey cannot measure the positive response generated towards the library from hospital administration and high-level clinicians for proactively responding to a need.

Conclusion: Re-purposing library space to meet the needs of the institution creates successful connections and positions the library as an active member of the health system team.

Keywords: effective space collaboration, institutional mission, leadership, responsive

Coming out [of the Stacks]: Reducing Informational Barriers to Care through Community Engagement at a Local Pride Festival

Jessica Petrey

Clinical Librarian, Kornhauser Health Sciences Library, Louisville, Kentucky

Objectives: To distribute lesbian, gay, bisexual, and transgender (LGBT) health information, increase library visibility as an ally, and promote resources we have selected and created, our library engaged the local LGBT community through outreach at a local Pride festival. This presentation details the planning of the booth and reflects on the results, successes, and learning experiences of the event.

Methods: Before the festival, we began evaluating and developing our collections on LGBT health and created an electronic LGBT consumer health resource guide through LibGuides. We also sought out free resources from professional organizations, such as the Fenway Institute and Gay and Lesbian Medical Association, to distribute. At the booth, we displayed our collections, promoted our LGBT consumer health LibGuide, spoke with community members about our offerings and goals, and increased visibility of our library though branded stress balls we gave away. We also handed out brochures promoting OutCare, a network connecting patients with LGBT friendly and competent healthcare providers, as well as guides on how to talk to doctors and how to find reliable LGBT health information online.

Results: Library faculty and staff reached a total of 474 members of the community, helped add one provider to OutCare's OutList, completed two literature search requests and recieved one private office visit to learn more about database searching and finding information online as a direct result of engagement at Pride. Our LGBT Health libguide saw a spike of 14 visits on the day of, and 18 visits that week.

Conclusion:

Keywords: LGBT, health disparities, barriers to care, community engagement, outreach

Using Team-Based Learning for Faculty Development on Copyright and Fair Use

Kitti Canepi

Director of Library Services, University Library, Henderson, Nevada

Objectives: Evaluate feedback from a one-hour faculty development session on Copyright and Fair Use for medical educators that used a shortened version of the Team-Based Learning protocols to determine whether it was an effective method for teaching basic Fair Use concepts.

Methods: Identify mean score and standard deviation on six pre-set evaluation questions and explore comments about lessons learned and strategy used to determine whether learning objectives were met and teaching method was effective. The faculty development session utilized a shortened version of the Team-Based Learning protocol that included assigned pre-class reading, a short Group Readiness Assurance Test (gRAT) to assess reading comprehension, a brief review of the reading material, and an activity in which the attendees worked in teams to apply key concepts related to Fair Use and Copyright to three real-world cases based on questions previously asked by other institutional faculty.

Results: Results will be made available during the presentation.

Conclusion: Conclusion will be made available during the presentation.

Keywords: Faculty Development, Team-Based Learning, Copyright, Fair-Use, Instruction

Working with Medical Education to Establish the Baseline

Janet L. Hobbs, AHIP

Manager, Medical Library, Medical Library, Los Angeles, California

Objectives: A measure of academic productivity of any institution is the annual output of peer reviewed publications. This abstract describes an author-level, unit- of-measure method used to identify and quantify scholarly productivity of the physician faculty and housestaff within an organization characterized as having a pluralistic medical model. This project will be useful to other medical librarians involved in similar projects.

Methods: The Medical Library retrieved the 2015 publication data from subscribed databases, including Cumulative Index to Nursing and Allied Health (CINAHL), PubMed, PsyInfo and Web of Science. These reports are predicated on institutional affiliation which presents opportunities for gaps and are insufficient to fully measure output. Most journal articles have multiple authors it was unclear which authors or departmental affiliation were aligned with the institution. The Medical Library correlated the internal 2015 list of faculty and housestaff to the list of Medical Library generated reports that detailed institutionally affiliated author publications. Individual names were cross checked with the external reporting of published author output. Departmental affiliations were matched and entered into an Excel table that aligned author first name, middle initial, last name, department, division, degree and professional title. publication title, publication year, volume and page numbers and the article title.

Results: The results of this study provided the ability to identify and measure the total number of published material, as well as the ability to review Medical Departmental and Divisional output. This data demonstrated that for FY2015, the Department of Internal Medicine had the highest percentage of faculty publication output with the Cardiology ranking the highest in the divisional structure. Analysis of housestaff data determined that almost half of the publishing output for FY2015 was generated by the Internal Medicine Department with General Internal Medicine ranking highest in the divisional structure.

Conclusion: This research demonstrated that by using a standardized methodology and units of measure, publication output from the faculty and the housestaff by Departmental and Divisional affiliation could be interpreted from a Medical Education Institutional view. This project demonstrates a more efficient evaluation method to document publication output and provides the opportunity to measure and compare scholarly output against other internal departments and other Academic Medical Institutions.

Keywords: Peer reviewed publication Scholarly Output Publication Analysis

Evaluating the Impact of Clinical Librarians on Inpatient Rounds

Riley Brian

Medical Student, Pritzker School of Medicine, Chicago, Illinois

Nicola Orlov

Assistant Professor of Pediatrics, Department of Pediatrics, Chicago, Illinois

Debra Werner

Librarian for Science Instruction & Outreach and Biomedical Reference, John Crerar Library, Chicago, Illinois

Shannon Martin

Assistant Professor of Medicine, Department of Medicine, Chicago, Illinois

Vineet Arora

Associate Professor of Medicine, Department of Medicine, Chicago, Illinois

Maria Alkureishi

Assistant Professor of Pediatrics, Department of Medicine, Chicago, Illinois

Objectives: Clinical librarians (CLs) are involved in medical education in various ways. Some CLs attend inpatient rounds where they perform literature searches and assist in answering clinical questions that arise. This study aimed to determine the effect of a CL on inpatient teams' clinical questioning and learner skills in asking evidence-based medicine (EBM) questions. This has not previously been objectively evaluated.

Methods: Clinical questioning was measured over 50 days of inpatient rounds by direct observation in which the CL was present for 25 days and absent for 25 days. Observations were evenly split between internal medicine and pediatrics. Question quality was assessed by a blinded evaluator using a rubric adapted from the validated Fresno Test for Evidence-Based Medicine. Surveys were distributed at the end of participants' rotations to assess learners' self-reported change in comfort asking EBM questions and performing literature searches. Descriptive statistics were generated for all data. A Wilcoxon rank-sum test was used to compare the number of questions asked and answered and the quality of questions for when the CL was and was not present. A Wilcoxon signed-rank paired test was used to determine whether survey respondents' ability, comfort, and confidence changed before and after their time on the team.

Results: The presence of the CL on rounds was associated with a significant increase in the number of questions asked ($p < 0.01$) and answered ($p < 0.01$) and in the time spent asking ($p < 0.01$) and answering questions ($p=0.02$). Questions asked in the presence of the CL were significantly higher in overall quality ($p < 0.01$). Eighty-eight percent of study participants completed post-rotation surveys. Participants were significantly more likely to report increased ability to ask, search for, and answer questions at the end of their rotations ($p < 0.01$). In free response answers, some participants reported examples of how the CL's presence had changed patient care.

Conclusion: The presence of a CL on inpatient rounds was objectively associated with more and improved clinical questioning and was subjectively perceived as improving participants' evidence-based medicine (EBM) skills. CLs may also directly affect patient care in some cases, although further work is required to assess this outcome. In conclusion, CLs on inpatient rounds may be an effective means for medical students and physicians to learn EBM skills.

Keywords: Evidence-Based Medicine; Rounds; Medical Education

Librarians as Systematic Review Coauthors: Correlation with Citation Impact Scores

Susan A. Fowler

Library Director, Washington University in St. Louis, St. Louis, Missouri

Angela C. Hardi

Clinical Resources Librarian, Bernard Becker Medical Library, St. Louis, Missouri

Amy M. Suiter

Scholarly Publishing Librarian, Bernard Becker Medical Library, Saint Louis, Missouri

Objectives: Guidelines recommend librarians design searches for systematic review research because of their advanced information skills. However, many published systematic reviews do not include librarian co-authors. Previous research revealed systematic reviews with librarian designed searches and librarian co-authors correlated with stronger methodology; our objective is to see if those systematic reviews with librarian co-authors also have higher citation impact scores.

Methods: We hypothesize published systematic reviews with librarian designed searches, and thus librarian co-authors, will have higher citation scores than those that do not include librarian co-authors because of their stronger methodology. A set of 630 systematic review citations, identified in a previous study comparing the methodology of systematic reviews with and without librarian co-authors, will be analyzed to determine if citation impact scores are higher for systematic reviews that include librarian authors. Relative citation ratio from iCites and field weighted citation impact scores from Scopus will be collected for all citations. We will compare citation metrics between systematic reviews that included librarians as co-authors and those that did not and report on the possible correlation between the inclusion of librarian authorship and higher citation scores.

Results:

Conclusion:

Keywords: Systematic Reviews

Impact Factors

Relative Citation Ratio

Field Weighted Citation Impact

Librarian Authors

Research

Working Together to Advance Scientific Inquiry: Successful Partnerships Between a University's Libraries and Translational Research Center

Karen Gau

Research and Education Librarian, Tompkins-McCaw Library for the Health Sciences, Richmond, Virginia

Objective: Provide an overview on the development of a collaborative relationship between Virginia Commonwealth University’s libraries and translational research center to more deeply engage the university community in opportunities for scientific inquiry and discovery through co-sponsored activities and events.

Methods: VCU Libraries' core values include advancing health information research and encouraging collaboration with and between the communities served. We have closely partnered with our Wright Center for Clinical and Translational Research to launch new programs and coordinate events to advance these values. Over the last few years, our joint accomplishments include launching an annual workshop of hands-on bioinformatics sessions, co-taught by librarians and bioinformatics specialists; initiating a fall speaker series, where selected postdoctoral researchers are given an opportunity to teach health sciences topics to multidisciplinary audiences; coordinating an experimental three-way speed networking event for clinicians, scientists, and engineers; and inviting speakers to educate the academic community on various research and education topics. Attendee evaluations of these programs and events will be discussed to assess the outcome of the partnership and explore further areas for collaboration.

Results: Based on an overall assessment of survey results, the co-sponsored programs and events appear to be fairly well received and successful in achieving their objectives. This partnership allows both organizations to share the workload, increase their marketing reach, and utilize different skillsets and knowledge for the common purpose of furthering health sciences research.

Conclusion: This partnership contributes to the goals of both organizations and, subsequently, to the university as a whole. The partner organizations plan to continue this collaboration and explore new ways to educate and support the academic health sciences community together.

Keywords: collaboration, partnerships, translational research

Finding It Outside the Library: An Analysis of Full Text Availability in Three Disciplines

Jason Burton

Health Sciences Librarian, Indiana University Bloomington Libraries, Bloomington, Indiana

Objectives: This papers examines the question; to what extent are resources cited in faculty publications available in full text outside of library holdings and subscriptions?

Methods: Citations were collected from journal articles, books (excluding textbooks), and book chapters for the years 2011-2016 from faculty in Genome, Cell & Developmental Biology, Optometry, and Speech & Hearing Sciences. An initial search for the cited works was done using library resources to establish a baseline of full text availability. Searches were then conducted on an off-campus network to determine the full text availability of the cited materials using Google, Twitter, and Sci-Hub without University network authentication. An analysis was then performed comparing library and non-library full text availability by overall availability across disciplines, availability by citing discipline, and citation age.

Results & Conclusion: Final results and conclusion will be presented during the talk

Keywords: citation, Sci-Hub, searching, collections

Peer-to-Peer Networking: Merging Two Libraries to Form One User Experience

Aileen McCrillis

Assistant Director, User Services, NYU Health Sciences Library, New York, New York

Irina Meyman

Director, Health Sciences Library, Brooklyn, New York

Erin C.H. Latchford

Department Administrator, Health Sciences Library, New York, New York

Stephen A. Maher

Assistant Director, Content Management and Scholarly Communication, Health Sciences Library, New York, New York

Jeff D. Williams

Deputy Director, NYU Health Sciences Library, New York, New York

Objectives: In the United States, hospital mergers are increasing rapidly in order to improve efficiency, access to care, and quality of care. This trend can have a major impact on medical libraries as their parent institutions consolidate. This case study discusses the process of merging two health sciences libraries and explores the challenges and opportunities presented in this process.

Methods: In 2016, a mid-size community hospital merged with a large academic medical center. Each institution had separate libraries that were to be integrated; including, staff, collections, services, and technology with the goal of establishing an expanded library system with consistent user experience across multiple locations. The authors share their experiences from the perspectives of both institutions and discuss the process used to merge library collections, services, and systems as well as integrating staff reporting lines, cultures, and processes. Many challenges were faced including a long period of uncertainty, geographical distance, and changing of staff roles; however, the integration also allowed for greater collaboration, cooperation, and resource sharing between institutions. The authors will also discuss the impact of the library integration on the user communities they serve.

Results:

Conclusion:

Keywords: hospitals, mergers, access services, user services, leadership, collaboration, culture, integration

Everyone Can Volunteer Here: Collaboration with a Public Library to Provide a Safe Volunteering Program for Survivors of Neurological Trauma

Rachel K. Stark, AHIP

Health Sciences Librarian, California State University, Sacramento, Sacramento, California

Preeti Oza

Assistant Professor, Physical Therapy, Stockton, California

Objectives: Collaboration between the public library, a hospital librarian, and a licensed physical therapist resulted in a volunteer program for survivors of neurological trauma. All participants were assessed for fall risk to ensure safety while volunteering, and staff were advised on appropriate tasks and given basic information about the special needs of survivors of neurological trauma.

Methods: Participants were recruited from stroke support groups, Easter Seals, and a community college’s neurological trauma support group. Three cohorts of no more than 6 participants attended an orientation where they were assessed for fall risk, completed a series of tests, and given a basic overview

of the public library and examples of tasks they would be doing as volunteers. Volunteers for this program were integrated into the general volunteer population and encouraged to volunteer for as many hours as they felt they could do. Every other month data was collected about the physical and neurological standing of the participants. At the end of the pilot project, data was collected regarding staff perceptions of the program and participant perceptions of the program.

Results: There were no injuries reported by participants during volunteer times at the public library. Participants reported increased capabilities, such as concentration, due to their volunteer work at the public library. Staff reported that the volunteers from this special program did not create more work than other volunteers and that they were willing to continue with the project beyond the pilot

Conclusion: This program indicates that there is a benefit to collaboration between public libraries and hospital libraries to create programs for community members who have survived neurological trauma. Hospital libraries often lack the space and man power for this kind of volunteer program, and public libraries often lack the knowledge to find participants and clinical partners. Through collaboration, it is possible to create a volunteer program for members of the community who otherwise don't' have an opportunity to volunteer. It is also possible to create a safe and effective program that doesn't result in more work for the public library staff.

Keywords: Volunteering

Collaboration

Coordination

TBI

Stroke

Hospital

Library

Program

Improving Library Class Awareness, Registration, and Attendance

John D Jones, Jr.

Instruction & Curriculum Librarian, University of Colorado Anschutz Medical Campus, Aurora, Colorado

Emily Petersen

2017 MLIS Candidate & Intern, Health Sciences Library, Aurora, Colorado

Objectives: Registration and attendance at the library classes on PubMed and other databases was declining. Preparation for a class is a fairly high investment of librarian's time. We reviewed our advertising and registration methods to try to figure out why we would have such low registrations and then only have about 50% of those registered show up. Could we improve?

Methods: We looked at our approach to classes offered and when scheduled. We reviewed communication channels used to notify the campus about the library classes - blog, newsletter, and website. We evaluated the pros and cons of our registration system. We wanted to move fairly quickly and stay agile so we essentially took a modified rapid prototyping approach to try changes, see what worked, what didn't and make more changes. We also realized that changing too many things simultaneously would make it harder to know which was having the biggest impact. Coincidence or perhaps rather convergence can also play an important role in understanding how patrons may or may not be using your library. Could we improve awareness? Could we improve registration? Could we improve attendance? Using a combination of emailing and calendaring we seem to be doing just that.

Results: The library advertised free classes via a campus listserv reaching over 10,000 faculty and staff. Upon review, we identified a 2 year gap using the listserv. We had not sent the monthly classes since July 2014. Anecdotally, we believed that many people registered so far ahead they didn't remember their registration. Participants didn't put the class on their calendars. Sending a 2 day reminder was inadequate. Many participants cancelled after the reminder. We created a shared Outlook calendar to invite registered participants so the class will appear on their calendars and they can easily decline the event to cancel their registration. We have seen a 50% increase in registration and attendance.

Conclusion: We'd dropped the ball communicating our class offerings and it was detrimental to our numbers as well as morale. Participants still don't always attend and don't tell us they're not coming, on a health sciences campus we understand. Participants like the Outlook appointments and they are not considered intrusive. We're happy with class change sizes from 2 to 4. We are seeing much bigger changes than that which have really boosted morale.

Keywords: classes registration advertising attendance improvement communication patron emailing calendaring

Sunday, May 28, 2017, 3:00 PM – 4:25 PM

Room: 611

Session: Daring to Grow: Building a Strong Workforce

Moderator: Molly Knapp, AHIP

Standards for Literature Searching: Research and Development

Brooke Ballantyne Scott

Librarian, Royal Columbian Hospital, New Westminster, British Columbia, Canada

Susan Baer

Director of Libraries and Archives, Health Sciences Library, Regina, Saskatchewan, Canada

Marcus Vaska

Librarian, Knowledge Resource Service, Alberta Health Services, Calgary, Alberta, Canada

Lori Leger

Regional Manager of Library Services, Horizon Library Services, Moncton, New Brunswick, Canada

Jackie Macdonald

Privacy Officer, Quality, System Performance and Legal Services, Bridgewater, Nova Scotia, Canada

Ashley Farrell, AHIP

Medical Librarian, Cancer Care Ontario, Toronto, Ontario, Canada

Danielle Rabb

Information Specialist, Information Services, Ottawa, Ontario, Canada

Pat Lee

Retired, Retired, Halifax, Nova Scotia, Canada

Objectives: Library services managers, professional searchers and search instructors lack a standard to support mediated search service instruction and accountable search service delivery. Through a literature review, a Delphi study and analysis, the first part of this work established a framework for five levels of searches against which search service performance may be measured. This paper validates the framework.

Methods: Three approaches were used to inform the work: a) a jurisdictional review of up to 10 leading organizations perceived as having higher than average search rigour to investigate their search methods and their approach to training new mediated searchers. Content gathered will be compared against and used to revise our framework. b) A group of up to 100 professional mediated searchers will execute one assigned search at each of our five levels, then provide search results, documentation on search methods and a post-search analysis of their approach compared with our framework. c) Search results and methods will be compared, evaluated and analyzed, with information used to validate the framework or indicate further revision is required.

Results: In anticipation of a validated framework, a mediated search standard will be drafted to support mediated search services with terminology, approach and methods.

Conclusion: Mediated searching is a core service in both physical and virtual health library settings, where other professional colleagues are certified or licensed and supported with service standards against which performance can be measured. Standards support consistent service experiences and enable performance measurement. This work will more closely align health librarians with other health professionals and help maintain the relevance and value of health services libraries within the health care system.

Keywords: Literature searching, mediated searching, professional searching, standardization of services, library management, library education, accreditation, environmental scan, jurisdictional review, delphi study

Accepting the Leadership Challenge: What Academic Health Sciences Library Directors Do to Become Effective Leaders

Rick L. Fought, AHIP

Director, University of Tennessee Health Science Center, Memphis, Tennessee

Mitsunori Misawa

Assistant Professor, Adult Learning and Adult Education, Department of Educational Psychology and Counseling, Knoxville, Tennessee

Objectives: Libraries face challenges as they continue to adapt to technology and changes in how information is created, organized, used, and preserved. They are under increased pressure to assess

their work and be accountable for returns on investment to their institutions. A better understanding of what makes academic health sciences library directors effective could provide valuable guidance to future library leaders.

Methods: Phenomenological research was used to investigate the research questions of the study. Phenomenology focuses on revealing meaning and understanding the essence of a shared experience, and works well with phenomena that does not lend itself to easy quantification, such as leadership effectiveness. The data for this study were collected through semi-structured interviews with eight directors, drawn from academic health sciences libraries at public research universities with a very high research activity classification. The list of participants was further limited by geographical region (Southeast and South Central United States) and membership in the Association of Academic Health Sciences Libraries. Thematic analysis was used to analyze the data. Interview responses were coded, then examined for patterns that could developed into themes.

Results: The major themes were: 1) Assessing Your Environment; 2) Strategies and Decisions; and 3) Critical Skills. These themes captured important aspects in what made the participants' effective library leaders and present practical guidance for improving leadership effectiveness. Assessing Your Environment discusses the need for directors to understand their context and institution to be successful. Strategies and Decisions looks at both long-term and short-term thinking that directors must engage in when leading. Critical Skills exams the skills participants thought were necessary to be effective as library leaders. Overall, the participants were able to convey much practical advice for improving leadership effectiveness.

Conclusion: Academic health sciences libraries are under greater pressure to assess their work and measure outcomes, as well as meet the challenges in an environment where technology and information are always changing in how it is used and created. This study explored the experiences of several experienced library directors to gain a better understanding of leadership effectiveness and provide guidance to future library leaders on how to be more effective leaders. There are many practical methods and skills they were able to share that can be used to improve leadership effectiveness and help all leaders successfully accept the leadership challenge.

Keywords: leadership, effectiveness, management, administration, career development, library directors

Connecting the Dots: Informing Online Graduate Library Curriculum Development for Health Sciences Librarianship through Employer Surveys

Jinxuan Ma

Assistant Professor, Emporia State University, Emporia, Kansas

Objectives: This is a two-phase study examining a developed cross-disciplinary model of online library curriculum development for health sciences librarianship (HSL). The model is built upon the evolving HSL core competencies interlinking to new demands and challenges imposed upon librarians and their emerging roles. These competencies overlap with the MLA's code of ethics, MLA's seven competencies, and SA's six core competencies with additional enabling competencies.

Methods: A systematic review is conducted to identify emerging HSL roles and the associated professional settings. The alignment of recognized competencies and collected roles of HSL provides an operational examination and research-based evidence for both developing and updating curricular learning outcomes, course content and delivery approaches, and student career pathways for existing online graduate HSL specialization courses in library programs. This study aims to further identify the required knowledge and skills for an entry-level health science librarian by surveying a purposive sample of employers to specify what knowledge base, subject knowledge, and specialized expertise a new librarian is expected to attain through graduate library study.

Results: The survey findings will be mapped to the cross-disciplinary model to complement and enrich the specified core competencies of HSL across the recognized new streams of professional settings and their innovative practices.

Conclusion: (will be available during the presentation.)

Keywords: Health Sciences Librarianship (HSL); online graduate library curriculum; code of ethics; professional core competency; enabling competency; text mining approach; research-based evidence

With a Little Help from My Friends: A Peer-Led Professional Development Program for Hospital Librarians

Rebecca L. Bayrer, AHIP

Library Services Manager, Kaiser Permanente, San Francisco and South San Francisco, South San Francisco, California

Dawn M. Melberg

Manager, Library Services, kpLibraries, Santa Rosa, Santa Rosa, California

Rachel K. Stark, AHIP

Health Sciences Librarian, California State University, Sacramento, Sacramento, California

Chandrika Kanungo

Manager, Library Services, kpLibraries, San Jose and Redwood City, San Jose, California

Carrie Walsh

Assistant Librarian, kpLibraries, San Rafael, San Rafael, California

OBJECTIVE: To describe a program of professional development for librarians in a large, geographically dispersed hospital system. Librarians at Kaiser Permanente typically function as solo librarians, leaving them little time or opportunity for continuing education and other professional growth activities. Our program, which may be easily adopted in other settings, has succeeded in offering popular, effective, and inexpensive professional development opportunities.

METHODS: During the past five years, our professional development committee has established a program to support the continuing development of librarians at our institution. The committee hosts a series of monthly in-house, web-based seminars; curates a list of relevant resources; and maintains a calendar of free webinars. Another popular feature is a monthly “TechTime,” an open discussion format where librarians exchange technology tips to increase their abilities and efficiency. Occasional full-day, hands-on sessions refine our research skills; and a Knowledge Exchange program matches mentors and mentees who are interested in teaching and learning from each other. Peer feedback guides the committee in planning future activities.

RESULTS: Over the past five years, the kpLibraries professional development committee has offered a wealth of training opportunities encompassing a wide range of medical librarian skills. Organizational leadership is supportive of our inexpensive and creative solutions. Our satisfaction surveys indicate that the committee is providing popular and effective professional development opportunities that are easy for librarians to access and are highly regarded.

CONCLUSION: Implementing a professional development committee is a viable solution for providing continuing education and professional growth opportunities to time-strapped medical librarians. Utilizing in-house resources ensures trainings are inexpensive, relevant, focused, and effective.

Keywords: professional development, continuing development, peer education, committees, learning, training, teaching, hospitals,

Evidence-Based Practice for Everyone: Getting Non-Health Sciences Librarians up to Speed

Molly Montgomery

Health Sciences Librarian, Idaho State University, Meridian, Idaho

Objectives: The objective was to determine if teaching the basics of evidence-based practice to non-health sciences librarians could increase their confidence, comfort, and ability when helping students with health-related research questions.

Methods: The health sciences librarian located at a satellite campus developed a flipped instruction program to help the non-health sciences librarians understand basic principles associated with evidence-based practice. The main campus librarians were asked to watch 3 narrated PowerPoints/screencasts, read brief articles, and then take a quiz to assess their learning. The health sciences librarian then taught a two-hour, face-to-face instruction session with mostly hands-on activities and discussions. Both portions of the instruction focused on developing research questions via PICO, determining the level of evidence needed, and how to search for high-level evidence.

The librarians who participated in the both the flipped and live portions of the instruction were assessed with a knowledge survey one week following the instruction, and again three months following the instruction.

Results: Three months following the training, the post-test indicated positive changes in knowledge and attitudes in issues related to EBP in the non-health sciences librarians. All librarians correctly identified Cochrane as a source for high-level evidence in the post-test versus only 53% in the pre-test. Only half of the librarians identified meta-analyses as the highest level of research in the pre-test, but all answered the question correctly in the post-test. Comfort in working with EBP topics also improved. Only 14% of the respondents indicated that they felt they could help a student build a question using PICOT(T) prior to the training, which improved to 56% following the training. Only 14% of the librarians knew how to locate systematic reviews in the pre-test, but following the training that number increased to 100%.

Conclusion: The librarians who took part in the evidence-based practice for non-health sciences librarians training reported increased confidence in helping students with health sciences questions. The post-training assessments found increased knowledge of basic evidence-based principles, even months after the instruction session.

Keywords: EBP, evidence-based practice, flipped instruction

Sunday, May 28, 2017, 3:00 PM – 4:25 PM

Room: 607

Session: Dreaming Up New Ways to Improve Your Searches

Moderator: Penny Coppernoll-Blach, AHIP

Evaluating the Consistency and Quality of Search Strategies and Methodology in Cochrane Urology Group Systematic Reviews

Jennifer A. Lyon, AHIP

Librarian, Children's Mercy Hospital, Kansas City, Missouri

Carrie Price

Clinical Informationist, Welch Medical Library, Baltimore, Maryland

Jamie Saragossi

Health Sciences Collection Development and Outreach Librarian, Stony Brook University Libraries, Stony Brook, New York

Clara Y. Tran

Science Librarian, Stony Brook University Libraries, Stony Brook, New York

Objectives: The Cochrane Urology Working Group has undergone multiple administrative and personnel changes over its history, including merging with the Cochrane Prostatic Diseases and Urological Cancers Group. Presently, there are nearly a dozen Information Specialists volunteering from several institutions and two countries. To identify areas for quality and consistency improvement, we evaluated the search methodologies in 64 Urology Group Reviews/Protocols.
Methods: All 64 published Urology Group Cochrane Systematic Reviews (CSR) and Protocols (CSP) were identified and downloaded from the Cochrane Library as of September 15, 2016. A data form was created using Qualtrics to collect evaluative assessments of the CSRs and CSPs. This form consisted of several sections, included expanded PRESS (Peer-Review of Electronic Search Strategies) elements, database and resource selection, search methodology reporting, consistency between search strategies, and related information. Four librarians, including two Cochrane Urology Group Assistant Information Specialists, evaluated 20 CSR/Ps each, allowing overlap to verify data extraction reliability. The form was pre-tested by all four librarians on multiple CSR/Ps before use.
Results: Librarian reviewers noted high variability in CSR/P search methods. The range of databases searched was extremely wide with only MEDLINE and a Cochrane database seen consistently. It was rare to see search strategies provided for all (or even most) databases, with higher inclusivity in more recent reviews. Many reviews were limited to term listings in the methods while others provided one strategy as an example. Publication type filtering was extremely variable, with alterations to standard Cochrane filters common. Most reviews included reference list searching and author contacts, but had little consensus regarding trial registries, conference proceedings, and journal table of contents. Within search strategies, common errors included wrong line numbers, misspellings, inappropriate syntax, and duplicated lines.
Conclusion: Full reporting of reproducible and documented search methods for reviews is vital. Cochrane Reviews are frequently viewed as the "gold standard" of systematic reviews, therefore the CSR/Ps should have high quality search methodology. This retrospective analysis represents a historical overview of search method reporting in CSR/Ps from the Cochrane Urology Group. Our results demonstrate a need for improved standardization including complete documentation of all strategies, more consistent database selection, less variation in strategy construction, increased use of peer-review and inclusion of the PRISMA Flow Diagram, and tighter adherence to the Cochrane Handbook/MECIR Standards.
Keywords: systematic reviews, expert searching, searching, databases, Cochrane, evaluation, quality improvement, search methodology, research

Comparing the Effectiveness of Conceptual Search Methods: Is a Fast Approach Sufficient for the Production of (Sound) Systematic Reviews: A Prospective, Double-Blinded, Controlled Study

Wichor M. Bramer

Biomedical Information Specialist, Medical Library, Rotterdam, Zuid-Holland, Netherlands

Melissa L. Rethlefsen, AHIP

Deputy Director / Associate Librarian, Spencer S. Eccles Health Sciences Library, Salt Lake City, Utah

Margaret Sampson, AHIP

Manager of Library Services, Medical Library, Ottawa, Ontario, Canada

Objectives: Recently we developed a method with which systematic review searches are created ten times faster than traditional. In retrospective analysis, reviews created with this method retrieved significantly more relevant references than other librarian-mediated reviews. Now a group of experienced information specialists compares the method prospectively with traditional methods for multiple reviews. Does it reduce search time without missing relevant references?

Methods: After receiving a request for a librarian-mediated search for a systematic review, a participating information specialist clearly documents the topic and sends this to two other information specialists. One of the three searchers uses the new method. All three searchers try to find as many relevant references as possible. All search results are then combined, deduplicated and presented to the researchers to be screened for relevance.

The search time is registered. Number and overlap of terms in the searches and references retrieved by the searches are determined. After the review is finished, an independent information specialist compares the recall (%retrieved) and precision (%relevant) of the three search strategies for ultimately included references.

Is the new method faster than the other, traditional, methods? Did it miss important references the other searches found? Did it find relevant references the other methods missed?

Results:

Conclusion:

Keywords: Review Literature as Topic; Databases, Bibliographic; Vocabulary, Controlled; Sensitivity and Specificity

Quality of Data Downloads from Bibliographic Databases Frequently Used in Systematic Reviews

Rachael Posey

Pharmacy Librarian, Health Sciences Library, Raleigh, North Carolina

Jennifer Walker

Cancer Information Librarian, Health Sciences Library, Chapel Hill, North Carolina

Objectives: To assess the quality of data downloaded from a variety of bibliographic databases commonly searched in systematic reviews, in order to provide guidance on the order in which database records should be uploaded into a citation manager and which record should be used as the “primary record” during the duplicate removal process.

Methods: We downloaded the bibliographic records of a random sampling of two hundred journal articles from the following databases: MEDLINE (via PubMed and Ovid), Embase, Web of Science, Scopus, Cochrane, CINAHL (via Ebsco), International Pharmaceutical Abstracts (via Ebsco), Sociological Abstracts (via Proquest), and Google Scholar. We required that each article be available in at least three databases and that each database contain at least fifty of these articles. We then assessed the completeness and correctness of the downloads using the full-text article as our gold standard. Records were scored on presence, completeness, and correctness of the following fields: title, authors, digital object identifier (DOI), volume, issue, pages, publication year, accession number, abstract, and URL. Using these data, we calculated an overall score for each database and a head-to-head score for each database combination using the article overlap for each database pair.

Results: Results will be made available during our presentation at MLA '17.

Conclusion: Conclusions will be made available during our presentation at MLA '17.

Keywords: systematic reviews, duplicate removal, citation managers, bibliographic databases, data analysis

Where Are We Now?: Current Library Data Support Services

Andrea H. Denton

Research & Data Services Manager, Claude Moore Health Sciences Library, Charlottesville, Virginia

Bart Ragon

Associate Director, Knowledge Integration, Research, and Technology, Health Sciences Library, Charlottesville, Virginia

V P. Nagraj

Research Data Analyst, Claude Moore Health Sciences Library, Charlottesville, Virginia

Objectives: Many biomedical libraries’ initial data-related services focused on providing support to researchers creating data management plans in response to funder requirements. In the 5+ years since the NSF data management plan mandate, how have data services evolved? This paper will discuss how biomedical libraries are currently serving both individual researcher’s and the research community’s data support needs.

Methods: The goal of this study is to describe the provision of data-related services to researchers by exploring current and planned services provided by biomedical academic libraries. An interview approach was utilized to capture data-related services provided by libraries, and to illustrate scenarios that might assist with strategic planning of new services. Services to individual researchers (e.g. one-on-one consultations) as well as the research community were examined. Emphasis was placed on examining library involvement throughout the research lifecycle, so that any variety in the services provided by biomedical libraries could be described. In-depth user stories were collected to further illustrate the impact of libraries through data-related services. Results may provide insight as to whether there are certain services shared by a majority of libraries, or if data support services are dependent on the organizations and population served, or other factors.

Keywords: Data management

Data support services

Biomedical researchers

Replicating a Dr. Google Study: Challenges of Reproducing Online Information-Seeking Studies across Domains

Kristine M. Alpi, AHIP

Director, Wm. R. Kenan, Jr. Library of Veterinary Medicine, North Carolina State Univ. Libraries, Raleigh, North Carolina

George Schaaf

DVM Candidate, College of Veterinary Medicine, Raleigh, North Carolina

Objectives: Reproducibility remains infrequently pursued for health information-seeking studies. Googling for a Diagnosis (BMJ, 2006) using New England Journal of Medicine cases has 350+ citations and inspired similar studies by medical specialties. We describe the challenges faced by a team comprising a specialist, a generalist, a student, and librarian in replicating the study in veterinary medicine while improving its methodological transparency.

Methods: In addition to addressing methodological concerns and limitations, we decided to explore the possible impact of clinician expertise on the searches and the final diagnoses. Starting in October 2014, we reviewed supplemental material for the detailed searches and discussed searching and expertise with several clinicians before deciding to have both a specialist and a generalist generate searches and diagnoses. The librarian and student selecting and blinding Journal of the American Veterinary Medical Association small animal medicine cases were independent from the clinicians developing Google searches and identifying diagnoses. We finished modifying the design in April 2015 and the changes included documenting clinicians' previous exposure to the published cases, experience levels, and their individual search strategies. The student conducted all searches within a tight time window and the results were provided to both clinicians to inform their final diagnoses.

Results: The primary outcome of both studies was the percentage of Google-assisted correct diagnoses. To address the limitations and questions raised in and about the BMJ paper findings, we compiled results on other factors to clarify the potential effect of the clinician on the search and therefore on the Google-assisted diagnostic performance. We calculated pre-Googling diagnostic performance and confidence, documented the final diagnoses, and whether the diagnosis changed after the search process. We characterized search strategies by word count, term count, and inclusion of signalment, and described types of resources that appeared in the results.

Conclusion: Replicating the BMJ Google Diagnosis study was challenging due to the lack of specificity in the original article. Changing the domain increased potential confounders. Thirty small animal internal medicine cases came from five years of JAVMA compared to 26 from a single year of NEJM. Veterinary information online remains scarce compared with human medicine. Finally, the nine year gap between studies raises questions about the impact that the growth of medical information and refinements of Google search algorithms likely have on comparison studies in terms of differences across domains and/or time periods.

Keywords: Reproducibility, health-information seeking, Google searching, case reports, diagnosis, veterinary medicine, expertise

Sunday, May 28, 2017, 3:00 PM – 4:25 PM

Room: 615

Session: Daring to Integrate into the Medical Curriculum

Moderator: Renee Gorrell, AHIP

Finding Biomedical Research Information and Communicating Science: A New, Librarian-Taught, Credit-Bearing Course for Graduate Students

Mary E. Edwards, AHIP

Reference & Liaison Librarian, Health Science Center Library, Gainesville, Florida

Michele R. Tennant, AHIP

Interim Fackler Director, Health Science Center Libraries, Gainesville, Florida

Hannah F. Norton, AHIP

Interim Fackler Director, Associate University Librarian, University of Florida, Gainesville, Florida

Margaret E. Ansell, AHIP

Nursing & Consumer Health Librarian, University of Florida, Gainesville, Florida

Objectives

Both out of a broad desire to more fully integrate information-related skills into the University of Florida's (UF) curricula and specific input from biomedical PhD students who were unable to dedicate time to educational sessions not offered for credit, four librarians developed a one-credit course for masters and PhD students: "Finding Biomedical Research Information and Communicating Science."

Methods

While librarians at the UF HSCL have long been integrated into the curricula of the programs we serve and offered stand-alone workshops on information-related topics, we had not previously taught any credit-bearing courses focused on information literacy. The instructional team designed a new one-credit course to introduce the most important concepts, resources, methods, and tools used in searching for and communicating biomedical information. Topics covered included literature searching, bibliographic citation software, basic NCBI resources, funding sources, data management, and plagiarism and information ethics. The course was approved through College of Medicine and university level curriculum committees, and their feedback was incorporated to ensure that students learned the conceptual basis and practical tools for finding biomedical information. The course was taught for the first time in the Fall 2016 semester.

Results

Anecdotal feedback and course evaluation data from the students indicates that the course content met the needs of PhD students in the basic sciences. After meeting with PhD program coordinators in the College of Public Health and Health Professions (PHHP) in the early spring, it was decided that the content may be tailored to fit their curricular and scheduling needs. In particular, a session focusing on genetic and genomic resources will be replaced with an introduction to systematic reviews for the PHHP audience. In terms of curriculum sequencing, rather than being in a 5 week block during the fall, the

summer course will be offered during one of the abbreviated summer semesters. The instructional team will also offer the course again in fall of 2017 to the original, biomedicine focused audience.

Conclusions

Developing and teaching a credit-bearing graduate course on information-related topics is one mechanism for librarians to reach those students who cannot attend stand-alone workshops. Once a course is developed and approved by a specific campus unit, it may prove relevant to other units and the content can be modified to fit the needs of other programs, thus widening the impact and helping to ensure that graduate students across a range of disciplines are exposed to similar information-related content.

Keywords: instruction, teaching, graduate students, information literacy

Transforming Curriculum, Transforming Roles: New Paths to Curriculum Integration

Whitney Townsend

Coordinator, Health Sciences Executive Research Service, Taubman Health Sciences Library, Ann Arbor, Michigan

Mark P. MacEachern

Informationist, Taubman Health Sciences Library, Ann Arbor, Michigan

Emily Ginier

Informationist, University of Michigan Taubman Health Sciences Library, Ann Arbor, Michigan

Objectives: To describe the process and outcomes of transforming an instructional session on critical appraisal of systematic reviews from a librarian-led large group active learning model to faculty-led small group discussions as a part of the curricular redesign of a large medical school.

Methods: Recent efforts to transform the curricula at many undergraduate medical schools in the US provide areas of unique opportunities and challenges to health sciences librarians involved in curriculum-integrated instruction. This paper will describe how a team of librarians adapted an existing session on critical appraisal of systematic reviews to be incorporated into the newly developed M1 curriculum at a large Midwestern medical school. This paper pays particular attention to the changing roles of the librarians from instructors to curriculum designers for faculty-led small groups as a part of the curriculum redesign process.

Results:

Conclusion:

Keywords: curriculum, undergraduate medical education, medical school, critical appraisal, faculty development, instructional design

Integrating Information Literacy through a Biomedical Informatics Course at National Autonomous University of Mexico Faculty of Medicine in Mexico

Alejandra Martinez del Prado

Medical Librarian, UNAM [National Autonomous University of Mexico], Distrito Federal, Distrito Federal, Mexico

Luis Fernando Kieffer Escobar

Pediatric Surgeon, Biomedical Informatics Department, Mexico, Distrito Federal, Mexico

Sandra G. Moncada Hernández

Head biomedical librarian, Instituto de Fisiología Celular, Mexico, Distrito Federal, Mexico

Xóchitl Marlette Lobato Valverde

Evaluation coordinator, National Autonomous University of Mexico, Mexico city, Distrito Federal, Mexico

Objectives: Undoubtedly information-based decision making is a necessary skill for health professionals. With the emergence and continuous development of Information Technologies the challenge has turned from finding information to finding quality information. This paper examines the integration of information literacy through a biomedical informatics (BMI) course at the National Autonomous University of Mexico (UNAM) Faculty of Medicine in Mexico and outlines the advantages that students have with this course during their professional career.

Methods: The UNAM Faculty of Medicine, one of the largest medical schools in Latin America, developed and implemented a biomedical informatics mandatory course for two years (BMI-1 and BMI-2). Information literacy components were included in BMI-1 according to the Association of American Medical Colleges and the International Medical Informatics Association recommendations on biomedical informatics education. BMI-1 has the unit 'Databases and medical digital libraries' which goal is to know the main biomedical information resources, apply and use the strategies and tools needed for their effective use. Librarians joined the health care team and have teaching roles with physicians since the BMI course started and implemented strategies and didactic tools to achieve this goal. Information searching skills of students have been observed and evaluated while they are in BMI-2 and when they use the Faculty of Medicine library in person and remotely.

Results: They will made available during our presentation at MLA '17.

Conclusion: They will made available during our presentation at MLA '17.

Keywords: Biomedical Informatics / undergraduate education / Information Literacy

A Program Description to Assess Information Literacy Initiatives by Librarians in the Online, Embedded Role

Trey Lemley, AHIP

Information Services Librarian, Biomedical Library, Mobile, Alabama

Objectives: To develop an assessment program for the evaluation of information literacy initiatives by embedded librarians in the curriculum of online, graduate nursing courses. Currently, library faculty and administration have no effective means for assessment; therefore, this project is designed to meet this need by measuring librarian effectiveness in helping students achieve learning outcomes.

Methods: Setting: An academic health sciences library serving a college of medicine, a college of nursing, and a college of allied health. Population: Graduate-level nursing students enrolled in exclusively online degree programs, nursing faculty, and embedded librarians. Research Design: Before the course

begins, based on learning objectives, a curriculum map will be used to develop a schedule for targeted library instruction. The librarian will institute various means of outreach, including “librarian office hours" and web-based tutorials. Potential specific assessment activities include gradable online assignments, categorizing grids, quizzes, and extra-credit assignments. Course assessment will be made twice per module, at the beginning and end, followed by a needs assessment to determine if objectives have been met. To obtain student input, specific techniques will be used, including surveys and end-of-course evaluations, among others.

Results and Conclusions will be made available during my presentation at MLA '17.

Keywords: Online education

Embedded librarianship

Information Literacy

Assessment

Program Description

New Roles for Australian Clinical Librarians in Developing and Delivering an Evidence-Based Practice Program for Undergraduate Clinical Science Students

Mary Simons

Clinical Librarian, Macquarie University Library, Sydney, New South Wales, Australia

Andrew Davidson

Neurosurgeon, Macquarie University, North Ryde, New South Wales, Australia

Objectives: To develop, implement and evaluate the first stage of a spiral evidence-based practice (EBP) curriculum that is embedded into an undergraduate clinical science program. Librarian-created online modules including a virtual game were piloted to evaluate their role in delivering innovative, blended learning activities. The role of a Library-Faculty collaboration in developing an EBP curriculum for undergraduate students was also assessed.

Methods: Non-academic clinical librarians were appointed as Faculty tutors and conveners to work with clinicians in developing, delivering and assessing an EBP curriculum for undergraduate students. Steps of the evidence cycle (ask, acquire, appraise) became modules of a spiral curriculum where learning was successively reinforced and extended during the two-year program. Clinical librarians took active roles in planning, tutoring and assessing student learning. Blended learning activities, linked to programmatic learning outcomes, underpinned the curriculum. Librarians developed ClinWise, an interactive, modular program to facilitate information literacy (IL) skills acquisition within a clinical context. An online game, Mission to Mars, was created to reinforce EBP understanding using a fictional clinical scenario and an evidence pyramid. The purpose of these self-paced learning activities was to stimulate and evaluate students’ curiosity and engagement with the principles and applications of EBP within an embedded curriculum.

Results: Evaluation of learning took place through student self-assessment, forum postings and tutorial feedback. An assessment task required the preparation of an annotated bibliography to assess knowledge of study design, synthesis of evidence and academic writing skills. Students also completed EBP quizzes, searches, and online learning activities. Reflective forum postings on their experience of the Mars game and ClinWise modules indicated an awareness of the importance of EBP knowledge in increasing research skills within clinical and scientific disciplines. Librarian and Faculty evaluation reinforced the librarians’ role in creating meaningful learning experiences that increase students’ understanding of basic EBP concepts.

Conclusion: The utilization of current learning theory and innovative approaches to EBP curriculum development by a Librarian-clinician team promotes engagement and learning among this student cohort. Blended, self-paced learning activities within a spiral curriculum provided most students with challenging and enjoyable experiences. The Library-Faculty collaboration underpinning this program not only promotes student learning of IL and EBP, but demonstrates the vital role for librarians in contributing to Faculty curriculum development, delivery and evaluation.

Keywords: evidence-based practice undergraduate education curriculum development blended learning clinical librarian

Sunday, May 28, 2017, 3:00 PM – 4:25 PM

Room: 603

Session: Evaluating Our Services: Daring to Take a Closer Look

Moderator: Ayaba Logan, AHIP

Daring to Calculate the Cost Impact of the Librarian

Claire Hamasu

Associate Director, Eccles Health Sciences Library / NNLM MidContinental Region, Salt Lake City, Utah

Kari Jones

Health Economist, -, Salt Lake City, Utah

John Bramble

Research Enterprise / Outreach, National Network of Libraries of Medicine, MidContinental Region, Salt Lake City, Utah

Barbara Jones

Library Engagement//Missouri Coordinator, J Otto Lottes Health Sciences Library, Columbia, Missouri

Objectives: Changes in reimbursement mechanisms are pressuring hospital administrators to better understand the economic impact of each department and employee. Previous research reveals a positive financial and quality-of-care impact when clinicians work with health sciences librarians to address their information needs. The NNLM MidContinental Region (NNLM MCR) proposed further investigating the current impact of librarians on costs of patient care.
Methods: A health economist worked with the NNLM MCR to develop a scalable methodology to estimate the patient cost impact of information searches performed by health science librarians. In general, the value of improved information from librarian searches could be estimated from the number of searches regarding patient care that librarians at an institution complete in a given period. For each type of improved patient care outcome or averted adverse event studied (e.g. reduced length of stay, avoided

readmission, etc.), the number of librarian searches could be multiplied by both the percentage of searches that are expected to result in each type of event, and the estimated savings from each type of occurrence. Summing over all types of improved outcomes or averted negative events yields estimated (gross) savings from improved information.

Results: This presentation will explain the need for, details, and scope of the study. Introduction of the project at this stage is meant to foster discussion of the design of and operationalizing various aspects of the methodology and the resources required to complete it.

Conclusion: The authors see that the next steps for implementing the study are:

- Recruit hospital librarians
- Establish the infrastructure for participant support, data collection and analysis
- Find additional funding
- Implement study
- Share results
- Share cost study tools

Keywords: Librarian-value, Cost-impact, Cost-effectiveness, Workforce-efficiency, Research-opportunity

The Role of MEDLINE in Patient Care: Results of a Secondary Analysis of the Value of Libraries Study

Joanne Gard Marshall, AHIP, FMLA

Research Professor, School of Information and Library Science, Chapel Hill, North Carolina

Amber Wells

Doctoral Graduate, Dept of Sociology, Chapel Hill, North Carolina

Kathel Dunn

Associate Fellowship Program Director, National Library of Medicine, Bethesda, Maryland

Joyce E.B. Backus

Associate Director for Library Operations, National Library of Medicine, Bethesda, Maryland

Objectives: What role does the MEDLINE database play in relation to other information resources that are available to health care providers? What is the role of MEDLINE in positively impacting patient care? Since health care providers use multiple information resources in providing patient care, what is the specific role of MEDLINE?

Methods: A previous survey on the use of health information resources for patient care obtained 16,122 responses from 56 hospitals in the U.S. and Canada. The study asked respondents to indicate resources used in answering specific clinical questions. On average, respondents reported using 3.5 resources to answer their question. This analysis used advanced descriptive statistics and regression analysis to examine the specific information resources used and how they were used in combination with one another. The use of more resources was associated with more changes made to patient care and increased avoidance of adverse events. MEDLINE was more likely to be among the resources consulted than any other information resource except journals. The analysis reported in this paper provides new insights into how MEDLINE is used in conjunction with other resources to answer clinical questions.

Results: Our additional analysis of the Value Study data found that MEDLINE and online journals were the two most frequently used information resources. Respondents reported using an average of 3.5

resources when seeking additional information related to a specific patient care decision making situation. Using more information resources was associated with improved clinical decision making and a higher probability of making changes to patient care and avoiding adverse events. MEDLINE was most likely to be among the combinations of information resources used by physicians, residents and nurses.

Conclusion: MEDLINE continues to be a key information resource for health care providers as they seek answers to patient care questions. The MEDLINE database is also used in the preparation of many other specialized health information resources and point of care information tools. Since health professionals use multiple information resources, libraries and librarians continue to have an important role in providing access to and supporting the use of a wide range of information tools.

Keywords: MEDLINE, PUBMED, libraries, surveys, data analysis, patient care, critical incident technique, physicians, residents, nurses.

Beyond Systematic Reviews: Finding Our Place in the Academic Research Process

Caitlin Pike, AHIP

Health Sciences Librarian, University Library, Indianapolis, Indiana

Rachel Hinrichs

Health Sciences Librarian, University Library, Indianapolis, Indiana

Objectives: Are library-based research services addressing the practical needs of faculty? Could librarians be neglecting a range of opportunities in the research process by only focusing on systematic reviews? Two medical librarians at a large, Midwestern institution sought to integrate themselves into the research processes for their respective schools by using a holistic approach towards embedded librarianship in an academic environment.

Methods: Across the research lifecycle, we have created a culture of acceptance for librarians to be included at every step. In the beginning, many faculty members were not aware of how we could help them beyond systematic reviews. As liaisons, we first began by marketing our services to junior faculty by finding relevant grant funding, conducting literature reviews that supported their research projects, and writing the methods sections regarding searches for any articles that resulted from our work. Following the publication of an article, we also helped with any public access compliance needs, worked to archive their manuscripts, and provided them with a metrics assessment for promotion and tenure purposes. We have actively worked towards being included in all aspects of faculty research, and have three years of combined data to endorse our program of embedded research support.

Keywords: academic, faculty, research, embedded librarianship

Managing a Biomedical Library's Instruction Program: Redefining Scope

Holly Thompson

Informationist, NIH Library, Bethesda, Maryland

Objectives: A Biomedical Research Library with a robust instruction program sought to realign the program to meet the needs of the institution while maintaining levels of attendance and customer satisfaction. The library has a curriculum that spans a wide-range of class topics, including core library

classes in database searching and information management, to specialized classes in data analysis tools and software.

Methods: Class topics, attendance data for the prior two fiscal years, and library policy were analyzed to determine the ideal frequency, rotation, and topics based upon class attendance and instructor skill sets. Additionally, student evaluation forms were consulted for overall satisfaction with the program. With this information, the head of the instruction program and a library staff team leader developed a year-long master schedule with the ideal frequency for each class to occur. Space was left in the schedule for new classes and grouped into main topic areas like data sciences and reference services. Finally, classes that lend themselves to a webinar format were identified and marked to be given as either a webinar or hybrid class.

Results:

Conclusion:

Keywords: Instruction, Program Evaluation, Assessment, Library training programs

Where's the Printer? How One Library Dared to Eliminate Printing

Jeff D. Williams

Deputy Director, NYU Health Sciences Library, New York, New York

Ashley F. Curran

Branch Manager, NYU Health Sciences Library, Forest Hills, New York

David DeSimone

Manager, NYU Health Sciences Library (Hosp Joint Dis Branch), New York, New York

Aileen McCrillis

Assistant Director, User Services, NYU Health Sciences Library, New York, New York

Objectives: This presentation describes the decision-making process and impact of eliminating printing at the NYU Health Sciences Library. Factors motivating this decision included planning for a new public facility, moving to a fully digital collection, supporting an iPad requirement for students and residents, changing user behavior, and a desire to eliminate the expense and hassle of printing.

Methods: During planning for a new library space, administration required the development of "business cases" for the potential services and resources under consideration. These cases were constructed through focus group meetings with stakeholders, research into best practices around modern library facilities, and emphasis on how users were expected to behave in the future. Data collected for the business case on printing included a survey of similar libraries, an assessment of available printing resources outside of the Library, and investigation of the cost and projected impact on library staff. Mitigation strategies for users were also identified, including public scanning, providing free USB drives, and the availability of staff printing for "emergencies." A final consideration for eliminating printing was the desire to make the space and equipment as environmentally sustainable as possible.

Results: The Library's new public space opened in June 2016 without providing printing. Subsequently in December, printing was eliminated at another branch of the Library. In both cases, this decision was met with confusion and/or dissatisfaction by some users that assumed printing would be available in the new

space, or had printed previously in the established space. In each case the user was offered alternatives including staff assistance in saving information onto a networked drive or portable storage device. Because user dissatisfaction was anticipated, there was preparation for significant expressions of dissatisfaction such as formal requests to the Library's Administration to reconsider this decision, or staff interaction with angry users. This has not happened. The implementation of "going paperless" is now considered a "non-event." Library staff have observed and experienced benefits from this decision, including: cost reduction, elimination of hassles, messiness, waste, and decreases of sensitive information, e.g., PHI, being left at printers. Eliminating printing also gives library staff the chance to educate users on alternative ways of retaining and accessing information.

Conclusion: Despite reservations about eliminating a service our users had learned to expect, they have demonstrated adaptability in developing new ways access and retain needed information.

Keywords: Evidence-based librarianship, User services, Management, Library facilities

Monday, May 29, 2017, 10:30 AM – 11:55 AM

Room: 611

Session: Clinical Rounding: Tips from the Field

Moderator: Angela Spencer

Clinical Rounding: Tips from the Field

Angela Spencer

Manager - Medical Library, C. Alan McAfee MD Medical Library, Chesterfield, Missouri

Christine Caufield-Noll, AHIP

Manager, Library Services, Harrison Medical Library, Baltimore, Maryland

Patricia Wynne

Associate Director, Library Information Services, Thomas Jefferson University Scott Memorial Library, Philadelphia, Pennsylvania

Kristen DeSanto, AHIP

Clinical Librarian/Assistant Professor, University of Colorado Anschutz Medical Campus, Aurora, Colorado

Elizabeth Laera, AHIP

Medical Librarian, Brookwood Baptist Health, Birmingham, Alabama

Tracy C. Shields, AHIP

Reference and Clinical Medical Librarian, Naval Medical Center Portsmouth (Navy), Portsmouth, Virginia

Judy C. Stribling, AHIP

Assistant Librarian, Samuel J. Wood Library & C.V. Starr Biomedical Information Center, Weill Cornell Medical College, New York, New York

Kristy Steigerwalt

Clinical Medical Librarian, University of Missouri - Kansas City, Kansas City, Missouri

Elizabeth Stellrecht

Liaison, School of Dental Medicine, Health Sciences Library, Buffalo, New York

Jonathan Hartmann

Senior Clinical Informationist and Head, Data Management, Georgetown University Medical Center, Washington, District of Columbia

Objectives: Educate attendees on perspectives, tips, and scenarios from librarians and others who participate in patient care rounds.

Methods: Panel will discuss roles, competencies, barriers and evaluation of patient rounding in a variety of settings. Panelists will discuss how they got started, what they are currently doing, best practices and tips for getting started and evaluation of program’s effectiveness.

Keywords: clinical librarian, rounds, rounding, hospital librarian

Sponsors: Clinical SIG, Federal Library Section, HLS

Monday, May 29, 2017, 10:30 AM – 11:55 AM

Room: 615

Session: Closing the Loop: Using Data Analysis and Visualization to Get the Most out of Your Library Assessment

Closing the Loop: Using Data Analysis and Visualization to Get the Most out of Your Library Assessment

Laura Menard

Health Sciences Librarian, Ruth Lilly Science Library, United States, Indiana

Objectives: This session will address one of the most difficult aspects of assessment and user feedback: closing the loop. Presenters from health sciences libraries ranging from a large R1 institution to a small liberal arts university will discuss projects they they have undertaken at their own institutions, giving the

audience case studies to consider. Incorporating strategies for customization and scalability, the presenters will walk the audience through the steps necessary for conducting data-driven assessment and synthesizing and sharing their assessment data in an understandable fashion to drive results. Upon completion of this session, attendees will: Identify possible sources of user feedback (surveys, gate counts, usage stats, etc) at their own institutions Become familiar with multiple tools for data analysis and visualization Understand the steps that they must take to implement and follow through on a data-driven library marketing, assessment, and/or outreach campaign. The audience will participate in the following ways: Identify successful and failed data gathering or assessment campaigns at their institutions Share possible sources of data and data collection methods at their institutions Discuss possible barriers to implementing a comprehensive, data-driven assessment plan and how those barriers may be surmounted Identify possible institutional partners for a successful small to large scale library assessment.

Keywords: assessment, data analysis, data visualization, marketing, collaboration, outreach, administration

Monday, May 29, 2017, 10:30 AM – 11:55 AM

Room: 613

Session: Engaging Strategies to Teach Information Literacy to Distance Learners

Moderator: Rachel C. Lerner

Engaging Strategies to Teach Information Literacy to Distance Learners

Molly Knapp, AHIP

Training Development Specialist, NNLM Training Office, Houston, Texas

Margaret A. Hoogland, AHIP

Clinical Medical Librarian, Mulford Health Sciences Library, The University of Toledo, Temperance, Michigan

Terri Ottosen, AHIP

Consumer Health Coordinator, Health Sciences & Human Services Library, University of Maryland, Baltimore, Baltimore, Maryland

Virginia Pannabecker, AHIP

Health Sciences Research Support Coordinator, University Libraries, Blacksburg, Virginia

Distance learning is a growing area of focus in today’s medical schools and health sciences departments. Libraries are seeking new and innovative ways to teach information literacy to distance learners who are not based on the physical campus, or may be attending classes and completing coursework online. What are some engaging strategies to reach these learners, and to teach them research skills and information

literacy? What are good methods of evaluating whether our approach is working? This special content session will feature a panel of speakers who will share their strategies of teaching and evaluating information literacy, and how to apply these lessons at your own institution and within your own library.

Monday, May 29, 2017, 10:30 AM – 11:55 AM
Room: 612

Session: Lightning Talks 2

Moderator: Jeffrey G. Coghill, AHIP

Developing a Library Clinical App to Bring the Evidence to the Point of Care

Michael Kronenfeld

University Librarian, AT Still Memorial Library, Mesa, Arizona

Harold Bright IV, AHIP

Electronic Resources Librarian, A.T. Still University of Health Sciences, Mesa, Arizona

Objectives: In 2005 Kronenfeld co-wrote an article calling for the development of an "Evidence-Based Health Care" model based upon clinicians in clinical settings accessing "secondary, action-oriented resources to quickly locate applicable answers which can be rapidly review and applied'. The focus of this project was to develop an app to provide access for ATSU students to use in their clinical rotations.

Methods: The Library contracted with Boopsie for Libraries to develop an app to facilitate quick, easy access to quality secondary clinical resources. The requirements for the app were: Rapid access not requiring authentication at each app use; Ability of Library staff to select and update resources included in the app; Functionality with both Apple and Android platforms; Multiple profiles supporting different health fields such as medicine and dentistry. While the Library's Electronic Resource Librarian worked with Boopsie in the development of the app the Library's Liaisons worked with their schools in selecting the resources in their professions to be included in the initial launch of the app. After extensive testing the app was launched with each Liaison leading its marketing to the schools they worked with.

Results: The app was formally launched in July with marketing efforts led by the Liaisons for each of the University's six schools. The initial response has been positive. In the first half of September, the app was downloaded 296 times and 1610 queries were made. In January a six month review of the apps usage will be prepared. In addition, focus groups of students currently in clinical settings will be held to assist in evaluating use cases and usefulness. This evaluation will be used in possible changes in the content and organization of the content in the app.

Conclusion: Clinicians have neither the time, access , or skill to use the primary literature in making clinical decisions at the point of care. The need is for efficient access to quality secondary sources to quickly answer specific clinical questions at the point of care. This presentation will introduce a new app the Library staff developed to support this access to and use of evidence.

Keywords: Evidence-based Practice, clinical settings, clinical rotations, point of care

Not Going as Planned: Lessons Learned from Co-Teaching a Case-Based Learning Activity

Charlotte Beyer, AHIP

Instruction and Reference Librarian, Boxer Library, Rosalind Franklin University of Medicine and Science, North Chicago, Illinois

Objectives: To describe a librarian's experiences of developing a pair of sessions with a faculty member from a department of family medicine for the third year family medicine clerkship. Although originally successful in the first year, ran it's course in the second year. Lessons learned from these sessions will be discussed, giving others factors to consider when designing similar instruction.

Methods: In 2014, a librarian was approached to collaborate with a faculty member in Family Medicine on designing a pair of case based learning sessions on the topics: Controversies in Medicine and Chronic Diseases. These sessions would be presented to each of the six family medicine clerkship blocks for a total of twelve sessions. The lessons were in a case based format with students being asked to answer questions on a particular clinical case. During the session the instructors (faculty member and librarian) would circulate as content experts, the family medicine faculty as the clinical expert and the librarian as the information one. The librarian created handouts for both types of sessions that were given out at the beginning of the activity. Assessment was done using a short three question survey and the answers from their worksheet.

Results: From July 2014 June 2015, students were engaged, and noted in the survey how helpful it was having a librarian present. When examining the worksheets, instructors notice students utilizing a variety of resources, and remarking how the information enhanced their clerkship experience. By July 2015 the library had purchased a few point-of-care databases that students became overly reliant on, and questions dramatically decreased for both the physician and librarian. Even with prompting, students only asked a handful of questions in the three hour sessions. In December 2015, it was mutually decided to end the librarian's direct involvement.

Conclusion: In December 2015, the instructors met to discuss why the activity ceased to be beneficial to both parties. Some of the factors identified included students becoming too reliant on one or two resources, information literacy instruction coming too late, objectives changing as time progressed, and the variability of students willing to initiate questions. Even though this activity did not go as planned, it was not a total loss as the process of collaborating opened the lines of communication between medical faculty and the library, highlighting librarians as partners in educating future physicians hopefully opening up new opportunities in the future.

Keywords: instruction, information literacy, faculty collaboration, third year medical students, medical education

SPACE INVADERS: How Hospital Library Space Reduction Can Be a Good Thing

Heather J. Martin, AHIP

Director, System Library Services, Providence Health & Services, Portland, Oregon

Basia Delawska-Elliott, AHIP

Medical Librarian, System Library Services, Portland, Oregon

Objectives: To create a modern and collaborative hospital library space on a shoe-string budget.

Methods: As a non-revenue generating department hospital library space is often at risk when other departments are looking to expand. Recognizing that the current physical library no longer met the needs of its patrons but lacking funds for a renovation, Library leadership set themselves up as a willing and cooperative partner by proactively approaching Administration about relinquishing physical space. When an opportunity presented itself, Library leadership collaborated with Administration and the Hospitalist department to surrender 2/3 of the physical footprint in exchange for a smaller, yet more modernized space.

Results: The Providence St Vincent Medical Library renovation allowed the library to take advantage of existing capital project funds to design a compact twenty-first century space which focused on technology and collaborative work space instead of a large yet dated print-based repository.

Conclusion: Hospital library space reduction isn't always a bad thing. By presenting yourself as a pro-active collaborator, harnessing existing capital project funds can allow libraries to achieve modernization while reducing space in a way that doesn't feel like a sacrifice.

Keywords: space, management, hospital libraries

Adventures in Scholarly Publishing: An Elective Course for M4 Students

Rebecca Welzenbach

Director, Strategic Integration and Partnerships, Michigan Publishing, Ann Arbor, Michigan

Marisa Conte

Assistant Director, Research and Informatics, Taubman Health Sciences Library, Ann Arbor, Michigan

Objectives: The publishing division of (redacted) partnered with the Medical School to launch a student-run journal and developed a for-credit elective in scholarly publishing. Librarian colleagues across campus collaborated on the course design and delivery. This elective, open to all M4 students and required for editors, prepared students for leadership roles on (name redacted), a student-run, peer-reviewed medical journal.

Methods: We will provide an overview of the journal, including its origins, history, publication model and editorial policies, before devoting most of the presentation to the elective.

This week-long workshop is intended to introduce students to the major issues in medical writing, editing, and publishing, and is held twice per academic year. The elective content covered 4 main areas, including scientific writing, scientific data display, the standards for selection and editorial processes in scientific publishing, ethical issues in scientific publishing. Additionally, students established and worked to improve their online publication profiles, while learning about bibliometrics.

Course assessment details, including results of student evaluations, will be provided.

Results:

Conclusion:

Keywords: journal, scholarly publishing, medical education, ethics, publishing

Kinesiology: Moving the Science of Movement into a New Relationship with the Health Sciences Library

Jacqueline Freeman

Informationist, Taubman Health Sciences Library, Ann Arbor, Michigan

Whitney Townsend

Coordinator, Health Sciences Executive Research Service, Taubman Health Sciences Library, Ann Arbor, Michigan

Objectives: The purpose of this project is to document the steps, stages, challenges, and opportunities of building a new relationship between the library, informationists, and faculty that are transferrable to other new relationships that the library would like to build.

Methods: In 2016, the health science library at our university began partnering with the School of Kinesiology, which had previously partnered with a different university library unit. This became a new area of focus for our library and our instructional and curriculum development, offering an opportunity to plan our learning and teaching strategy (what we teach, when we teach it, how we teach it, and how we measure learning.) We will describe our approaches to building this relationship, focusing on emerging pertinent issues, including the incorporation of specialized resources, knowledge, and needs that had not previously been part of health sciences; developing a program of immersion in a new academic area; and managing expectations on both sides for what engagement and partnership look like.

Results: We will present the current state of our relationship with this school and our plans for our next steps in building this relationship.

Conclusion: Results of our project will be made available during our presentation at MLA '17.

Keywords: relationship building, kinesiology, embedding, partnering, liaison role

Designing and Implementing a Microsoft Access Database for Patron Interaction Tracking: A Budget-Friendly Reference Statistics Solution

Lydia Howes

Research and Education Librarian, Medical Library, Sptringfield, Illinois

Carol Gordon

Education & Research Librarian, Medical Library, Springfield, Illinois

Objectives: While tracking, managing, and analyzing reference statistics is essential for libraries, the process can be burdensome and commercial solutions are expensive. A small, academic medical library upgraded their system of tick-marks and Excel spreadsheets by designing a custom database and form in Microsoft Access. The new system provides greater flexibility, precision, and the ability to quickly access and analyze data.

Methods: The old system required staff to transcribe paper records into Excel spreadsheets, which was time consuming and could introduce error. Budget constraints eliminated commercial products, while free web-based solutions such as Google Forms would not improve upon Excel's analytical abilities. Access is included in the Microsoft Office suite of programs already licensed by most institutions, and it provides greater flexibility for data analysis than Excel. After identifying which metrics to record, we constructed the database according to basic relational database design principles. It is divided into a front-end (form) and a back-end (data) stored on the library's shared server. The library began Beta-testing the new tracking

system at the start of the 2017 fiscal year. For the first several months staff recorded patron interactions using both the new electronic and old paper forms, allowing for glitch fixes and other improvements.

Results: Beta-testing ended in October 2016 when the Patron Interactions Database completely replaced the old paper tracking system. The database makes accessing and interpreting data easier and more convenient and has already been useful in decision making: The detailed statistics collected in the database supported the decision to close the Medical Library on weekends.

Conclusion: For many libraries Microsoft Access can be an effective, inexpensive solution for interaction tracking. Access is a resource already available to many institutions and can handle the needs of library systems of varying sizes. However, there are other costs and requirements to consider when selecting a tracking solution. Development and maintenance demands at least basic familiarity with database principles and requires significant staff time and effort, particularly in development. Cooperation and coordination with IT may be necessary depending on database complexity and the library's computing setup. For this library, a custom Microsoft Access database has already proven to be an excellent investment that will save time and increase statistic practicality and use.

Keywords: reference statistics, Microsoft Access, inexpensive solutions

After the Systematic Review Search: A Case Study on Responding to Peer Reviewers' Comments on Systematic Review Manuscript Submissions to Medical Academic Journals

Sarah Bonato

Reference and Research Librarian, CAMH Library Services, Toronto, Ontario, Canada

Objectives: The peer review process is an essential component of journal article submission. When librarians participate as part of the research team, it's likely they will be asked to respond to peer reviewers' comments when the manuscript is submitted to a journal. This paper describes the process, challenges and the lessons learned when a librarian must respond to peer reviewers' comments.

Methods: Peer review is important for selecting quality articles for publication, improving the article for publication and for assessing the scientific merit of the article methodology. Often, even though the peer reviewers are not librarians, they will provide commentary on the execution of the search strategy and search methods and ask for justification for the librarians' choice of search methods. This paper will provide a case study of one librarian's experience of how non librarian peer reviewers critique manuscript submissions and how the librarian responded to reviewers' questions. Examples of the specific questions asked and how the librarian responded will be discussed. Key recurring themes of common questions regarding a librarian's choice of search methods will also be identified. Strategies and suggestions on how to respond to peer reviewers' comments will also be addressed.

Results: Results will be shared at the presentation

Conclusion:

Keywords: Systematic reviews

Peer review

Librarians as co-authors

Search strategies

Being Mortal and Beyond: Meeting Spiritual Needs of Staff and Caregivers

Mary Shah

Medical Librarian & Archivist, Horblit Health Sciences Library, Danbury, Connecticut

Objectives: This work examines the development of a partnership of the Danbury Hospital librarians, the Spiritual Care Department, and the newly formed Goldstone Caregiver Center: broadening its outreach to the staff, as well as the patients and their caregivers in the community.

Methods: At Danbury Hospital, a non-profit teaching hospital, health sciences librarians, the Director of Spiritual Care and her staff as well as the manager (a licensed social worker) of the Goldstone Caregiver Center. The purpose of this partnership hoped to provide easily accessible and understandable information to the staff of the hospital and the caregivers in the community. The first step in the partnership was to transfer the consumer health collection from the library (which is focused on the information needs of the staff) to the caregiver center which promotes the well-being of caregivers through compassionate support and a healing environment. The transition was so successful, the professionals decided to work together on an outreach program: a discussion series on the book "Being Mortal" by Atul Gawande for the staff of the hospital.

Results: There were a series of six weekly sessions held for the staff at various times to meet the needs of all shifts. The most attended session was at noon, followed by the evening then the early morning. Librarians teamed with a staff from the spiritual care department to facilitate all of these discussions. Sessions were held at the Caregiver Center. Using the book as a framework, the groups discussed aging and the process of dying as well as the cultural and individual perspectives in a judgment-free zone without hierarchy. I have since facilitated a one-time book discussion with summer research interns. I have created annotated bibliographies for the Caregiver Staff as well for their outreach programs.

Conclusion: Though the topic of the discussion can be emotionally overwhelming, those who participated in the program found it worthwhile. The discussion groups met the stated goals of discussing aging, complications, hard conversations, and mortality. Most participants stated that the talks changed their practice and personal lives as well as their competency in approaching these subjects. When asked about barriers to change, lack of time as well as difference of opinions were the most frequently listed. Most participants also suggested having fewer discussions per book in the future.

Keywords: Spiritual Care, End of Life, Dying, Books, Discussion, Outreach, Partnership

Partnering Across Campus to Implement an Electronic Laboratory Notebook System at the University of Michigan

Tyler Nix

National Library of Medicine Associate Fellow, University of Michigan Taubman Health Sciences Library, Ann Arbor, Michigan

Marisa Conte

Assistant Director, Research and Informatics, Taubman Health Sciences Library, Ann Arbor, Michigan

Objectives: Electronic laboratory notebooks (ELN) offer many advantages over traditional paper notebooks including: providing a secure platform for research data collection, facilitating data archiving and sharing, and assisting with research compliance. The authors partnered with a health system IT team to provide training and documentation to health sciences researchers, and led a cross-library team to promote a new ELN implementation at the University of Michigan.

Methods: This project centers around two discrete collaborations. In the first, we partnered with IT colleagues to 1) participate in the Request for Proposal, 2) develop outreach materials, and 3) create

customized training and documentation. Our training targeted basic and clinical health sciences researchers from 5 different schools and integrated the ELN with existing library data services.

For the second collaboration, we assembled and led an interdisciplinary team of library stakeholders from across six different campuses. The team developed training materials for subject specialist librarians in a variety of departments. We also coordinated library-wide outreach to raise awareness about the new ELN.

In addition to addressing the benefits and challenges of these collaborations, we will: provide background information on the ELN system, share examples of training and promotion materials, and detail next steps for both collaborations.

Results: Will be made available during the conference presentation.

Conclusion: Will be made available during the conference presentation.

Keywords: Electronic Lab Notebook, research data management, collaborative projects

A Guide to Critical Appraisal Tools and Reporting Guidelines: Matching Purpose and Type of Evidence with the Appropriate Tool

Claire O. Sharifi

Reference Librarian: Liaison to School of Nursing & Health Professions, Gleeson Library/ Reference Department, San Francisco, California

Robin Buccheri

Professor, School of Nursing and Health Professions, San Francisco, California

Objectives: This project reports on the creation of a guide to the often overwhelming terrain of critical appraisal tools and reporting guidelines. This guide will help both novice and experienced nurse researchers quickly select the best tool(s) to use for critical appraisal and the reporting of research.

Methods: The critical appraisal of evidence is an essential step for nurses who are designing an evidence-based project, quality improvement project, or research study. Nurses need to be able to efficiently locate and use critical appraisal tools and reporting guidelines. There are a plethora of critical appraisal tools and reporting guidelines available, from tools that are specific to an individual methodology or study type, to more generic tools that can be applied to many types of evidence. The co-authors of this project found that graduate and doctoral nursing students struggled with selecting appropriate critical appraisal tools, and a clear, easy to understand guide was needed. A comprehensive search was conducted to identify commonly used critical appraisal tools and reporting guidelines in nursing.

Results: Selected tools were evaluated, summarized in plain language, and categorized based on the study type they were developed for. This guide includes a clear description of each tool, as well as a table that allows researchers to quickly and easily identify an appropriate tool.

Conclusion: The guide has not yet been evaluated, but we expect it will improve students' ability to identify critical appraisal tools.

Keywords: evidence based practice

critical appraisal

reporting guidelines

nursing education

Dare to Invite Yourself to the Table

Alissa Fial

Education & Research Services Librarian, McGoogan Library of Medicine, Omaha, Nebraska

Emily J. Glenn, AHIP

Education & Research Services Librarian, McGoogan Library of Medicine, Omaha, Nebraska

Teresa Hartman

Associate Professor / Education & Research Services Librarian, McGoogan Library of Medicine, Omaha, Nebraska

Objectives: As strategic education partners and leaders, library faculty can help improve an institution's education programs, leading to improved student learning experiences. This paper presents strategies for librarians to embrace their role at an academic medical center by growing expertise in teaching modalities, becoming an integral member of campus education teams, and leading inquiry and research-focused curriculum change objectives.

Methods: Academic medical librarians have distinct opportunities to serve clinical faculty beyond their biomedical information and research needs. At one institution, librarians leveraged their university committee service to gain appointments on teams that steer education and learning: the College of Medicine curriculum redesign group, a newly formed teaching academy, and a faculty development committee. Librarians recognized opportunities, built relationships, improved their own skill sets, accepted the challenges of the new roles, and sharpened their professional contributions as faculty members in each of their roles. These three case studies highlight experiences of faculty librarians as partners within an institution's education infrastructure and describe outcomes beneficial to the campus community. Benefits for the librarians include increased confidence, deepening one's relationship with the campus culture, and moving towards integration into Interprofessional programs.

Results:

Conclusion:

Keywords: professional development

faculty role

campus culture

education

curriculum

interprofessional

"What Type of Review Are You?" An Interactive Game to Teach Users about Review Literature Typology

Nha Huynh

Education and Instruction Librarian, Client Relationship Management, Houston, Texas

Objectives: With the proliferation of systematic reviews comes numerous misconceptions about the actual methodology of conducting the review itself. “What type of review are you?” is an interactive online quiz-style game that aims to educate researchers of different types of reviews and help them decide which will be most suitable for their research topic.

Methods: The author gathered information on review types from PubMed sources and adapted the concepts to a more user-friendly multiple choice format. Built with JavaScript, the game uses simple algorithms to match the player’s answers to predetermined parameters (such as time availability, number of reviewers, purpose of the review) and gives user a “best match” review suggestion at the end. A link to the game is posted on the TMC Library's EZProxy login page to encourage users to play. The author also included links that redirect to The TMC Library website’s Literature Search and Consultation Services request forms, should the player wishes to contact a librarian for assistance with their project.

Results: N/A

Conclusion: Librarians are always looking for effective ways to teach about systematic reviews. This interactive game provides an interactive spin on useful theoretical information researchers should know when selecting the appropriate type of review to fit their projects.

Keywords: gamification, systematic review, literature reviews, reviews, game, instruction, technology, web

Informationists and Library Impact on Patient and Population Care: Strategies, Process, and Practicalities

Judith Smith

Informationist, University of Michigan, Ann Arbor, Michigan

Jean Song

Assistant Director, Academic and Clinical Engagement, Taubman Health Sciences Library, Ann Arbor, Michigan

Carol Shannon

Informationist, Taubman Health Sciences Library, Ann Arbor, Michigan

Mark P. MacEachern

Informationist, Taubman Health Sciences Library, Ann Arbor, Michigan

Gurpreet K. Rana

Global Health Coordinator, Taubman Health Sciences Library, Ann Arbor, Michigan

Jacqueline Freeman

Informationist, Taubman Health Sciences Library, Ann Arbor, Michigan

Objectives

Informationists are embedded into the work of academic and clinical partners, but the impact of their role is not always clear. This project describes the process at the Taubman Health Sciences Library (THL) at the University of Michigan to better understand its impact on patient and population care within a large university health system.

Methods:

An assessment working group at THL sought to develop and pilot an evaluation program to determine how well the library was meeting the information needs of its academic and clinical partners in a complex university health system. The group first defined five broad assessment categories (education/learning, research, patient and population care, service delivery, internal processes), eventually focusing on patient and population care. A subsequent working group was formed to determine how well the library is working with providers, faculty, staff and students to identify, appraise, and apply information to provide high quality care by: 1) developing a plan for conducting an assessment; 2) creating instruments to implement assessment in this area; and, 3) writing a final report stating the accomplishments of the group and results from the implementation plan.

Results:

Developing a plan for evaluating the library's impact on patient and population care provided challenges surrounding research methodology for measuring a population for which there is no direct contact (i.e., information provision directly to patients). Through literature searching and discussions with faculty leaders at Michigan Medicine and the Department of Learning Health Sciences, informationists honed in on specific evaluation research: conducting a mixed methods, exploratory sequential design study. Informationists will survey THL staff to help identify stakeholders, and will subsequently conduct qualitative interviews to measure impact. Based on themes elicited, the team will create a second quantitative survey instrument for broader reach.

Conclusion:

This study is one component of the library's assessment planning. Using a mixed methods exploratory sequential design, the assessment working group has created a plan for the project, which will consist of individual interviews that will inform our further work, which is the deployment of survey instruments to appropriate stakeholder groups. The assessment task force also will conduct other assessments in conjunction with its mission, such as informationists' role in guideline creation and citation analyses of our published work.

Keywords:

Assessment, evaluation, patient care

Monday, May 29, 2017, 10:30 AM – 11:55 AM

Room: 608

Session: Dreaming of a Healthier Community: Librarians Doing Research

Moderator: Montie' Dobbins, AHIP

The Effects of a Telemedicine Health Information Prescription in Uncontrolled Hypertensive Patients in a Family Medicine Ambulatory Clinic

Erica Lake, AHIP

Associate Librarian, Spencer S. Eccles Health Sciences Library, Salt Lake City, Utah

Bernadette Kiraly

Medical Director, Sugar House Health Center; Associate Professor, Family and Preventive Medicine, Sugar House Health Center, Salt Lake City, Utah

Rachel Christensen

Student - Research, Family Medicine, Salt Lake City, Utah

Kimiya Nourian

Medical Assistant, Sugar House Health Center, Salt Lake City, Utah

Objectives: Only 50% of hypertensive patients have their blood pressure under control despite treatment. Limited understanding of hypertension contributes to poor outcomes. Patients who receive materials customized to health literacy level and learning style show gains in knowledge. Our study aims to improve blood pressure control and increase self-management in hypertensive patients by utilizing librarians and telemedicine to provide tailored information.

Methods: In this randomized, unblinded feasibility study, we recruited 50 adults with uncontrolled hypertension. All participants completed five validated assessments and questionnaires before and after the study intervention: Single-Item Health Literacy Screener; High Blood Pressure Questionnaire (assesses patient disease knowledge); Learning Style Preference assessment; Patient Activation Measure; and Press Ganey Patient Satisfaction survey. The intervention group received an information prescription to visit with the health librarian via the health information kiosk (a dedicated clinic computer equipped with telemedicine technology). Prior to the visit, the librarian reviewed the patient's chart in the electronic health record and the assessment/questionnaire results in order to tailor the educational materials provided. The control group received standard patient education and information from their primary care providers. Participants' providers were also surveyed about their satisfaction with the intervention and with librarian involvement on the health care team.

Results: Results are pending - the six month post-assessments are currently being conducted. A qualitative analysis with kiosk session participants will be conducted. We will have results to present by May 2017.

Conclusion: Results are pending -we will have conclusions to present by May 2017. We hypothesize the telemedicine provision of tailored patient education by health librarians outside of the provider-physician visit in patients with uncontrolled hypertension will reduce the time to achieve blood pressure goal, increase patient activation and self-management practice, increase patient satisfaction with their care, increase patient hypertension-specific health literacy, and increase provider satisfaction as compared to standard care.

Keywords: Information prescription Hypertension High blood pressure Telehealth Tailored health information Patient outcomes Patient education Inter-professional team-based approach to health care delivery

Health Insurance Information Needs: How Librarians Can Help

Emily Vardell

Teaching Fellow, School of Information and Library Science UNC Chapel Hill, Chapel Hill, North Carolina

Objectives: It is a widely perceived but poorly documented problem that many individuals lack clear understanding of health insurance. Librarians can address the unmet information needs that leave many unable to make appropriate health insurance choices. For those with lower levels of health insurance literacy, the ability to procure appropriate levels of health insurance coverage may be limited, which can have dire effects on individuals' health statuses.

Methods: This study employed semi-structured interviews to explore how newly hired employees at a large university in the southeastern United States understand health insurance concepts and make health insurance purchase decisions. This paper will offer examples of the information needs they described and an analysis of the trends across individuals.

Results: Participants divulged a lack of understanding of insurance-related terminology (e.g., coinsurance). Their confidence in navigating the health insurance system decreased as education levels increased (i.e., those with a doctoral degree reported the lowest level of confidence in navigating health insurance enrollment). Participants also expressed difficulty in locating insurance information resources that were unbiased. These findings underscore an important role that librarians can play in providing access to unbiased, authoritative definitions of health insurance terms.

Conclusion: The collected data form the foundation for the construction of a model of the health insurance decision-making process and offer insight to the library and information science community on how to support health insurance information needs. The proposed model and discussion demonstrate continued difficulty with understanding health insurance concepts and the factors which impact health insurance literacy and decision-making.

Public Libraries in the Age of the Affordable Care Act

Alla Keselman

Senior Social Science Analyst, Specialized Information Services, Bethesda, Maryland

Catherine A. Smith

Associate Professor, University of Wisconsin-Madison, Madison, Wisconsin

Janice E. Kelly, FMLA

Acting Deputy Associate Director, Specialized Information Services, Bethesda, Maryland

Colette Hochstein

Technical Information specialist, Specialized Information Services, Bethesda, Maryland

Objectives: The investigators' previous research showed that public libraries are challenged by the public's information needs in the health and financial domains. The Patient Protection and Affordable Care Act (ACA) has ingredients of both. What have been the challenges of information provision about the ACA, and what has been the impact on public libraries and library workers?

Methods: The principal investigators developed an online survey targeting librarians at 1500 central libraries located in 20 of the 50 U.S. States: 10 with federal health exchanges for enrollment under the ACA; 10 with state exchanges. Initial invitations were sent by mail to library directors and heads of

reference/public services, identified in the IMLS Public Libraries Survey dataset. Survey respondents were asked 22 questions and could submit free-text responses when appropriate. Topics of questions included: online resources used to answer patrons' health information questions; specific training received about the ACA; agency and community partners; specific activities the library has engaged in around the ACA enrollment periods; and opinions of information resources available from NLM and other sources. People completing the survey were eligible to enter a raffle to win an iPad for their library. The survey ran from September to November 2016 and achieved a response rate of 32%.

Results and Conclusion: Will be made available during the presentation at MLA 2017.

Keywords: Affordable Care Act; public libraries; health information resources; consumer health; surveys

Analyzing South Carolina Public Libraries Reach through Twitter Health Information Dissemination

George Shaw, Jr.

Student, School of Library and Information Science, Sumter, South Carolina

Objectives: Twitter has grown in popularity since its inception in 2006. Libraries have increasingly adopted social media sites (SNS) such as Facebook, Instagram, and Twitter to communicate with patrons regarding services. Health librarians have utilized social media tools to distribute health information to their patrons, but few studies have examined the exposure/reach of health related tweets by public libraries. Moreover, what type of health information is normally shared by public and health libraries in Twitter.

Methods: This research study seeks to expound upon exploratory results from a previous study that analyzed tweeted health information by six public libraries in the state of South Carolina. Using text mining techniques with R programming, Twitter data will be collected for a 12-week period to compare the health information retweeted and liked (favorites) by public and two prominent health libraries. Eight public libraries and two health libraries in the state of South Carolina will be used for this study. The study analyzes the number of retweets and favorites related to the health information tweeted. Using a qualitative research technique for data analysis, a content analysis will be conducted to identify the type of health related information disseminated by public and health libraries.

Results:

Data is still be collected and analyzed. Complete results will be discussed and available during the conference.

Conclusion: *will be available during conference presentation.*

Keywords: text mining; public library; social networking; health communication.

What Roles Do Chinese Health Sciences Libraries Play in Their Nation's Cigarette Smoking Public Health Crisis?

Charles J. Greenberg, AHIP

University Library Director, Wenzhou-Kean University, Wenzhou, Zhejiang, China (People's Republic)

Lin Wu, AHIP

Instructional Associate Professor / Pharmacy and South Texas HSC Campus Librarian, Medical Sciences Library, Kingsville, Texas

Xuyu Zhou

Bodong Chen

Student assistant, Wenzhou- Kean University, Wenzhou, Zhejiang, China (People's Republic)

Yutong Feng

Student, Wenzhou Kean University, Wenzhou, Zhejiang, China (People's Republic)

Objectives: Cigarette smoking remains a major cause of death in China. Second-hand smoke in China is a public health problem. A 2011 Chinese study estimated 16.5% of all deaths in men and 1.7% in women were smoking-related. Are health sciences libraries in China currently providing awareness, advocacy, or research support for the societal benefits of reducing dependence on tobacco products?

Methods: An institutional review board reviewed the proposal for protection of human research subjects. Library contacts for Chinese schools of medicine, public health, and pharmacy were identified, using the World Directory of Medical Education to identify Chinese schools. Authors used social networks and institutional websites to obtain the names and email addresses of participants. Individual names or identities would not be reported. A bilingual Chinese-English Qualtrics® online survey was constructed and distributed to Chinese health sciences libraries to obtain respondents' demographic detail, anonymous personal information, and answers to questions about library resources and services that constitute academic awareness, advocacy, curriculum, or research support about tobacco and smoking and the consequences of long-term smoking and tobacco use. Respondents were offered the opportunity to submit their contact information to receive copies of completed research.

Results: Survey responses were received from librarians at medical centers and schools in Beijing, Shanghai, and 13 additional provinces. 43% of reporting librarians work on a smoke-free campus. 100% of all reporting libraries work in smoke-free libraries, though 6% of the reporting libraries offer a smoking room for staff. All reporting libraries contain printed material on the dangers of smoking; 17% report a significant amount. Student requests for materials or acquisition recommendations are infrequent, and responses ranged from occasionally to never. More than 60% of the librarians report medical residents occasionally ask for tobacco-related literature. Nearly 60% of librarians reported faculty occasionally ask for material about the consequences of smoking. More than 60% of instructors were reported to occasionally ask for database searches about cigarettes or tobacco. Only 33% of librarians reported creating a collection guide about smoking. 15% of reporting libraries hosted a traveling exhibit on the consequences of smoking.

Conclusion: Some Chinese health sciences libraries are providing public health information and collaborating with faculty and students to support the reduction of smoking and tobacco use. Anecdotal statements collected from survey participants confirms their awareness of the positive roles librarians play in their country's smoking crisis.

Keywords: China, Public Health, Cigarettes, Smoking, Health, Sciences Libraries, Outreach, Advocacy

Monday, May 29, 2017, 10:30 AM – 11:55 AM

Room: 604

Session: Daring to Dream: Facilitating Scientific Communication

Moderator: Claire B. Joseph, AHIP

The Value of Case Studies in Evidence-Based Librarianship: An Examination of the Journal of the Medical Library Association

Katherine G. Akers

Biomedical Research and Data Specialist, Shiffman Medical Library, Detroit, Michigan

Kathleen Amos, AHIP

Assistant Director, Academic/Practice Linkages, Public Health Foundation, Washington, District of Columbia

Objectives: The Journal of the Medical Library Association (JMLA) publishes case studies to enable authors to make a scholarly contribution to the practice of health science librarianship and allow readers to discover new or innovative library services and programs. Here, we describe JMLA's history of publishing case studies and argue for the value of case studies in an evidence-based librarianship context.

Methods: We explored the JMLA's history of publishing case studies articles by examining changes over time in the average of number of case studies published per issue and by interviewing past JMLA editors about their opinions of and editorial policies toward case studies. To assess the perceived value of case reports to readers, we examined the usage statistics of case studies articles as reported by PubMed Central. This data will inform our discussion of the educational and practical value of case studies to both authors and readers, the advantages and disadvantages of case studies compared with more rigorous study designs, and tips for writing high-quality case studies for publication in JMLA.

Results:

Conclusion:

Keywords: case studies, scholarly communication, writing, publishing, innovative library services and programs, evidence-based librarianship, research

Developing a Model for Faculty Scholarly Metrics Services across Diverse Health Sciences Audiences: From Dreaming to Doing

Catherine Pepper

Associate Professor and Library Field Services Coordinator, Texas A&M University, Austin, Texas

Sheila W. Green

Bryan Campus Librarian, Texas A&M University, Bryan, Texas

Arwen Meador

Instructional Assistant Professor / Health & Life Sciences Librarian, Medical Sciences Library, College Station, Texas

Heather K. Moberly, AHIP

Professor / Coordinator of Veterinary Services, Medical Sciences Library, College Station, Texas

Lin Wu, AHIP

Instructional Associate Professor / Pharmacy and South Texas HSC Campus Librarian, Medical Sciences Library, Kingsville, Texas

Bruce Herbert

Director of the Office of Scholarly Communication, University Libraries, College Station, Texas

Esther E. Carrigan, AHIP

Associate Dean and Director, Texas A&M University, College Station, Texas

Objectives: University administrations are increasingly requesting evidence of impact from their faculty in annual evaluations and for promotion and tenure. Librarians are well-poised to assist faculty, scholars, and researchers with gathering and reporting scholarly metrics. This paper describes the activities that an academic medical library has undertaken to establish a scholarly communications service with its diverse clientele and shares lessons learned.

Methods: The task force met to brainstorm a plan to develop a new service model aimed at establishing an overall scholarly metric and impact support service for the library's various client groups. First steps focused on sharing existing individual work and collaborating with the University Libraries' Office of Scholarly Communications (ScholComm), whose director shared expertise and provided training and resources. Librarians next initiated communications with their liaison groups, "shadowed" ScholComm's presentations to these audiences, and developed resources and services that could be shared and adapted. Librarians met with faculty and administrators to demonstrate how to develop faculties' scholarly profiles with citation metrics resources, and created a sequence of activities and tools to build the service and engage target clientele, including the director's charge, needs assessments, basic and advanced training modules, webinars, and templates for initiating conversations and sustaining contact with clientele.

Results: Needs assessments were conducted with various colleges indicating faculty need for support in developing their scholarly narratives. Survey results for the pharmacy school indicated that 50% of the faculty had limited knowledge about scholarly metrics and research impact; 73% have since claimed their scholarly profiles. Nursing tenure-track faculty incorporated citation metrics into their dossiers. Presentations for medical, veterinary, and public health faculties resulted in requests for focused individual consultations on metrics and scholarly identities, and accelerated development of a researcher discovery tool deployed across campus. A tool for automatic calculation of citation metrics was created and adapted for all schools.

Conclusion: The library's partnership with ScholComm has helped establish a new service model for the research and scholarly needs of its client groups in the first of three ScholComm areas: scholarly reputation and impact, open access, and digital scholarship and publishing; the library is now augmenting the model to include the remaining two areas. The service has enhanced the library's value to faculty constituencies into areas most had not previously associated with library activities. Along with practical and adaptable powerpoints, instruments, templates, and tools, we provide an overarching "blueprint" for partnerships among librarians, scholarly communications specialists, and health sciences academic units.

Keywords: Research impact, Citation tracking, Scholarly identity, Researcher metrics, Scholarly Communications

Collaborating with Faculty Physicians to Mentor Residents' Writing: Expanding Clinical Librarian's Role to Support Residents' Scholarly Activities

Young-Joo Lee

Senior Clinical Librarian, Howard University, Washington, District of Columbia

Objectives: Mentoring programs have been recognized as an effective tool in resident training. This study aims to explore how clinical librarians can contribute to mentoring residents in their scholarly activities. The author will demonstrate via an overview of a successful writing mentoring program that she coordinated with faculty and physicians at her institution and explain the challenges faced in doing so.

Methods: The author will first describe the Manuscript Review Committee and their mentoring activities which include hosting monthly workshops and seminars to help residents prepare to write a case report and reviewing residents' manuscripts prior to journal submission. The program evaluation will be mainly qualitative with some quantitative data. The qualitative data will include interviews of participating faculty mentors and residents. Additional quantitative data featured will include workshop attendance, manuscripts submitted to the committee, and usage of workshop materials including presentation recordings and PowerPoint slides. Finally, the author will share her thoughts on her contribution to the program as a clinical librarian as well as the challenges she encountered.

Results: Will be made available during the presentation.

Conclusion: Will be made available during the presentation.

Keywords: Residents Scholarly Activities, ACGME, GME, Mentoring, Publication, Clinical Librarian, Collaboration

Increasing Clinical Scholarship: Promoting the Publication of Medical Case Reports

Katherine G. Akers

Biomedical Research and Data Specialist, Shiffman Medical Library, Detroit, Michigan

Objectives: Publishing a medical case report in a journal is a relatively easy way for clinical faculty, residents, and medical students to engage in scholarship. However, clinicians may face barriers such as low acceptance rates and high author fees. To promote clinical scholarship, our academic medical library introduced new educational sessions and services aimed at promoting the publication of case reports.

Methods: We took a three-pronged approach. (1) To recommend suitable journals, we identified over 160 new, mostly open access journals that focus on publishing case reports. However, many of these journals engage in questionable publishing practices and might be "predatory". (2) To share this information with authors, we developed a "Publishing Case Reports" educational session for clinical faculty, residents, and medical students covering how to write a high-quality case report and choose a reputable journal. (3) To help authors pay for publishing their case reports, we commenced an institutional fellowship to the journal BMJ Case Reports to waive submission fees for affiliated authors. The success of these efforts will be evaluated using information related to the educational sessions (e.g., number of sessions, attendance, feedback from attendees) and BMJ Case Reports submissions (e.g., number of fee waivers distributed, number of published articles).

Results:

Conclusion:

Keywords: scholarly communication, scholarship, publishing, writing, open access, predatory publishing, clinical faculty, medical residents, medical students

Dreaming and Daring Beyond the Traditional: Building an Interdisciplinary Scholarly Community

Katie A. Prentice, AHIP

Associate Director for User Experience and Assessment, University of Oklahoma-Tulsa, Tulsa, Oklahoma

Shelly A. Fowler

Grant Coordinator, Office of the Assistant Vice President for Research - Tulsa, Tulsa, Oklahoma

Objectives: This paper will describe how the Schusterman Library, a small academic library, is working to develop an interdisciplinary scholarly community through unique faculty development workshops, a faculty peer-writing group, and a research-focused student, faculty, and staff informal support group.

Methods: In a campus partnership the Schusterman Library launched a workshop series, a faculty writing group, and a research support group for students, faculty, and staff. Workshop topics included collaboration with the Institutional Review Board, altmetrics, publishing and rejection, grant writing, expert searching, and personal branding. The writing group meets monthly to discuss submissions, share accountability, and provide mutual support for scholarly work. The informal support group is a monthly brown bag lunch for networking and discussion. Each activity is designed to develop scholarship and research in the campus community.

Results: All three programs for scholarly community on campus are popular and well-attended. Faculty who previously have not shown interest in Library services are now making service requests. Students and staff requested the launch of independent writing groups. All three events are hosted in the Library and several participants have been first-time library visitors.

Conclusion: A student-focused Library is able to step into new faculty development and research development activities utilizing campus and community resources and partnerships. As a result of these new initiatives more faculty, students, and staff recognize the Library and librarians as leaders in scholarly and research activities.

Keywords: scholarship, research development, community, writing, faculty development, library roles

Monday, May 29, 2017, 10:30 AM – 11:55 AM

Room: 603

Session: Librarians as Innovative Collaborators: Doing What it Takes to Form New Partnerships

Moderator: Francesca Allegri

Providing Support for an Interdisciplinary Research Group with a Multidisciplinary Informationist Team: Is It Effective?

Margaret Henderson, AHIP

none, none, Ramona, California

Karen Gau

Research and Education Librarian, Tompkins-McCaw Library for the Health Sciences, Richmond, Virginia

Julie Arendt

Science and Engineering Research Librarian, VCU Libraries, Richmond, Virginia

Martha Roseberry

Science and Engineering Research Librarian, VCU Libraries, Richmond, Virginia

John Cyrus

Research & Education Librarian, Tompkins-McCaw Library / Research and Education, Richmond, Virginia

Objectives: Three subject librarians and a data librarian, representing three departments and two libraries at a university, were awarded an NLM Informationist Supplement to support an interdisciplinary Research Group. The Informationist Team developed and tested a model to utilize the skills of multiple librarians to inform future support for the increasing number of interdisciplinary and interprofessional research groups at the university.

Methods: The Informationist Team provided many services, including literature searches, creating search alerts, bibliographic citation management and sharing, and data management support to an interdisciplinary Research Group over a two year period. Librarians routinely attended lab meetings and shared notes with each other in a group document to monitor researcher needs and questions. Methods were developed to insure all Research Group information needs were met. Two new library workshops were also developed after observing researcher needs. Graduate students were encouraged to attend data management boot camps held in the Libraries. Surveys were administered to the Research Group prior to, during, and after a two-year period to assess the effectiveness of an Informationist Team model that employs the skills of multiple librarians.

Results: Surveys administered to the Research Group showed that the group increased their use of appropriate resources to find scientific and technical information over a two-year period. Additionally, by the final survey, all members of the Research Group had worked with a librarian and all felt it had saved them time. The data librarian also facilitated the creation of a form to collect data for a lab protocol that helped the group avoid significant errors.

Conclusion: The relationship between the multidisciplinary Informationist Team and the Research Group was mutually beneficial. The Research Group was able to improve its knowledge and efficiency with the Informationist Team's assistance, and regular librarian attendance at lab meetings enabled librarians to enhance their understanding of basic science research. Having a multidisciplinary team of librarians allowed for sharing the workload and for deeper assistance to the Research Group in specialized areas such as data management.

Keywords: Informationist, team librarianship model, interdisciplinary, interprofessional, data management, biomedical research support,

Monday, May 29, 2017, 10:30 AM – 11:55 AM
Room: 603

Session: Librarians as Innovative Collaborators: Doing What It Takes to Form New Partnerships

Moderator: Francesca Allegri

Paving the Way to Better Coordinated Clinical Research Services using a Interdisciplinary Model: the Translational Research and Information Lab (TRAIL)

Emily Patridge, AHIP

Assistant Director, Clinical Research and Data Services, Health Sciences Library, Seattle, Washington

Tania Bardyn, AHIP

Associate Dean and Director, University of Washington, Seattle, Washington

Objectives: University of Washington's TRAIL was developed to provide focused data and information services to researchers and faculty for research success. TRAIL is a partnership between the Health Sciences Library (HSL), the Department of Medicine and a CTSA funded department.

Methods: TRAIL provides a collaboration lab for researchers using data, technology, and tools to facilitate learning, discovery, and innovation. The TRAIL space was designed by librarians to include a data wall to allow up to 6 to share information and data. During the initial year, HSL cross-trained librarians to support REDCap and teach REDCap beginner classes. An assessment will be done on REDCap training classes, consultations, and number of times the space was reserved. In addition, questions and suggestions will be gathered to assess and improve upon the service in the second year.

Results: The TRAIL initiative is allocating library space, staffing, expertise and leadership to provide integrated support to researchers. Developing a shared understanding of institutional commitments, scaling up collaborations to five partners, and merging existing data support efforts across units is risky. This new partnership within the institution developed a shared service model to help researchers learn how to be innovative and use data walls, REDCap, emerging technology, and collaboration space. We have learned that communication is crucial for buy-in. In two months, 17 groups reserved the TRAIL, including for virtual reality and 3D modeling. Since September 2016, twelve REDCap classes have had 110 attendees. 6 librarians are now teaching REDCap classes and supporting REDCap questions.

Conclusion: It is easier to keep a researcher than recruit a new one, especially given competition for extramural funding. Medical libraries who partner with other organizations and departments to ensure integrated support across the campus, are planning futures that will provide ongoing researcher success, and show that medical libraries help faculty demonstrate research impact. By creating a team and forming an intra-institutional collaboration, the library provided space, the CTSA provided initial REDCap and Team Science expertise, and all are working together to get cross-trained to provide investigators a consistent way to access librarians, and research IT services and collaboration spaces.

Keywords: risk taking, shared services, library research spaces, translational research support, technology, collaborations, innovation, data walls

Monday, May 29, 2017, 10:30 AM – 11:55 AM
Room: 603

Session: Librarians as Innovative Collaborators: Doing What it Takes to Form New Partnerships

Moderator: Francesca Allegri

Implementing the Vision: Making Research Evaluation Services a Reality

Karen Gutzman

Impact and Evaluation Librarian, Galter Health Sciences Library, Chicago, Illinois

Michael E. Bales

Research Impact and Evaluation Informationist, Samuel J. Wood Library, New York, New York

Christopher W. Belter

Informationist, NIH Library, Silver Spring, Maryland

Liza Chan

Research Librarian, J. W. Scott Health Sciences Library, Edmonton, Alberta, Canada

Kristi L. Holmes

Library Director and Associate Professor of Preventive Medicine, Health and Biomedical Informatics Division, Galter Health Sciences Library, Chicago, Illinois

Ya-Ling Lu

Informationist, National Institutes of Health, Bethesda, Maryland

Lisa A. Palmer, AHIP

Institutional Repository Librarian, Lamar Soutter Library, Worcester, Massachusetts

Rebecca Reznik-Zellen

Head, Research and Scholarly Communication Services, Lamar Soutter Library, Worcester, Massachusetts

Cathy C. Sarli, AHIP

Senior Librarian, Becker Medical Library, St. Louis, Missouri

Amy M. Suiter

Scholarly Publishing Librarian, Bernard Becker Medical Library, Saint Louis, Missouri

Thane Chambers

Research Librarian, John W. Scott Health Sciences Library, Edmonton, Alberta, Canada

Terrie R. Wheeler

Director, Samuel J. Wood Library, New York, New York

Objectives: The increased demand for evaluation of research has led many libraries to consider providing evaluation support services. This paper discusses the different service models adopted by seven institutions, representing four medical libraries, one federal library, and one academic library and a funding support agency. Each model is described in detail with historical context, current service offerings, and future plans.

Methods: It can be very helpful to build a network of colleagues for support and discussion when implementing or augmenting services offered by the library. A group of seven libraries have consistently come together as colleagues to discuss advances in bibliometrics and new directions in research assessment. These seven institutions developed a narrative that describes their experiences building evaluation support services, reflects upon past evaluation-based projects, and discusses their future plans. This narrative serves as a trusted blueprint for any library considering these services and as a reliable companion for those already in the field.

Results: In implementing any new service, what works for one library may not for another. Some libraries have formal research evaluation services that exist as either a department or specialized group within the library, while others have adopted a decentralized approach where librarians provide services for their users on an as needed basis. Some libraries provide complimentary access to all of their services, while others have suggested providing a menu of options outlining complimentary and fee-based services. Seven institutions share their methods to demonstrate how libraries can offer these services and utilize only those aspects that make sense in their context.

Conclusion: The implementation of increasingly sophisticated literature databases, repositories, and research networking platforms means that libraries now have access to large quantities of digital data. This data is both qualitative and quantitative and include bibliographic data; gray literature; social media data; grant funding data, and new software applications that allow for visualization of data. Librarians can harness these data because of their expertise in capturing, describing, curating and visualizing data for a variety of purposes. Librarians can serve as neutral but active participants in research evaluation by advising on reliable measures, providing appropriate data, and promoting responsible use of metrics.

Keywords: research evaluation, research assessment, bibliometrics, library services, research support, research information systems

The Librarian’s Role in Attracting and Caring for Preceptors

Amy Chatfield

Information Services Librarian, University of Southern California, Los Angeles, California

Objectives: Many health sciences degree programs rely on preceptors to facilitate clinical learning for students. As more degree programs open in a geographic area, and accreditation requirements mandate additional services for preceptors, attracting and retaining qualified preceptors becomes more difficult. Education and support provided by the library and promoting subscribed resources can be one way to attract and maintain preceptors.

Methods: External pressures forced changes to preceptor services. New accreditation requirements came into effect for Pharm D programs; preceptors must now receive “facile access to current scientific literature.” The University of Southern California School of Pharmacy restructured experiential learning, leading to a review of preceptors and their skills. Preceptors desired support for their professional development and curricula to use in clinical scenarios with students. New schools opened in Southern California leading to competition for preceptors. The School partnered with the Norris Medical Library to create further enticements for preceptors in addition to library access, revamp handouts and websites created for preceptors, streamline the method for gaining access, and update library-provided instruction for preceptors. Preceptor use of the new website, volume and type of questions received from preceptors, and turnover of preceptors were used to assess the impact of these changes.

Results: 48 new preceptors were recruited, joining the 300 existing preceptors; no preceptors left the program. Hits on the revised preceptor LibGuide, clicks on links, and downloads of handouts increased slightly over prior year usage, but videos were not viewed at all. In prior years, the majority of preceptor questions focused on requesting and activating personal accounts to access library-licensed resources. The number of questions in late 2016/early 2017 remained the same, but fewer questions focused on access and more questions considered selection and use of resources. Preceptor library use traditionally spikes in spring; additional data will be provided at the conference.

Conclusion: Additional preceptors were recruited from the community, but it is difficult to connect this to the library’s efforts. Enhancement of library efforts did not significantly change preceptor behaviors with the library and its resources. Despite the lack of the desired impact, building a closer relationship between the library and the School of Pharmacy’s Office of Professional Experience Programs; learning about preceptor needs and how the library’s collection can fulfill these needs; and streamlining the process for preceptors obtaining library-licensed resources were valuable activities to undertake to support the increased number of preceptors.

Keywords: Pharmacy Preceptors Instruction Public Services Liaison partnerships

Horsing Around: Creating an Exhibit on Equine Anatomy from a Historical Veterinary Medicine Collection

Andrea C. Kepsel, AHIP

Health Sciences Educational Technology Librarian, MSU Libraries, East Lansing, Michigan

Objectives: The study of equine anatomy holds a prominent place in the history of veterinary medicine and was an ideal frame for an exhibit highlighting notable veterinary works at the Michigan State University Libraries. Developing the exhibit provided the perfect opportunity for the veterinary librarian to learn about the history of the field and led to collaborations within both the MSU Libraries and the College of Veterinary Medicine.

Methods: Taking advantage of a large historical veterinary medicine print collection, the librarian created an exhibit on equine anatomy to highlight its notable works. Creation of the exhibit involved several individuals from across the library, including special collections, conservation, graphic services, and

collaboration with the veterinary college and its faculty. Due to interest generated by the exhibit's content, after the library-based exhibit ended the material has been transformed into additional physical displays with artifacts at the college. A digital exhibit is also in process of being developed. In this presentation, the librarian will talk about the development and design of the exhibit, the opportunities for collaboration it led to in both the library and the veterinary college, and plans for future exhibits of the collection beyond the topic of equine anatomy.

Results: The equine anatomy exhibit successfully generated interest in works that are part of the MSU Libraries historical veterinary medicine collection. The skills and experience of many individuals in the library were necessary for the exhibit's success. Artifacts for the exhibit were generously loaned by the veterinary college and borrowing them provided an opportunity to connect with an alumnus of the College of Veterinary Medicine. Creation of a digital version of the exhibit has led to a greater conversation of how the MSU Libraries can use virtual exhibits to highlight other notable collections that are housed in special collections, and how best to design and organize a digital exhibit. The exhibit is used as an example of how subject specialist librarians can collaborate with special collections librarians, and the veterinary librarian is exploring other ways to strengthen and expand this relationship beyond exhibits.

Conclusion: Creating an exhibit on equine anatomy using items from the historical veterinary medicine collection at the MSU Libraries provided ample opportunities to connect and work with individuals within the library and beyond. The exhibit highlighted an underutilized collection and has led to larger conversations about how other subject specialist librarians can bring more attention to items in their own subject areas.

Keywords: veterinary medicine, special collections, history of medicine, exhibits, collaboration

Monday, May 29, 2017, 10:30 AM – 11:55 AM

Room: 607

Session: Systematic Reviews: From Start to Finish

Moderator: Susan A. Fowler

Building Capacity for a Systematic Review Core in an Academic Health Sciences Library

Mellanye Lackey

Associate Director for Education and Research, Eccles Health Sciences Library, Salt Lake City, Utah

Michelle Fiander

Associate Librarian, Systematic Reviews/Evidence Synthesis, Eccles Health Sciences Library, Salt Lake City, Utah

Mary McFarland

Information & Technology Consultant, Spencer S. Eccles Health Sciences Library, Salt Lake City, Utah

Melissa L. Rethlefsen, AHIP

Deputy Director / Associate Librarian, Spencer S. Eccles Health Sciences Library, Salt Lake City, Utah

Objectives: After an unsuccessful bid to fund a permanent full time librarian specializing in systematic reviews, the library partnered with the Population Health Foundation of the Center for Clinical and Translational Science (CCTS) and shifted to a model of charging groups for librarian's expertise to participate in systematic reviews.

Methods: Three librarians and one very experienced library staff member provide the expertise for systematic reviews. Two hours of free consultation include reviewing best practices for conducting a systematic review, confirming the group has adequate capacity to conduct a true systematic review, clarifying the scope of the project, educating about methodologies, and encouraging the group to register a protocol for the review. After the free, two hour consult, the librarian creates search strategies translated for relevant databases, download citations into EndNote, export a de-duplicated library, and write up the methods section for the final manuscript. Librarians are named as authors on final manuscripts.

Results: SR teams have been willing to pay for librarian expertise. A librarian was hired as an "Evidence Retrieval and Synthesis Librarian" to work full time on systematic reviews. The salary of the new librarian is paid for two years by the library, using a chargeback method to sustain the salary in the future. One limitation of the project is that groups may avoid asking the library for help, regardless of the free two hours, because they do not have funds to fully include a librarian. Quality of those reviews may suffer.

Conclusion: The chargeback model has been very successful, with multiple groups requesting librarian participation. Collaboration with the Population Health Foundation of the CCTS has led to new research partnership and increased advocacy for our services.

Keywords: Systematic review, capacity building, research, fee for service

Daring to Realize the Dream of Published Systematic Reviews

Allison M. Howard, AHIP

Research and Education Librarian, University of South Florida, Tampa, Florida

Nancy Schaefer, AHIP

Reference and Instruction Librarian, University of Florida Health Science Center Library, Gainesville, Florida

Objective: Systematic reviews (SRs) are consuming increasing amounts of librarians' time. This study sought to collect librarian experiences and opinions on unsubmitted SRs as well as suggestions for specific actions, policies, and procedures to assist in reducing their number. The goal is to increase the likelihood that librarian and researcher time and effort results in submission for publication.

Methods: An IRB-approved electronic survey was distributed to specific medical librarian email lists. Questions focused on perceived barriers to successful completion of SRs and efforts to eliminate or reduce these barriers. Checkboxes and a free-text box were provided for each question. A combination of Qualtrics and Excel was used to tabulate results. Follow-up phone interviews were conducted with some of those who volunteered their contact information.

Results: Most of the 128 participants who finished the online survey self-identified as academic librarians, with more non-tenure track than tenure-track. Only 7% of librarians reported systematic reviews as their primary responsibility. Respondents reported at least sometimes performing the following tasks for co-authorship: developing or editing search strategies (98%), searching grey literature (86%), snowballing/pearling (61%), requesting ILLs/scanning articles (60%), and hand-searching (52%). Fewer

indicated they at least sometimes assessed study quality (21%), extracted data (18%), or submitted the article for publication (13%).

The most noted barriers to submission included poor communication, unrealistic team size or timelines, too broad/narrow topics and inclusion/exclusion criteria, changing topics or personnel, and incomplete knowledge of or adherence to SR procedures. Participants most often implemented the following solutions: regular communication, checklists, formal instruction, limiting simultaneous SRs, and policies regarding fees, required protocols, and pre-consultation forms. Participants supported candidly discussing timelines, personnel involvement, and appropriate SR procedures at the initial meeting. SRs were declined due to faculty resistance to following SR protocols and workload commitments, although one person indicated declining wasn't an option.

Conclusion: With the majority of librarian tasks occurring early in the SR process, the initial meeting is the time to determine if a SR is the appropriate review type, and discuss expectations, time demands, and workload. The use of checklists and procedural policies could facilitate interaction with the PI. Administrative support for these activities and policies on concurrent SRs and permission to decline could protect librarians from over-commitment. In addition, SR fees might winnow requests, leaving more time for those with strong commitment and improve the rate at which work-effort results in submission.

Keywords: "Systematic reviews", submission, publication, barriers

Systematic Reviews at the University of California, San Francisco: An Evaluation of Methodology

Jill Barr-Walker

Clinical Librarian, ZSFG Library, University of California, San Francisco, San Francisco, California

Evans Whitaker

Research Librarian, UCSF Library, San Francisco, California

Julia Kochi

Director, Collections, Education and Research Services, UCSF Library, San Francisco, California

Peggy Tahir

Education & Copyright Librarian, UCSF Library, San Francisco, California

Min-Lin Fang

Research Librarian, UCSF Library, San Francisco, California

Objectives: UCSF Library has one year trial access to Embase with the express goal of providing a systematic review service to assist authors create this increasingly important study type. The aim of the current study is to analyze the last 10 years of systematic reviews written by UCSF authors for databases used and conformity with IOM and PRISMA recommendations.

Methods: A search of PubMed, Embase, and Web of Science for UCSF-authored systematic reviews between 1/1/2006 through 8/23/2016 found 2133 articles. Title/abstract screening eliminated 979 articles that did not meet inclusion criteria. Data was extracted from the remaining 1154 articles.

The following data will be extracted: number and names of databases searched, extent of librarian involvement in the review, and other methodological components identified in PRISMA and IOM guidelines. Descriptive statistics will be used to look for trends, and bivariate analyses may be used to examine relationships between methodological factors (e.g. librarian involvement) and other variables (e.g. departmental affiliation). Our conclusions will help inform instructional services, database purchase decisions, and targeted promotion of our systematic review services. Future investigation will include effect of these efforts on UCSF community conformity with Systematic Review best practices.

Results: Results will be shared at the presentation.

Conclusion: Conclusions will be shared at the presentation.

Keywords: systematic review, collection development, instruction

Advancing a Librarian's Role in a Rapid Review: Data Screening

Maylene (Kefeng) Qiu

Systematic Review Coordinator and Clinical Liaison Librarian, Biomedical Library, University of Pennsylvania, Philadelphia, Pennsylvania

Melanie E. Cedrone

Collections & Information Services Librarian, Biomedical Library, Philadelphia, Pennsylvania

Yong Chen

Yu-Lun Liu

Jonathan R. Treadwell

Objectives: To describe a pilot project where two health science librarians explored a new role by performing data screening after retrieving and managing literature search results in a rapid review project led by the clinicians in the Trauma Department of Presbyterian Hospital at the University of Pennsylvania.
Methods: Rapid review (RR) is an emerging methodology addressing the demands of policymakers, decision makers and clinicians who often require timely access to resources that succinctly and methodically synthesize evidence to inform their decisions. It may be driven by clinical urgency or may be determined by limited time and resources to conduct full SRs.

A one-clinician-centered team aimed to develop a program to improve patient care in the Trauma Department based on a RR. With limited time and staff resources, they strongly desired that librarians contribute as much as possible in generating the RR.

Considering the patrons' special need, the librarians advanced their traditional role of retrieving literature from databases in a systematic review project and performed the data screening for the clinicians. Abstrackr, a data screening tool, was employed in this pilot project.
Results: There were 1677 records related to the topic of mental health issues among gunshot wounded civilians retrieved from multiple databases searches. They were exported to EndNote for deduplication and 1049 records were exported to Abstrackr. In Abstrackr, a set of 500 records in a randomized order was set up for screening. The titles and abstracts of the records were screened. The prediction of relevancy or irrelevancy was provided to the unscreened 549 records. The results with p value were

downloaded for further examination. Measurement of efficiency and accuracy of Abstrackr was performed.
Conclusion: Librarians can advance our role in literature searching to literature screening in rapid review projects. Tools such as Abstrackr can be adopted to facilitate the process. The experiment revealed that Abstrackr enhanced the efficiency in saving time and reducing workload, and reached reasonable prediction.

Keywords: Data screening, data screen, literature screening, data screening tool, librarians' role, rapid review, systematic review, abstrackr,

Group Discussion Time

Monday, May 29, 2017, 1:00 PM – 2:25 PM

Room: 604

Session: Negotiating for Yourself and Your Library

Moderator: Ariel Deardorff

Negotiating for Yourself and Your Library

Kathel Dunn

Associate Fellowship Program Director, National Library of Medicine, Bethesda, Maryland

Kristi L. Holmes

Library Director and Associate Professor of Preventive Medicine, Health and Biomedical Informatics Division, Galter Health Sciences Library, Chicago, Illinois

Cynthia L. Henderson, AHIP

Executive Director, Howard University, Washington

Have you ever negotiated for a higher salary or a larger collections budget? Maybe you thought about negotiating for something but weren't sure how to do it effectively? This special content sessions is designed to give librarians practical negotiation skills. The session will begin with a half-hour interactive session on the basics of negotiation led by Kathel Dunn, Coordinator of the National Library of Medicine Associate Fellowship. This will be followed by talks from Kristi Holmes, Director of Galter Health Sciences Library and Cynthia Henderson, Associate Dean Health Sciences Libraries & Director Norris Library, who will share their experiences negotiating on behalf of themselves and their libraries. Finally, audience members will have a chance to ask questions and share their own experiences.

Monday, May 29, 2017, 1:00 PM – 2:25 PM
Room: 608

Session: Systematic Searching Live

Moderator: Margaret J. Foster, AHIP

Systematic Searching Live

Wichor M. Bramer

Biomedical Information Specialist, Medical Library, Rotterdam, Zuid-Holland, Netherlands

Susan A. Fowler

Library Director, Washington University in St. Louis, St. Louis, Missouri

Joy A. Russell, AHIP

Systematic Review and Grants Education Librarian, UT Southwestern Medical Center, Dallas, Texas

Objectives: Systematic searching is a task that many librarians aim to provide for clients. Building this skill requires practice, examples, and continuing education as new resources, tools, and concepts continue to evolve. While some guidelines are available, there is no consensus on the optimal method to develop search strategies. This session will provide an interactive live session developing, sharing and comparing searching techniques based on a few questions which will be publicized before the session as well as some provided by the participants. Audience will be encouraged to interact in a variety of methods- by developing search strategies for the questions provided before the session, and then during the session to provide examples of research topics they would like to see a search developed on, ask questions, and interact through discussions and online platforms during the session.

Keywords: systematic reviews, searching

Monday, May 29, 2017, 1:00 PM – 2:25 PM
Room: 615

Session: From Dreaming to Doing: Navigating the Research Pipeline

Moderator: Liz A. Amos

Kristina Elliott

Katherine Masterton

Patrick McLaughlin

Holly Thompson

From Dreaming to Doing: Navigating the Research Pipeline

Caitlin Bakker

Biomedical/Research Services Liaison, Bio-Med Library, Minneapolis, Minnesota

Sandra L. De Groote, AHIP

Scholarly Communication Librarian, University of Illinois at Chicago, Chicago, Illinois

Emily Hurst, AHIP

Head, Research and Education, Tompkins- McCaw Library for the Health Sciences, Richmond, Virginia

Stephen Kiyoi, AHIP

Library Director, UCSF at ZSFG, San Francisco, California

Medical librarians are now commonly expected to contribute to both the medical literature and the library profession through original research. The quality of research is dependent on a researcher's ability to both develop questions and methods and communicate their findings. This session will give attendees the tools and skills to develop their research from start to finish. Attendees will leave the session ready to identify research opportunities at their institutions, enabling them to take the "first step" into contributing to the field through original research. They will understand how to begin a research project and how to navigate barriers that medical librarians often face in conducting original research.

Panelists will address how to identify a research question, empowering librarians of all experience levels to improve the quality and frequency of their research output. In response to reported barriers to research activities, panelists will give examples on how to integrate research into regular work duties; apply for research funding; and find research mentors. A moderated discussion will facilitate an in-depth discussion of the topic. Time will be included for attendees to ask questions and share their own research challenges to encourage an ongoing dialogue about the medical librarian's role in research activities.

Keywords: Research, Original Research, Scholarly Communication, Early-Career Librarians, Mentorship

Monday, May 29, 2017, 1:00 PM – 2:25 PM

Room: 611

Session: Daring to Work on the Front Lines as a Clinical Librarian

Moderator: Jennifer A. Lyon, AHIP

Improving the Pediatric Clerkship

Emily Brennan

Research Informationist, Medical University of South Carolina Libraries / Research and Education Services, Charleston, South Carolina

Objectives: During their third year pediatric clerkship, medical students complete an evidence-based practice (EBP) project. The librarian and clerkship directors have improved the project by moving it earlier in the clerkship, as well as changing the content, length and methods of library instruction. Students have reported improved EBP abilities, and increased use of library resources.

Methods: Pediatric clerkship students work in teams to complete an evidence-based practice (EBP) project based on a patient they have seen on clinical rounds. At the beginning of the clerkship, the librarian teaches a review session in which students practice formulating a clinical question, searching the literature, and appraising evidence. Based on feedback, this review session is now longer and more interactive, includes hands-on critical appraisal, and is available virtually to off-site students. The librarian rounds with students on the inpatient wards to answer clinical questions at the point-of-care, consult on EBP projects, and demonstrate mobile apps. The students present their final EBP projects to the librarian, clerkship faculty, and their peers. The EBP project is now more structured, includes critical appraisal, and is presented halfway through the clerkship. The pediatric clerkship engages students in a clinically relevant evidence-based practice project.

Results:

Conclusion:

Keywords: pediatrics, clerkship, evidence-based, medical students, clinical rounds

Learning to Educate: A Resident Initiative to Develop Improved Patient Handouts in Pediatrics

Ruti M. Volk, AHIP

Lead, Patient Education and Health Literacy Program, Michigan Medicine, Ann Arbor, Michigan

Margeaux Naughton

General Pediatrics Clinical Instructor, Department of Pediatrics, Northville, Michigan

Priyanka Rau

Pediatric Chief Resident, Department of Pediatrics, Ann Arbor, Michigan

Stephanie Booms

Pediatrician, Department of Pediatrics, Indianapolis, Indiana

Heather Burrows

Program Director, Pediatric Residency Program, Department of Pediatrics, Ann Arbor, Michigan

Objectives: Pediatric faculty and residents were dissatisfied with the quality of patient instructions provided to patients through the EMR, and chose this as their required Quality Improvement /Patient Safety residency project. The goals were to create high-quality, patient-centered patient instructions handouts in plain language and to improve residents and faculty understanding of health literacy precautions and patient education principles

Methods: The librarian provided a training session to residents on health literacy and plain language writing and created an online training module with similar content for those who were not able to attend in person. After this basic training residents surveyed peers and compiled a list of high yield topics. Residents wrote the first draft of handouts utilizing a standard template that follows plain language guidelines. Faculty mentors reviewed handouts for clinical accuracy and the librarian provided a plain language review and gave feedback to writers. Handouts were uploaded to the Electronic Medical Record system. Residents created a screencast and a poster to educate their peers and faculty on utilizing the EMR system to send patient instructions handouts to print with the After Visit Summary. Utilization of the handouts was evaluated via a chart review and polling faculty and residents.

Results:

Residents created 19 waiting area posters and 24 patient instructions handouts over a 2 year period. Over 12 general pediatric residents and 6 general pediatric faculty have been involved in the creation of these materials. The materials were uploaded to the EMR system and also linked to the Patient Education Clearinghouse, a web-based database that includes all patient education materials that have been approved to be used in the health system.

Conclusion: Teaching residents to create and use the materials themselves helps residents to learn what constitutes readable and effective patient-education materials. It also teaches residents to take these created materials and put them into practice in their own clinics. This process engendered a collaborative effort between resident physicians, pediatric faculty, and a patient-education librarian. Once familiar with the resources available, such as the Patient Education Clearninghouse and librarian expertise, residents have been able to continue to create and use instructions and educational materials.

Keywords: Patient education, Patient Instructions, EPIC, plain language, health literacy, pediatrics, Electronic Medical Record, EMR

Applying Knowledge Management Best Practices to Capture, Support, and Archive Systems-Embedded Clinical Decision Support Evidence

Elizabeth Frakes

Information Scientist, Vanderbilt University Medical Center, Nashville, Tennessee

Zachary Fox

Information Scientist, Center for Knowledge Management, Nashville, Tennessee

Jing Su

Information Scientist, Center for Knowledge Management, Nashville, Tennessee

Mallory Blasingame

Information Scientist, Center for Knowledge Management, Nashville, Tennessee

Marcia Epelbaum

Senior Information Scientist, Center for Knowledge Management, Nashville, Tennessee

Spencer DesAutels

Information Scientist, Center for Knowledge Management, Nashville, Tennessee

Qinghua Kou

Health Systems Analyst Programmer III, Center for Knowledge Management, Nashville, Tennessee

Annette Williams

Senior Information Scientist, Center for Knowledge Management, Nashville, Tennessee

Nunzia B. Giuse, FMLA

Vice President for Knowledge Management / Professor, Biomedical Informatics, Center for Knowledge Management, Nashville, Tennessee

Objectives: To highlight the methods used by information scientists at a large academic medical center to successfully capture the processes around providing relevant and most current evidence to clinical decision support systems, and to explore the best practices for capturing and archiving changes in evidence over time and substantiating and documenting instances in which local practice decisions override published evidence.

Methods: Capitalizing on this team's history of clinical evidence provision, and prompted by the medical center's need to extract and organize clinical decision support (CDS) content from existing systems, leadership approached the team to lend its expertise to discovering/archiving evidentiary support for existing CDS. The urgency of this request comes in light of preparation for migration to a new clinical system. In conjunction with a project externalizing CDS rules in human-readable format, information scientists examine and evaluate all CDS content to discover existing embedded evidence, ascertain newer evidence or differing viewpoints, and document/preserve decisions driving local practice. The team will review literature on institutional experiences reconciling differences between local practice and published evidence. Preserving institutional knowledge, through documentation of situations in which published literature and local practice do not coincide, is a unique opportunity for information scientists to interject their expertise.

Results: The CKM team developed a robust, extensible knowledge acquisition and archival tool, CS-KAAT (Clinical Support Knowledge Acquisition and Archival Tool). As part of an integrated process, CKM Information Scientists analyzed and categorized over 8,000 CDS rules into evidentiary concepts, identified related evidence, and flagged for further review CDS rules which seemingly conflict with published evidence. To date, CKM has defined 755 evidence concepts supported by over 1600 citations scheduled for iterative review. As part of the process, 24 evidence flags were raised - 6 of which were escalated to clinician experts for resolution and the resulting decision is documented within CS-KAAT.

Conclusion: Decision support logic rules embedded in the physician order entry system, ordersets used to guide care for specific clinical conditions, and at-a-glance visual indicators of a patient's status within the EHR all rely on up-to-date evidence to improve patient care. As clinical information systems and

decision support rules continually evolve, the need to provide ongoing organizational and evidentiary support for the health information infrastructure is critical, thus presenting our profession with a significant, novel opportunity to integrate our skills and expertise within clinical and health information technology teams.

Keywords: Knowledge Management, Clinical Decision Support, Best-Practices, Archiving, Evidence

Library-Friendly Contract Language for Hospital Libraries

Kellee L. Selden

Manager - Learning & Knowledge Management, Education / Library Services, Milwaukee, Wisconsin

Michele L. Matucheski, AHIP

Medical LIbrarian, Ascension Wisconsin, Oshkosh, Wisconsin

Objectives: The purpose of this project was to work with our Contracts and Acquisitions Departments to develop a document/template that includes Library-friendly text that can be used to negotiate better Library eResource contracts and pricing for Hospitals and Health Systems.

Methods: Background : Although organizational acquisitions and legal departments may have templates and legal language appropriate for the entire organization, they don't have a good or complete idea of what Libraries require in a contract, nor how we use the contracted products and services. As a result, certain things important to Libraries may be overlooked or never even considered when Libraries are not directly involved with the contracting. Vendors will include language beneficial to them, but may not necessarily provide verbiage that will benefit and protect the Library with whom they are contracting. Methods : Developed a checklist of Library-Friendly language to include in contracts covering the following areas which may otherwise be overlooked by Acquisitions, Legal, or the Vendor. We pulled language from Lib-License and several of our own well-crafted consortia agreements.

Results: We shared this document with our Contracts Champion in Acquisitions at both the local and the national level, and our legal department to develop a template for better Hospital Library contracts.

Conclusion: When Librarians work with their corporate Purchasing and Legal departments on license agreements, we get better licenses with better terms, and pricing. Additionally, our corporate partners develop a better understanding of the contracting needs of Library Services, and can negotiate for us.

Keywords: contracting, license agreements, Purchasing Dept, Legal Dept, Acquisitions, e-resources

Monday, May 29, 2017, 1:00 PM – 2:25 PM

Room: 613

Session: Today's Dreamers, Tomorrow's Doers: New Voices

Moderator: Brenda Linares, AHIP

Information Highway: Navigating the Pathway to Medical Student Success: The Implementation of an Innovative Online Program to Assess Critical Information Skills

Melissa L. Mendelson

Director, Programs, Columbia University Medical Center, New York, New York

Amy J. Sommers

Program Coordinator, Columbia University Medical Center, New York, New York

Objectives: To determine whether an online intervention utilizing an instant feedback mechanism was successful in assessing first-year medical students' ability to effectively: 1) construct a research question; 2) conduct literature reviews; 3) recognize the e-Link icon to retrieve full text; 4) compare pros/cons of citation programs; 5) utilize informationist services; and whether these information skills are then applied in participants' internships.

Methods: The specific need for this targeted intervention was identified in Fall 2014 after piloting a competitive online program for first year medical students. Upon conducting post-program semi-structured interviews with these participants, it was determined that 1) a more optimal time during the academic year was needed to launch the online program and 2) the application of learned skills needed to be linked to the respondents' summer research internships. The second iteration of the program (Fall 2015) incorporated these changes. Next, an innovative feedback mechanism, instantaneously combining pre- and post-test surveys, was instituted to assess participants' information skills and increase program interactivity. After the participants' internships, a final post-test questionnaire was implemented to determine whether information skills were applied during the summer internships.

Results: The Knowledge Center's *Information Highway* program, launched in Fall 2015, was designed around the 5 learning objectives above, each with a dedicated round. The five rounds were conducted over the course of five consecutive days, one round per day. 52 first-year medical students at Columbia University's College of Physicians and Surgeons answered at least one question throughout and 29 participants played at least one full round. Seven participants completed all five rounds of the game. With the aim of participants achieving one objective daily, we found that 58% of all participants answered a question correctly on the first try. We also tracked the percentage of participants who correctly answered a question after receiving a hint via the instant feedback mechanism: 35% on day 1, 63% on day 2, 67% on day 3, 13% on day 4, and 100% on day 5, results varying by the nature of the question and participation level on a given day. In the 2015 post-test questionnaire, 100% of respondents felt prepared to apply information skills to their internship after completing *Information Highway*.

Conclusion: The instant feedback mechanism successfully assessed participants' information skills. The final post-test questionnaire revealed that Information Highway prepared students to apply information skills to their summer research internships. Future research with subsequent first-year cohorts will determine if findings may be replicated.

Keywords: INFORMATION SKILLS; MEDICAL STUDENTS; RESEARCH QUESTION; PROGRAM DESIGN; ONLINE INTERVENTION

Dream: Improving Health Literacy Dare: Enhancing Readability of Patient Education Material Do: One Document at a Time

Diana Almader-Douglas

Librarian, Hospital Library, Phoenix, Arizona

Objectives: Our institution's Patient/Family Education Subcommittee is tasked with revising and updating written material directed at patients and family members. A member of this subcommittee, a librarian, is participating in the editing process of patient education material by applying plain language and easy-to-read principles while increasing readability to improve health literacy of patients and family members.

Methods: The subcommittee receives internal requests for revisions and updates of patient education material from staff, departments, and specialty areas within our institution. The subcommittee revises terminology, repairs grammatical errors, and updates changes in contact information or room locations. To meet the objective of this project, the librarian selects and edits documents that appear to contain sophisticated language, medical jargon, or complex sentences or paragraphs. Before editing begins, original text is 'tested' and scored for readability and reading level by Readability-Score.com, a free online tool that assesses text for readability and grade level using multiple reading assessment formulas. Next, using plain language resources, wordlists, toolkits and guidelines, the librarian edits the document, aiming for a 6th grade reading level. After revision, text is re-scored by Readability-Score.com. Last, 'before and after' documents are compared to determine if readability or grade level changed.

Results: Four patient education documents were edited during this project. The average grade level of material before revisions was 10.35 and the average grade level of material after revisions was 9.15. On average, readability improved by a 1.2 grade level. In addition, revised material became more visually appealing due to the use of white space, larger font, headings and smaller words. Page counts increased after revision, however, this was inconsequential. During the project, subcommittee members were educated about the global movement of plain language and health literacy, and members adopted and implemented plain language and easy-to-read principles to the editing process. Recently, the subcommittee experienced administrative re-organization, resulting in changes to the review process of patient education material. To sustain the project, existing patient education material is being randomly selected for rating, and data is being collected with the purpose of proposing an enterprise-wide project with the Office of Patient Education.

Conclusion: This project inspired staff to adopt plain language practices and to become proactively involved in the effort of improving health literacy. Even small-scale changes have the power to improve readability of patient education material. Librarians should continue to contribute to plain language and health literacy awareness and practice.

Keywords: Health Literacy, plain language, consumer health information, patient education

References:

1. Federal Plain Language Guidelines. Available from: http://www.plainlanguage.gov/howto/guidelines/bigdoc/fullbigdoc.pdf.

2. Plainlanguage.gov [cited 2016 March 7]. Available from: http://www.plainlanguage.gov/.

3. How to Write Easy-to-Read Health Materials Bethesda, Maryland: National Library of Medicine; [cited 2016 March 8]. Available from: https://www.nlm.nih.gov/medlineplus/etr.html#plan.

4. Create a health literacy improvement plan toolkit. 2nd:[Available from: https://www.ahrq.gov/sites/default/files/wysiwyg/professionals/quality-patient-safety/quality-resources/tools/literacy-toolkit/healthlittoolkit2_tool2.pdf.

5. Assess select and create easy to understand materials toolkit. Available from: https://www.ahrq.gov/professionals/quality-patient-safety/quality-resources/tools/literacy-toolkit/healthlittoolkit2-tool11.html.

6. Quick Guide To Health Litearcy: Health Literacy Basics. What is Health Literacy? Available from: http://health.gov/communication/literacy/quickguide/factsbasic.htm#one.

7. Promoting Health Literacy Through Easy-to-Read Materials [3/14/16]. Available from: http://nnlm.gov/training/healthliteracy.

8. National Assessment in Adult Literacy (NAAL) Key Findings 2003 [2/9/17]. Available from: https://nces.ed.gov/naal/kf_demographics.asp.

9. The Health Literacy of America's Adults: Results From the 2003 National Assessment of Adult Literacy2006.

10. Plain Language Thesaurus for Health Communications: Centers for Disease Control and Prevention (CDC); 2007 [updated September 2007; cited 2016 March 8].

11. Busselman KM, Holcomb CA. Reading skill and comprehension of the dietary guidelines by WIC participants. Journal of the American Dietetic Association. 1994;94(6):622-5.

12. Lee SY, Bender DE, Ruiz RE, Cho YI. Development of an easy-to-use Spanish Health Literacy test. Health services research. 2006;41(4 Pt 1):1392-412.

13. Lee SY, Stucky BD, Lee JY, Rozier RG, Bender DE. Short Assessment of Health Literacy-Spanish and English: a comparable test of health literacy for Spanish and English speakers. Health services research. 2010;45(4):1105-20.

14. Medicine NNoNLo. Health Literacy [March 10 2016]. Available from: https://nnlm.gov/outreach/consumer/hlthlit.html.

15. Nielsen-Bohlman L, Institute of M, Committee on Health L. Health literacy : a prescription to end confusion. Washington, D.C.: National Academies Press; 2004.

16. Wilson FL, Mood DW, Risk J, Kershaw T. Evaluation of education materials using Orem's self-care deficit theory. Nursing science quarterly. 2003;16(1):68-76.

Librarian Involvement in an Introductory Clinical and Translational Research Course

Susan M. Harnett

Medical Information Services Librarian, University of Florida-Jacksonville, Jacksonvilel, Florida

Nancy Schaefer, AHIP

Reference and Instruction Librarian, University of Florida Health Science Center Library, Gainesville, Florida

Hannah F. Norton, AHIP

Interim Fackler Director, Associate University Librarian, University of Florida, Gainesville, Florida

Mary E. Edwards, AHIP

Reference & Liaison Librarian, Health Science Center Library, Gainesville, Florida

Michele R. Tennant, AHIP

Interim Fackler Director, Health Science Center Libraries, Gainesville, Florida

Objectives: To describe librarian involvement in a multi-disciplinary, intensive course designed to introduce junior faculty, PhD students, fellows and others interested in clinical and translational research to study design, data management and grantsmanship. The two week course consists of daily lectures

followed by an hour of small group work toward the presentation of a research proposal on an assigned topic.

Methods: Librarians at our library are involved with the course in several ways. New librarians are encouraged to take the course to enhance their understanding of the research process and its complex interactions with the university. Librarians also assist in developing the curriculum for the course and suggest themes for the small group presentations. In addition, they are embedded as guest lecturers on search methods, NCBI tools, and measuring and increasing research impact.

Each small group is advised by a team of faculty members which may include a clinician, an epidemiologist, a biostatistician or other researcher. The librarian assigned to each small group facilitates the discussion of the assigned topic and helps the group discover a manageable research question. She identifies relevant literature and data to support the proposal and provides input on the cohesiveness of the presentation.

Results: Since 2009, eleven librarians have participated in the course as students. Over time, our roles have shifted from class participants to facilitators of the small groups. The increased level of participation provided impetus to suggest themes for the small group work, which were related to HSCL funded projects. The course coordinator became more familiar with the breadth and depth of librarian knowledge of research methods and tools, increasing the lectures by librarians from 1 in 2003; to 4, scheduled for this coming summer.

Conclusion: The Introduction to Clinical and Translational Research series is designed to introduce the novice researcher to concepts and experts in study design, biostatistics, grantsmanship and information services. It also serves as a networking experience for those contemplating research projects at the University. Anecdotal evidence indicates that librarian presentations and contributions to small group work have increased student appreciation for the knowledge base of health science librarians and familiarity with research resources available to them through the process of the small-group project while librarians have gained valuable insight into types of information needed by their patrons to execute their research and grants.

Keywords:

Clinical research

Guest lectures

Instruction

Librarians

Research

Research proposals

Small group work

Translational research

Dreaming of Datasets: Outreach Strategies for an Institutional Data Catalog

Nicole Contaxis

Data Catalog Coordinator, NYU Health Sciences Library, New York, New York

Alisa Surkis

Head, Data Services and Translational Science Librarian, NYU School of Medicine, New York, New York

Fred LaPolla

Knowledge Management Librarian, NYU Health Sciences Library, New York, New York

Kevin B. Read

Knowledge Management Librarian, NYU Health Sciences Library, New York, New York

Objectives: A data catalog was created at an academic medical center to facilitate collaboration among researchers by helping them discover and share datasets relevant to their work. The catalog describes public and licensed datasets as well as datasets created internally by institutional researchers. Librarians sought to grow the number of records for internally generated datasets in the catalog.

Methods: The library hired a project coordinator with outreach experience to engage researchers and increase the number of catalog records for datasets created by institutional researchers. A variety of outreach strategies were employed to identify institutional researchers interested in sharing descriptions of their data as a means to increase the discoverability of their work. Strategies used include: (1) searching publications in journals that require data sharing; (2) searching grant databases for grants that require data sharing; (3) presenting at faculty meetings; (4) developing marketing materials; (5) researcher and liaison librarian referrals. Throughout the outreach process, data was collected on how each researcher was identified, why they chose to share, how they chose to share, where their data lives, where they published their research, their grant funding, data collection standards that they use, and referrals to other library data services including data management, REDCap, or data visualization.

Results: To date, consistent outreach has resulted in 24 consultations with researchers and staff. These consultations have produced 21 new, published dataset records with 23 other datasets currently in the process of being cataloged, edited, and published. Preliminary analysis of our outreach data demonstrates that researcher and liaison librarian referrals are the most successful outreach strategy, followed by contacting authors who had been identified through publications in journals that require data sharing. The most often reported reason for sharing data is a pre-existing dedication for sharing their research outputs, followed by a desire to locate collaborators in other disciplines and departments. The majority of datasets in the catalog generated by institutional researchers are NIH funded, and some are funded by the CDC and the Robert Wood Johnson Foundation.

Conclusion: Increasing the number of internally generated contributions to an institutional data catalog requires strong and consistent outreach that prioritizes building relationships with researchers and staff. The outreach process gives librarians an opportunity to engage in conversation with researchers, better understand their needs, and build new services based on researcher feedback.

Keywords: Outreach, Data Sharing, Data Curation, Collaboration, Discoverability, Marketing, Data Management

"Is There Life on Mars?" Gamification of Evidence-Based Practice (EBP) Learning at Macquarie University

Heather Cooper

Library Discipline Group Leader, Medicine, Health and Human Sciences, Macquarie University Library, Sydney, New South Wales, Australia

Grai Calvey

Library Discipline Group Leader, Medicine, Health and Human Sciences, Macquarie University Library, Sydney, New South Wales, Australia

Mary Simons

Clinical Librarian, Macquarie University Library, Sydney, New South Wales, Australia

Objectives: Use the principles of gamification (defined goals, scoring, feedback and choice) to embed EBP learning activities for undergraduate students enrolled in a new Bachelor of Clinical Science program in the Faculty of Medicine and Health Sciences. The interactive game, Mission to Mars, uses a fictional scenario to engage students in critical thinking strategies and skill development, in identifying different levels of evidence within an EBP program.

Methods: The project team, led by Librarians, included clinical, educational, gaming and information technology experts. A non-human disease was created as the scenario so students would have no prior knowledge of the illness. A pathogen that is transferred from bees to humans on the planet Mars formed an intriguing context. The game required students to navigate each level of an evidence pyramid by successfully completing tasks that tested their knowledge of clinical study designs before proceeding to the next level and eventually eradicating the disease. Tasks consisted of multiple choice questions, cloze tests and matching activities. The final version of the game was piloted with Library staff and university students, before being added to the EBP module of the clinical science Moodle platform. Each student undertook the game after completing all of the related EBP learning activities included in the module.

Results: Students posted to the online forum a reflection on the value of the game to their EBP knowledge and to their future careers. Reflective feedback indicated an appreciation of the game's role in reinforcing EBP principles in a stimulating, enjoyable format and promoting EBP learning within clinical and research contexts. Students began to understand how EBP principles could be applied to a clinical scenario to solve a problem. Recommendations for improvement of the game included increasing task difficulty and varying the nature of tasks. Librarians noted that technical issues which may have impeded the seamlessness of the game should be reviewed in the next version.

Conclusion: Mission to Mars actively engaged students in basic EBP concepts and furthered their understanding of their role in developing clinical and research skills. The use of gamification principles provided a new context for creating relevant, innovative ways to introduce EBP concepts to undergraduate students. The ability of the game to incorporate clinical applications of the learning activities increased students' appreciation of the value of EBP to clinicians.

Keywords: gamification, evidence-based practice, EBP, clinical science, moodle, medical education, undergraduates, higher education

Monday, May 29, 2017, 1:00 PM – 2:25 PM

Room: 612

Session: Bibliometrics in Action

Moderator: Shawn Steidinger, AHIP

A Series of Unconnected Events: A Library's Effort to Promote Research Education & Services

Lynn Kysh

Clinical & Research Librarian, Norris Medical Library, Los Angeles, California

Jennifer E. Dinalo

Information Services Librarian, Norris Medical Library, Los Angeles, California

Robert E. Johnson

Clinical and Research Librarian, Norris Medical Library, Los Angeles, California

Objectives

Share an institutional scholarly communication definition and efforts to address research knowledge gaps at an academic health science campus

Methods

The education and reference department focused its efforts on engaging the research community through a rebranding, restructuring, and expansion of education services. The department was renamed and librarian positions changed in order to dedicate more time to support researchers. A lunch time education series focused on technology shifted to include scholarly communication topics, and efforts were made to include data management education in the medical school curriculum. Systematic review services were expanded to include a department scoping review methodology and consultation services. Online tutorials on open access, choosing a journal, and identifying predatory journals were created and hosted on the library's webpage. The results of these efforts were identified and prioritized concepts within scholarly communication and increased service to researchers with positive feedback and potential to build an active librarian-researcher community.

Results This is a program description and does not have results as such.

Conclusions: This is a program description and does not have conclusions as such.

Keywords: scholarly communication, research, data management, systematic reviews, scoping reviews, open access, predatory journals

Daring to Dive Deep into the Citation Data: Going Head to Head with SciHub

Christy Jarvis, AHIP

Head of Information Resources and Digital Initiatives, University of Utah, Salt Lake City, Utah

Melissa L. Rethlefsen, AHIP

Deputy Director / Associate Librarian, Spencer S. Eccles Health Sciences Library, Salt Lake City, Utah

Objectives: To analyze the extent to which the library is fulfilling the information needs of its patrons in light of recent data showing heavy usage of Sci-Hub in [our metro city] and specifically to gain insight into the percentage of referenced resources and content areas that were not made available to researchers through library funded subscriptions.

Methods: Using Scopus, we identified papers published by the institution's health sciences faculty in each of the previous 5 years. Papers co-authored by researchers at other institutions were excluded in order to focus the analysis solely on information resources available to scholars at the target institution. The citations from the resulting set of publications were extracted to spreadsheets, where they were subjected to a data normalization process. Incomplete or obviously erroneous references were removed. The remaining set of citations was sorted alphabetically by journal title, then secondarily by citation year.

Using a combination of data sources, including publisher entitlement reports, catalog records, and previously downloaded holdings reports, we compared each citation to the library's collection at year of citation to determine if access to the cited resource was provided and paid for by the library.

Results: Published literature authored by University of Utah health sciences faculty between 2012 and 2016 yielded 119,794 citations for analysis. The libraries had print or electronic access to 99,298 (82.89%) of these cited resources. Another 9,220 (7.7%) were accessible from open access platforms, leaving only 11,084 (9.25%) citations that needed to be obtained from other sources, such as interlibrary loan, pay-per-view, or illegitimate sites such as Sci-Hub. Of the cited, but not provided, literature, 26% comes from backfile content whereas 74% comes from more recently published work. Identified collection gaps include the disciplines of Neurology, Cardiology, and Oncology.

Conclusion: A comprehensive evaluation of health sciences author citations over a 5-year period demonstrated that the vast majority were available from library-funded resources, thereby suggesting that the library's collection has been adequately meeting the needs of researchers and scholars. This study provides valuable insight into faculty information-seeking behaviors and has implications for future collection development, service offerings, and funding priorities within the library. Further study is needed to explore correlations between library-funded access to cited resources and other university metrics such as faulty recruitment and grant funding, as well as to investigate purported Sci-Hub activity in light of our findings.

Keywords: Citation analysis, scholarly publishing, library collections, Sci-Hub

HAMMERing out the Details: What Can an Online Bibliometrics Engine Tell Us about Research in Animal-Assisted Therapy?

Jane Yatcilla

Health & Life Sciences Information Specialist, Veterinary Medical Library, West Lafayette, Indiana

Objectives: The objectives of this study were (1) to field test HAMMER, a free web-based tool for performing statistical analyses on citation data. Is this tool accurate? Does this tool tell us what we really want to know? And, (2) to determine important aspects of the developing field of animal-assisted therapy (AAT) research, including important authors, journals, and topics.

Methods: Librarians are increasingly called upon to provide bibliometric or scientometric analyses for their institutions or disciplinary colleagues. To test a new bibliometrics tool, HAMMER, a data set of research articles citations and abstracts about AAT (the "corpus") was gathered from the Web of Science: Core Collection database. WOS: Core Collection was chosen because its records include fields that other databases do not, such as cited references from the articles, and information about funded research. Search terms included "animal assisted therapy/ies," "animal assisted psychotherapy," "equine assisted therapy," and "pet therapy." Document types were limited to research-oriented materials (articles, reviews, and proceedings papers), and were not limited by publication year or language. 455 citations constitute the corpus. The corpus was then uploaded into HAMMER to undergo its prescribed set of analyses, which include visualizations.

Results: Results included most productive authors, most cited authors, most popular publications, and most cited publications. CK Chandler was the author with the most articles but was not cited by other articles in the corpus, while SB Barker was most often cited and ranked third among top authors. While Anthrozoos was both the most popular journal and the most highly cited journal, there was a high degree of disparity between the most popular and the most often cited journals. A much higher degree of similarity was found between most popular keywords and most cited keywords. 25 "influential" articles were identified.

Conclusion: A free, automated tool like HAMMER can be useful for getting a quick snapshot of the landscape of a field of research. However, HAMMER has limitations. It can ingest records only from WOSCC, which may omit newer or rising journals, and HAMMER does not permit the user to perform any

data clean-up before analyses are run. "Popular journals" and "cited journals" do not represent the actual number of articles in each category, but are computed based on an influence formula. Some desired analyses may not be available. Comparison with other bibliometric software is warranted.

Keywords: animal-assisted therapy AAT human-animal bond human-animal interactions bibliometrics scientometrics HAMMER software NAILS project

Daring to Move Beyond the H-Index: Communicating Researcher Impact with the Relative Citation Ratio

Alisa Surkis

Head, Data Services and Translational Science Librarian, NYU School of Medicine, New York, New York

Kevin B. Read

Knowledge Management Librarian, NYU Health Sciences Library, New York, New York

Joey Nicholson

Education and Curriculum Librarian, NYU Health Sciences Library, New York, New York

Stuart Spore

Lead, Scholarly Output Assessment/Senior Systems Advisor, NYU Health Sciences Library, New York, New York

Objective: The relative citation ration (RCR) is a new field-normalized metric for citations per year that is benchmarked to R01 grant publications. The RCR presents an alternative to the commonly used h-index, which is dependent on a researcher’s field of study and career length. We sought to demonstrate the value of this metric within an academic medical center.
Methods: In response to requests from three academic units seeking bibliometric indicators with which to gauge the impact of their unit as a whole or of the faculty within their unit, reports were produced using the RCR. The units were presented with visualizations, a description of the advantages of this metric over other citation metrics, particularly the h-index, and information on its proposed use by the Clinical and Translational Science Awards Common Metrics efforts. Reports produced were as follows: 1) for basic science institute, mean RCR by year compared to department average; 2) for population health department, overall mean RCR and mean RCR by year compared to department faculty of the same rank; 3) for neuroscience institute, RCR of faculty within the institute compared to similar faculty outside institute.
Results: The use of the RCR for the three units was mixed. The basic science institute reported that they found the metric useful, the population health department found it useful but their interest was more in an RCR summary metric than the visualizations, and the neuroscience institute did not use the metric for their use case of telling positive stories for an institute event as a clear story did not emerge from the RCR analyses. The process was valuable in determining how best to use the RCR to reflect and clearly communicate research impact. Administrators from two of the three academic units, along with two of the authors of this abstract were subsequently named to a workgroup charged with redoing an institutional bibiliometrics dashboard. Based on the initial work, agreement to use the RCR was quickly reached, and the previous work was refined to arrive at a set of dashboard visualizations.

Conclusions: The RCR is a valuable new metric that overcomes a number of issues with metrics that have commonly been used to evaluate faculty and academic units. Introduction of this metric into departmental and institutional evaluation workflows met few barriers after clearly communicating both the important characteristics of the RCR and the importance of this metric to NIH.

Pattern of Coauthorship of Biomedical Researchers in the University of Ibadan

Toluwase V. Asubiaro

Librarian II, E. Latunde Odeku Medical Library, College of Medicine, University of Ibadan, Nigeria, London, Ontario, Canada

Objectives: The paper is a descriptive research which is aimed at investigating the pattern of co-authorship of biomedical researchers that are affiliated with University of Ibadan (UI) over a ten year period (2006 to 2015). Furthermore, to identify the degrees of collaboration and types of research collaboration of the UI researchers. Lastly, this article aims at investigating the geographical locations and collaborating researchers' institutions of affiliation with UI's biomedical researchers.

Methods: Data for this research work consisted of 1,942 articles/publications that were retrieved based on two criteria on PubMed database. First, the articles are those whose authors have affiliation with UI. Secondly, the time frame for publication dates is a ten-year period of 2006 to 2015. Variables such as number of authors, institutions of authors and countries of authors were collected and analyzed. The level of research collaboration or co-authorship was analysed as the number of co-authors per paper. The types of research collaboration were categorized as intra-institutional, national and international (African and global) collaboration. The analysis also included the degree of collaboration which was calculated based on collaboration coefficient.

Results: First, the number of papers written increased each year as the number of single-authored papers decreased. Most of the co-authored papers were written by 3 authors (25.10%), followed by 2 authors (22.69%) and four authors (17.90%). The Average number of authours per paper increased from 3.85 per paper in 2006 to 6.21 in 2015. 91.72% of published articles were written by more than one author, 48.67% of the collaboration was intra-institutional while 23.95% was international collaboration. The degree of collaboration also increased from 0.64 to 0.70 between 2006 and 2015. UI biomedical researchers collaborate mostly with researchers from the United States of America, followed by the United Kingdom, Switzerland, South Africa and India. The biomedical researchers collaborate with researchers that are affiliated with African countries mostly from South Africa, followed by Ghana, Uganda, and Kenya.

Conclusions: The trend in productivity and collaboration pattern changed significantly after 2013. These significant changes could best be attributed to the withdrawal of teaching services of the biomedical researchers due to a nationwide strike for a period of six months. This suggests that if the productivity and collaboration behaviour of the biomedical researchers can be enhanced if the Government can ease the teaching workload of the researchers by employing more academic staff in UI.

Keywords: co-authorship analysis, research collaboration, university of Ibadan, scientometrics

Monday, May 29, 2017, 1:00 PM – 2:25 PM

Room: 603

Session: Technology Dreamin': New Applications for Current and Emerging Technologies

Enhancing Accessibility of Rare Disease Literature for Researchers and Patients

Erin Foster

Data Services Librarian, Ruth Lilly Medical Library, Indianapolis, Indiana

Nicole Vasilevsky

Biocurator/Research Assistant Professor, OHSU Library/Ontology Development Group, Portland, Oregon

Mark Engelstad

Associate Professor/Pediatric Oral and Maxillofacial Surgeon, Oral & Maxillofacial Surgery Department, Portland, Oregon

Peter Robinson

Professor of Computational Biology, Genomic Medicine, Farmington, Connecticut

Sebastian Kohler

Research Associate, Institute of Medical Genetics and Human Genetics, Berlin, Berlin, Germany

Chris Mungall

Computer Research Scientist, Environmental Genomes and Systems Biology, Berkeley, California

Melissa Haendel

Associate Professor/Director of the Ontology Development Group, Oregon Health & Science University Library, Portland, Oregon

Objectives: In rare disease research, structured phenotype information is crucial to document in order to draw connections between other known cases and work towards diagnosis and treatment of disease. The Human Phenotype Ontology (HPO) is a standardized vocabulary that describes phenotypic abnormalities encountered in human diseases. To enable the increased identification of traits (i.e., phenotypes) associated with rare diseases, the HPO was expanded to include layperson synonyms to make the ontology more accessible and useful to patients. Additionally, the HPO was used to annotate phenotypes in a sample of rare disease case reports to provide structured annotations of rare disease phenotypes.

Methods: The HPO was systematically reviewed and 'layperson synonyms' were added to include terms used by patients and non-medical professionals. Subsequent work annotated phenotypic descriptions in a sample of rare disease case reports with HPO terms. The literature sample was identified by filtering 'case reports' in PubMed and excluding articles that were already included in the Online Mendelian Inheritance of Man (OMIM) database. The sample set was further restricted to articles from the European Journal of Human Genetics for the pilot set, which resulted in a final sample size of 143 articles. The papers were reviewed and annotated for the following information: disease name, associated gene(s), and corresponding phenotypes.

Results: The review of the HPO resulted in approximately half of the terms including layperson synonyms. A subset of the literature sample was annotated to determine the best curation workflow. Of

that subset of papers (n=20), 353 total phenotypes were identified. 12% of these phenotypes were not included in the HPO and required new term and/or synonym requests. Some challenges encountered in this work included maintaining consistency in HPO term definitions and use, as well as annotation reliability.

Conclusion: This work contributes to knowledge of rare diseases by curating the existing literature to provide structured annotation of rare disease traits, which helps with information retrieval and data interoperability and reuse. Additionally, the expansion of the HPO to include layperson synonyms enables patients to 'self-phenotype' and contribute to the identification of rare disease traits. Following the completed annotation of the literature sample, future work will focus on incorporating the annotations into databases that collect rare disease phenotypic information. Further work is also being done to add additional layperson synonyms to the HPO through review of patient forums and medical message boards to continue to identify terminology used by actual patients.

Keywords: Rare disease, Ontologies, Phenotypes, Interoperability, Citizen science

Imagination, Adventure, and Practical Application: Virtual Reality in a Health Sciences Library

Kimberley Barker

Librarian for Digital Life, Claude Moore Health Sciences Library, Charlottesville, Virginia

Bart Ragon

Associate Director, Knowledge Integration, Research, and Technology, Health Sciences Library, Charlottesville, Virginia

Stephanie Fielding

Technology in Education Consulting Specialist, Claude Moore Health Sciences Library, Charlottesville, Virginia

Objectives: To determine if the implementation of virtual reality as a service (“service” is defined as workshops, classes, consultations, and demonstrations) in the Health Sciences Library can successfully support the work of faculty and clinicians, and the education of students. To measure patron usage of the virtual reality service, and to document ways in which it is being used. To determine if the Virtual Reality service adds value to the educational mission of the Library and Health System. To determine whether or not the Virtual Reality service expands the Library’s partnerships, and leads to interdisciplinary collaboration.

Methods: The site for this study is the University of Virginia Health System, and included faculty, staff, and students interested in the application of Virtual Reality technology for research and the delivery of patient care. An intellectual goal of this study is to explore this emerging technology through the perspectives of library workshop participants and through library partners. A mixed-methods approach was utilized to explore the phenomena of the use of Virtual Reality at an academic medical center, and potential support roles for librarians. Data will be triangulated from interviews and workshop evaluations to best understand the meaning of the data collected, and inform how biomedical libraries can create new service roles in supporting virtual reality technology. Broader implications may help to provide insight to libraries on how support of emerging technology can be perceived as meaningful to the organizations they serve.

Results and Conclusion: In spring 2017 the library will conduct a series of workshops and other public events introducing virtual reality technology to UVA health professionals. Interactions will be used to create a dialog with participants exploring the application of Virtual Reality technology in a health care environment. Survey data will be administered to workshop participants assessing the perceptions of participants on the medical education, pain management, exposure therapy, patient education, and medical simulation. Results will be triangulated and shared at the annual meeting.

Keywords: Virtual Reality, Emerging Technology, Game Changer, Future, This is really cool stuff

Embedded Librarians Integrating 3D Printing Technology into Master's Level Health Sciences Courses

Jamie Saragossi

Health Sciences Collection Development and Outreach Librarian, Stony Brook University Libraries, Stony Brook, New York

Objectives: The objectives of this project were to educate Occupational Therapy and Physical Therapy students and faculty on 3D printing in healthcare and provide them with hands-on experience using this new technology while increasing the collaboration between librarians and faculty in the Touro College School of Health Sciences.

Methods: A survey was administered to students prior to the start of the project. Questions captured students' attitudes towards 3D printing, their practical knowledge of 3D printing in the health sciences and their interest in a hands-on experiential learning project within their course curriculum. Questions were answered on a Likert scale allowing for comments. After the assignment completion, the same survey was again administered to students. The results were compared to assess if knowledge and comfort levels with the new technology, their attitude toward 3D printing and their willingness to use this in their future practice had changed. Qualitative data was also collected through observations and interviews to obtain suggestions for future implications.

Results: The survey results illustrated an increase in student's understanding and familiarity with 3D printing upon course completion. However, there were not large deviations in the rest of the responses from pre to post-test. Qualitative interviews conducted with key faculty, determined that having more control over file creation was necessary for success of project moving forward however the overall impression of the project and partnership with the library was positive. This project met its goal by increasing the visibility of the library and providing a platform for librarian collaboration within the curriculum.

Conclusion: The students enrolled in the two pilot courses received exposure to 3D printing in Rehabilitation Science. The OT students who had the most time with the printer were able to identify a patient need and create a device that they were able to see in practice. They were proud of their finished products and grateful for the opportunity to have a hands on experience with 3D printing to assist their patients. The pilot was deemed successful and will be rolled out on a larger scale and formally adopted into Occupational Therapy curriculum going forward.

"This project has been funded in whole or in part with the Federal funds from the Department of Health and Human Services, National Institutes of Health, National Library of Medicine, under contract no. HHSN-276-2011-00003-C, with the University of Pittsburgh"

Keywords: 3D Printing, Embedded Librarians, Experiential Learning, Allied Health Education

Gamification of Student Learning Assessment in an Online Graduate-Level Health Informatics Course

Stephanie H. Kinsler

Assistant Professor, Research & Learning Librarian, University of Tennessee Health Sciences Library, Memphis, Tennessee

Zeb Mathews

Assistant Professor/Librarian, University of Tennessee Health Sciences Library, Memphis, Tennessee

Sajeesh Kumar

Associate Professor, Health Informatics and Information Management, Memphis, Tennessee

Objectives: To use a gamified learning experience (video game) to measure graduate health informatics students' understanding of primary and secondary sources after online, video instruction and Blackboard discussion board activities on how to identify primary and secondary sources.

Methods: Research and Learning librarians collaborated with an informatics professor to create a lesson for graduate level health informatics students enrolled in an online *Concepts of Research Methodology* course. The research team focused on a single learning objective for this course, identifying primary and secondary sources. Students completed a pre-test designed to establish a baseline score. Students viewed a video lecture on Primary and Secondary sources and completed bi-weekly discussion board exercises to reinforce the video content. A video game, which was created using Unity software, was utilized to assess students' learning after the completion of the primary/secondary lesson modules. After verifying that they completed the game, students were given a qualitative survey on their game experience as well as a primary/secondary source post-test.

Results: Students who completed both the pre- and post-test improved their scores by several points. All students were able to complete the required game modules within a short timeframe. Students expressed an overall positive learning experience with the learning modules created by the embedded librarians, and the majority felt that the gamification of the primary and secondary course content assisted with their learning process.

Conclusion: The gamification of the online learning module was well received by our mostly-millennial learners. The authors conclude that this particular video game might be more effective as a learning reinforcement tool rather than a standalone assessment. The competitive nature of the game environment, which requires students to beat the clock and earn a score, shows great promise in enhancing the medical student learning experience.

Keywords: gamification, collaboration, instruction, student engagement, assessment, technology

iPad Innovations During Clinical Rotations from Seven Medical Schools in the United States: Lessons Learned

Julie K. Gaines

Associate Professor; Head of the Medical Partnership Library, Augusta Univ/Univ of Georgia Medical Partnership, Athens, Georgia

Kalie Deutsch

OB/GYN Resident, Emory University School of Medicine, Atlanta, Georgia

Janette R. Hill

Professor of Education, UGA College of Education, Athens, Georgia

Michelle A. Nuss

Campus Dean; Professor of Medicine and Psychiatry, Augusta Univ/Univ of Georgia Medical Partnership, Athens, Georgia

Objectives: To identify the uses and limitations of mobile technology in the clinical undergraduate medical education curriculum, and guide medical schools in implementing iPad programs into their curriculum.

Methods: Since 2010, many US medical schools have introduced the use of mobile technology into their curriculum. Preclinical use of mobile technologies has been well studied, but use in the clinical years has been less explored. Semistructured telephone interviews were conducted with key personnel at seven US medical schools selected as having established mobile iPad technology, programs, and published content indicating iPad implementation in the clinical years. The key personnel interviewed included a Library Director, a Dean of Curriculum, and an Associate Dean of a medical school. Purposeful sampling was used to select medical school participants based on a comprehensive literature review. The strategy for analyzing the data consisted of transcribing the interview dialog, reading and reviewing this material to identify general themes, and then categorizing the data using a constant comparative method.

Results: Analysis of the interviews identified seven major themes: (1) goals, (2) teaching and learning, (3) EHR access, (4) culture, (5) benefits, (6) barriers, and (7) suggestions for implementation. Using these major themes, eight ‘‘best practices’’ for introducing mobile technology in the clinical years were identified: (1) plan before implementation, (2) define focused goals, (3) establish a tablet ‘‘culture,’’ (4) recruit appropriate implementation team, (5) invest in training, (6) involve students in mentoring, (7) accept variable use, and (8) encourage innovation.

Conclusion: There is growing interest in using mobile technology for teaching and learning in the clinical curriculum. Following the identified best practices may assist schools with integrating technology into the curriculum and better prepare medical students to manage increasing clinical use of technology.

Keywords: iPads; medical school; medical school curriculum; mobile technology; technology integration; clinical rotations; tablets

Monday, May 29, 2017, 1:00 PM – 2:25 PM

Room: 607

Session: The Dream Realized: Instruction In and Beyond the Classroom Walls

Moderator: Misa Mi, AHIP

Defining Evidence-Based Practice across the Disciplines: First Steps for Interprofessional Education

Megan von Isenburg, AHIP

Associate Director, Research and Education, Medical Center Library & Archives, Durham, North Carolina

Jamie Conklin

Research & Education Librarian, Duke Medical Center Library, Durham, North Carolina

Leila Ledbetter

Librarian, Duke University Medical Center Library, Durham, North Carolina

Brandi Tuttle, AHIP

Research & Education Librarian, Duke University, Durham, North Carolina

Megan G. Van Noord

Research & Education Librarian, Duke University Medical Center Library & Archives, Durham, North Carolina

Objectives: Increased interest and emphasis on interprofessional education (IPE) opens the possibility for librarians who currently teach evidence-based practice (EBP) to develop an IPE EBP curriculum, creating a blended classroom of students from across health profession disciplines. This study aims to identify how EBP differs among the health professions in terms of context, processes, types of questions, and definitions.

Methods: Before designing an interprofessional curriculum in evidence-based practice, we want to understand what differences exist in how health professions conceptualize and teach EBP.

With a focus on medicine, nursing, physician assistant, and physical therapy programs, we will conduct a thematic content analysis through identification and analysis of curricular materials, including health professions educational standards, sample cases within EBP textbooks, and EBP course syllabi. We will analyze the contents for themes. Specifically, we will code categories related to question types and focus areas where we expect to find some differences that will inform the feasibility of and, if possible, the curriculum development for instructing interprofessional groups of students.

Results: Evidence-based practice content is mandated by national curricular standards for nursing, medical, physician assistant, and physical therapy students. Some health professions disciplines are more closely aligned in evidence-based practice process and context than others. For example, medicine and physician assistant programs teach EBP skills similarly. Nursing, however, teaches EBP in a broader context that includes quality improvement, research, and patient safety. Physical therapy includes a strong emphasis on patient preferences and values in all steps of EBP.

Conclusion: The challenges in delivering a single evidence-based practice course to an interprofessional group of health professions students exceed logistical concerns for timing and space. Additional considerations must be given to the variable emphasis within each program, discipline-specific steps in EBP processes, types of questions, and the relationship of EBP to quality improvement and research.

Keywords: interprofessional education, evidence-based practice, qualitative research

Experience of Implementing an Embedded Librarian Program

Annie C. Nickum, AHIP

Research and Education Librarian, Library Resources of the School of Medicine and Health Sciences, Grand Forks, North Dakota

Dawn E. Hackman, AHIP

Research & Education Librarian, Health Sciences Library, Grand Forks, North Dakota

Marcia J. Francis, AHIP

Southwest Clinical Campus Librarian, Health Sciences Library, Bismarck, North Dakota

Amber Amidon

Northwest Clinical Campus Librarian, Health Sciences Library, Minot, North Dakota

Kelly Thormodson

Interim Director, Health Sciences Library, Grand Forks, North Dakota

Erika Fisher

Southeast Clinical Campus Librarian, Harley E. French Library of the Health Sciences, Fargo, North Dakota

Objectives: This paper examines the efforts of one small academic health sciences library to create a role for its embedded librarians within a new physical environment and a new pedagogical initiative. This paper will discuss the first year of activity in the new building.

Methods: In summer 2016, the library moved into a new health sciences building, the design reflecting cultural and pedagogical shifts within the school. Changes to librarian duties and involvement have grown exponentially. Participation in curriculum committees and task forces allowed the librarians to meaningfully network with faculty and staff. Embedded librarians found faculty were open to collaboration. Librarians began initiating changes to respective curriculums to include active learning. For the first time, librarians trained and began facilitating problem-based learning (PBL) groups during the fall semester. New opportunities have included more consultations, collaboration on grants, and invitations to present. Challenges facing the librarians include communication among librarians and library staff in the new environment and the need to find balance for ourselves in lieu of the success and increased requests for our time.

Results: Liaison librarians have been embedded in suites with faculty and administration related to educational and research programs. This increased exposure to faculty led to librarians assisting with NIH compliance, increased requests for mediated searches and opportunities for collaboration in active learning teaching, and research projects. Those who participated as facilitators in the medical curriculum report that it took longer than expected to prepare, facilitate, and then evaluate their students. The reformatting of several library sessions to incorporate active learning have been well received by students and learning objectives seem to be retained from one session to the next. Technology including webcams and telephone headsets were purchased to allow embedded librarians to fully participate in online meetings. This has increased collaboration and shared duties of liaison librarians.

Conclusions: A great deal of time and effort was spent preparing for the new building and the embedded role of the librarians. Despite what seemed to be thorough planning, there were challenges to overcome including technology, communication, and budgetary issues. Librarians have also enjoyed several successes. PCL facilitation took more time than expected, but was felt to be a rewarding experience. Overall, the librarians are pleased with increased opportunities to work closely with faculty and students.

Keywords: embedded librarian, active learning, facilitation

The Data Visualization Clinic: A Library-Led Workshop on Data Visualization

Fred LaPolla

Knowledge Management Librarian, NYU Health Sciences Library, New York, New York

Denis Rubin

Senior Quantitative Analysis Specialist, NYU Data Services, New York, New York

Objective: Many researchers have difficulty designing effective data visualizations and connecting with others involved in visualization. The library leveraged its role as a campus hub to connect researchers for two purposes: a) to aid in designing data visualizations and b) to foster a community of practice for researchers with visualization challenges.

Methods: After attending several “Data Viz Clinic” events piloted at our medical center’s affiliated, main-campus (non-medical) library, we adapted the program for our patrons: medical center faculty, staff and students. Email blasts, announcements during library presentations, and liaison outreach were used to market across the medical center. The recurring event was held in a library classroom, and a number of participants presented their visualization projects. A data visualization librarian managed logistics, led the session and facilitated discussion, which included peer and expert feedback. Presenters were allotted 10-15 minutes for presenting their work and discussion, which consisted of audience members asking questions and providing suggestions. Evaluation forms collected data on participants’ experience.

Results: Three sessions were held since fall 2016, with 48 individuals attending and 85 online enrollments. Clinic participants represented a range of departments and roles at the med center, including clinicians, basic science researchers, students and project managers. Ninety-four percent of participants said they either would highly recommend or recommend the session, and ⅔ of the total participants rated the experience at the highest level in our evaluation forms. Of 85 class signups, 14 individuals were repeat enrollments, meaning 16% felt sufficiently motivated to sign up again. Additionally, the events fostered discussion among diverse library patrons, leading to an environment of open communication for attendees and allowing them to network in the context of data visualization.

Conclusions: The Data Visualization Clinic is a low-cost, low-barrier-to-entry way to leverage library space for providing data visualization services. The event relies on participation from attendees to present projects and offer insights, allowing the librarian to serve primarily as a facilitator. As such the event brings in expertise from the clinic’s participants and allows them to share their knowledge and foster a community of practice. The Data Visualization Clinic helps promote library services both in data visualization and as an institutional resource, while allowing participants to shape content and form connections.

Keywords: data visualization, data services, data lifecycle, data analysis, scholarly communications, workshops, technology, innovation, community of practice, library as hub

Establishing a Peer Review Process to Evaluate Research Guides

Heather L. Brown, AHIP

Head, Collection Services, McGoogan Library of Medicine, Omaha, Nebraska

Emily J. Glenn, AHIP

Education & Research Services Librarian, McGoogan Library of Medicine, Omaha, Nebraska

Christian I.J Minter

Assistant Professor/Education & Research Services Librarian, University of Nebraska Medical Center, McGoogan Library of Medicine, Omaha, Nebraska

Danielle Drummond

Education & Research Services Associate, University of Nebraska Medical Center, McGoogan Library of Medicine, Omaha, Nebraska

Larissa Krayer

Digital Archivist, University of Nebraska Medical Center, McGoogan Library of Medicine, Omaha, Nebraska

Alison Bobal

Head of Collection Development and Metadata, University of Nebraska Medical Center, McGoogan Library of Medicine, Omaha, Nebraska

Objectives: Springshare's LibGuide platform has become synonymous with online library pathfinders and instruction support. However, without oversight of the collection of guides and standards for their creation, unwieldy numbers and lack of uniformity prevail. This paper will outline the creation of standards and application of systematic peer review to increase the value and relevance of research guides.

Methods: Prior to migration to the newest version of LibGuides, existing research guides were culled. Following migration, the library's Web Committee took the charge of developing standards and a peer review process for creating new guides, improving previously existing guides, and removing guides with low usage.

Standards and a checklist for creators were created for standalone and course-embedded guides, based on practices at peer institutions. Guide idea proposals were submitted through an online form. If proposals were accepted, the creator would then submit a private URL to the committee for review of the guide and application of the standards. Usage of previously created guides was analyzed through the LibGuide statistics module and Google Analytics. Recommendations for improvement or retention were made as a part of a larger improvement of online presence and shift to the use of durable learning objects.

Results: This project is in progress. Results will be presented at MLA.

Conclusion: This project is in progress. Results will be presented at MLA.

Keywords: Peer review

Process improvement
Audience
Planning
Workflow
Education
Standards

Association of Academic Health Sciences Libraries Task Force Evaluating Association of American Medical Colleges Core Entrustable Professional Activities

Heather Collins, AHIP

Assistant Director of Research and Learning, A.R. Dykes Health Sciences Library, Kansas City, Kansas

Nancy E. Adams

Associate Director, Coordinator of Education & Instruction, Harrell Health Sciences Library: Research and Learning Commons, Hershey, Pennsylvania

Emily Brennan

Research Informationist, Medical University of South Carolina Libraries / Research and Education Services, Charleston, South Carolina

Iris Kovar-Gough, AHIP

Liaison Librarian to the College of Human Medicine, Main Library, Michigan State University, East Lansing, Michigan

Elizabeth R. Lorbeer, AHIP

Chair, Department of the Medical Library, Western Michigan University School of Medicine, Kalamazoo, Michigan

Joey Nicholson

Education and Curriculum Librarian, NYU Health Sciences Library, New York, New York

Rikke S. Ogawa, AHIP

Team Leader for Research, Instruction, and Collection Services, Louise M. Darling Biomedical Library, Los Angeles, California

Neil Rambo

Director, Health Sciences Library, New York, New York

Judy M. Spak

Curriculum Services Librarian, Cushing/Whitney Medical Library, New Haven, Connecticut

Kelly Thormodson

Interim Director, Health Sciences Library, Grand Forks, North Dakota

Megan von Isenburg, AHIP

Associate Director, Research and Education, Medical Center Library & Archives, Durham, North Carolina

Objectives: To describe the Association of Academic Health Sciences Libraries (AAHSL) task force investigating the Association of American Medical Colleges (AAMC) Core Entrustable Professional Activities (EPAs) for Entering Residency.

Methods: In May 2014, the Association of American Medical Colleges (AAMC) published the Core EPAs for Entering Residency. In response to this change, in the Spring of 2016, AAHSL formed a task force to evaluate how the new Core EPAs will affect the medical school and thus the engagement of librarians in that curriculum. The task force is charged with identifying libraries participating in Core EPA activities, developing a methodology to characterize the nature of participation, and mapping and cross-referencing existing competencies, but specifically focusing on EPA 7 (application of evidence to advance patient care) and EPA 13 (culture of safety and improvement). This paper will report on the results of the Fall 2016 national survey of AAHSL member libraries' involvement with integrating and assessing Core EPAs into the medical curriculum.

Results:

Conclusion:

Keywords: medical curriculum, Core Entrustable Professional Activities, Core EPAs, evidence-based practice, AAHSL, AAMC

Monday, May 29, 2017, 5:00 PM – 6:25 PM

Room: 607

Session: Tech Trends

Moderator: Mary E. Edwards, AHIP

Mobile Librarian: iThink Tech Savvy (Meeting Students Where They Are)

Jahala Simuel

Medical Librarian, Louis Stokes Health Sciences Library, Washington, District of Columbia

Objectives: The rapid advancements in technology can create a bridge in connecting with student learners at health sciences libraries. It is imperative for the library to be the learning connection for students. As librarians we are required to think innovatively and creatively to bridge the gap between the deliverance of information to the current technology commonly being used by learners. What better way

to connect with learners but through the phones, tablets, and gadgets used in their everyday life? Technology provides the chance for librarians to innovate, boost quality, measure success, and align services with the priorities of their institutions. With technology, librarians can reintroduce themselves as visible, valuable, and essential partners in achieving common goals. The younger generation of technologically capable medical professionals in training, such as students and residents, harness the power of innovative apps to improve learning. The mobility of a smartphone, or tablet allows students to carry a plethora of clinical resources in a convenient and searchable package. This poster will outline various mobile apps that will encourage the flexibility of the mobile platform and provides interactivity in teaching and a more personalized education.

Methods: The process of Health Sciences Librarian being mobile and going to where the students are and surveying students to identify the use of mobile devices for overall academic activities, including need for access to library resources and services. In higher education many leaders are embracing new teaching practice that involves making the students the center of their learning. Learner-centered environments incorporates various teaching practices and uses techniques to improve students overall experiences that allows them to have more active roles in their academic learning. Many students have shown that this new pedagogy is effective and successful. It is imperative for health sciences libraries to embrace this new paradigm and begin using mobile apps to support their programs and services.

Keywords: technology, mobile apps, teaching, students, mobile, learning, education, librarians, outreach,

Please Follow Us: Tools and Tips for Getting the Most Out of Social Media

Rachel Helbing, AHIP

Health Sciences Librarian, University of Houston Libraries, Houston, Texas

Nha Huynh

Education and Instruction Librarian, Client Relationship Management, Houston, Texas

Social media efforts can be more successful when they move beyond broadcasting information about the library to a passive audience, to fostering interaction between the library, its users, and colleague institutions. This can be done in an efficient way that optimizes staff time and collects meaningful analytics. We will demo some online tools and improved workflows to help you get the most out of your library's social media accounts.

Keywords: Social Media, Engagement, Policies, Analytics, Facebook, Twitter, Instagram

Finding a Niche: Where 3D Printing Services Can Have the Most Impact

Jennifer Herron

Emerging Technologies Librarian, Ruth Lilly Medical Library, Indianapolis, Indiana

Objectives: Identify and promote a “niche” for 3D printing in a medical school community.

Methods: Reviewed 3D print requests filled by library and identified trends with requests. Broke requests down by level of student and proposed purpose of print. These trends helped to better understand the needs for 3D printing and has provided an outlet where the library can develop and enhance it's presence in student medical education.

Keywords: 3D Printing, medical students, medical school curriculum;

Tuesday, May 30, 2017, 3:00 PM – 4:25 PM

Room: 603

Session: Environmental Health: Addressing Health Disparities at the Source

Moderator: Kate Flewelling

Environmental Health: Addressing Health Disparities at the Source

Kate Flewelling

Executive Director, National Network of Libraries of Medicine, Middle Atlantic Region, Pittsburgh, Pennsylvania

James Anderson

Physician Assistant, Evergreen Treatment Services, Seattle, Washington

Linda Hasman

CTSI Liaison Librarian, University of Rochester Medical Center, Rochester, New York

Laura Bartlett

Technical Information Specialist, National Library of Medicine, National Institutes of Health, Bethesda, Maryland

Objectives: Illness is clearly impacted by the social and environmental forces which surround patients in their communities. A review of the literature finds that zip codes can determine one's health as much as their genetic code. Some of these factors that play key roles in health inequities are increased presence of environmental toxins, as well as location-related poverty, racism, violence, and lack of access to nutrition, medical care, education and recreation. Despite improvements in access to care, health disparities still exist for racial and ethnic minorities. This session will consist of four interconnected presentations. A health professional will begin the session with an introduction to Environmental and Health Equity. Then, we will use the issue of lead in urban neighborhoods as a case study to examine health effects, injustice, and the importance of high quality information. We will use relevant information resources to find the evidence and support environmental health and social justice initiatives. We will end the session talking about avenues for outreach, including relevant health professions, disciplines and community-based organizations.

Keywords: evidence-based public heath practice

environmental health

social justice

health disparities

consumer health

Tuesday, May 30, 2017, 3:00 PM – 4:25 PM
Room: 607

Session: Practical Approaches to Assessing Outreach and Marketing Initiatives at Your Library

Moderator: Stephanie Swanberg, AHIP

Lara Handler

Lessons Learned from Marketing Health Information Services to Underserved Community Members

Margaret E. Ansell, AHIP

Nursing & Consumer Health Librarian, University of Florida, Gainesville, Florida

The Health Science Center Libraries at the University of Florida, as part of an HIV/AIDS Community Information Outreach Project award from the National Library of Medicine, hosted several events outside of its typical patron base that needed to be marketed to at risk members of the local community. During this process, project team members learned much about the challenges of marketing to new populations, and as a result the have decided to be more proactive in its marketing efforts, through the formation of an interdepartmental HSCL Marketing Team.

Assessment of Marketing Library Services to Hospital Clinicians

Andrea L. Ball

Care Management and Population Health Librarian, Health Sciences Library, Seattle, Washington

Frances Chu

Clinical Liaison & Content Librarian, Health Sciences Library, Seattle, Washington

Using Google Analytics to Assess the Library’s Social Media Presence

Andrew Hickner

Web Services Librarian, Cushing/Whitney Medical Library, Yale University, New Haven, Connecticut

Effectiveness of Marketing a New Informationist Program

Ayaba Logan, AHIP

Research and Education Informationist/ Assistant Professor, Medical University of South Carolina, Charleston, South Carolina

Tuesday, May 30, 2017, 3:00 PM – 4:25 PM
Room: 608

Session: From Dreaming to Doing: Implementing Research Assessment Services

Moderator: Kristi L. Holmes

From Dreaming to Doing: Implementing Research Assessment Services

Karen Gutzman

Impact and Evaluation Librarian, Galter Health Sciences Library, Chicago, Illinois

Christopher W. Belter

Informationist, NIH Library, Silver Spring, Maryland

Sally A. Gore

Research Evaluation Analyst, University of Massachusetts Medical School, Worcester, Massachusetts

Terrie R. Wheeler

Director, Samuel J. Wood Library, New York, New York

Liza Chan

Research Librarian, J. W. Scott Health Sciences Library, Edmonton, Alberta, Canada

Thane Chambers

Research Librarian, John W. Scott Health Sciences Library, Edmonton, Alberta, Canada

Michael E. Bales

Research Impact and Evaluation Informationist, Samuel J. Wood Library, New York, New York

Cathy C. Sarli, AHIP

Senior Librarian, Becker Medical Library, St. Louis, Missouri

Amy M. Suiter

Scholarly Publishing Librarian, Bernard Becker Medical Library, Saint Louis, Missouri

Ya-Ling Lu

Informationist, National Institutes of Health, Bethesda, Maryland

Caitlin Bakker

Biomedical/Research Services Liaison, Bio-Med Library, Minneapolis, Minnesota

Objectives: This session will start with panel presentations by health sciences librarians from six government, private, and public research institutions in the United States. The presentations will highlight ways that librarians have combined their research, analytic, and visualization skills with a growing range of evaluative data sources to provide innovative research assessment services at their institutions. The presentations will also discuss how implementing these services has helped advance their institutions' research and strategic endeavors and allowed the libraries to contribute more directly to the research missions of their institutions. The session will offer an interactive online component facilitated through a shared Google document. Following the presentation, there will be an interactive discussion with attendees about the role of librarians in research evaluation, the service models through which these services can be delivered, and the skills necessary to perform them. This session will conclude with a perspective on how research assessment services can not only expand the practice of librarianship, but also allow librarians to more effectively contribute to the bigger picture at their institutions.

Keywords: research assessment, research impact, evaluation, library services, bibliometrics

Tuesday, May 30, 2017, 3:00 PM – 4:25 PM

Room: 611

Session: Lightning Talks 3

Moderator: David Bickford

Hosting a Science Hack Day...and You Can Too!

Erin Foster

Data Services Librarian, Ruth Lilly Medical Library, Indianapolis, Indiana

Robin Champieux

Scholarly Communication Librarian, Ontology Development Group, Portland, Oregon

Objectives: As part of the Open Insight Portland (PDX) initiative, several medical librarians, graduate students, and local open source developers and scientists collaborated to host a Science Hack Day event in Portland, Oregon. Open Insight PDX is a project, co-sponsored by Oregon Health & Science University Library and the National Library of Medicine, designed to stimulate early career researchers' engagement with open science practices through hands-on learning and conversations with leaders in the field. Science Hack Days are nationally and internationally held events organized to catalyze innovation by providing a space for people to identify and develop solutions to science problems, share ideas, and build new interdisciplinary connections.
Methods: In order to host a Portland Science Hack Day, the event organizers reached out to local and national organizations for sponsorship, identified complementary partnerships within the local community, and encouraged interdisciplinary participation by using the diversity of the organizers' backgrounds to promote the event. The event schedule and activities were modeled after previous Science Hack Days.
Results: The Portland Science Hack Day took place October 7-8, 2016 and had approximately 75 attendees that participated in both days. The event kicked off with several lightening talks by open science advocates, including representatives from local open data and hardware groups, Mozilla, and the key note speaker, Jenny Molloy – a UK biologist who works on developing open tools and software. These talks were followed by team formation, which involved linking together individuals with complementary skill sets to work on "hacking" (i.e., generating quick and often creative solutions to) science problems/projects proposed by attendees. The event was 24 hours, including an overnight camp-out option, and culminated in an open house where the attendees showcased their project work.
Conclusion: Due to the success of the initial Portland Science Hack Day, there are plans to hold the event again in the fall of 2017. Additionally, the collaboration between event organizers and attendees has resulted in additional development and promotion of open science events, open tools, and open workflows geared toward supporting and enabling innovative scientific research.
Keywords: Open Science, Science Hack Day, Community Event, Interdisciplinary collaboration

Clinical Decision Making for Dental Students: The Outcomes of a Collaborative Evidence-Based Curriculum Designed by Dental Faculty and Health Sciences Librarians

Vida M. Vaughn

Assistant Director, Kornhauser Health Sciences Library, Louisville, Kentucky

Pauletta G. Baughman

Associate Clinical Professor, General Dentistry and Oral Medicine, Louisville, Kentucky

Gustavo Oliveira

Clinical Assistant Professor, Operative Dentistry, Chapel Hill, North Carolina

Elizabeth M Smigielski

Nursing Student, MEPN Program, School of Nursing, Louisville, Kentucky

Objectives: Evidence-based dentistry relies on effective critical thinking skills. This study measured the outcomes of a critical-thinking and evidence-based dentistry curriculum developed and taught to first year dental students by dental school faculty and clinical librarians.

Methods: In the course of five-hours of class time, dental school faculty taught summary lectures on evidence-based dentistry. Librarians gave hands-on lectures about the acquisition and appraisal of evidence-based dentistry resources. A dental pharmaceutical company sales representative presented a 15-minute sales pitch on tooth whitening products to simulate an office sales call. Students then applied evidence-based practice skills in locating and appraising literature related to the presented product. Critical thinking skills were utilized to analyze the representative's sales claim. A validated critical thinking assessment tool was used to measure students' critical thinking performance

Results: Ninety one percent of students (n=108) demonstrated a high/positive response in considering multiple perspectives in subject matter. Eighty percent of students scored high/positive in understanding the components of a clear question. Regarding teaching performance, 90% found that the teachers supported the student’s ability to demonstrate good thinking, to apply insight to new situations, and to make the students feel engaged in the class. Eighty percent rated the instruction as high/positive impact on the development of the students’ skills in working through complex clinical question.

Conclusion: The use of dental faculty and librarians to teach an applied, “real life” evidence based dentistry curriculum improved first year dental students’ critical thinking skills and engaged them during class, as measured by a validated critical thinking skills inventory.

Keywords: Critical Thinking Evidence-based Dentistry Clinical Librarians Dental Faculty Multidisciplinary Curriculum

Developing an Ontology Research Guide

Lynne Frederickson

Informationist, Taubman Health Sciences Library, Ann Arbor, Michigan

Objectives: Ontologies facilitate a common understanding of biomedical concepts and enable data discovery, access and reuse. Although they play a key role in research, ontologies and other language-based organizational systems are oftentimes misunderstood, in particular by researchers that are new to data science. We have created an online research guide to support researchers who need to understand, create, or use ontologies.

Methods: A survey of existing research guides determined that although ontologies were mentioned in some guides, there was no comprehensive guide available to support researchers who need to understand, create, or use ontologies.

A comprehensive research guide for biomedical ontologies was developed to fill this gap. This guide presents and defines ontologies and other language-based organizational systems such as controlled vocabularies and codesets, and presents a curated list of resources to support their development and use. The guide also lists and describes the main biomedical ontologies and terminologies, and provides resources for researchers interested in developing a domain-specific ontology, including links to tools, training, and other information.

An overview of the concepts and content of the guide and an assessment of the guide's usefulness and impact will be presented, as well as plans for future additions to the guide.

Results:

Conclusion:

Keywords: ontologies
terminology
data access and reuse
data science
biomedical concepts

An Evidence Mapping Approach to Discovering Roles of Librarians in Systematic Reviews

Emily Ginier

Informationist, University of Michigan Taubman Health Sciences Library, Ann Arbor, Michigan

Patricia F. Anderson

Emerging Technologies Informationist, Taubman Health Sciences Library, Ann Arbor, Michigan

Objectives: Systematic reviews standards and methodology articles often designate skills and competencies required for those responsible for various tasks in the process, however the competencies and training expected vary by article. The goal of this literature review project was to extract the roles which have been identified as appropriate for librarians to perform as members of systematic review research project teams.

Methods: A literature search was conducted to identify systematic review standards and articles discussing librarian roles in systematic reviews and meta analyses. Articles were also identified through citation mining of included articles and standards. Inclusion and exclusion criteria were identified, and articles assessed by two reviewers. In the case where there was a question regarding inclusion of a role, the reviewers followed a consensus building strategy, and documented the resulting criteria from those discussions. If the original source was unclear as to which team member performed a required task, the task description and context was assessed by the co-authors to determine inclusion or exclusion, including the same consensus building strategy. For each identified role extracted from any article, data was also captured regarding how many articles of the selected set included that role.

Results: Twenty-five articles were discovered through the iterative search process, and librarian roles extracted from these. Over sixty roles were identified for librarians in the systematic review process. These fall into the broad groups of project management, support and training, literature searching, generation and delivery of results and data, post-search process, publication process, and the post-publication process. Results are described by frequency of specific roles appearing across the range of articles, and the density of roles described in specific articles.

Conclusion: Librarians bring a rich range of expertise and competencies to the systematic review process and can be deeply valuable resources and active members of systematic review teams.

Keywords: systematic reviews, librarian roles

Dream Big: Hosting a MakerHealth Faire

Jennifer Herron

Emerging Technologies Librarian, Ruth Lilly Medical Library, Indianapolis, Indiana

Objectives: Host a MakerHealth Faire that was dreamed up in the hopes of promoting not only 3D printing, but the MakerHealth movement, and the resources available on campus to the medical school community to assist in these ventures. Additionally, including a blend of different backgrounds to present their work to help identify areas for interprofessional collaboration.

Methods: Host a daring MakerHeath Faire!

"Makers" on campus were identified and invited to present their work at a table during the event. 18 tables were filled and included presenters from the schools of: Engineering, Informatics, and Health & Rehabilitation Sciences. Additional tables were filled by the university's information technology services, and advanced visualization lab. A local Makerspace was also invited and presenting at a table.

To generate even more ideas and help attendees understand impacts of "making" a panel discussion was created with some of the leading makers discussing their work. Panelists' backgrounds included: pathology, occupational therapy, informatics, dentistry, and advanced visualization. The MakerHealth Faire will be made available to students, faculty, and staff not on the campus by livestreaming table presenters and the panel discussion and making a recording available for viewing after the event.

Results: The MakerHealth Faire is just weeks away and there are no results to report yet. All tables have been filled with makers from a variety of backgrounds and promotional material has already generated buzz amongst the medical school community.

Conclusion: I plan to include number of attendees and survey results from attendees on the event. Additionally, will include list of table topics discussed by presenters. Survey results and success of event will determine if this becomes an annual affair for the library.

Keywords: 3D Printing; MakerHealth; MakerFaire; Making

Development of Data Science Education Resources for Use by Anyone, Anywhere

Nicole Vasilevsky

Biocurator/Research Assistant Professor, OHSU Library/Ontology Development Group, Portland, Oregon

Jacqueline Wirz

Assistant Dean, Oregon Health & Science University/Office of the Dean, Portland, Oregon

Bjorn Pederson

Instructional Designer, OHSU/Department of Medical Informatics and Clinical Epidemiology, Portland, Oregon

Ted Laderas

Post-doctoral Research Fellow, OHSU/Department of Medical Informatics and Clinical Epidemiology, Portland, Oregon

Shannon McWeeney

Associate Director, Translational Bioinformatics, OHSU/Department of Medical Informatics and Clinical Epidemiology, Portland, Oregon

Bill Hersh

Professor and Chair, OHSU/Department of Medical Informatics and Clinical Epidemiology, Portland, Oregon

David Dorr

Associate Professor and Vice Chair, Clinical Informatics, OHSU/Department of Medical Informatics and Clinical Epidemiology, Portland, Oregon

Melissa Haendel

Associate Professor/Director of the Ontology Development Group, Oregon Health & Science University Library, Portland, Oregon

Objectives: A current trend in medical libraries is how to help scholars and patrons engage with 'big data', or data whose size or complexity confounds usual analytics. Oregon Health & Science University is working on two efforts to provide access to 'big' data science education by holding skills courses and developing Open Educational Resources (OERs) for use by anyone.

Methods: This project took a two-pronged approach: 1) to offer skills courses in an in-person learning format to diverse audiences, from beginner to more advanced students, including librarians, graduate students, clinicians-in-training, post-docs, staff and faculty; and 2) to develop OERs that include slide decks, video tutorials, exercises, and recommended readings (available at: https://dmice.ohsu.edu/bd2k/). The courses and OERs cover a variety of topics including problem description, data set discovery, preparation and creation of data sets, implementation of analytic techniques, ethics, and effective communication. The OERs are designed to be remixed and reused by users - be it professionals or novices. To demonstrate utility for the library community, we mapped the topics in the OERs to the professional competencies outlined by the Medical Library Association. Feedback for the skills courses and OERs were collected by online evaluation forms.

Results: To date we offered five in-person skills courses, taught in a variety of ways, from a one week intensive, 2-4 evenings, and half-day sessions. Evaluations and feedback from the skills courses were positive, with weaknesses in areas of engaging the students in discussion, providing a balance between instruction and practice, and creating content that is appropriate for the stated level of the class. The OERs cover 20 topics, and are freely available online for educators and learners who can use them "out of the box" or adapt them for their own purposes, such as use by librarians to help satisfy data sharing, data rigor, or reproducibility requirements. These modules address several of the professional competencies outlined by the Medical Library Association and a mapping is available on our website (https://dmice.ohsu.edu/bd2k/mapping_MLA.html).

Conclusion: The ultimate goal of these training opportunities is to educate diverse learners as data scientists - including librarians - in order to improve best practices in training future scientists and, ultimately, knowledge discovery. We encourage librarians to use or repurpose these materials for educational purposes, such as in continuing education or professional development or for dissemination to instructors in various fields.

Keywords: data science, open educational resources, skills courses, curriculum development, educational opportunities, big data, training

Online Research Data Management Training Modules for Health Sciences Librarians

Alisa Surkis

Head, Data Services and Translational Science Librarian, NYU School of Medicine, New York, New York

Catherine Larson

Head, Systems & Technology/Systems Librarian, NYU Health Sciences Library, New York, New York

Karen Yacobucci

Content Management Librarian/Marketing Coordinator, NYU Health Sciences Library, New York, New York

So-Young Oh

Senior Instructional Designer, Institute for Innovations in Medical Education, New York, New York

Suvam Paul

Senior Data Analyst, Institute for Innovations in Medical Education, New York, New York

Colleen Gillespie

Direct, Division of Education Quality, Institute for Innovations in Medical Education, New York, New York

Kevin B. Read

Knowledge Management Librarian, NYU Health Sciences Library, New York, New York

Objectives: Academic health sciences librarians with extensive experience teaching research data management (RDM) to researchers and librarians sought to disseminate their knowledge to medical librarians nationwide. Their objective was to provide biomedically focused educational material about RDM best practices and the current RDM and data sharing landscape, and context on the differences between clinical and bench researchers' workflows and RDM challenges.

Methods: With funding from the NIH Big Data to Knowledge Initiative, seven biomedically focused online modules were created. The module topics included the following: 1) The story of data; 2) The data lifecycle; 3) Understanding researchers; 4) The RDM climate; 5) Data documentation best practices; 6) Data standards; 7) Storage, preservation, and sharing. The web-based modules used interactive educational technologies and were reviewed by a collaborator with expertise in evidence-based instructional design. These modules were piloted with over 70 librarians and included online assessments of knowledge gain, satisfaction, comfort level with material, and intent to use. Qualitative follow-up interviews were held with a subset of the pilot participants to further elucidate ways in which the modules could be improved.

Results: Following the pilot testing, the material was updated, and the online modules were made broadly available. The original seven modules were updated based on pilot participant feedback. Additionally, an eighth module was added to provide advice for initiating RDM instruction, due to a need expressed in both the online evaluations and follow-up interviews.

Conclusion: This resource is an important addition to existing RDM educational materials in two important respects. First, the modules have a biomedical focus and so include examples, resources, and requirements relevant to biomedical researchers. In addition, the modules include context about the differences in how bench and clinical researchers work and how that results in differences in their key data management issues.

Keywords: research data management

biomedical researchers

web-based modules

Pass Rates and Partnerships: Addressing Changes to the North American Pharmacist Licensure Examination (NAPLEX) and Multistate Pharmacy Jurisprudence Examination (MPJE)

Laura Menard

Health Sciences Librarian, Ruth Lilly Science Library, United States, Indiana

Objectives: After the update to the NAPLEX blueprint in 2016, pass rates fell across the country. The objective of this program is to return the institutional pass rate for the NAPLEX/MPJE to pre-2016 update benchmark.

Methods: The pharmacy liaison librarian examined national trends in pass rates, surveyed colleagues, and put together a support structure for students preparing for their licensure exams leveraging library resources and spaces. The support structure consists of the following:

1.) A LibGuide, developed in conjunction with pharmacy faculty and students, as a landing page for students interested in comparing exam prep options. These options include free, institution-supported materials as well as paid subscriptions and courses.

2.) A collection development strategy that prioritizes exam prep books and other resources in an effort to support students in their test prep and improve pass rates.

3.) Cooperation with colleagues and students within the pharmacy program to market these resources via multiple communication channels.

4.) Plan in place for library-sponsored mock NAPLEX exams for the students as test time approaches.

Results: After being live for a week, the LibGuide has received 52 views. Initial student responses are good, and each one of the library resources recommended in the guide has seen an increase in circulation/use. By MLA in May, statistics on student signups for the mock exams hosted by the library will also be available. Since the students have not yet taken their licensure exams this year, more concrete results of this program will be forthcoming once the cumulative results are provided to program administrators.

Conclusion: From the response to the support program in its first week, we can infer that students were feeling anxious about the changes to their licensure exam and appreciate having guidance. Pass rates will provide more concrete evidence of the program's potential success; however, the intangible benefits of a program like this are already becoming obvious through usage statistics and initial student feedback.

Keywords: Pharmacy, NAPLEX, LibGuide, MPJE, assessment

Rigor and Reproducibility Opens the Door for a Required Library Course for Doctoral (PhD) Students

Alisa Surkis

Head, Data Services and Translational Science Librarian, NYU School of Medicine, New York, New York

Fred LaPolla

Knowledge Management Librarian, NYU Health Sciences Library, New York, New York

Nicole Contaxis

Data Catalog Coordinator, NYU Health Sciences Library, New York, New York

Kevin B. Read

Knowledge Management Librarian, NYU Health Sciences Library, New York, New York

Objectives: Librarians at an academic medical center sought to address information and data management skills gaps observed in basic science graduate students. Leadership of the graduate program recognized the need for this instruction and were strongly supportive of a library course to meet these needs, but low registration by graduate students was a barrier to providing this instruction.

Methods: The need to improve basic science graduate student competencies in information and data management was identified in discussions between the Vice Dean for Science and the Library Director. An eight class course was developed that included instruction in literature searching, citation management, data management, and data visualization. The initial offering of the course was cancelled due to low registration. At the request of the dean of the graduate program, the library added course content on rigor and reproducibility, specifically with respect to NIH requirements in this area. NIH training grants require that students receive instruction in this area, thus allowing the dean to leverage this requirement to establish the library course as a requirement of all first year PhD and MD/PhD students.

Results: The eight class course was offered to all 42 first year PhD and MD/PhD students in the graduate institute of an academic medical center, with registration split between fall and spring offerings of the course. One element of the evaluation data collected was whether students would use what they learned. Despite low registration before the course was required, the median percentage across course topics of students in the fall semester reporting that the content of each of the classes was valuable was 83%.

Conclusion: There is great value in requiring instruction in information and data management for basic science graduate students, as these are skills that they will use in their work, but these students often either do not value or do not prioritize this type of instruction, and so do not take advantage of optional instructional offerings in this area. Requirements around instruction for students on training grants in NIH rigor and reproducibility requirements can provide critical leverage for making these types of classes required.

Keywords: data management

data visualization

rigor and reproducibility

library instruction

basic science

A Systematic What? Implementing a Systematic Review Service in a Circum-Medical Setting

Kate Ghezzi-Kopel

Applied Health Sciences Librarian, Cornell University, Albert R. Mann Library, Ithaca, New York

Erin R.B Eldermire

Head, Flower-Sprecher Veterinary Library, Cornell University Veterinary Library, Ithaca, New York

Objectives: Implementation and evaluation of a service to support interdisciplinary systematic review (SR) projects at a large research university.

Methods: In response to increased need for library support of SRs in Nutritional Science, a formal service was developed to provide SR methodology guidance and collaboration with multidisciplinary research groups across campus. Since implementing the service in January 2016, the 2.5 FTE librarians on the SR team have provided 192 consultations across 11 disciplines (Applied Economics & Management, Biology and Society, Communication, Ornithology, Design & Environmental Analysis, Earth & Atmospheric Sciences, Public Health, Human Development Studies, Nutritional Science, Veterinary Medicine, and Population Medicine). Implementing a systematic review service at a non-medical university that supports research in the health sciences has required creativity and flexibility in unexpected realms.

Results: Two tiers of SR support are offered: Tier 1 includes basic consultations on methodology and search strategy development at no charge, and Tier 2 includes serving as a member of a review team and co-authorship of the resulting SR. A SR service request form and online resource guide are available to the university community. 13 requests for SR support have been received via the service request form, 6 of which were received in January and February 2017, showing a marked increase in uptake in recent months. Requests for SR support are also received via department library liaisons and direct contact with SR team members. 4 requests for SR support have resulted in Tier 2 support and are in the fields of Human Development, Ornithology, Public Health, and Population Medicine.

Conclusion: The systematic review service has expanded to include related methodologies such as scoping reviews and rapid reviews to meet the needs of a diverse patron base. Librarians supporting the service have taken a leadership role in conducting SRs and related studies, including educating scholars and peer librarians about SR methodologies. The SR service is quickly becoming popular, and 2.5 FTE librarians are maxed out on their capacity to offer support for incoming SRs and related requests. Implementation of this service has identified clear need for library support of SRs in a non-medical university setting.

Keywords: Systematic reviews, Scoping reviews, Library services, Research methodologies

Dream, Dare, Do: Rethinking Library Strategy

Heather N. Holmes, AHIP

Associate Director of Libraries, Medical University of South Carolina, Akron, Ohio

Emily Brennan

Research Informationist, Medical University of South Carolina Libraries / Research and Education Services, Charleston, South Carolina

Susan D. Hoffius

Curator, Medical University of South Carolina Libraries/Waring Historical Library, Charleston, South Carolina

Shannon Jones, MLS, AHIP

Director of Libraries, Medical University of South Carolina Libraries

Vivian Scarborough, AHIP, MLS

Systems Engineer, Medical University of South Carolina Libraries

Joshua Ivey

Learning Commons Coordinator, Medical University of South Carolina Libraries

Randall Watts, AHIP

Associate Director, University of Tennessee Health Science Center, Memphis, Tennessee

Kathryn Church

Principal Consultant, Strategic Focus Associates

Objectives: To describe the process of developing a strategic plan in order to strengthen the Libraries' capacity to serve the campus and align with the university's new strategic plan.

Methods: In January 2015, our University launched an enterprise-wide five-year strategic plan. The Libraries, under new leadership, formed a "futures" team to develop its own strategic plan to align with the university's new strategy. The consultant who led the development of the university's strategic plan also guided the futures team. The team collected internal and external data by conducting benchmarking interviews of peer and aspirational libraries, as well as focus groups of library patrons. Data and themes that emerged from benchmarking and focus group results were used to inform strategy. Library staff was involved at critical points throughout the process. For example, staff worked in small groups to brainstorm vision statements. The futures team incorporated that feedback to develop the final vision statement. Mirroring the university's strategy, the library identified five major goals. Each goal has strategic objectives with supporting initiatives.

Results: Will be made available during the presentation

Conclusion: Will be made available during the presentation

Keywords: Strategic Planning, Strategy, Visioning, Action Planning, Program Planning, Organization Development

Visualizing PubMed: Displaying Search Results by Topic, Region, and Time

Edwin V. Sperr, Jr.

Clinical Librarian, Augusta University/University of Georgia Medical Partnership, Athens, Georgia

Objectives: This project is an attempt to visualize the results of PubMed searches in a variety of different ways: topics and how they overlap with Venn diagrams, aspects of a search (MeSH categories and

subheadings) with bar graphs, geographic areas by choropleth, and how results change by year using a line graph.

Methods: Using the NCBI API (E-utilities), a series of tools have been developed to visualize PubMed searches. Querying of the API and parsing of the results is done in the browser via JavaScript/jQuery. Visualization of results is also done in the browser (for the most part with Google Charts and with the venn.js overlay of d3.js for Venn diagrams).

Results: The applications successfully display a visualization of the aggregate results of an individual search and facilitate the easy comparison of that search with others. The applications and their source code can be found at https://github.com/esperr.

Conclusion: Visualizing the results of a search in aggregate is a potentially useful way for experienced searchers to refine their search strategies. Viewing PubMed in this way is also an interesting exercise for those who utilize bibliometric analysis of the biomedical literature. Finally, these applications are intended as examples of how relatively simple programing can stitch together APIs and libraries to create new interfaces to our databases.

Keywords: PubMed,

data visualization,

bibliometrics,

database interfaces

Teaching Together: Combining Diverse Expertise into Collaborative Interdisciplinary Instruction

Rebecca C. McCall, AHIP

Clinical Librarian, University of North Carolina-Chapel Hill, Chapel Hill, North Carolina

Cindy Todd

RN-BSN Instructor, Mercy College of Nursing & Health Sciences, Southwest Baptist University, Springfield, Missouri

Objectives: A medical librarian and a nurse educator partnered to teach online courses in the Bachelor of Science in Nursing program at a Midwestern university. The goal of this instruction partnership was to improve students' professional writing skills, knowledge of information literacy and evidence-based practice concepts, and ability to integrate evidence into practice.

Methods: Previously published nursing education literature indicates that collaborations between librarians and nursing faculty are positive experiences for educators and can improve student learning. For this interdisciplinary collaboration the medical librarian and nurse educator worked together to develop a new course that focused on the basics of library research and professional writing in healthcare and co-taught an existing course on research and evidence based practice.

Results: After two years of this interdisciplinary instruction collaboration, both instructors have learned from each other new strategies for learning experiences, assessment, and evaluation. Student feedback during and after each course is very positive; the students have appreciated having instructors with different subject expertise developing assignments and guiding their learning.

Conclusion: The instructors have gained collaboration strategies that will be helpful to others interested in interdisciplinary instruction. The lightning talk will share tips and tools for successful interdisciplinary collaboration in health sciences distance education.

Keywords: interdisciplinary education, embedded librarianship, information literacy

Global Health Outreach: Organizing a Book Drive for Malawi

Karen McElfresh, AHIP

Resource Management Librarian, The University of New Mexico, Albuquerque, New Mexico

Laura Hall

Division Head, Resources, Archives and Discovery, The University of New Mexico Health Sciences Library and Informatics Center, Albuquerque, New Mexico

Patricia Repar

Director, Arts-In-Medicine Program, University of New Mexico, Department of Internal Medicine, Albuquerque, New Mexico

Objectives: This presentation will describe the organization of a book drive for the Malawi College of Health Sciences in Blantyre, Malawi. The College's library serves about 700 students, as well as health care professionals from a neighboring hospital. However, the library lacks sufficient resources, with most of their books being outdated or in poor condition, diminishing health care quality in Malawi.

Methods: The first step was to determine how to get the books to Malawi. We decided to work with the African Library Project (ALP), an organization that coordinates book drives and ships books to libraries in Africa. The Malawi College of Health Sciences submitted an application to ALP and after they were accepted, we developed a plan to collect approximately 500 gently used health sciences books. We created a set of collection criteria based on the needs of the College to ensure that all the books we sent were as relevant and recent as possible. Internet access in Malawi is not reliable, so print books are essential. We solicited donations at our campus and within our community, and collected books for about two months. Books were organized by subject as they were received in order to facilitate the shipping process.

Results: The book drive will be completed in early May 2017. Results will be made available during the presentation at MLA 2017.

Conclusion: Conclusions will be made available during the presentation at MLA 2017.

Keywords: global health, outreach, Malawi

Library Participation in Center for Transdisciplinary Evidence-Based Practice (CTEP) Evidence-Based Practice Education

Marie St. Pierre, AHIP

Medical Librarian, Clinical and Research Library, Children's Hospital Colorado, Aurora, Colorado

Catisha Benjamin

Manager, Clinical and Research Library, Children's Hospital Colorado, 80045, Colorado

Objectives: CTEP is the Center for Transdisciplinary Evidence-Based Practice, Ohio State University. They provide nurses/allied health with one-week intensive workshops. We were asked to teach the library use part, and we went through the full course to learn all aspects. We formed PICO/t questions, help critique others' projects, gathered our evidence and helped our classmates.

Methods: We taught the library part, in mentoring time we assisted, and we learned along with the others and did our own project too during the week-long workshops.

Before the workshop the second year, we were asked by several nurses if we could prepare and teach some classes on general computer skills/library skills such as PubMed, CINAHL, and note, so they would feel more comfortable before getting to the CTEP class itself.

Results: We saw the EBP process from start to finish, and learned how nurses research the literature and how they apply it to their practice, learned more about how and why evidence based practice is used, which gives us a better understanding of how we can help.

The prep classes were appreciated and the nurses felt more confident in the CTEP workshop, and we librarians were put to good use since the students knew us and felt comfortable with our knowledge. They do not hesitate to come to us with EBP questions now that the workshop is done, and we have educated nurses since then in this style.

After CTEP, we began using a PICO/T question reminder on our literature search request page, and we now very frequently get literature search requests in the form of a PICO/T question.

Conclusion: Librarians can enrich their own understanding of evidence based practice by participating, as either instructors or students in 'train the trainers' type courses of this type. In modern nursing and allied health, EBP is becoming more used as a gold standard, so it is incumbent on medical librarians to have these skills to then teach others. We know that at Children's, evidence based practice (EBP) is indeed being used, taught to the new nursing grads, and we know that we need to be well-informed on EBP and to become qualified CTEP mentors. In keeping with the spirit of this MLA conference, Dream to assist your clinical staff in getting to know CTEP/EBP more, Dare to become familiar with CTEP or EBP and Do it.

Keywords: evidence based practice, education, clinical, nursing staff, allied health

Tuesday, May 30, 2017, 3:00 PM – 4:25 PM

Room: 612

Session: Daring to Serve All: Medical Librarian Engagement with Diverse Populations

Moderator: CeCe Railey, AHIP

LGBTQ2+ Health Research, Resources, and Promotion: Building an Academic, Hospital, and Public Library Partnership to Support Pride Health Needs and Information

Robin MN Parker

Evidence Synthesis and Information Services Librarian, Dalhousie University, Halifax, Nova Scotia, Canada

Katie McLean, AHIP

Librarian Educator, Staff Training & Education, Health Sciences Library, Halifax, Nova Scotia, Canada

Objectives: People in the LGBTQ2+ community have unique challenges when it comes to accessing healthcare and health information. In order to support the information needs of this community as well as the healthcare providers, researchers, and policy makers involved in delivering and improving health services, we developed a LGBTQ2+ Health Information LibGuide through a partnership between academic, hospital, and public libraries.

Methods: Working with the prideHealth coordinator for Nova Scotia Health Authority and public services at Halifax Public Libraries, we collected and vetted health information resources specific to the needs of the LGBTQ2+ health consumers. To support LGBTQ2+ health researchers and providers, we also assembled research evidence and methods resources. These health information resources are presented using the LibGuide platform to highlight services provided by the partners in this project. In order to meet the needs of the various information seekers, we consulted with individuals working in academic and health care delivery settings as well as members of the LGBTQ2+ community. Promotion for the guide includes distribution through the partnership and additional communication to key stakeholders involved with LGBTQ2+ health. We assess the impact of LGBTQ2+ Health Information LibGuide by examining the usage statistics and reviewing usability with our target audience.

Results: Results are forthcoming and will be presented during the presentation at MLA '17.

Conclusion: Conclusions will be presented during the presentation at MLA '17.

Keywords: LGBTQ2+ health, research support, hospital library, library partnership, consumer health, queer health information, information needs

Dare to Dream: Promoting Indigenous Children's Interest in Health Professions through Book Collections

Sandy Iverson

Manager, Health Information & Knowledge Mobilization, St. Michael's Hospital, Toronto, Ontario, Canada

Monique Clar

Librarian, Bibliothèque de la santé, Montreal, Quebec, Canada

Objectives: Indigenous peoples in Canada face significant health challenges. They represent 4.3% of the population, but less than 1% are doctors, and few pursue careers in the health sciences. We will present descriptions of innovative programs by medical librarians in two Canadian provinces designed to encourage children in First Nations communities to dream of careers in the health professions.

Methods: Case studies of two special projects will be presented in this paper. The first project was led by a university library in Quebec. Book collections in science and health were developed for Indigenous school libraries. Library and information science students, as well as a librarian from the University were involved in health and education related activities in the recipient schools. This experience informed the second project, which was adopted as the community service project of the joint CHLA/MLA/ICLC Mosaic 2016 conference. The mechanics, benefits, and challenges of the programs will be discussed including: methodology for selecting books, methodology for collecting or purchasing books, and partnerships formed for distributing books to remote First Nations communities. Some qualitative evaluative data collected informally from recipient communities through conversations with teachers and community leaders will provide evidence of the value of these projects.

Results: Hundreds of books have been delivered to First Nations schools in both Quebec and Ontario. Qualitative evaluative data collected from the recipient communities indicates that the books are well received and has allowed us to identify children's favorite topics. This information will iinform future updating of the collections in the ongoing project. Some difficulties in providing optimal access to the

books were identified due to communication problems or the relative lack of library infrastructure in these schools.

Conclusion: Reading for pleasure is linked to student's academic success. Access to varied and quality literature is therefore important for school achievement. Books are often in limited quantites in remote communities, and it is believed that the books donated will benefit both the students and their communities. Even if the effect of these collections on impacting the students' future decisions to enter in to health professions cannot be easily measured, potential impact on future life chances and the fact that indigenous high school graduates choosing to pursue schooling tend to select a profession linked to the needs of the community are enough to consider those two programs as successful.

Keywords: Health professions

Aboriginal peoples

Indigenous peoples

First Nations

Canada

Children

Consumer health

Children's literature

Library school students

Community engagement

Research Services for the Functionally Diverse

JJ Pionke

Assistant Professor and Applied Health Sciences Librarian, Social Sciences, Health, and Education Library at UIUC, Champaign, Illinois

Objectives: As an under-served population, the functionally diverse often struggle to get assistance that takes their needs into account. The Research Services for People with Disabilities Pilot Project sought to improve services via training of reference specialists in order to more holistically assist patrons with disabilities (the functionally diverse) and engaging in an ongoing dialogue with users to further refine services.

Methods: A previous project that interviewed patrons with different kinds of disabilities indicated the need for more focused assistance. Once the need for a more robust assistance model was defined, the pilot project took shape. We began by exploring the policies of other institutions, identifying current gaps in services and training, and conversing with disability access specialists about the needs of current students, faculty, and staff. Next, we engaged in reflection about the project: lessons learned and impact.

Results: The project has had very mixed results - some of it positive and some of it negative. Regardless, it's been a learning experience in what we are doing right and what we are doing wrong.

Conclusion: The project is transitioning and broadening into a more sustainable and ongoing model which includes continued use of the program as a test bed for ideas as well as a cycle of reflection and growth.

Keywords: disability, pilot project, reference, user services, training, interviews, accessibility

Developing Locally Relevant, Easy-to-Read HIV/AIDS Print Resources

Margaret E. Ansell, AHIP

Nursing & Consumer Health Librarian, University of Florida, Gainesville, Florida

Hannah F. Norton, AHIP

Interim Fackler Director, Associate University Librarian, University of Florida, Gainesville, Florida

Ariel Pomputius, AHIP

Health Sciences Liaison Librarian, Health Science Center Library, Gainesville, Florida

Michele R. Tennant, AHIP

Interim Fackler Director, Health Science Center Libraries, Gainesville, Florida

Nina C. Stoyan-Rosenzweig

Senior Associate in Libraries, Health Science Center Library, Gainesville, Florida

Objectives: As part of an HIV/AIDS Community Information Outreach Project funded by the National Library of Medicine, the project team at the University of Florida (UF) developed a series of locally relevant, easy-to-read print handouts on HIV/AIDS testing, including the difference between anonymous and confidential testing, the process of getting an HIV test, and information about local HIV support groups.

Methods: Librarians at UF discovered during discussions with local community organizations that providers of free or low-cost health-related services had a high need for easy-to-read print handouts that offered information about locally available resources. During the project, the team conducted an in-depth information needs assessment with representatives from community organizations that identified several key topics upon which the team could focus their efforts. The team drafted documents on anonymous & confidential testing, the testing process, and local support groups, and an external evaluation panel reviewed them for accuracy and clarity. The team also verified information about HIV testing locations in Gainesville and Jacksonville and included the list of locations and their contact information on the back of the two testing documents.

Results: The print resources were distributed to local community organizations as hard-copies and were made freely available online. The team plans to evaluate the impact of the resources on the ability of local community organizations to provide HIV/AIDS information to their users.

Conclusion: During outreach efforts, librarians can better meet the information needs of their intended audiences if community partners are included in project planning from the outset. In the case of this project, despite the availability of trustworthy HIV/AIDS resources online, community partners made it clear that for high-risk populations with limited access to the internet and a low level of literacy, easy-to-read print handouts were a simple and feasible way of sharing trustworthy HIV/AIDS information quickly and in appropriate amounts. By including information about local HIV testing locations, the handouts provide potential contacts for users interested in seeking additional help.

Keywords: consumer health, easy-to-read, HIV/AIDS, resource development, patient handouts

Field Testing--and Improving--a Health Advocacy Web-Based Curriculum

Alexa Mayo, AHIP

Associate Director for Services, Health Sciences and Human Services Library, Baltimore, Maryland

M.J. Tooey, AHIP, FMLA

Associate Vice President, Academic Affairs/Executive Director, HSHSL, Health Sciences and Human Services Library, Baltimore, Maryland

Objectives: In 2014, the Project SHARE team developed a web-based curriculum to build students' health advocacy skills. In 2016, five Area Health Education Centers (AHECs) in diverse settings chose the curriculum for a national health literacy project. This presentation describes how the Project SHARE team used the students' and instructors' experiences in the field to enhance and improve the curriculum.

Methods: In 2014, the Project SHARE team developed a web-based health advocacy curriculum aimed at building students' skills to advocate for improved health at the personal, family, and community level. It comprises six modules: Overview of Health Disparities; Quality Health Information; Taking Charge of Your Health; Smart Food Choices; Promoting Health and Wellness in Your Community; Crafting and Delivering the Message. Each module contains lessons plans and experiential learning activities. In 2016, five AHECs in frontier, urban and rural settings chose the curriculum to support a national health literacy project. This presentation will report on the effectiveness of the curriculum in the field and will describe how the Project SHARE team used students' and instructors' in-the-field experiences to expand the curriculum. Areas addressed include developing content in health equity and creating innovative ways to more fully engage students.

Results: With funding from the Center for Public Service Communications, the Project SHARE team incorporated the five AHECs' (year 1) field experiences into the curriculum. Three AHECs – Southeastern Primary Care Consortium Atlanta AHEC, Eastern Connecticut AHEC, and Southeast Pennsylvania AHEC - are using and adapting the SHARE curriculum in year 2 of the national health literacy project.

Conclusion: By incorporating recommendations from lessons learned in the field, the Project SHARE curriculum stays relevant as it builds students' health advocacy skills.

Keywords: health literacy; health advocacy; community outreach; community engagement; curriculum; health equity.

Tuesday, May 30, 2017, 3:00 PM – 4:25 PM

Room: 604

Session: Data Librarians: Daring to Move Beyond Traditional Roles

Moderator: Magaret Peloquin

"Databrarians" and Beyond: Competencies, Education, and Requirements for an Emerging Role

Lisa M. Federer, AHIP

Research Data Informationist, NIH Library, Bethesda, Maryland

Objectives: As science becomes increasingly data-intensive, many information professionals have found new roles as data specialists. This study considers the professional and educational backgrounds of data specialists and the range of competencies and expertise that help them achieve success. These findings have potential implications for continuing education, as well as training for the next generation of information professionals and data specialists.

Methods: This study employs a mixed methods approach to examine the types of positions in which data specialists work, as well as the educational and professional backgrounds of the individuals in these positions. First, job announcements from relevant sites were identified and analyzed to identify common requirements for education, experience, and competencies. Second, a survey was distributed to librarians and information professionals who self-identify as data specialists. This survey asked respondents about their actual educational and professional backgrounds. Respondents were also asked to identify the competencies they considered most important to their success in their position.

Results: Results will be made available during the presentation at MLA '17.

Conclusion: Conclusions will be made available during the presentation at MLA '17.

Keywords: data management, competencies, professional education, data science, research support

Adding to the Evidence: A Survey of Faculty Research Data Management Practices and Needs

Margaret Henderson, AHIP

none, none, Ramona, California

Objectives: A faculty survey was conducted to understand research data management practices and faculty needs to develop research data services for a university. The Library worked with stakeholders throughout the university to develop questions and support institutional policy. Data collected will be compared to similar surveys at other universities to help develop an evidence base for setting up institutional

Methods: A survey was developed in conjunction with the office of research, technology services, and other stakeholders at the institution. IRB approval was received, and the survey was sent to all teaching and research faculty using REDCap. Two reminders were sent to faculty before the survey was closed. Data will be analyzed using SPSS. All university faculty were surveyed. The full set of results will be compared to similar multidisciplinary surveys at other institutions. A subset of results from biomedical departments will be used to compare the university responses to several recent studies based in biomedical libraries. The similarities and differences between disciplines and institutions will be considered for the development of policies and contributions to an evidence base for research data management services in libraries.

Results:

Conclusion:

Keywords: research data management, surveyy, evidence base, faculty

Training in Support of Data-Driven Research: A Qualitative Study of Library Workshops in Top National Institutes of Health-Funded and National Science Foundation-Funded Universities

Tanja Bekhuis, AHIP

Principal Scientist, TCB Research & Indexing LLC, Pittsburgh, Pennsylvania

Objectives. In an age of data-driven research and competitive funding, libraries help their patrons acquire new skills. To do so, some offer workshops about knowledge discovery, analysis, and management of various kinds of data. The primary objective of this qualitative study was to explore the nature of workshops offered by libraries to support data-driven research in top NIH- and NSF-funded universities. Additionally, we developed a catalog of workshops, and indexed the resources and thematic content.

Methods. To identify top-funded universities, we used NIH Research Portfolio Online Reporting Tools and the NSF Budget Internet Information System. From corresponding websites, we extracted information on 99 workshops offered by health sciences libraries (n=5) and main libraries (n=5) in schools funded by NIH and NSF, respectively. Workshop title, duration, and description were catalogued by library and source of federal funding for the university. We used NVivo 11 Pro (QSR International) for qualitative data analysis and TExtract® (TEXYZ) to semi-automate indexing the content of the catalog. Themes were first identified in textual patterns and then were refined by an analyst. Thematic overlap was described across funding source. Additionally, we identified themes unique to each subset.

Results. Main libraries in NSF-funded schools offered 36% more workshops than health sciences libraries in NIH-funded schools (57 vs 42). Overall workshop duration ranged from 1 to 16 hours in a bimodal distribution (1st mode = 1 hour; 2nd mode = 3 hours). The distribution of duration for NSF schools differed from the NIH distribution. We identified 15 main themes overall: *statistical programming* and *data visualization* occurred most often, and *finding funds for research* and *open science* least often. Thematic distributions varied with funding source. For example, *bioinformatics* occurred most often in the NIH-funded subset and *statistical programming* in the NSF subset. For each subset, 20 most informative indexing terms were identified after sorting and discretizing into 7 quantiles. Top indexing terms included: *data visualization*, *pathway analysis*, and *data management* (NIH schools); *data analysis*, *data management plan (DMP)*, and *Python* (NSF schools).

Conclusion. A catalog of workshops organized by university funding source and library, along with 2 indexes (resource and subject), will be publicly available. The analytical results, as well as index content, yield insights regarding workshop coverage. Implications for strategic planning and development of library workshops in support of data-driven research will be discussed.

Keywords. Research libraries, data-driven research, library workshops, strategic planning, competitive advantage

REDCap and Trade: Negotiating a Library Role in Clinical Research Data Management

Kevin B. Read

Knowledge Management Librarian, NYU Health Sciences Library, New York, New York

Fred LaPolla

Knowledge Management Librarian, NYU Health Sciences Library, New York, New York

Objectives: REDCap, an electronic data capture tool, supports good data management but many researchers lack familiarity with the tool. A service gap was identified in an academic medical center where a REDCap administrator provides technical support and a clinical data management unit provides study design support. Librarians with REDCap expertise sought to increase and improve usage through outreach, workshops, and consultations.

Methods: Two librarians developed REDCap expertise by building forms for a clinical quality improvement project and a clinical research study supported by an informationist supplement from the National Library of Medicine. In consultation with a REDCap administrator and the director of the clinical data management unit, a role for the library was established in providing REDCap training and consultations. Liaison relationships led to consultations, with an increase in requests following a REDCap course offered by the library to the medical center community. Data was collected from the REDCap course assessing the level and quality of material, effectiveness of presenters, knowledge gained, and interest in other classes. Consultation data was collected from each appointment, outlining the researchers' department, the nature of the consult, referrals to other departments, and how researchers' learned about the library offering this service.

Results:

To date, the library has conducted 6 workshops, and 28 consultations that were held in-person or via email. The majority of interactions included basic training on the REDCap database for faculty, administrators and students across several departments. Radiology, College of Nursing, Emergency Medicine, and Internal Medicine were the most frequent audiences. Users heard about the library offering REDCap services most often through previous encounters with our Data Services Team, referrals from liaison librarians, and a regular data training series offered by the library three times a year. In turn, the library referred users to our clinical data management unit 7 times and the REDCap administrator 9 times.

Conclusion:

Filling a gap by offering REDCap training has provided the library's Data Services Team with a new opportunity to add to their existing suite of services. Offering REDCap training in collaboration with our clinical data management unit and REDCap administrator has created a more seamless workflow for providing clinical data management support, and has increased the level of communication the library engages in with these departments.

Keywords: REDCap

Clinical Data Management

Research Data Management

Data Services

Outreach

Teaching

Consultations

Workshops

Workshops

Visualizing Success: Development of a Data Visualization Service in an Academic Medical Library

Fred LaPolla

Knowledge Management Librarian, NYU Health Sciences Library, New York, New York

Objective: Data visualization is an important element of scholarly communication and analysis. A request for data visualization instruction came from the Associate Dean of our graduate studies program, and it became clear that educational support and guidance for data visualization services was an unmet need at our academic medical center, which the library sought to fill.

Methods: Our health sciences library began developing course materials on data visualization with a focus on fundamentals of visualization and design best practices, which was leveraged into a stand-alone, introductory class. A librarian new to data visualization services engaged in training through online resources including Coursera and Lynda, attended community-based meetups, and read literature on the topic. The librarian gained practice by visualizing library bibliometric data. Data visualization services were marketed through our medical center's online internal portal, listservs and liaison librarians. A second workshop was created on communication for different audiences in response to a departmental request, which was generated as a result of these marketing efforts. These resources were leveraged into classes for the general medical center community, which in turn led to opportunities for data visualization consults and an participatory "Data Visualization Clinic."

Results: To date, uptake at the medical center has been strong. Over 10 class sessions and workshops have been held in data visualization topics in the past year, including sessions requested by individual departments as well as classes hosted in a recurring library class series. Sixteen consults with either individual researchers or small groups of researchers have been held. In addition to classes on introductory data visualization topics, the library has hosted several successful data visualization clinics and developed an online social media community on our medical center's intranet with 29 members currently. A new educational offering has also been developed on software offered at our medical center.

Conclusions: Data Visualization offers a relatively new area for libraries to provide data services. Because enthusiasm for the topic is high, buy-in from departments can be quickly achieved. Moreover, the high volume of quality, online educational resources that many libraries already subscribe to or that can be used for free online allow librarians to gain knowledge in this service area. Additionally, for many librarians meetup and common interest groups offer a community oriented approach to learning.

Keywords: data visualization, data services, data lifecycle, library led workshops, data education, library education, technology, innovation, departmental collaboration, scholarly communication

Tuesday, May 30, 2017, 3:00 PM – 4:25 PM

Room: 615

Session: Daring to Design: Librarian Roles in Curriculum

Moderator: Nena Schvaneveldt, AHIP

Dare Your Students to Debate: An Innovative and Collaborative Approach to Teaching Evidence-Based Medicine

Marie T. Ascher

Lillian Hetrick Huber Endowed Director, Health Sciences Library, Valhalla, New York

Ofelia Martinez

Clinical Director and Course Director, Clinical Skills Center, Valhalla, New York

Fred Moy

Course Director, Biostatistics and Epidemiology, Department of Pathology and Cell Biology, Valhalla, New York

Objectives: In Fall of 2014 a new task force was charged with evaluating and redesigning the biostatistics, epidemiology, and evidence based medicine elements of the medical school curriculum. This poster describes a collaborative project involving the course directors in Epidemiology and Foundations of Clinical Medicine II, along with the Library Director, to teach EBM in the second year of medical school.

Methods: The Library Director served as course director for the EBM component which was given designated lecture and self-study slots in both the first and second years of the medical school curriculum. The multi-part project culminated in a team debate related to breast cancer screening -- should it begin at age 40 or age 50? The students were provided first with a lecture related to screening programs. In Epidemiology they were presented with the statistical concepts related to screening and diagnosis. A librarian-led lecture introduced the EBM process, including asking questions (PICO), literature searching, and critical appraisal. And in subsequent self-directed learning slots they were to complete individual and group assignments to prepare for the debate. During the debate students exercised their new EBM skills to argue their position. Volunteer faculty from all areas including the library acted as debate moderators.

Results:

Conclusion:

Keywords: evidence based medicine, instruction, teaching, debate, self-study, self-directed learning

Patient Cases, Sticky Notes, and String: Teaching Undergraduate Students to Use a Reference Information Model (RIM) for Capturing Clinical Data

Sarah K. McCord

Associate Professor, MCPHS Library, Boston, Massachusetts

Objectives: The Health Level 7 (HL7) version 3 clinical data framework can be used to create clinical documents that capture health care interactions and support communication and compliance both within and beyond an institution. This session reports on a method for teaching undergraduate informatics students to apply the object-oriented reference information model (RIM) underlying the HL7 clinical document standards.

Methods: Students were introduced to HL7 and the RIM through background readings and lectures before being asked to apply the RIM in class using a patient case as the data source for their model. Each of the six object classes in the RIM is represented by a color, and relationships between classes are specified. With the classroom walls as their canvas, students used sticky notes corresponding to the RIM object class colors to create Refined Message Information Models (RMIMs) that encoded content specific to their cases. Students indicated relationships between objects by linking the sticky notes with string. Each student then explained his or her model to other students in the class, who offered critique. Effectiveness of this activity was measured on the final semester project, where students were asked to create ten additional RMIMs capturing interactions within their cases.

Results: After completing the readings, lectures, and in-class activities, students were able to create well-structured RMIMs using the six basic HL7 object classes, and demonstrated their mastery of this task later in the semester by successfully creating RMIMs for their final projects. In addition, students applied their practice with the HL7 reference information model to other object-oriented models discussed in a required software development course. Students commented positively on the in-class RMIM exercise in response to open-ended questions on course design and delivery on end-of-term evaluations.

Conclusion: The combination of readings, lectures, and hands-on activities is an effective method to teach the use of object-oriented reference information models to students with little background in computer or information science. An additional advantage to this method is that it does not require the use of computers or specialized software.

Keywords: Teaching, Active Learning, Undergraduates, Informatics, Information Models, Health Level 7

Daring to Do Problem-Based Learning in Undergraduate Nursing: Teaching Information Literacy and Evidence-Based Practice Skills through Scenario-Based Projects

Kathryn M. Houk

Health & Life Sciences Librarian, SDSU Library, San Diego, California

Amy Heiss

Lecturer, School of Nursing, San Diego, California

Objectives: Purpose: This paper discusses the design, implementation, and outcomes of redesigning three research projects within an undergraduate-nursing course utilizing problem-based learning techniques.

Methods: Setting/Participants: The nursing instructor and nursing librarian at a large, public university redesigned the research projects in a core undergraduate-nursing course of approximately 70 students.

Brief Description: In this project, a single clinical scenario was designed as a storyline to provide background and purpose for three research assignments. Each assignment scaffolds research skills as the semester progresses, as well as introduces students to the different information resources and types available to them. The scenario helps tie the projects together, and also helps students understand how the particular skills being developed might have real world applications in their future careers.

Results: Evaluation Methods: Information literacy knowledge gains were assessed with pre- and post-tests, as well as from the first and third assignments. Evaluation of the entire program was collected from students with an end of course survey.

Conclusion: Students were often unaware of the overarching story or how it related to the various projects. A pilot implementation is always rocky, but formative assessments and evaluations gave good insight into improvements to be made for the next implementation. We are currently in our second semester, and have made changes to our instruction approach, assignment guidance and assignment structure.

Keywords: problem-based learning, undergraduate nursing, scenario-based learning, curriculum design, information literacy instruction, evidence-based practice instruction, research instruction

Developing Competency-Based Data Literacy Instruction for Tiered Integration into Nursing Education

Kate Saylor

Health Sciences Informationist, Taubman Health Sciences Library, Ann Arbor, Michigan

Tyler Nix

National Library of Medicine Associate Fellow, University of Michigan Taubman Health Sciences Library, Ann Arbor, Michigan

Marisa Conte

Assistant Director, Research and Informatics, Taubman Health Sciences Library, Ann Arbor, Michigan

Objectives: Data literacy is an important training component for students planning a research career. Even students who don't plan to follow a research career track need a basic understanding of how clinical data can be used in research. We developed competency-based DL instruction and leveraged relationships with the (redacted) School of Nursing to integrate this into the undergraduate and graduate curricula.

Methods: The primary goals for this project are to: 1) Identify key stakeholders and foster partnerships with faculty around DL. 2) Develop competency-based DL instruction. 3) integrate DL into curricula. 4) Assess instruction and resources.

At the start of the project, we gathered information, raised awareness of library expertise in DL, and identified partners by presenting at curriculum committee, departmental, and faculty meetings.

The next stage focused on program development, which included allocating resources and building content. We worked with faculty and students to develop a nursing-specific curriculum based on the Data Literacy Competencies developed by colleagues at (redacted).

The third stage addressed program implementation. Based on input from our stakeholders and partners, we delivered the information through various channels, e.g. workshops, curriculum-integrated instruction, and online tools.

For the final stage of this project, we will conduct assessments to refine our instruction and inform future programs.

Results:

Conclusion:

Keywords: data literacy, data management, nursing, curriculum, instruction, partnerships, competencies

Do the Flip: Using the Flipped Classroom Model to Deliver Information Literacy Instruction

Sandy Iverson

Manager, Health Information & Knowledge Mobilization, St. Michael's Hospital, Toronto, Ontario, Canada

David Lightfoot

Information Specialist, Scotiabank Health Sciences Library, Toronto, Ontario, Canada

Bridget Morant

Information Specialist, Health Sciences Library, Toronto, Ontario, Canada

Carolyn Ziegler

Information Specialist, Health Sciences Library, Toronto, Ontario, Canada

Objectives: Flipped classroom is a blended learning instructional model that reverses the traditional construct of content being delivered in the classroom, followed by activities conducted outside the classroom. Traditionally, the model is used in school settings. Our experience suggests the model can work in a variety of contexts, including information literacy instruction in an academic hospital library.

Methods: In response to an expressed need for more hands on experience and individualized support in information literacy workshops, as well as a need to keep workshops within a one hour time frame, this academic hospital library experimented with the flipped classroom model in a number of their standard workshops, including workshops to teach basic search skills on PubMed and Medline. Pre-workshop content was delivered using the LibGuides content management system. LibGuides were created and included instructional videos, text, sample exercises, and contact information. Learners were expected to work through the information on the LibGuides prior to the workshops and the in-class workshop focused on reviewing the sample exercises and answering students' questions. Evaluative data from both instructors and students were collected and analyzed. LibGuide usage data was also analyzed.

Results: In one year, 168 learners participated in the flipped workshops. Evaluations were collected evaluations from both learners and instructors. We will report on this experience from both the learner and instructor point of view. We will discuss issues that arose and some of our attempts to mitigate these issues.

Conclusion: Based upon our analysis of both instructor and learner evaluations, as well as LibGuide usage data, our conclusion will explore in more detail when the flipped workshop model works and when it doesn't and provide some strategies for adapting the model. We will revisit some of the literature and compare how our experience resonates (or not) with the literature. We will discuss some of the ideas that we have for further modifications of this model to support our work.

Keywords: eLearning Flipped classroom Blended learning Library instruction Hospital libraries

Tuesday, May 30, 2017, 3:00 PM – 4:25 PM

Room: 613

Session: Maximizing the Web: Doing What We Do Best

Moderator: Michelle Fiander

Level Up: Modern Web Trends for Libraries

Nha Huynh

Education and Instruction Librarian, Client Relationship Management, Houston, Texas

Objectives: This presentation aims to provide an overview of the current trends observed among health sciences libraries' websites, identify areas for improvement, and suggest ideas for building effective, search-optimized, and user-focused web sites based on best practices in web development.

Methods: The author examined a sample of academic health sciences libraries' websites for common patterns, from the front end's user interface to the back end's site architecture . The author then identified potential areas for improvement (such as responsive design, performance, and accessibility). In addition,

the author also researched best practices from the web development industry and curated those that would be relevant for library implementation.

Results: N/A

Conclusion: For most libraries, the library's website is the primary portal through which patrons access electronic resources. Thus, it is increasingly important to ensure the design of the website is up-to-date with most common web standards, such as being responsive, accessible, and search-optimized.

Keywords: website, web design, user experience, responsive design

Tuesday, May 30, 2017, 3:00 PM – 4:25 PM

Room: 613

Session: Maximizing the Web: Doing What we Do Best

Moderator: Michelle Fiander

Search Engine Optimization and Health Libraries: Does Optimizing for Google Impact Library Site Visibility?

Emily Couvillon

Clinical Informationist & Data Management Librarian, Georgetown University, Washington, District of Columbia

Objectives: Libraries excel at optimizing holdings metadata to appear in catalog searches. However, findability is not always prioritized when structuring library web content. By neglecting to properly structure the content found on most institutional sites, libraries miss reaching a wider audience. Does implementing search engine optimization techniques to optimize websites for Google impact health science library site visibility in search results?

Methods: The author conducted a technical audit on a sample of 25 randomly selected AHHSL library websites. Moz Keyword Explorer and Google trends were used to generate 65 target keywords for health science libraries. Wincher was used to evaluate how well each library ranked in Google for these key terms. Next, the author examined the link structure of each site using Screaming Frog SEO Spider Tool. Using these tools, the author evaluated the efficacy of structuring content according to established SEO principles outlined in the Moz SEO checklist for improving library site rankings.

Results: The majority of the health library websites in the sample were not optimized according to SEO standards and had poor search engine visibility for health science topics. Those that ranked in the top 20 on search results pages met a larger percentage of the Moz checklist requirements. The highest ranking sites benefited from including well-structured XML sitemaps and original content created by librarians. Rather than providing simple lists of links and resources, successful libraries provided their own insight and analysis through subject guides and blog posts. This finding is in line with the SEO adage “content is king,” and demonstrates that libraries can improve their visibility in search engines without a high level of web development knowledge.

Conclusion: Libraries can increase the visibility of their products and services by implementing SEO standards. By creating useful content and uploading a well-structured XML sitemap to improve crawling, librarians can better demonstrate their expertise to researchers.

Keywords: SEO, Google, marketing, web development, content marketing, search engines

E-Channel: Publishing the Work of Dreamers

Christy Jarvis, AHIP

Head of Information Resources and Digital Initiatives, University of Utah, Salt Lake City, Utah

Jean P. Shipman, AHIP, FMLA

Executive Director, Knowledge Management & Eccles Health Sciences Library, Spencer S. Eccles Health Sciences Library University of Utah, Salt Lake City, Utah

Objectives: To grow and expand the Spencer S. Eccles Health Sciences Library's (EHSL) platform (e-channel) for the dissemination of creative scholarly output related to innovation through targeted content solicitation and strategic partnerships. To develop new collections based on expressed needs and institutional activity and to improve discoverability and usability through a platform re-design.

Methods: EHSL's initial incarnation of *e-channel*, an online platform for showcasing innovations was hampered by the inability to quickly accommodate new collection content and to search across collections and content types. To address these issues, a dedicated team of EHSL staff explored numerous solutions. Analysis of the options led the team to conclude that the site needed to migrate onto a WordPress platform in order to achieve the level of flexibility and scalability required. Once the migration had been executed, the e-channel team was able to focus on the development of robust and rapidly growing collections, such as the *Innovation Vault* and *Healthi4U* student-produced health videos. The integration of a customized Google Search Appliance enabled cross-platform searchability that was previously unavailable, the ability to collect usage statistics, as well as create access tiers for future revenue-generation.

Results: Six months after migrating to the WordPress platform, the number of e-channel collections grew by 75%. Enhanced site searchability and discoverability resulted in a 280% increase in video content views. Six months later, at the end of 2016, e-channel collections had grown by 150% and video content views had grown by 500%. Numerous new partners and content collaborators, both at the University of Utah and beyond, have committed to using e-channel as a platform for disseminating their creative scholarly output.

Conclusion: At a critical juncture in its development as a dissemination tool for creative scholarly innovation-themed output, the migration of *e-channel* to a more flexible and expandable platform enabled EHSL staff to focus on content solicitation and organization, rather than on system infrastructure limitations. The new site has enabled rapid creation of new digital collections and enhanced discoverability of all types and formats of content. Eliminating the alpha site's technical challenges gave staff the time to concentrate on creating additional collaborations for content development. Content can now be easily added from other innovative institutions to create the one-shop stop envisioned for e-channel – a place where innovators can find the information they need to accelerate their entrepreneurial opportunities.

Keywords: Digital collections, library publishing, innovation, creative scholarly output

Up in the Air: Using the Airwatch Mobile Device Management Platform to Provide Access to Core Clinical Apps

Whitney Townsend

Coordinator, Health Sciences Executive Research Service, Taubman Health Sciences Library, Ann Arbor, Michigan

Chase Masters

Enabling Technologies Informationist, University of Michigan, Ann Arbor, Michigan

Carol Shannon

Informationist, Taubman Health Sciences Library, Ann Arbor, Michigan

Objectives: To describe a partnership between the library, health system IT, and clinical administrators to utilize an existing health system mobile device management to increase utilization of subscription apps by medical students and clinicians in a large academic health setting.

Methods: Academic and hospital libraries and individual clinical departments are often the primary suppliers of access to subscription-based clinical health and medical resources within their institutions. Other frequently used resources are freely available to all users. This confusing information landscape is further muddied when considering access to the mobile versions of these subscription and free resources. Differences in access levels, registration procedures, and mobile device platforms can serve as barriers to medical student and clinician adoption and use of available mobile resources. This paper will describe how a team of librarians, clinicians, and health system IT personnel developed and deployed a collection of free and subscription mobile applications to enrollees in a health system mobile device management platform, the challenges that arose throughout the project, and the effect on access to and utilization of the applications by medical students and clinicians.

Results:

Conclusion:

Keywords: mobile, smartphone, apps, clinical tools

Getting the Champagne out of the Bottle: Koha as a Local Authors Catalog

Fred King

Medical Librarian, MedStar Washington Hospital Center Washington, DC 20010, Washington, District of Columbia

Objectives: In addition to providing patient care, MedStar associates produce a substantial body of research and publications. The library has designed a local authors catalog using a modified version of the open-source ILS Koha and put it online. We hope that this project can serve as a low-cost model for other institutions wanting to track and publicize their research.

Methods: We modified Koha’s MARC bibliographic framework to accommodate new elements such as DOI and ORCID, and edited templates to display the new information to users. Medline tags were assigned existing or new MARC tags, and we designed MARC framework for posters and conference activities. The modified Koha catalog can display records for books, journal articles, and conference activities and can be searched by fields including author, subject, department, institution, MeSH term, and ORCID ID.

Results:

Conclusion:

Keywords: Koha, publications, publicity, authors, Medline, open-source, cataloging

Contact Us

Hours
Mon – Fri, 9am – 9pm ET
Phone
(877) 426 6323 (toll free)
(410) 638 9239 (direct)
Email
support@cadmiumcd.com

ADODB.Recordset error '800a0e7d'

The connection cannot be used to perform this operation. It is either closed or invalid in this context.

/conferenceportal2/includes/html/StickyFooter.asp, line 86
