# Supplementary material for: 117th Annual Meeting Medical Library Association, Inc. Seattle, WA May 26–31, 2017
Source: J Med Libr Assoc. 2018 Jan 2;106(1):E1–E28. doi: 10.5195/jmla.2018.421 (PMC5764600; doi:10.5195/jmla.2018.421)
Supplement: Appendix [file jmla-106-E1-s002.pdf]

Medical Library Association

MLA ’17 Poster Session Abstracts

Abstracts for the poster sessions are reviewed by members of the Medical Library Association National Program Committee (NPC), and designated NPC members make the final selection of posters to be presented at the annual meeting.

Poster Number: 1

Time: Sunday, May 28, 2017, 2:00 PM – 2:55 PM

Bioinformatics, Then and Now: Systematic Review 2005-2016, Findings and Future Prospects

Brian Oliver, PhD Student/Medical Librarian, Dominican University/Academy of Nutrition and Dietetics, Chicago, IL

Objectives: Based upon a review of developments in bioinformatics, I seek to propose the new directions of bioinformatics, which are in their infancy and on the horizon, e.g., modifications of domain analysis; algorithmically-focused data management; and, contextual data analysis, in order to best support scientific discovery and application efficiently and effectively.

Methods: I will provide a visual and narrative history of bioinformatics from its emergence to its recent multiform proliferation, with special attention on the ways it has evolved since 2005. I will also explore current trends and suggest best practices in bioinformatics, especially those that are applicable to bio/medical information professionals in their collaborations with researchers.

Results: Will be given during the poster session.

Conclusion: Will be given during the poster session.

Keywords: Bioinformatics, Literature Review, Systematic Review, Domain Analysis, Data Analysis

Poster Number: 2

Time: Monday, May 29, 2017, 2:30 PM – 3:25 PM

Bio-Retrospective: Fifteen Years as a Library-Based Molecular Biology Information Service

Carrie L. Iwema, Information Specialist in Molecular Biology; **Ansuman Chattopadhyay**, Head, Molecular Biology Information Service; Health Sciences Library System, Health Sciences Library System, Pittsburgh, PA

Purpose: Our health sciences library has provided bioinformatics-based support to researchers at a large biomedical institution since 2002. In the last fifteen years we have grown from a single-person fledgling program into a two-person multi-pronged enterprise. This poster highlights the evolution of the service, with a focus on trends in bioinformatics resources that have waxed and waned over this time period.

Description: Proficiency in the use of bioinformatics tools is the key for success in today's big-data intensive molecular life sciences research. Unfortunately the resources landscape changes at a rapid pace so it can be challenging for biomedical researchers to self-train and stay updated. To meet the increasing demand for bioinformatics assistance, we have taken a "4-pronged" approach: (1) Educational outreach—frequent departmental meetings and curriculum inclusion, and greatly fluctuating workshop attendance and topics, (2) Licensing of commercial software—types of tools and number of licenses are continually modified and registrations have increased, (3) Consultations—both face-to-face and virtual discussions have surged, and (4) Online resources—we offer a web portal, guides, listserv, and searching/research support innovations that reflect the changing needs of our researchers. To remain responsive, we continuously solicit feedback in person and via email, online forms, and post-workshop evaluations.

Keywords: bioinformatics, molecular biology, data analysis, research support, genomics

Poster Number: 4

Time: Tuesday, May 30, 2017, 2:00 PM – 2:55 PM

Do Direct-to-Consumer Genomics Services Put the ME in Medicine? Raising Awareness of Personalized Genomics for Patients and Physicians

Patricia F. Anderson, Emerging Technologies Informationist; **Marisa Conte**, Assistant Director, Research and Informatics; Taubman Health Sciences Library, Taubman Health Sciences Library, Ann Arbor, MI

Objectives: Personalized genomics are important to current clinical practice and emerging research initiatives in precision medicine. The appearance of direct-to-consumer (DTC) personal genomics services present new opportunities for patients, providers and researchers. These same resources can also complicate patient-provider relationships. To address this issue, we developed and conducted training sessions for patients and clinicians, and developed a suite of informational resources.

Methods: Developing this course required understanding the differing needs of patients and providers. For patients, we focused on instruction and exercises focused on patient goals: 1) Strengths and weaknesses of direct-to-consumer (DTC) personal genomics services; 2) Awareness of data formats, and tools available for analysing personal genomics reports and findings, 3) Awareness of major debates regarding DTC personal genomics risks/benefits; 4) Options and appropriate strategies for communication and clinical integration of patient-supplied data from DTC personal genomics services. For providers, we focused on helping physicians understand options for responding when a patient brings personal genomics findings to an appointment, including when to refer to a genetic counselor or clinical expert. In addition to describing our courses and information resources, we will provide assessment data. Finally, we will share future plans for library-based precision medicine / personal genomics services and resources.

Keywords: personal genomics, personal genetics, precision medicine, training, instruction, outreach, ethics, direct-to-consumer

Poster Number: 5

Time: Sunday, May 28, 2017, 2:00 PM – 2:55 PM

Implementing a Streamlined Resource Management Process in a Multistate Health Care Organization

Valerie J. Lawrence, AHIP, Consultant, Care Management Institute, Kaiser Permanante, Clinical Library, Oakland, CA; **Eve Melton, AHIP**, Regional Director, Northern California Libraries, Northern California Region, Kaiser Permanente, Stockton, CA; **Mary E. White**, Director for Library Services, National Pharmacy Program, Kaiser Permanente, Drug Information Services, Downey, CA

Objectives: In 2011, Kaiser Permanente (KP) libraries nationally shared a common web site and online catalog but were separated by organizational fragmentation, which resulted in uncoordinated purchasing of print and electronic resources. The objective was to implement organizational management of knowledge-based resources, develop a common collection development strategy, and improve users' access to resources.

Methods: The National Library Operations Group, which included librarians representing each of the regional stakeholder groups, investigated the issues surrounding coordinating collection management across a widely dispersed system and the potential benefits of moving to an all-digital collection. The group completed site visits to similar library organizations and interviews with staff in university and hospital libraries that had digitized their collections. The group collected and reviewed user population information, staff and user satisfaction with each institution's coordination efforts, and any impacts on those institutions' finances, staffing, and library space. They also reviewed internal KP financial and

organizational statistics pertaining to stakeholder populations, library staff, budgets, and amounts contributed to purchasing electronic and print resources. They then developed and implemented standardized usage assessment tools and metrics for resource usage across the system.

Results: The group created a project plan and assessment tools to focus on balanced purchase decisions that benefited both the organization and patrons. Subscription decisions were made based on resource quality, user demand, cost, availability, whether it supported KP goals and initiatives, and internal usage of the print version. Updated policies and guidelines supported the transition of services towards a digital library that is not confined by geography and is efficiently managed.

Although no net cost savings were realized in switching from print to all-electronic, reduced spending on print allowed the libraries to divert money to the purchase of e-journals. This led to reduced duplication of print in the libraries and allowed for better access to all of our resources. By 2017 all print subscriptions had been cancelled organization wide.

Conclusion: The work of the National Library Operations Group allowed us to provide increased access to our resources. Coordinating operations led to consolidating subscriptions, minimizing redundant materials, and eliminating print, which reduced routine tasks and freed up time for professional librarians to focus on providing high value clinical and research services. Increased electronic access allowed KP Libraries to better meet the information needs of clinicians and providers with the right information, at the right time, in the right place.

Keywords: collection development collaboration resource management

Poster Number: 6

Time: Monday, May 29, 2017, 2:30 PM – 3:25 PM

Scaling Change in Higher Education: Collection Management for New Academic Programs

Sophia N. Prisco, Librarian, Center for Graduate Studies, West Coast University, Los Angeles, CA

Objectives: This project describes an innovative approach to creating a collection proposal from budgeting to implementation as a graduate school of health sciences constructs a Doctor of Occupational Therapy (OTD) program. The unique emphasis on the use of standards is in contrast to collection management that relies heavily on faculty input, which may be nonexistent for departments in development.

Methods: Planning practices for the library collection begins as the new academic program considers the application process where concrete budget projections affect program feasibility. External professional organizations and accrediting bodies provide standards that are utilized to organize proposal components and also serve as evidence when communicating with administrators for support. The Association of Colleges and Research Libraries “Standards for Libraries in Higher Education” provides justification for the budget. “Institutional Learning Outcomes” from the parent institution guide collection development for print and electronic resources. And WASC Senior College and University Commission “Standards of Accreditation” guide implementation and assessment. Although this project is from the perspective of a small academic library (fewer than 1,000 FTE), it is intended for librarians of any level of expertise and at any size institution.

Keywords: collection management, collection development, collection proposal, curriculum expansion, planning, small academic libraries, budgeting, accreditation, feasibility studies

Poster Number: 8

Time: Tuesday, May 30, 2017, 2:00 PM – 2:55 PM

E-Book Package Subscription Model: Benefits for the Library or the Publishing Industry?

Jie Li, Assistance Director, Collection Management, Biomedical Library, University of South Alabama, Mobile, AL; **Geneva Staggs, AHIP**, Acting Director and Assistant Director for Hospital Library Services, University of South Alabama, Biomedical Library, Mobile, AL

Objectives: Traditionally, journals are sold in a subscription model as journals add new content in new issues and volumes multiple times a year. However, books are traditionally sold in a perpetual model as books do not have new content added until new editions come out. This usually happens every few years, although, some are updated more often, even annually. Since the advent of electronic journals and books, book publishers (some of them also being journal publishers) have followed the publication model of journals to sell e-books on a subscription model and grouping books into a package. The objective of this poster is to find out if the e-books package subscription model benefits the library and its users.

Methods: In this study, a major e-books database and specialty e-book databases are included. AssessMedicine (AM), AccessSurgery (AS), ClinicalKey (CK), LWW Health Library Premium Basic Sciences Collection (HL BS), LWW Health Library Clerkship & Clinical Rotations Collection (HL Clerkship) and LWW Health Library Surgery Collection (HL Surgery) analyzed. Publication years of e-books in packages and each book's age have been examined. For books with multiple editions, intervals of new editions are analyzed. Both usage of e-books by individual title and usage of each package are calculated to determine cost-effectiveness.

Results: Percentage of books published in 2014-2017: AM 60.18%, AS 59.38%, CK 34.37%, HL BS 25.00%, HL Clerkship 44.90% and HL Surgery 42.48%. Percentage of books published in 2011-2013: AM 31.86%, AS 18.75%, CK 36.49%, HL BS 54.17%, HL Clerkship 34.69% and HL Surgery 37.75%. Percentage of books from 2004-2010: AM 7.96%, AS 21.88%, CK 29.14%, HL BS 20.83%, HL Clerkship 20.41% and HL Surgery 19.58%. AM and HL Clerkship each have a 2004 book. CK has no book published before 2009. Not including first editions the average intervals of new edition publication: AM 3.39, AS 5.08, CK 7.23, HL BS 4.61, HL Clerkship 4.62 and HL Surgery 5.21. In one database 75% of titles were not used. Another two showed approximately 50% were heavily used.

Conclusion: Analysis of publication years, intervals of new editions, and book and package usage statistics show that the publishing industry benefits more than libraries by using the e-book package subscription model. Many titles are not used and inclusion of supplemental products does not benefit users on our campus.

Keywords: e-book, collection management

Poster Number: 9

Time: Sunday, May 28, 2017, 2:00 PM – 2:55 PM

Comparing E-Books across Different Platforms, Providers, and Devices

Emily K. Chan, Scholarly Communications Librarian, Dr. Martin Luther King Jr., Library, San Jose State University, San Jose, CA; **Susan L. Kendall, AHIP**, Education and Government Publications Librarian, Dr. Martin Luther King, Jr. Library, San Jose State University Library, San Jose, CA; **Mary H. Nino**, Reference and Instruction Librarian, San Jose City College, Cesar Chavez Library, San Jose, CA

Objectives: Identify the health sciences ebook publishers and platforms that offer a suite of functions to accommodate varying learning styles in a responsive, device-agnostic environment.

Methods: Using health science ebooks held at the San Jose State University Library, the authors evaluated eight ebook platforms on the basis of text adjustment, search/navigation, annotations, in-platform text-to-speech, dictionary support, printing, downloading and citing across different devices.

The ebooks were viewed on smartphones (Android and iOS), tablets (iPad and Kindle), and desktops (Windows and iOS). The authors used the appropriate browsers, based on the device.

Results: A sampling of health sciences ebook titles were evaluated for text adjustment, search/navigation, annotations, in-platform text-to-speech, dictionary support, printing, downloading, and citing across eight ebook platforms using different devices. Results from the analysis indicate that tools and ease of use varied greatly among the platforms, providers, and devices.

Conclusion: Aggregators and single publishers present and provide access to ebooks using different models with differing restrictions. Aggregator platforms, particularly EBL and ebrary, seek to provide a book-like experience, but force users to comply with highly restrictive digital rights management (DRM). Publishers, with their extensive journal holdings, tend to treat books like special issues, parsing books into downloadable chapters.

Keywords: Ebooks, Publishing, Accessibility, Health Sciences, Evaluation, Mobile Devices

Poster Number: 10

Time: Monday, May 29, 2017, 2:30 PM – 3:25 PM

2017 Nursing and Allied Health Resources Section Selected List of Physical Therapy Journals

Betsy Williams, Health Professions Liaison Librarian, Grand Valley State University, Frey Foundation Learning Center, Grand Rapids, MI; **Eileen G. Harrington**, Health & Life Sciences Librarian, Priddy Library, The Universities at Shady Grove, Rockville, MD; **Diane Kearney, AHIP**, Lead Medical Librarian, Northern Virginia Community College, Medical Education Campus Library, Springfield, VA; **Lea Leininger**, Health Sciences Librarian, University Libraries, Research, Outreach, and Instruction Department, Jackson Library, Greensboro, NC; **Michelle Rachal**, Reference & Web Librarian, University of Nevada, Reno School of Medicine, Savitt Medical Library, Reno, NV; **Jill Turner**, Associate Librarian, University of Detroit Mercy, School of Dentistry Library, Detroit, MI; **Ansley Stuart, AHIP**, Allied Health Sciences Information Librarian, Augusta University, Robert B. Greenblatt, M.D. Library, Augusta, GA

Objectives: The 2017 NAHRS Selected List of Physical Therapy Journals will help librarians with collection development and provide a useful resource to assist faculty in identifying options for publishing. Research in physical therapy is often published in interdisciplinary journals, and these are included in the list. The list compares database coverage and full text options for each title, including open access.

Methods: The team consists of one chair and seven NAHRS members. The 2012 NAHRS Selected List of Nursing Journals served as the foundation for the 2017 Selected List of Physical Therapy Journals. The initial list of journal titles was compiled from Ulrichsweb™, CINAHL, the NLM catalog, the Directory of Open Access Journals (DOAJ), and studies mapping the core journals of physical therapy. The team developed the following inclusion criteria:

• Peer reviewed

• Published in English or bilingual English/other

• Currently published with a print or electronic ISSN

• Indexed in a database

After resolving journal title changes and removing ceased titles, each team member verified journal information, database coverage, and full text access. The team will assess the usefulness of the list, possibly via page views, citations, or a survey. Evidence-based practice content may be included in the future.

Results: The journal project began in earnest in July, 2016, and journal information and database coverage was completed in March, 2017. The final list includes 236 titles. Of these, 60% are

interdisciplinary journals, included because they were identified as highly cited in mapping studies of the physical therapy literature. The project team chose to defer collection of evidence-based practice content data in order to hasten dissemination of the list, which will be published on the NAHRS website.

Conclusion: The 2017 NAHRS Selected List of Physical Therapy Journals was based on the methodology and format of the 2012/2016 NAHRS Selected List of Nursing Journals, with some modifications to reflect database coverage relevant to physical therapy. Using the nursing list as a model saved the team a considerable amount of time. We were also fortunate to have four studies mapping the physical therapy literature for reference. As in the case of the nursing journal list, the Selected List of Physical Therapy Journals combines important information all in one place, and will be useful in collection development and faculty publishing decisions.

Keywords: Physical therapy, allied health, collection development, publishing

Poster Number: 11

Time: Tuesday, May 30, 2017, 1:00 PM – 1:55 PM

An Analysis of Physician Assistant LibGuides: A Tool for Collection Development

Scott Y. Johnson, Education/Technology Librarian, Marshall B. Ketchum University, M.B. Ketchum Memorial Library, Fullerton, CA; **Catherine V. Johnson**, Librarian, Research and Instruction, Assistant Professor, University of La Verne, Wilson Library, La Verne, CA

Objectives: Objectives: The physician assistant (PA) specialty encompasses many subject areas and requires many types of library resources. An analysis of PA LibGuides was performed to determine most frequently recommended resources.

Methods: Methodology: A sample of LibGuides from U.S. institutions accredited by the Accreditation Review Commission on Education for the Physician Assistant (ARC-PA) was included in this study. To generate a list of qualifying LibGuides, a keyword search for "physician assistant" on the Springshare LibGuides Community site (http://community.libguides.com/) was performed. Results were filtered by Library Type by selecting Academic Institution. This retrieved 570 guides. These guides were compared to a list of ARC-PA accredited institutions obtained from the ARC-PA website (http://www.arc-pa.org/). This resulted in 68 guides from accredited U.S. institutions. A random number generator in Excel was used to select 45 guides for analysis. Resources presented on guides were tabulated and organized by resource type.

Results: Databases and point-of-care tools had resources that were listed by the most libguides, including Pubmed (40), CINAHL (35), Cochrane Library (32), AccessMedicine (28), MEDLINE (23), UpToDate (22), and ClinicalKey (21). There were over 1,000 books listed on the 45 guides, including over 600 unique books listed. Harrison's Principles of Internal Medicine (18) was the most common. There were fewer journals listed, 163, with JAAPA (18), JAMA (12), and NEJM (11) the most popular. Anatomy.tv (13) and Exam Master (13) were the most frequently listed video database and certification resource, respectively.

Conclusion: Overall, while the 45 LibGuides evaluated list many unique resources in each category, a librarian can create an accepted list of the most frequently listed resources from the data gathered.

Keywords: physician assistant, LibGuides, collection development, guides, resources

Poster Number: 12

Time: Tuesday, May 30, 2017, 1:00 PM – 1:55 PM

Do We Dare to Discard? Retention of Print Serials in a Digital Age

Annie M. Thompson, Director; **Cari Lyle**, Library Supervisor; **Matthew Codey**, Serials Assistant; **Rebecca Davis**, Information Services Librarian; Wilson Dental Library, Los Angeles, CA

Objectives: Retention of print collections is an ongoing concern for health sciences libraries in the digital age. This poster will provide a snapshot of health sciences libraries' current practices regarding print serials with the hope of informing collection-related decision-makers of the benefits or consequences of withdrawal versus retention.

Methods: The current literature does not present clear data regarding of health sciences libraries' practices related to print serial subscriptions. The authors plan to perform a thorough literature review as well as collect data via a survey instrument that will be sent to health sciences technical services listservs. The authors would like to address the benefits or consequences of print retention versus moving to an e-only environment; specifically focusing on: user accessibility, financial impact, and use of physical space in the world of 'library as place'. Overall, the goal of this research hopes to guide technical services professionals, those in collection development positions, as well as library directors in the decision-making process related to print serial subscriptions.

Results: Results will be presented during the MLA 2017 poster session. Data collection and analysis is ongoing.

Conclusion: Conclusion will be determined prior to the MLA 2017 poster session.

Keywords: serials, print serials, retention, collection development, technical services, survey, digital versus print, literature review, library directors, decision-making

Poster Number: 13

Time: Sunday, May 28, 2017, 2:00 PM – 2:55 PM

Reflections on the First Five Years of Outreach Initiatives at an Emerging Medical Library

Stephanie Swanberg, AHIP, Assistant Professor, Information Literacy & eLearning Librarian; **Keith Engwall, AHIP**, Assistant Professor, Web & Emerging Technologies Librarian; **Misa Mi, AHIP**, Associate Professor, Medical Librarian; Oakland University William Beaumont School of Medicine, Rochester, MI

Objective: When starting a new academic medical library, certain areas take priority including hiring staff, building collections, and integrating instruction into the curriculum. Outreach is usually saved until libraries are more established. We took a different approach. This poster discusses the successes and lessons learned of implementing a comprehensive outreach program during the first five years of a new medical library.

Methods: As part of a medical school that highly values community engagement, the Oakland University William Beaumont School of Medicine (OUWB) Medical Library decided early in its development that it wanted to prioritize that aspect of its mission. Though starting informally, the library's outreach program has matured and now sustains a wide array of activities each year targeting the campus and local community. Outreach initiatives include: promoting reliable health information at community health fairs; participating in youth summer enrichment programs; partnering with student organizations to co-host events, such as fundraisers and bone marrow donor registration drives; hosting medicine-themed exhibits; and conducting a needs assessment project to discover the health information needs of underserved and vulnerable populations in a homeless shelter. Other libraries considering engagement programs could adapt these strategies to contribute to the educational mission of their home institutions.

Results: The OUWB Medical Library has taken a three-prong approach to outreach activities: 1) participate in existing school-sponsored activities; 2) partner with medical student organizations to host programs; and 3) independently coordinate activities, including hosting 12 NLM traveling exhibits. As

such, since 2011, we have participated in 5 summer programs for high school students and volunteered at 9 community health fairs, all sponsored by the medical school. Results of the needs assessment project have been used to tailor the materials brought to these fairs, such as oral health information. In addition, we've partnered with medical student organizations to co-host 3 gaming fundraisers for a local hospital and 5 organ & bone marrow donor registration drives. This approach has proved successful in sustaining such a high level of activity without straining staff, time, or budget. The ongoing challenge is assessing the impact of these activities beyond attendance, registration, or fundraising metrics.

Conclusion: Through these outreach initiatives, the library has promoted the unique role that medical librarians can serve in educating the community about quality health information as well as meaningfully contribute to the mission of the medical school without draining valuable resources.

Keywords: outreach, community engagement, strategic planning, program development

Poster Number: 14

Time: Monday, May 29, 2017, 2:30 PM – 3:25 PM

Health Behavior Changes Brought upon by a Blood Pressure Kiosk in a Health Library

Edgar Lopez, Librarian, Stanford Health Library, Palo Alto, CA

Objectives: How does a public-access blood pressure kiosk located in a health library based at a low-income clinic impact the health behavior and library usage of the general population and staff?

Methods: Focus groups and interviews were held by the librarian to document how the blood pressure kiosk impacted the general community and staff. The setting is a clinic located in a low-income area that serves a tri-lingual population of African-Americans, Hispanics, and Pacific Islanders. The health library includes one full-time librarian. Earlier this year the American Heart Association offered the clinic a free blood pressure kiosk. The AHA also offered training to the health coaches. the CMO of the clinic also included the librarian to be part of the training. The goal was for the health coaches to identify 100 high-risk patients and for the patients to check their blood pressure numbers every two weeks for a period of four months. Workshops were also held in English/Spanish and Tongan.

Results: Since the inclusion of the blood pressure kiosk in the health library, blood pressure inquires have increased both among the general public and the staff.

Conclusion: Health behavior changes have varied among both the staff and the general population. Results an conclusions will be made available during MA '17

Keywords: blood pressure, low-income, health library, African-American, Hispanic, Pacific Islanders

Poster Number: 15

Time: Tuesday, May 30, 2017, 1:00 PM – 1:55 PM

San Francisco Plague of 1900-1904: Economics, Politics, and Racism

Merle Rosenzweig, Informationist, Taubman Health Sciences Library, Taubman Health Sciences Library, Ann Arbor, MI; **Anna Cupito**, Graduate Student, University of Michigan, Taubman Health Sciencs Library, Ann Arbor, MI; **Elise Wescom**, Media Assistant/Graphic Artist, University of Michigan, Taubman Health Sciencs Library, Ann Arbor, MI; **Chase Masters**, Enabling Technologies Informationist, Taubman Health Sciences Library, University of Michigan–Ann Arbor

Objectives: Before 1900 the United States was free of the Bubonic Plague. That changed when a ship arrived in San Francisco from Hong Kong in 1899 carrying two cases of plague on board, starting an

epidemic in the city. This poster follows the business, political and racist factors that influenced the denial of the presence of the plague, allowing its spread.

Methods: The authors gathered the information for this poster from the papers of the members of the San Francisco Plague Commission—Frederick G. Novy (housed at the Bentley Historical Library), Simon Flexner (housed at the American Philosophical Society), and Lewellys F. Barker (at the Chesney Medical Archives) —and Joseph Kinyoun, the chief quarantine officer of the San Francisco Marine Hospital Service who went on to become the first director of the Hygienic Laboratory, forerunner of the NIH. His papers are housed at the National Library of Medicine. We also gathered newspaper stories of The Plague from Chronicling America: American Historic Newspapers digital collection at the Library of Congress.

Results: The efforts to combat the plague epidemic in San Francisco in the 1900s were hampered by the interference of powerful business interests, by the local newspapers, and by political actors. This meant the efforts of public health officials were mocked, and the containment strategies were hamstrung by inefficient implementation. Public dissemination of information about disease was notably absent, funding for containment efforts was minimal, and the primarily affected Chinese community in San Francisco actively resisted quarantine efforts. This allowed the plague to spread for years, killing hundreds and creating a breeding reservoir in the ground squirrel population, which continues today.

Conclusion: Parallels can be seen in the response to the HIV/AIDS epidemic in the same city, 80 years later. After initial discovery of a cluster of rare cancers in homosexual men, and even after the discovery of the virus causing the disease, the political response was slow, funding for research was deemed inadequate, and members of the affected community fought the preventative measures designed to contain it. It calls to mind the old adage: those who do not learn from history are doomed to repeat it.

Keywords: Bubonic plague, Economics, Politics, Racism

Poster Number: 16

Time: Tuesday, May 30, 2017, 2:00 PM – 2:55 PM

Food Allergy Awareness and Education: Opportunities for Librarian Community Engagement

Helen Look, Collection Analyst, University Library, University of Michigan Library, Ann Arbor, MI

Objectives: To examine how the skills of librarianship can be applied towards increased community engagement in support of individuals and families with food allergies.

Methods: Librarians can play a critical role in their communities by supporting increased awareness and understanding of health conditions, such as food allergies. This poster session will explore different opportunities of community engagement to support food allergy related needs of local community members. Librarians can serve as a resource for local support groups in connecting the public to reliable health information. They can also partner with advocacy organizations to develop guidelines to be used by support group leaders nationwide for evaluating health information on the web. Librarians can also encourage authors or publishers to provide free or discounted food allergy related books to local schools and libraries to promote awareness. Librarians can also engage in a community needs assessment by working with an interdisciplinary community engagement council (that includes physicians, school nurses, lawyers, educators, students, and food allergy families) to improve community understanding and to provide support for those affected by food allergies.

Conclusion:

Results will be made available during the poster session at MLA ’17.

Keywords: food allergies, community engagement, advocacy, collaboration, consumer health

Poster Number: 17

Time: Sunday, May 28, 2017, 2:00 PM – 2:55 PM

Health and Wellness To Go: A Community Collaborative

Barbara Platts, AHIP, Manager, Knowledge Management Services, Munson Healthcare, Traverse City, MI

Objectives: The intent of the Health & Wellness To Go program is to strengthen partnerships with community-based organizations as well as reach out to specific populations. The program is designed to promote the role of the health information professional as well as the Community Health Library in the delivery of health information. The program is intended to support the health system's strategic priority surrounding population health. The success of this program will be based on obtaining identified outcome measures.

The program provides bundled health information, in a bag, to community members in the health system's service area. Bags contain a variety of health resources related to diseases/conditions that are prevalent in the region.

Methods: The program provides bundled health information, in a bag, to community members in the health system's service area. Bags contain a variety of health resources related to diseases/conditions that are prevalent in the region. Disease/condition baseline data was obtained by consulting the regional Community Health Needs Assessment, Healthy People 2020, and the Community Health Library operating statistics.

Phase I of Project:

1. Researched program models
2. Evaluated ability to replicate and customize program models
3. Developed a project plan
4. Identified community partner(s)
5. Solicited feedback from stakeholders
6. Identified diseases/conditions prevalent in region
7. Identified needed resources in order to implement program
8. Developed budget
9. Developed a project time line

Phase II of Project:

1. Identified internal and external sources of funding
2. Examined funding requirements including fiduciary compliance and reporting
3. Determined sustainability of project
4. Applied for a NLM Community Engagement Award
5. Received award from NLM
6. Accepted contract from NLM

Phase III of Project:

1. Program Development

Phase IV of Project:

1. Program implementation

Results: Outcome measures are analyzed on a quarterly basis.

Conclusion: Based on feedback from stakeholders, the program was expanded to included additional disease/condition resources.

Keywords: community health, community collaborative, population health, community-based organizations, wellness

Poster Number: 18

Time: Sunday, May 28, 2017, 2:00 PM – 2:55 PM

Gathering Expert Panel Recommendations for the Design of a Consumer Environmental Health Resource

Alla Keselman, Senior Social Science Analyst; **Karen Matzkin**, Outreach Specialist for NLM; **Judy Kramer**, Public Health Specialist; **Andrew Plumer**, Information Specialist; **Janice E. Kelly, FMLA**, Acting Deputy Associate Director; Specialized Information Services, Bethesda, MD

Objectives: This work reports findings from two expert panels tasked with helping National Library of Medicine (NLM) develop an environmental health and toxicology consumer health information resource. The objective of the panel was to identify environmental health information needs of lay individuals and community organizations and help design information architecture addressing those needs.

Methods: NLM maintains a number of environmental health and toxicology resources for different populations, including Tox Town, a portal about potentially toxic chemicals in everyday places for the public. A recent usability study and a review of departmental priorities suggested redesigning or supplementing Tox Town, making it more responsive to information needs of specific user groups. To develop specifications for the resource, NLM assembled two panels comprised of environmental health and information professionals working with the public and community organizations. The experts represented federal organizations, libraries, academic institutions, and consumer and environmental health advocacy organizations. Questions addressed environmental health information needs of various communities; concerns unique to different geographic areas and populations; characteristics of desired information and information resources; roles in which consumers seek environmental health information; and more. Results are to inform resource development.

Results: Experts suggested that an environmental health resource for consumers should organize information in multiple ways (e.g., by chemicals, health conditions, routes of exposure), include local data / resources / statistics, and provide actionable information. It should also translate complex information into consumer-friendly language.

Conclusion: Tox Town is a useful consumer and community resources that can be further improved with expert panels' recommendations.

Keywords: Community health, environmental health, expert panel, general public, online portals, toxicology

Poster Number: 19

Time: Tuesday, May 30, 2017, 1:00 PM – 1:55 PM

Academic Health Sciences Library Partners with Local Public Library to Improve Community Health

Yingting Zhang, AHIP, Research Services Librarian, Rutgers University, Robert Wood Johnson Library of the Health Sciences, New Brunswick, NJ; **Karen T. Parry**, Manager of Information Services, East Brunswick Public Library, Information Services, East Brunswick, NJ

Objectives: This poster is to present the collaboration between an academic health sciences librarian and a public librarian in contributing to the development of 2016 CHIP as mandated for nonprofit

hospitals every three years. The ultimate goal is to encourage more academic health sciences librarians to reach out to public librarians to seek joint solutions to improve community health.

Methods: Studies show that low health literacy is associated with poor health outcomes, primarily among at-risk populations that includes the poor, elderly, and new immigrants. Librarians are natural crusaders in the fight to promote better health awareness in their communities. Librarians offer research, analytical, and social skills supplemented by a ready-made infrastructure that taps into a vast network of community resources. Libraries provide health information resources, offer training opportunities, and partner with local health organizations and providers to advance health literacy. Academic and public health sciences librarians were invited to serve on the RWJUH and SPUH CHIP Cultural Competency/Health Literacy Working Group and participated in the 2016 CHIP Planning Session for the Community Health Consortium for Central Jersey (CHCCJ). A symposium is being organized to train public librarians to provide consumer health information services to their local communities.

Results: The Academic and Public Librarians found synergies to address Priority One of the 2016 CHIP. The academic health sciences librarian advocated for health literacy by introducing reliable consumer health resources, authoring a Wikipedia article on cultural competence in health care, and conducting a related research study. The public librarian developed a community health literacy program called Just for the Health of It that uses trained consumer health librarians to provide custom health research, mobile outreach, etc. The librarians identified commonalities and ways to share and leverage staff expertise, knowledge and resources to improve access to care in the 2016 CHIP.

Conclusion: Academic and public libraries who serve the health information needs of healthcare providers, students, and the community ought to seek collaboration rather than operate in discrete silos. Partnerships between academic health sciences and public librarians should be advocated, encouraged, and supported. Shared knowledge between academic and public libraries will improve the quality and breadth of critical health information and resources available to the community at-large and will be a catalyst to break down barriers to equitable health among at-risk populations.

Keywords: Community Health, Health Literacy, Cultural Competency, Culturally Sensitive Services, Cultural Competence in Health Care

Poster Number: 20

Time: Tuesday, May 30, 2017, 2:00 PM – 2:55 PM

Delivering Continuing Education to Rural and Frontier Nurses Using a Course Management System

Katie Jefferson, Library Services Liaison; **Michelle Rachal**, Reference & Web Librarian; **Mary Shultz**, Director; University of Nevada, Reno School of Medicine, Savitt Medical Library, Reno, NV

Objectives: The Savitt Medical Library at the University of Nevada, Reno was granted a Cooperative Agreement with the Pacific Southwest Region of the National Network of Libraries of Medicine. The agreement is to provide instruction and support for evidence-based information through in-person and online instruction to community health nursing clinics across the State of Nevada.

Methods: All participating clinic nurses were interviewed and/or surveyed on their current use of information resources and their current needs. From those results, a program of both in-person and remote instruction was developed which includes 1-2 site visits per clinic and 12 online modules.

Each module will offer 1 unit of Continuing Education credit. The series of modules will cover a variety of topics including: infectious disease information resources; mental health resources, insurance; immigrant/refugee health resources; rural & Native American resources; disaster preparedness, etc. The modules will be offered through a Course Management System (Canvas). Each module will include video instruction, readings, and assignments.

Results: Based on the initial needs assessment, the first in-person CE session was designed to cover the project portal, MedlinePlus, Health Reach and other language sources, Google Scholar, and advanced Google search functions. The evaluations of these sessions showed all participants felt comfortable using the resources and all said they will make changes in their practice to incorporate using information resources. Of the open-ended responses, the nurses indicated they would change their practice to: pass this information along to co-workers, social workers, and counselors; direct patients to quality information sources, and provide patient handouts; and that their search techniques would improve.

Conclusion: Based on the needs assessment and site visits, a series of 10 online modules using the Canvas Course Management System is under construction. The sessions have been approved for Nursing Continuing Education (CE) credits and will be available for at least one year. All modules will be released by May 2017. The librarians noted a high level of nurse engagement and interest in suggesting not only links for the portal but needed course content. The site visits allowed the team to see how and where the nurses worked and what type of resources and access were available.

Keywords: Community, behavioral, health, nurse, rural, frontier, instruction, Nevada, continuing education

Poster Number: 21

Time: Sunday, May 28, 2017, 2:00 PM – 2:55 PM

Community Access to Health Information Training in San Diego

Naomi C. Broering, AHIP, FMLA, Dean, Libraries, Medical Library, Pacific College of Oriental Medicine, La Jolla, CA; **Gregory Chauncey, BSEE, MBA**, Health Information Instructor and Program Mgr., Pacific College of Oriental Medicine, Medical Library, San Diego, CA

Objectives: In 2016, the Pacific College of Oriental College (PCOM) received 9 month funding from the NN/LM PSR to launch a collaborative health information outreach project with eight San Diego organizations including public libraries, churches, community centers and affiliated clinics to provide training workshops for consumers and health professionals. The Goals are to increase awareness and access to NLM/NIH online resources through training workshops for all residents, with emphasis on reaching multicultural, diverse and minority populations at their communities, and to use the latest technologies to conduct the project. The Objectives are to conduct instructional workshops directly at partnering institutions located in the target residential areas and teach attendees how to access the information they need, search health topics from MedlinePlus, Pub Med, and Clinical Trials sources to improve communication with health practitioners, and acquire knowledge to lead healthier lives. Flexible workshop strategies meet attendees' needs and interests, such as bilingual sessions, interactive sessions, mobile tools, power point slides, live searching using iPad, WiFi, smartphones, and demonstrations at professional meetings and clinical settings for interns and patients. Handouts are provided

Methods: The medical library created a community outreach environment with the goal of informing local residents how to improve their health and health care by accessing authoritative information using the NLM and authenticated databases. Methods include: 1. Initiating partnerships with senior centers, church-based organizations, public libraries, affiliated clinics to provide computer workshops, and conducting demonstrations at the Pacific Symposium and Successful Aging Independence conferences. 2. Instructing attendees (consumers, health practitioners, clinic patients) to find evidenced-based medical information on various health conditions. 3. Emphasizing use of MedlinePlus, Pub Med, and Clinical Trials as primary resources and access to the PCOM Library's updated webpage. 4. Featuring a unique range of topics including western and integrative medicine, drug information, public health, and emergency preparedness. Publicity to attract attendees used includes newspapers, brochures, fliers and posters

Results: Over 30 workshops and two conference sessions for 2,150 diverse attendees in several San Diego county neighborhoods were provided.The poster includes images of project objectives, impact, and workshop locations plus analysis of evaluation questionnaires.

Conclusion: The poster includes images of project objectives, impact, and work shop locations. Impact and results will be provided.

Keywords: Consumer Health, Outreach Programs, Information Literacy, Mobile Resources, WiFI access, Integrative Medicine, MedlinePlus, Pub Med, Multiple language source

Poster Number: 22

Time: Monday, May 29, 2017, 2:30 PM – 3:25 PM

Culinary Medicine: Health Professionals Collaborating to Bring Health and Nutrition to the Community

Trey Lemley, AHIP, Information Services Librarian, Biomedical Library, Biomedical Library, Mobile, AL; **Margaret Sullivan**, Deputy Director of Business Development and Outreach, University of South Alabama, Mitchell Cancer Institute, Mobile, AL; **Austin Cadden**, Associate Manager, Epidemiology, Cancer Control, and Population Health, University of South Alabama, Mitchell Cancer Institute, Mobile, AL; **Rachel Fenske**, Information Services and Outreach Librarian, University of South Alabama, Biomedical Library, Mobile, AL

Objectives: This research describes the development of a culinary medicine program in which health professionals (including librarians) and social service agencies connect with underserved populations to teach the basics of good nutrition and healthy lifestyle choices, with the goal of improving the health of these populations. In the process, librarians build partnerships for community engagement and outreach.

Methods: Setting: A multi-campus academic medical center with a medical library, college of medicine, college of nursing, and college of allied health. Classes take place in the teaching kitchen of a local community college, and in addition, participants make field trips to the grocery store to learn to shop for nutritious and affordable food. Population: Public health professionals, social workers, librarians, physicians, nurses, medical/nursing students, and members of the community referred by social service agencies. Research design: In this program, librarians partner with a broad range of health professionals and program participants to accomplish the stated goals of healthier living through healthier eating. In particular, librarians provide appropriate, relevant health information and perform literature searches. In addition, librarians introduce participants to free, health-related governmental websites, including MedlinePlus.gov and Nutrition.gov. Assessment will be made by questionnaire at the end of each module.

Conclusions: Results and Conclusions will be made available at my MLA '17 poster onsite.

Keywords: Culinary Medicine

Health Disparities

Community Engagement

Consumer Health

Outreach

Poster Number: 23

Time: Tuesday, May 30, 2017, 1:00 PM – 1:55 PM

Strategies for Reducing Suicide: A Collaboration in Washington State

Patricia J. Devine, Outreach & Communications Coordinator, Health Sciences Library, Pacific NW Region, Seattle, WA

Objectives: To demonstrate how a collaboration between a medical librarian and a State Medical Quality Assurance Commissioner did the following:

- Educated healthcare providers about State Department of Health Suicide Prevention Continuing Education Requirements.
- Identified tools for screening at-risk patients.
- Demonstrated suicide prevention techniques
- Provided resources for patient and family education and support.

Methods: To better understand the information and education needs of the population of medical providers served, knowledge of the regulatory climate was sought. State Medical Commissions are educational as well as disciplinary bodies, with a mission of promoting patient safety and enhancing the integrity of the professions through licensing, discipline, rule-making, and education. Suicide rates in the United State have risen since 1999, with certain vulnerable and at risk populations having higher rates. How can librarians help the medical providers they serve address this crisis? Understanding the laws, CME requirements and the rule-making the State Medical Commissions positions us to provide a valuable service. This project describes the collaboration and makes recommendations.

Keywords: collaboration, healthcare, providers, CME, legislation, suicide prevention

Poster Number: 24

Time: Tuesday, May 30, 2017, 1:00 PM – 1:55 PM

We're Way Past Peas: Supporting Genetic Information Needs of Clinicians and Consumers

Carolyn Martin, AHIP, Consumer Health Coordinator, University of Washington, NN/LM Pacific Northwest Region, Seattle, WA; **Diana Nelson. Louden,** Biomedical and Translational Sciences Librarian, Health Sciences Library, University of Washington–Seattle

Objectives: With the growing availability of genetic information for clinicians and consumers, new segments of the population want to know how to locate and interpret genetic information. Accordingly, more diverse groups of librarians will want to connect their users with suitable genetic information resources. A consumer health coordinator and a biomedical sciences librarian are collaborating to prepare librarians for this work.

Methods: Two librarians developed an MLA CE-approved course and then identified in-person and online venues for providing training to librarians. By including an accessible introduction to the science of genetics in relation to human health, the course would be appropriate for a broad audience. The course was designed to engage attendees by describing uses of genetic information in health care; challenges and opportunities in clinical and direct-to-consumer genetic testing; and societal issues pertaining to genetic information. Attendees would then learn how the MedGen portal, GeneReviews, Genetics Home Reference, and other resources could be used effectively by clinicians, consumers, and librarians

Results: The course "We're Way Past Peas: Uses of Genetic Information to Understand Human Health and Guide Health Care Decision Making" was taught in person at state and regional medical librarian conferences in 2016, and it is being offered online in March 2017 to a national audience through the National Networks of Libraries of Medicine. Attendees have included hospital, public, and academic health sciences librarians, as well as librarians from health content providers and technology transfer offices. Interestingly, although NCBI created the MedGen portal in 2012, none of the in-person course attendees were familiar with MedGen prior to the course.

Conclusion: Increasing the genetic literacy of librarians will contribute to the genetic literacy of the consumers and clinicians they support. Familiarity with the utility and effective use of key genetic information resources will equip librarians to identify relevant, reliable information on the intersections between genetics and health.

Keywords: genetics, continuing education, health literacy, consumer health, NCBI resources

Poster Number: 25

Time: Sunday, May 28, 2017, 2:00 PM – 2:55 PM

Working with Community Partners to Develop Locally Relevant HIV/AIDS Video Resources

Nina C. Stoyan-Rosenzweig, Senior Associate in Libraries, Health Science Center Libraries, Health Science Center Library, Gainesville, FL; **Matthew Daley,** IT Expert, University of Florida, Health Science Center Library, Gainesville, FL; **Hannah F. Norton, AHIP,** Interim Fackler Director, Associate University Librarian, Health Science Center Library, Biomedical and Health Information Services, University of Florida–Gainesville; **Margaret E. Ansell, AHIP,** Nursing & Consumer Health Librarian, Health Science Center Libraries, University of Florida–Gainesville; **Ariel Pomputius, AHIP,** Health Sciences Liaison Librarian, University of Florida, Health Science Center Library, Gainesville, FL; **Michele R. Tennant, AHIP,** Interim Fackler Director, Health Science Center Library, Biomedical and Health Information Services, Health Science Center Libraries, Gainesville, FL; **Gretchen Kuntz,** Director, Borland Library, Jacksonville, FL

Objectives: As part of a HIV/AIDS Information Outreach Project funded by the National Library of Medicine, the project team at the University of Florida Health Science Center Libraries, in collaboration with the Center for the Arts in Medicine and community groups developed 4 videos targeted to at-risk groups that focused on the importance of HIV/AIDS testing, prevention, risk factors, and living with HIV.

Methods: Librarians at the UF Health Science Center Libraries, in collaboration with Arts faculty, developed videos based on discussion with local groups on needs for information among at-risk populations. During the project, the team utilized methods to assess the populations at greatest risk and determined methods to reach these groups, which included university students, low-income groups utilizing free services, seniors, and individuals living with HIV/AIDS. The team worked with arts-in-medicine faculty who wrote the scripts, recruited actors among local community organizations and directed the videos. Library staff also filmed and edited the videos. Scripts were evaluated by experts on scientific information, and communicating health messages, while a medical student who received funding for research to evaluate the videos. Videos were uploaded to the library youtube channel and further disseminated by community partners and other interested parties, with provision for continuing video evaluation through embedded surveys.

Results: The videos were completed and placed on the youtube channel, and team members developed a plan to disseminate the videos to community partners, health center administrators, interested faculty and a wider audience. In addition, a medical student researcher began the process of using focus groups to evaluate the impact of videos on the target groups.

Conclusion: All video viewers expressed satisfaction with the resultant materials, and evaluation work shows both that there is a strong market for the videos, and that, in order to provide effective educational resources, there is a vital need to produce highly targeted videos.

Keywords: HIV/AIDS, videos, at-risk groups, community partners

Poster Number: 26

Time: Monday, May 29, 2017, 2:30 PM – 3:25 PM

Creating New Opportunities for Inpatient Outreach

Meghan D. Evans, Community Health Librarian, Community Health Library, Danville, PA

Objectives: The purpose of this program is to create new opportunities for inpatients, or their family members, to actively seek disease focused and other health information during their hospital stay from the Community Health Library (CHL).

Methods: To better reach the inpatient population, CHL collaborated with an in house technology team that had recently launched a program to assign iPads to patient rooms. These iPads include a link to the CHL webpage with links to consumer health information databases. A secure request form is available if patients or family members wish to have the librarian research a specific topic. Results are delivered to the patient/family member within 24-hours. The CHL librarian rounds to inpatient rooms, introduces the library, its services and how to utilize the iPads.

Qualitative surveys are used to assess patients' or family members' experience utilizing iPads to learn more about their condition, find out about tests, and make requests electronically. Quantitative data is gathered regarding the number of interactions during rounding and the number of health information requests CHL received.

Results: Will be made available durring the poster presentation at MLA'17.

Conclusion: Will be made available durring the poster presentation at MLA'17.

Keywords: Inpatient, technology, consumer health information, embedded librarian

Poster Number: 27

Time: Tuesday, May 30, 2017, 1:00 PM – 1:55 PM

Dreaming, Daring, Doing: Creation of Relaxation and Patient Education Stations in a Hospital

Dana D. Kopp, AHIP, Medical Librarian, Providence System Library Services, Missoula, MT

Objectives: To replace an obsolete VHS-based patient education system and to support noise coverage, and sleep and patient education initiatives in the hospital, the Librarian developed and licensed programming for two stations to run on the hospital's closed cable system via high definition media players. The development, challenges and lessons learned will be discussed.

Methods: The Relaxation & Patient Education Stations Project, funded in part by a NN/LM Pacific Northwest Region Pilot Project Award was designed to tackle two significant issues in hospital patient care: noise and patient education, plus provide the opportunity to promote library services. Research shows that while patient education has long been known to improve outcomes, noise negatively impacts sleep, increases stress, and is detrimental to healing. Anticipating the failure of an antiquated patient education system, the Medical Librarian developed a modern system that retained local programming control, did not require major upgrades in phone or infrastructure technologies and had a more acceptable cost compared to packaged systems. Two TV channels were developed by using high definition digital media players to send the programming through filters into the hospital's closed circuit cable TV system. The Librarian was responsible for licensing, digitizing, scheduling and uploading the programming which had been selected with input from clinical staff and specialty physicians.

Results: The two channels have been running for nearly 5 years. Schedules for both stations are based on a continual play configuration with 24 hours of programming repeating daily. Relaxation Station programming is 24 hours of music or natural sounds with videos of animals, fish or scenery or narrated guided relaxation. The Librarian was able to create videos with hospital-licensed photographs, licensed music/sounds and basic Microsoft Movie Maker™ software. The Patient Education Station plays more than 70 licensed patient education, cardiac pre- and post-surgery and locally produced videos during

the normal waking hours. At night, the station runs as a black screen with the continuous sound of a fan for noise coverage or other sleep needs. Library marketing and patient reminder messaging appeared between programs as well as on a TV Channel Guide placed in the rooms. Facilities engineering and biomedical technicians provided assistance with cable filters and troubleshooting. Challenges included estimation of the time needed to make videos and failure of the original media player to run consistently.

Conclusion: A hospital librarian can create a CCTV system to provide relaxation, sleep support and patient education to patients.

Keywords: Patient Education, Closed--circuit television, Sleep, Relaxation

Poster Number: 28

Time: Tuesday, May 30, 2017, 2:00 PM – 2:55 PM

Preparing Our Library for Scientific Wellness-Based Medicine

Isaac Huffman, AHIP, Library Director, City of Mount Vernon, WA, Olympia, WA; **Carrie Grinstead**, Medical Librarian for Southern California, Providence Health & Services, Library Services, Burbank, CA

Objectives: Fueled by big data and genomics projects such as the Precision Medicine Initiative, a new form of medical practice called Scientific Wellness is emerging. Scientific Wellness leverages personal data clouds to promote individualized care, identify disease in pre-clinical stages, and engage consumers. We aimed to better prepare our library staff to support this new form of medicine.

Methods: Working with our Scientific Wellness partners, we conducted a gap analysis and literature review to identify the knowledge and competencies librarians need to support wellness-based medicine. Critical needs include knowledge of informatics, data analysis, Bayesian statistics, and consumer health behavior. We found that these skills were not fully represented among our library staff and developed a two-pronged approach to address the gaps. First, a data residency was developed, in which a librarian joined a research team and will participate in all stages of a data-driven project, including literature search, protocol development, data collection, and data analysis. Second, our staff is undergoing consumer health education, which will consist of at least six hours of course work. Preliminary analysis of this strategy was conducted via documentation of new skills in our first program participants.

Results: One librarian was able to join a research project investigating two interventions to reduce patient no-show rates in outpatient clinics in Washington. The librarian participated in editing the study protocol, developed specifications for intervention monitoring reports, and wrote standard operating procedures for data management. In preparation for data analysis, the librarian has engaged in the self-directed learning of inferential statistics using R.

The same librarian completed a graduate-level course in consumer health informatics, gaining knowledge in emerging technologies, consumer engagement, and privacy and data security.

Conclusion: The data residency has allowed a member of our staff to build relationships beyond our traditional patron base and to gain competence in research design, data management, and statistical analysis. The residency has been conducted within our organization and was, therefore, a low-cost method for developing expertise within our staff. Further, because of our team-based library model, the librarian was able to devote time to the research project without compromising the standard level of service.

Next steps include sharing knowledge gained through both the data residency and consumer health education training with the rest of the library team. Consumer health service lines, such as information prescription programs, will be considered.

Keyword: Scientific Wellness, Precision Medicine, Data Libraries, Consumer health education

Poster Number: 29

Time: Sunday, May 28, 2017, 2:00 PM – 2:55 PM

Health Education for South Florida Transgender Women: The Florida International University Herbert Wertheim College of Medicine Medical Library Outreach Project

Francisco J. Fajardo, Clinical Engagement Librarian, Florida International University, Florida International University Medical Library, Miami, FL; **Hector R. Perez-Gilbe, AHIP**, Research Librarian for the Health Sciences, University of California, Irvine, Collection Development, Tustin, CA; **Jorge E. Perez**, Digital Learning and Information Technology Librarian, Florida International University, Herbert Wertheim College of Medicine, Miami, FL

Objectives

To collaborate with community-based organizations servicing the transgender population of South Florida while disseminating local and general consumer health information through workshops designed by the Florida International University College of Medicine (FIUCOM)-Family Medicine Department, local transgender activists, and the FIU Medical Library.

Methods: As part of a grant from the Miami Foundation, librarians collaborated with the FIUCOM's Department of Health, Humanities, and Society and FIU College of Public Health's Department of Epidemiology to develop a questionnaire used to gather demographic information and identify health risks/needs of the transgender population. The Department of Biostatistics at FIU College of Public Health collaborated in analyzing the data obtained from the questionnaire used for a cross-sectional study.

Keywords: transgender, consumer health, LGBT, LibGuide, PrEP, health information

Poster Number: 30

Time: Monday, May 29, 2017, 2:30 PM – 3:25 PM

Creating a Web-Based Digital Photographic Medical Archival Display: One Hospital Library's Experience

Caroline D. Marshall, AHIP, Senior Medical Librarian, Public Services, Medical Library, Cedars-Sinai Medical Center, Los Angeles, CA; **Fozia Ferozali**, Assistant Dean, Medical Education, Cedars-Sinai, Los Angeles, CA

Objectives: The objective was to create a Web-Based Digital Photographic Medical Archival Display, in a major community hospital in Los Angeles. This digital archive would allow the library to preserve and display its photographic collections and would also allow patrons to access the hospital's large historical photographic collection online.

Methods: We chose an open source web based software product, Omeka, which provided us with storage space of 5 gigabytes of data on an outside server. This allowed patrons to access the photographs from outside our firewall. To start the selection process, we analyzed archival reference questions from the past 2 years, the majority of which were requests for photographs. We also looked at the plans for the Timeline Wall the institution was creating for the hospital's 100-year celebration, to determine which photographs from the archive should be used. We advertised at 2 Library Science Schools for interns to work on the project. A data entry template was created using Dublin Core to ensure that the metadata was entered uniformly through the archive. Prior to scanning we spoke with our legal department regarding copyright issues.

Results: Within 8 months we had digitized and added metadata to over 500 photographs. The accessibility of the photographic archive has allowed other departments to access our historical photographs. Several departments have asked us for photographs for events and we have been able to direct them to the digital archive, where they can select and download images. This had increased our efficiency with handling requests and also has reduced the need for original photographs to be handled. We now have over 900 items in the archive.

Conclusion: Reviewing our collection and selecting which photographs to scan has led us to creating a more curated collection. The lessons we have learned has made us more aware of our collection, our goals and the material we are accepting. We will continue to build the archive and work with library science students.

Keywords: Digital archive, digital images, digital history, hospital library, digitization, metadata, photographs, Omeka

Poster Number: 31

Time: Tuesday, May 30, 2017, 1:00 PM – 1:55 PM

Developing a Digital Collection of Doctor of Nursing Practice (DNP) Student Work

Kevin Pardon, Health Sciences Librarian, Arizona State University, Dpc Library, Phoenix, AZ

Objectives: While PhD dissertations are typically accessible as part of a university library's general collection, or as content within a proprietary database, many other terminal degree projects remain invisible and inaccessible to a greater audience. This poster will describe the development and creation of a digital repository collection containing doctor of nursing practice (DNP) student's final projects.

Methods: The "Doctor of Nursing Practice (DNP) Final Projects Collection" was created over the course of one semester and included initial discussions with program faculty and administrators, the creation of a student consent form, the development of a process for adding student work to the repository collection, and a presentation to graduating students. This poster will describe the process in more detail, discuss benefits and challenges, as well as highlight the considerations to keep in mind when developing and creating a digital collection of student work. Additionally, best practices and lessons learned will also be described to provide valuable information to others considering creating this type of collection at their own institution.

Results: At the end of the first semester of implementation, twenty student projects existed in our public collection: https://repository.asu.edu/collections/260. On the whole, both faculty and students were pleased to have a collection highlighting the work being done in their program. Valuable lessons were learned that can be applied in the next semester of implementation. Specifically, metadata consistency was an issue during the initial uploading process. Gaining select faculty and student buy-in by allaying concerns related to some student's wanting to publish in a peer-reviewed journal on the topic of their final project remains vital.

Conclusion: Creating open access collections of student applied final projects or capstone projects allows for greater visibility of this type of often overlooked student scholarship. Specifically, the final projects showcased can now be found and accessed by potential employers, researchers, other schools, and other DNP students. In many cases these final projects have applied real-world impact related to answering clinical questions or patient care that should be shared with the world.

Keywords: DNP Final Projects, Student Scholarship, Institutional Repository, Collaboration, Doctor of Nursing Practice

Poster Number: 32

Time: Tuesday, May 30, 2017, 2:00 PM – 2:55 PM

Creating a Research Footprint: Introducing an Institutional Repository to Scholars

Melissa A. Spangenberg, Digital Services Librarian, New York Medical College, Health Sciences Library/ User Support, Education and Research Services, Valhalla, NY; **Carrie Levinson**, Scholarly Communications Librarian, Touro College Libraries, Touro College Libraries Midtown, New York, NY; **Jovy-Anne O'Grady**, Scholarly Communications Librarian, New York Medical College, Health Sciences Library, Valhalla, NY

Objectives: Our campus Institutional Repository was launched in 2016, utilizing bepress's Digital Commons. Our objectives are to:

• Determine campus-wide awareness of institutional repositories, subject repositories, and academic social networks.

• Assess knowledge of the scholarly benefits of depositing work.

• Discern what population would be most interested in depositing works.

• Discover respondent's reasons for and against deposit.

Methods: We plan to administer a survey assessing baseline institutional knowledge of the IR. Dissemination will be via email blast to all faculty and graduate students. Solicitation text will be included in the email and in the survey. Random self-selection will be utilized to ensure anonymity. The survey will be administered and analyzed using Qualtrics software.

After assessing institutional knowledge of institutional repositories, the scholarly benefits of depositing work in the repository, and reasons for or against deposit, the library plans to create a concrete game plan to establish buy-in to the Institutional Repository using handouts, presentations, customized guides, liaison outreach, tutorials, and webinars.

Results:

Conclusion:

Keywords: Institutional Repositories

Academic Research

Surveys

Poster Number: 33

Time: Sunday, May 28, 2017, 2:00 PM – 2:55 PM

Implementation of a Librarian-Curated Digital Repository Promoting Institutional Scholarship and Library Staff Value

Leanna Stager, Scholarly Publications Librarian, Hofstra Northwell School of Medicine, Health Sciences Library, Hempstead, NY; **Debra Rand, AHIP**, Associate Dean for Library Services, Health Sciences Library, Hofstra Northwell School of Medicine, Hempstead, NY

Objective: The Hofstra Northwell School of Medicine library implemented a digital repository to collect and showcase the scholarly output of the medical school and our health system, including works by faculty, trainees, and staff. This centralized website, with links to the full text as well as faculty profiles, facilitates access and discoverability, aligning with the value of scholarship at our institution.

Methods: Librarians initiated a planning process for the repository, conducted a survey of AAHSL libraries, reviewed repository sites and vendors, presented recommendations to the dean, and met with stakeholders to obtain input on key elements. Challenges included displaying the work of a large,

diverse group of researchers and accommodating a variety of workflows for accessing full text. Seed collections of publications from 2014-2016 were uploaded for each department. Presentations to leadership demonstrated the benefits of including additional categories of academic works. The complementary faculty profiles site, with works populated by the repository, is replacing the static faculty listings on the school's website as an ongoing collaboration between the library and the faculty affairs staff. Evaluation of the repository's effectiveness will be tracked by download counts and a survey of leadership will be conducted, collecting feedback and ideas for future directions.

Results: Following the AASHL survey and conversations with several existing customers, BePress' Digital Commons was selected to serve as the IR platform. Challenges addressed include data organization, batch upload of existing records, the use of two authentication systems across different sites, and download tracking. Faculty profiles pages were well received and will be replacing the current directory listings with the rollout of the new Hofstra Northwell School of Medicine website. Librarians are collaborating with other departments to reduce duplication of effort regarding the collection of profile content and analysis of publication metrics. Library staff are presenting the IR and profile sites to multiple target audiences, including the Chairmen's meeting and at academic department meetings.

Conclusions: Employing a soft rollout strategy allowed for consultation with key stakeholders at various steps in the process, enabling feedback to be incorporated into the repository before formally announcing the IR. Although this has extended the setup process it allowed librarians to engage with the faculty and administrators who were unaware of the benefits provided by a repository and created opportunities for collaboration on this and other projects.

Keywords: institutional repositories, scholarship, faculty publications, collaboration with faculty

Poster Number: 34

Time: Monday, May 29, 2017, 2:30 PM – 3:25 PM

Gathering Qualitative Feedback and Demonstrating Value for an Institutional Repository (IR)

Daniel G. Kipnis, Senior Education Librarian & Editor, Jefferson Digital Commons, Scott Memorial Library and the Center for Teaching and Learning, Scott Memorial Library & Center for Teaching and Learning, Philadelphia, PA

Objectives: The reports of institutional repositories dying have been greatly exaggerated. I have developed a method to gather feedback from researchers who find content in an academic health sciences institutional repository. The objective was to compile the feedback, demonstrate value of open access research and share it with the global community, University and Hospitals administrators, Deans and President.

Methods: I created a visible feedback link on cover pages of assets deposited in institutional repository and then shared feedback on institutional repository homepage. Created a 'What People are Saying about the institutional repository' page on the homepage of the IR to help demonstrate value. Also included feedback in quarterly reports sent to entire institution.

Results: Created a link for researchers to submit feedback on content they find in institutional repository and then gather feedback and share on repository homepage. To date 40+ responses have been received. Poster will outline lessons learned in creating the feedback form with visuals of how the workflow is designed.

Conclusion: Gathering user feedback from researchers who find and use content found in our IR is an effective method to help demonstrate value to University administrators, Deans and President. The 'What People Are Saying About the IR' link is a powerful tool to demonstrate the value assets in our institutional repository have had with domestic and international researchers. This poster will share the workflows in setting up the link and examples of the feedback and value that has been garnered by global users. This project echoes the theme of the conference to "Dream Dare Do"

Keywords: Institutional Repository, Value, Feedback, Qualitative, Impact, IR

Poster Number: 35

Time: Tuesday, May 30, 2017, 1:00 PM – 1:55 PM

We Built It...and They Came! Building a Repository Solution for the Future

Violeta Ilik, Head, Digital Systems & Collection Services, Northwestern University, Galter Health Sciences LIbrary, Chicago, IL; **Digital Initiatives Working Group**, Working Group, Northwestern University, Galter Health Sciences LIbrary, Chicago, IL; **Kristi L. Holmes**, Library Director and Associate Professor of Preventive Medicine, Health and Biomedical Informatics Division, Galter Health Sciences Library, Galter Health Sciences Library, Chicago, IL

Objectives: We will discuss the design and implementation of a next-generation institutional repository for an academic medical campus. This institutional repository aims to preserve a wide range of intellectual works created by the medical scholarly community, enhance the visibility of medical scholarship, and promote its authors by enabling discovery and accessibility of these works by the international scientific community.

Methods: A collaborative, cross-departmental working group (WG) was convened to execute the design, implementation, and engagement for a scholarly repository to allow scholars the ability to create, share, and preserve attractive, functional, and citable digital outputs. The WG meets in person weekly to discuss progress on key areas (technical, data, outreach, etc.) and plan upcoming work. GitHub is used to manage the development progress. The WG worked together to develop a guiding vision, and worked to develop a stable solution which facilitates reliable data management, data sharing, and citation tracking for a variety of outputs.

Results: The repository is built on a Fedora4/Hydra stack and enables machine-readable linked open data for scholarly outputs, ranging from research papers to conference materials, educational materials, white papers, case reports, supplemental images, and most recently, data sets associated with publications to help investigators comply with journal data sharing policies. Medical Subject Headings terms and persistent identifiers (e.g. DOIs) were incorporated and data streams were extended to allow us to move data seamlessly between the repository and other research information systems. Support materials were developed for users, including user guides and YouTube videos and a regular workshop.

Conclusion: The repository allows scholars the ability to create, share, and preserve attractive, functional, and citable digital outputs. The repository is prominently featured in key campus initiatives and our innovative development agenda and integration with existing systems enriches the repository and increases its value to the university. Deposited objects are unambiguously identified through persistent identifiers, enabling better association of metadata and a way of citing (and attributing) non-traditional outputs that might otherwise be undiscovered. Through blue-sky thinking and a pragmatic step-wise process, the WG delivered a cutting-edge solution which has been widely embraced by the campus.

Keywords: linked data, repository, credit, integration, interoperability, preservation, persistent identifiers, ontology

Poster Number: 36

Time: Tuesday, May 30, 2017, 2:00 PM – 2:55 PM

Implementing an Institutional Repository for Sharing, Archiving, and Accessing Research Data

Lynne Frederickson, Informationist, University of Michigan, Taubman Health Sciences Library, Ann Arbor, MI; **Marisa Conte**, Assistant Director, Research and Informatics, Taubman Health Sciences Library, Taubman Health Sciences Library, Ann Arbor, MI; **Amy Neeser**, Research Data Curation Librarian, University of Michigan, Research Data Services, Ann Arbor, MI

Objectives: Open access to research data is increasingly important to biomedical researchers. Funding agencies and publishers are implementing data sharing mandates, and researchers are recognizing that sharing data can increase the impact of their research and reusing data can advance their own science. To promote open data, a major research university library developed and launched an institutional research data repository.

Methods: Project goals:

Provide a means to publish data through a protected and secure repository.

Make research data more findable to other scholars.

Enable compliance with funding agency and journal requirements to share and archive data sets.

Facilitate citation and correct attribution by assigning a Digital Object Identifier (DOI) upon deposit.

Preserve data for future use.

House data from all disciplines, and in all data formats.

Make these services freely available to all faculty and research staff.

Provide local assistance regarding data preparation and submission.

Implementation:

Technical/platform specifications

Challenges (buyin, budget, expertise, multiple constituencies)

Rollout:

Beta phase for early promotion, user testing, feedback

Promotion strategy, including communications plan

Tailored messages for various audiences

Outcome:

Current stats (data sets, represented departments, usage)

Success stories

New/enhanced relationships with researchers/health sciences campus admin units

Connectivity w/ other campus systems

Future possibilities

Lessons learned

Results:

Conclusion:

Keywords: repository, open access, research data, data sharing, compliance, NIH funding

Poster Number: 37

Time: Sunday, May 28, 2017, 2:00 PM – 2:55 PM

Biomedical Research Data Management Open Online Education: Challenges and Lessons Learned

Julie Goldman, eScience Coordinator, NN/LM New England Region, University of Massachusetts Medical School, Worcester, MA; **Elaine Russo Martin**, Director and Chief Administrative Officer, Harvard Medical School, Francis A. Countway Medical Library, Shreswsbury, MA

Objectives: The Best Practices for Biomedical Big Data project is a two year collaboration between Harvard Medical School and University of Massachusetts Medical School, funded by the NIH Big Data to Knowledge (BD2K) Initiative for Resource Development. The Best Practices for Biomedical Research Data Management Massive Open Online Course (MOOC) provides training to librarians, biomedical researchers, undergraduate and graduate biomedical students, and other interested individuals on recommended practices facilitating the discoverability, access, integrity, reuse value, privacy, security, and long term preservation of biomedical research data. This poster highlights lessons learned from the first year of this project. These lessons learned will assist course development beyond this project, adding to best practices for creating massive open online courseware.

Methods: Built upon the New England Collaborative Data Management Curriculum, the development team sought to use existing curricular materials to create a fully online course. The course is designed with an open course platform, WordPress Learning Management System (WPLMS), in order to facilitate broad access. Each of the MOOC's nine modules is dedicated to a specific component of data management best practices and includes video lectures, presentation slides, research teaching cases, readings, activities, and interactive quizzes.

Results: The project team overcame multiple challenges related to creating an open online course:

1. Curriculum: outdated content in a format designed for in-person instruction

2. Audience: content focused on teaching research data management to librarians

3. Software: open source platform with limitations and restraints

Conclusion: Working towards overcoming these, the Best Practices for Biomedical Research Data Management MOOC development team has moved slowly and deliberately, created additional content, and added content experts to provide guidance. Lessons learned include:

1. Curriculum & Teaching Method: teaching method influences the curriculum; content should not be developed in isolation from the teaching method

2. Audience & Content: content is dependent on audience; create supplementary content to bridge audience gaps

3. Software & Technology: implementing new or unfamiliar technologies is challenging; allow more time in the timeline for project team to work with open source platform

Keywords: BD2K, biomedical, research data management, MOOC, online education, curriculum development

Poster Number: 38

Time: Monday, May 29, 2017, 2:30 PM – 3:25 PM

Journal Clubs as a Learning Method to Increase Librarians' Knowledge of Biomedical Big Data Applications

John Bramble, Research Enterprise / Outreach, Spencer S. Eccles Health Sciences Library, National Network of Libraries of Medicine, MidContinental Region, Salt Lake City, UT; **Emily J. Glenn, AHIP**, Education & Research Services Librarian, Medical Library, McGoogan Library of Medicine, Omaha, NE; **Alicia Lillich**, Kansas/Technology Coordinator, National Network of Libraries of Medicine/MidContinental Region (NN/LM-MCR), The University of Kansas Medical Center, Kansas City, KS; **Courtney R. Butler**, Clinical Librarian - Informationist, Library Services, Children's Mercy Kansas City, Kansas City, MO; **Megan Molinaro**, Library Technician, The Children's Mercy Hospital, Library Services, Kansas City, MO; **Catherine M. Burroughs**, Associate Director, National Network of Libraries of Medicine, Pacific NW Region, University of Washington–Seattle

Objectives: This poster examines the learning outcomes of two coordinated and similarly structured journal clubs, facilitated by National Network of Libraries of Medicine, Pacific Northwest Region and MidContinental Region, focusing on Biomedical Big Data and the role health sciences librarians can play to support research in this area. Session discussions focused on journal articles addressing case studies of data projects in academic medical centers, using data for quality assessment and research in clinical settings, dynamic simulation modeling approaches to understanding data use, and empowering personalized medicine through semantic web technologies.

Methods: Twenty-four health sciences librarians participated in two similarly structured online journal clubs over a period of several weeks. Following the last club session, club members completed a brief questionnaire to help facilitators understand the effectiveness of the journal clubs and learn more about the participants' overall experience. The questionnaire asked participants to rate their level knowledge of big data in clinical care settings before and after participation. Open-ended questions solicited feedback on the online journal club as a type of learning method, significant points of learning as a participant, and how the experience could have been improved.

Results: Twenty participants responded to open- and closed-ended questions, resulting in an overall response rate of 83% (20 of 24). Responses to open-ended questions were coded by the poster authors.

Conclusion: Based on the analysis of the survey data, the authors conclude that journal clubs are an effective method for increasing knowledge on the subject of biomedical big data. The number of participants reporting to have "Poor" knowledge of biomedical big data before participating in the journal club decreased from five to zero. The number of participants reporting to have "Good" knowledge increased from zero to ten. Based on the interpretation of the responses to three open-ended, participants had an overall positive experience combining an synchronous/asynchronous journal club format resulting in balanced real-time discussion sessions with extended time for reflection. Comments on the online journal club format itself, respondents suggested opportunities for improvements by incorporating techniques found in adult learning theories, inviting guest speakers, finding more efficient methods for selecting articles, and adding more meeting sessions.

Keywords: Journal clubs, data science, training, research

Poster Number: 41

Time: Sunday, May 28, 2017, 2:00 PM – 2:55 PM

How Data Are Shared by Researchers: Analysis of Data Sharing Methods in PLOS Articles by an Institution's Researchers

Yingting Zhang, AHIP, Research Services Librarian, Rutgers University, Robert Wood Johnson Library of the Health Sciences, New Brunswick, NJ

Objectives: The poster is to present how researchers share their data by investigating the patterns of data sharing methods used by Rutgers Biomedical and Health Sciences (RBHS) researchers as stated in their articles published in PLOS journals so that the health sciences librarians will understand their researchers' needs better and provide more appropriate research support services to the researchers.

Methods: Thorough literature searches were conducted in PubMed to locate all the articles published by RBHS researchers in all the eight PLOS publications during the period from March 3, 2014 when the data sharing policy began to be enforced to June 30, 2016 which is the end of FY2016 for the studied institution. All the retrieved citations and publications were placed in an EndNote library. Data were collected from the Data Availaibility Statements in the retrieved journal articles. Final data were exported to an Excel sheet for calculation and analysis.

Results: In total, 148 articles (n=148) were retrieved from PubMed meeting the search criteria with 84% of them (n=124) published in PLOS One. Among the 148 articles, 59% (n=88) of the articles had a statement that the all relevant data are within the paper and its Supporting Information files. 11% (n=17)

papers indicated that data were deposited in a repository with 11 deposited in a subject repository and 6 in a general repository. 3 articles indicated that data were available upon request and 4 stated that data were available via the third party. 36 articles had no data statement as they were submitted prior to March 4, 2013.

Conclusion: The study findings revealed that researchers do not seem to make their data available by depositing data in either subject or general repositories, as anticipated by many librarians. Instead they prevailingly chose to make data available by including the relevant data within the paper or supporting information files. Whether it is because of their lack of awareness of the repositories or unwillingness to use a repository is not known from this study. Further research should be conducted to identify researchers' attitudes toward data deposition in repositories.

Keywords: Data, Data Sharing, Data Sharing Policy, Data Availability, Researchers

Poster Number: 42

Time: Monday, May 29, 2017, 2:30 PM – 3:25 PM

Can I Get a Visual? The Impact of the New Clinical Informationist Program

Jonna Peterson, Senior Clinical Informationist; **Karen Gutzman**, Impact and Evaluation Librarian; **Corinne H. Miller**, Clinical Informationist; Galter Health Sciences Library, Chicago, IL

Objective: Clinical informationists provide expert support in the clinical care environment by fostering evidence-based decision making. In 2015, the Galter Health Sciences Library began a Clinical Informationist Service. The informationists collect data to illustrate the value of the service and highlight connections to patient care. Visualization techniques demonstrate how data is used to analyze and build upon our current service model.

Methods: Evaluation is an important element in implementing a new service. There are three topical areas that are tracked by the Clinical Informationist Service and compiled into a monthly data report. The first area helps us assess our productivity by tracking our interactions on the clinical floors and our work with clinical departments through liaison relationships. The second area helps us better leverage our resources by tracking the questions asked and the resource used to answer them. The third area focuses on additional work generated as a result of the clinical informationist service and the liaison relationships.

Results: From August 2015 through December 2016 the clinical informationists participated in 87 unique patient care rounds. They interacted with attending physicians, medical residents, and medical students. There were 80 unique questions harvested from in-person interactions. The questions were categorized into four areas and each question can fall into more than one category. Information was also collected on the database or resource, and document type used to answer the question. Data from each topical area has been visualized using a variety of tools to better communicate the impact of the service.

Conclusions: The clinical informationists provided evidence-based information in the complex patient-care environment by their interaction with more than 180 people over a 16 month period. Detailed monthly reports to track questions, interactions and effort help demonstrate the value of the service and lay the groundwork for a more formal evaluation in the future.

Keywords: Evaluation, Impact, Visualization, Clinical Informationist, Evidence-based clinical practice

Poster Number: 43

Time: Tuesday, May 30, 2017, 1:00 PM – 1:55 PM

National Information Center on Health Services Research and Health Care Technology (NICHSR) ONESearch: A New Search Interface for Information Retrieval

Patricia E. Gallagher, AHIP, Librarian, National Library of Medicine, NICHSR, Bethesda, MD; **Lisa Sedlar**, Librarian, National Information Center on Health Services Research and Health Care Technology, NICHSR, Bethesda, MD; **Brooke Dine**, Head, Web & Information Management Unit, Public Services Division, National Library of Medicine, Bethesda, MD

Objectives: Create a new interface to seamlessly connect researchers to the breadth of resources supplied through the National Library of Medicine's National Center on Health Services Research and Health Care Technology.

Methods: In partnership with NLM's Reference and Web Services and Office of Computer and Communications Systems, NICHSR developed a new search interface that allows users to search four important web resources curated by NICHSR. HSRProj, which provides information on projects in progress; HSRR, a database of datasets, instruments and software related to health services and public health systems and services research; and HSRIC and PHPartners, two current awareness services with a wealth of web resources and grey literature related to public health and hsr.

Results: The new search interface provides quick access to all resources, or to "jump off" to a particular website for more complex searching.

Conclusion: Using web metrics and search reports, the team will continue to perfect the search interface to better meet the needs of users.

Keywords: health services research, public health, datasets, research, information systems

Poster Number: 44

Time: Tuesday, May 30, 2017, 2:00 PM – 2:55 PM

An Invitation for Informationists: Joining a Data Harmonization Research Project Already in Progress

Diana Nelson. Louden, Biomedical and Translational Sciences Librarian, Health Sciences Library, University of Washington–Seattle; **Joanne Rich**, Information Management Librarian, Health Sciences Library, Health Sciences Library, Seattle, WA

Objectives: Inspired by the National Library of Medicine’s call to add informationists to existing R01 research teams, an internal medicine researcher met with librarians to explore a collaboration. The group developed a successful administrative supplement proposal submitted directly to the National Institute on Aging (NIA). This poster documents the initial activities of two informationists participating on an Alzheimer's Disease research team.

Methods: The aims of the NIA administrative supplement are to make the products of the team’s data harmonization research more accessible and useful to the scientific community in the long term. The informationists joined the team in the third year of a four-year funded project. The initial plan was to conduct a comprehensive information needs assessment including: (1) gaining familiarity with the aims, methods, and team members of the Alzheimer’s Disease research project; (2) documenting the types of data inputs and outputs and the data analysis being conducted; (3) learning the team's current data management practices; and (4) investigating suitable repositories, documentation methods, and mechanisms for sharing data. Outcomes will include: (1) developing new skills to support the research endeavor and (2) developing an action plan for realizing the ultimate goal of facilitating the secondary use of the team’s harmonized data.

Results: The informationists quickly became familiar with the research team's work via two main mechanisms: examining existing documentation in the team project folder and attending regularly scheduled team meetings where the collaborative research is carried out. This helped them identify the key data analyses and the data types involved. Through the data harmonization process, items on psychometric tests are assigned to one of four variables being measured; then recalibrated psychometric scores of patients from multiple longitudinal studies are analyzed against their genetic profiles to look for correlations with the four variables. To understand current data management practices, the informationists are interviewing team members and observing the team's collaborative process of analyzing data and publishing articles. The informationists are also investigating existing models of managing harmonized data, the availability of suitable repositories (both general and discipline specific), and documentation requirements and recommendations.

Conclusion: The informationists are expanding their knowledge of reproducibility of research, data management vocabulary, and data sharing practices. Their focus is to determine which types of data can be shared, how best to share, when to refer to other data sources, and how to enhance the discoverability and secondary use of the research products in a self-sustaining manner.

Keywords: informationists, data sharing, research data management, harmonized data

Poster Number: 45

Time: Sunday, May 28, 2017, 2:00 PM – 2:55 PM

National Library of Medicine Informationist Supplement Grant: Daring to Dive into Documentation to Determine Impact

Barbara Rochen. Renner, Library Services Evaluation Specialist and Liaison, Allied Health Sciences and Professor, Department of Allied Health Sciences, School of Medicine, Health Sciences Library, University of North Carolina at Chapel Hill, Chapel Hill, NC; **Francesca Allegri**, Assistant Director & Head of User Services, Health Sciences Library, University of North Carolina at Chapel Hill, Chapel Hill, NC; **Barrie Hayes**, Bioinformatics and Translational Science Librarian, Health Sciences Library, Chapel Hill, NC

Objectives: Three informationists were integrated with an NIH R01-funded research team for two years on an NLM Administrative Supplement for Informationist Services from 2014 to 2016. What impacts, implicit and explicit, have resulted from this partnership? What recommendations can this team make about research services generally and specifically to this library? What suggestions can this team make for this grant program going forward?

Methods: Prior informationist awardees supported by National Library of Medicine administrative supplements have reported on their experiences through webinars, publications, grant reports, and posters. This collective wisdom was valuable in planning for the evaluation portion of this library's administrative supplement. This team used a logic model; researcher and informationist interviews; notes from more than 30 team meetings and debriefings; field notes and memos; and impact tables (positive and negative impacts on the R01 research team/their research and on the informationists/library) to collect data. Analytical methods included 1) an independent review of these data by two librarians to compile a master list of key themes and 2) a second independent review by these librarians for impact evidence (direct quotes, observations, and inferences). Broad categories that have emerged to date include impacts resulting from informationist participation; challenges; successes; replicable service ideas; how researchers work; and lessons learned (e.g., for informationists, for the NLM grant program). This poster will summarize these methods, findings, and recommendations.

Results: The use of a logic model at the beginning of the grant and ongoing updates of the impact tables throughout aided tracking and reporting of progress and impacts. The added decision to collect qualitative data, the act of collecting it, and first-pass analysis of those data also helped the team a) demonstrate the value of embedded informationists, b) understand researchers' work context, and c) identify librarians' professional development needs. Understanding researchers' work contexts helps

librarians target not only resources and services but how and when to get researchers' attention. In order to support researchers more effectively, librarians in liaison roles need expanded knowledge of the local research infrastructure as well as knowledge of local and major granting agencies' funding mechanisms, processes, timelines, jargon, and staffing.

Conclusion: The use of varied evaluation techniques, including a logic model, qualitative methods, and keeping detailed impact tables throughout the life of an NLM Administrative Supplement for Informationist Services is a worthwhile strategy for demonstrating the impact of informationists embedded in research teams.

Keywords: informationists, qualitative data, impact, research services, documentation, NLM grant program, qualitative data analysis

Poster Number: 46

Time: Monday, May 29, 2017, 2:30 PM – 3:25 PM

Text-Mining PubMed Search Results to Identify Emerging Technologies Relevant to Medical Librarians

Patricia F. Anderson, Emerging Technologies Informationist, Taubman Health Sciences Library, Taubman Health Sciences Library, Ann Arbor, MI; **Skye Bickett, AHIP**, Assistant Director of Library Services, Library, Georgia Campus - PCOM, Suwanee, GA; **Joanne Doucette**, Associate Director, Library and Learning Resources, MCPHS Library, Boston, MA; **Pamela Herring, AHIP**, Electronic Resources Librarian, Harriet F. Ginsburg Health Sciences Library, Harriet F. Ginsburg Health Sciences Library, Orlando, FL; **Andrea C. Kepsel, AHIP**, Health Sciences Educational Technology Librarian, MSU Libraries, East Lansing, MI; **Tierney Lyons**, Librarian, Cerebros Medical Systems, Jessup, PA; **Scott McLachlan**, Librarian, Oxford Institute for Energy Studies, Oxford, England, United Kingdom; **Carol Shannon**, Informationist, Taubman Health Sciences Library, Taubman Health Sciences Library, Ann Arbor, MI; **Lin Wu, AHIP**, Instructional Associate Professor / Pharmacy and South Texas HSC Campus Librarian, Texas A&M University, Medical Sciences Library, Kingsville, TX

Objectives: The Emerging Technologies Team, part of the Medical Library Association (MLA) systematic review (SR) projects, conducted a pilot study to identify emerging technologies relevant to medical librarians. The team analyzed results from its previously reported PubMed Search filter using text-mining to identify patterns, themes, and trends important to the practice of medical librarianship.

Methods: We began by establishing a common competency base through custom training sessions from higher education data-mining experts. Next, the team 1) reviewed and finalized the emerging technologies PubMed search strategy created for the project; 2) exported the data; 3) used automated tools to clean extraneous data from the data set; and 4) tested the data by running preliminary text-mining scans. Steps 3 and 4 were repeated to refine and focus the results. Data export, cleaning, and visualization were done using tools such as GREP, OpenRefine, AntConc, R, FLink, pubmed.mineR, and others.

Results: Will be provided at MLA Annual Meeting.

Conclusion: Will be provided at MLA Annual Meeting.

Keywords: text mining, data visualization, OpenRefine, emerging technologies, systematic review, PubMed

Poster Number: 47

Time: Tuesday, May 30, 2017, 1:00 PM – 1:55 PM

Tracking Departmental Scholarly Publications

Gale Oren, AHIP, Librarian, Kellogg Eye Center, Kellogg Eye Center, University of Michigan, Ann Arbor, MI

Objectives: Ongoing faculty publication statistics are of great value to administrators. They provide productivity metrics on both the author and departmental levels. However, identifying, tracking, and reporting of these publications creates a few substantial challenges, including name ambiguity, Epub ahead of print, avoiding duplication, and meaningful presentation and distribution of this data. Statistical reports can be requested for quarterly, calendar or fiscal year, funding source, or by article type.

This poster will demonstrate the process used by the librarian at the University of Michigan Department of Ophthalmology and Visual Sciences. It will identify and offer solutions for the problems and challenges faced in this endeavor.

Methods: Articles are identified on a weekly basis using author name searches in PubMed and by Researcher ID searches in Scopus. Authors are also encouraged to provide advance notice of articles in press, or anything not otherwise reported. Identified records are imported into annual EndNote libraries where they are labeled by publication month (or Epub), and type of article (clinical, bench, translational). They are further processed with funding details and NIH Public Access Policy compliance information where appropriate. Records labeled as Epub are updated as final publication details become available.

Results: Weekly emails are sent out to the department as a whole showcasing scholarly activity, and this has been well received. Using EndNote search features and record labeling, reports are generated on demand for quarterly internal faculty communications, medical school reports, funding source bibliographies, annual reports, etc. The annual libraries are also utilized by the librarian for managing and tracking NIH compliance.

Keywords: publication tracking, faculty productivity, bibliometrics, scholarly publishing

Poster Number: 48

Time: Tuesday, May 30, 2017, 2:00 PM – 2:55 PM

Assessing Research Impact: How Good Is Good Enough?

Megan von Isenburg, AHIP, Associate Director, Research and Education, Medical Center Library & Archives, Medical Center Library & Archives, Durham, NC; **Virginia M. Carden, AHIP**, Administrative Research Librarian, Duke University, Medical Center Library/Administration, Durham, NC; **Patricia L. Thibodeau, AHIP, FMLA**, Associate Dean for Library Services & Archives, Medical Center Library & Archives, Library Administration, Durham, NC; **Brandi Tuttle, AHIP**, Research & Education Librarian, Medical Center Library & Archives, Duke University, Durham, NC

Objectives: To compare a recently developed gold standard method for determining the research impact of authors to more automated processes available through Web of Science, Scopus and Google.

Methods: Librarians developed a comprehensive “gold standard” process for determining an author’s career and 10-year h-index. The process includes manually checking citations against CVs, determining prior work and education locations, and disambiguating similar names. While the final data gathered is thorough and clean, the process is time consuming and unrealistic on an institutional level. To assess how much was gained by this cumbersome but accurate process, we selected a purposive sample of authors from our data set based on various criteria that may predict a difference in the comprehensive “gold standard” h-index versus the automated h-indices from Web of Science and Scopus. Factors examined were length of career, degree (MD or PhD), length of time at current institution, and commonality of last name. H-indices from Web of Science, Scopus, and Google were compared to the “gold standard.”

Keywords: research impact, metrics, faculty, bibliometrics

Poster Number: 49

Time: Sunday, May 28, 2017, 2:00 PM – 2:55 PM

Novel Approach to Institutional Publication Tracking

Ann Marie Clark, Library Director, Arnold Digital Library, Seattle, WA; **Beth Levine**, Systems Librarian, Fred Hutchinson Cancer Research Center, Arnold Library, Seattle, WA; **Allysha Eyler**, Scholarly Communications Librarian, Fred Hutchinson Cancer Research Center, Arnold Library, Seattle, WA; **Jessica L. Tobin**, Technical Services & Systems Librarian, Fred Hutchinson Cancer Research Center, Arnold Library, Seattle, WA

Objectives: Create a system to streamline the identification and tracking of all publications authored by our institution's faculty and collaborators associated with our scientific initiatives. Collect the associated citations and apply metadata including local authors, organizations, core grant-funded shared resources utilization and publication and person identifiers. Use results in reports to leadership, on websites and in analytics.

Methods: • Automated, weekly search of PubMed and Web of Science by institution/address.

• Results collected in EndNote, de-duplicated, then output in a custom style.

• Citations are uploaded to a SharePoint/Excel synced table where custom workflows run our list of authors against the search results to identify possible matches.

• SharePoint workflow identifies potential local author, culling citations from 600-800 to ~150.

• The remaining ~150 citations are assigned to Librarians that review each paper and confirm as ours or reject as a false positive.

• Unique identifiers and local authors are “attached” to papers in SharePoint and papers are added to the internal repository.

• Verified publications are compiled via a SharePoint workflow into a weekly update distributed to leadership, communications, senior faculty and legal.

• Publication lists and reports are routinely prepared for administrators and funders.

Keywords: Scholarly Communications; Institutional Publication Tracking; Bibliometrics; Publication Analytics; Author Disambiguation

Poster Number: 51

Time: Tuesday, May 30, 2017, 1:00 PM – 1:55 PM

Searching by Grant Number: Analysis of Web of Sciences and PubMed Search Results

Kimberly R. Powell, Research Impact Informationist , Woodruff Health Sciences Center Library, Emory University Woodruff Health Sciences Center Library, Atlanta, GA

Objectives: This case study showcases an academic health sciences library's experience with designing comprehensive search strategies for a given grant number. Through our research impact and analysis services, we assist with annual progress reports and funding renewals. Key to this is the identification of recent citations to specific grant support. Award administrators also seek to supplement self-reporting with robust search-strategy alerts.

Methods: Using a research center grant awarded by the NIH (P30 grant) as a case study, we will discuss the search strategies developed for the PubMed and Web of Science databases as well as the different indexing practices and result variations. Of particular importance are the common author mistakes in award citations (confusion of letters and numbers, transpositions, missing zeros, etc.) and

how each database responds to such scenarios. Since the example center grant covers over 200 associated researchers, the documentation and careful inclusion of common variations is an important element of comprehensive searching. Final search results were compared against NIH RePORTER records (https://projectreporter.nih.gov/reporter.cfm) to determine extant of coverage in each databases under the finalized search strategies and the effect of author reporting variations on database results.

Results: Results found a total of 15 unique grant variations in Web of Science and 13 unique citation variations in PubMed. These variations returned 549 total publications in Web of Science and 851 publications in PubMed. NIH RePROTER listed 685 publications linked to our grant of interest. All NIH RePORTER listings were returned by the PubMed variations. However, both Web of Science and PubMed returned unique results when compared to the NIH RePORTER listings as well as to each other. Excluding the most current year (2016) approximately 20% of grant citing publications were not captured in NIH RePORTER. For those publications not in NIH RePORTER, 99.9% had PMCIDs. Causes for exclusion have yet to be determined.

Conclusions: Searching by standard grant citation formats in Web of Science and PubMed respectively yielded 55% and 82% of total identified publications. Once citation variations for a given grant have been determined, PubMed offers the most robust best coverage. Comprehensive results should be supplemented with Web of Science searches to capture additional unique articles. NIH RePORTER offered only about 80% citing publications identified, all of which were included in PubMed results. Ongoing analysis of variation possibilities may be needed to ensure that saved searches or alerts continue to capture relevant citation variations.

Keywords: Bibliometrics Citation analysis Web of Science PubMed Grants/funding Search strategies

Poster Number: 52

Time: Tuesday, May 30, 2017, 2:00 PM – 2:55 PM

The Faculty Publications Metrics Project (FPM) at the New York University (NYU) Health Sciences Library

Stuart Spore, Lead, Scholarly Output Assessment/Senior Systems Advisor, NYU Langone Medical Center, NYU Health Sciences Library, New York, NY; **Catherine Larson**, Head, Systems & Technology / Systems Librarian, NYU Langone Medical Center, NYU Health Sciences Library, New York, NY; **Brian Han**, Senior Web Developer, NYU Langone Medical Center, NYU Health Sciences Library, New York, NY; **Ian Lamb**, Senior Developer, NYU Langone Medical Center, NYU Health Sciences Library, New York, NY; **Aileen McCrillis**, Assistant Director, User Services, NYU Health Sciences Library, NYU Health Sciences Library, New York, NY; **Fred LaPolla**, Knowledge Management Librarian, Data Services, NYU Health Sciences Library, New York, NY; **Kevin B. Read**, Knowledge Management Librarian, NYU Langone Medical Center, NYU Health Sciences Library, New York, NY; **Alisa Surkis**, Head, Data Services and Translational Science Librarian, NYU Health Sciences Library, NYU School of Medicine, New York, NY

Objectives: The NYU Health Sciences Library hosts a comprehensive database of faculty publications (the Faculty Bibliography) which supports a range of services. Among these are bespoke reports for department chairs and other administrators. These are created manually on request. The FPM project seeks to replace the manual process with a self-service online tool for report creation.

Methods: In 2013 the Library established a Faculty Bibliography API which provided simple lists of publications, but was not designed to support complex bibliometric reports. When needed such reports were scripted by hand. This was opaque, cumbersome, and time consuming. Consequently the service was simultaneously underutilized and burdensome for Library staff.

In late 2015 we began in depth discussion on how to improve the situation. Initially the project was conceived as a simple portal to existing reports, but it rapidly became clear that a more unified and interactive solution was desirable and that integrated visualizations would be needed.

In the following months, the Library defined use cases and thrashed out policy and design issues, examining similar efforts at other medical centers.

Results: By late Spring 2016 we reached a working consensus on project design requirements and coding began in earnest. The result is the NYU Faculty Publication Metrics site which is now in the process of being rolled out to our users.

Keywords: Bibliometrics, Visualization, Service Design

Poster Number: 53

Time: Sunday, May 28, 2017, 2:00 PM – 2:55 PM

Daring to Think Outside the Box: Using EndNote to Create a Research Matrix

Virginia M. Carden, AHIP, Administrative Research Librarian, Duke University, Medical Center Library/Administration, Durham, NC; **Beverly Murphy, AHIP, FMLA**, Assistant Director, Communications & Web Content Management; Hospital Nursing Liaison, Medical Center Library & Archives, Duke Medical Center Library & Archives, Durham, NC; **Connie Bishop**, Clinical Assistant Professor, North Carolina A&T State University, College of Health and Human Services - School of Nursing, Greensboro, NC; **Jamie Conklin**, Research & Education Librarian, Duke University, Duke Medical Center Library, Durham, NC

Objectives: School of Nursing PhD and Doctor of Nursing Practice students accumulate articles for their Capstone/Theses projects over several years. This poster shows how EndNote was used to facilitate creating a research matrix to efficiently organize and evaluate journal articles.

Methods: A need for a way to reduce duplication of data recording was identified by a student during a library EndNote session. The student and librarian then collaborated to explore the use of EndNote as a means of gathering and reporting article data (e.g. purpose, setting sample, research design, variables, results, and evidence). Taking advantage of numerous and generally unused fields in an EndNote reference, the journal article reference type was modified to create the matrix fields. The APA 6th Output Style was augmented to include the various matrix fields using a common delimiter. The completed references were then exported into EXCEL and formatted as needed. A handout was developed with detailed instructions and used in succeeding classes.

Keywords: EndNote, Research Matrix, Collaboration

Poster Number: 54

Time: Monday, May 29, 2017, 2:30 PM – 3:25 PM

All Ready Reference: A Program to Increase Knowledge and Use of Disaster Information Resources

Sarah Carnes, Clinical Librarian, Bedford VA Medical Center, VHA New England, Arlington, MA

Objectives: This project investigated options to increase awareness and use of disaster-related resources from the National Library of Medicine Disaster Information Management Research Center and other organizations among Massachusetts libraries and librarians.

Methods: I adapted the National Network of Libraries of Medicine outreach project guide and booklets' guidance and divided my tasks by phases. The first phase was the assessment of Massachusetts libraries' status and the factors affecting outreach. The assessment synthesized interviews, surveys, a literature review, and training observations. The second phase focused on the design of a training strategy and products, incorporating a logic model and schedule. Evaluation and assessment is a

necessary component of any outreach activity, so I also identified process assessment methods and developed an outcomes assessment plan. The third phase was the delivery and reception of the strategy. As the purpose of this initial delivery is to ensure the quality of the strategy, infographic, and research guide, the chosen evaluation method was qualitative, with quantifiable indicators and objectives.

Results: The post-activity survey feedback was very useful as it led to some revisions. It also validated the program design and content: all respondents were interested, enthusiastic, and supportive of the strategy and materials. They were satisfied by the training and believe that the outreach activities will be useful and effective. These results meet the indicator goal and exceed the objective of 70% indicating satisfaction. Additionally, the respondents felt the materials were comprehensive and inclusive while still being user-friendly. Several commented that they did not know about the available resources and were excited to use them.

Conclusion: Based on the delivery feedback, I believe that the program implementation strategy is a viable way of increasing knowledge of and use of resources and tools that will enhance libraries' and cultural institutions' preparedness in Massachusetts and other states. Various means of publication, including posting on stakeholder websites with a brief introductory video, will enhance its appeal and acceptance. Periodically, the program and materials should be reviewed to ensure that they incorporate the most recent and relevant guidance. As the program was developed as part of fieldwork with a federal agency, the All Ready Reference materials are freely available.

The program has been adopted by several libraries in Massachusetts. They tools have also been adapted for the State Library of New Jersey.

Keywords: Disaster information, Infographic, Research Guide, Training Program, Assessment, Emergency Preparedness, Public Libraries, Outreach

Poster Number: 55

Time: Tuesday, May 30, 2017, 1:00 PM – 1:55 PM

Helping Health Information Go Viral: Building a Disaster Information Specialist Network

Siobhan Champ-Blackwell, Health Sciences Librarian, Disaster Information Management Research Center, National Library of Medicine, Bethesda, MD; **Robin Taylor**, Librarian, ICF (supporting National Library of Medicine) , Arlington, VA; **Stacey Arnesen**, Chief, Disaster Information Management Research Center, National Institutes of Health, National Library of Medicine, Bethesda, MD; **Cynthia Love**, Technical Information Specialist, National Institutes of Health, , Bethesda, MD

Objectives: The Disaster Information Specialist Program is a collaborative effort to explore and promote the role of librarians and others in the provision of disaster-related information resources throughout all phases of a disaster or public health emergency. A three pronged approach was created to implement the program.

Methods: The Disaster Information Specialist program consists of a three-pronged approach:

1. A series of freely available online training courses that provide a foundation to build capacity for public health personnel, librarians, emergency workforce and others.

2. Monthly webinars that provide the opportunity to hear from experts on the latest issues in disaster medicine and public health.

3. A community of practice that functions through an online discussion forum, email updates and social media.

Results: As of October 2016, 66 people in 20 states and three internationally-based persons have earned a Disaster Information Specialization certificate from the Medical Library Association.

Conclusion: The program has been instrumental in providing a cadre of responsive individuals, across the United States and beyond, who are involved in preparing and providing health information before, during, and after disasters.

Keywords: disaster, public health, emergency, training, disaster medicine

Poster Number: 56

Time: Tuesday, May 30, 2017, 2:00 PM – 2:55 PM

"Lights, Camera, and...Action": Librarians as Simulated Patients: A New Frontier for Student Skills Development

Madeleine Bruwer, Subject Librarian, Monash University, C L Butchers Pharmacy Library, Parkville, Victoria, Australia; **Barbara Yazbeck**, Learning Skills Adviser, Monash University, CL Butchers Pharmacy Library, Parkville, Victoria, Australia; **Maxine Cuskelly**, Manager Pharmacy Library, Monash University, CL Butchers Pharmacy Library, Parkville, Victoria, Australia

Objectives: Skills development and training is an integral part of the medical librarian's role. This poster describes a recent foray into professional communication skills development where librarians in collaboration with their learning skills counterparts act as simulated patients in the Bachelor of Pharmacy. This presents an opportunity for librarians and learning skills advisers to be recognised for their professional competencies, including interpersonal and communication skills that contribute to the development of graduate employability skills. Our goal is to equip students with the interpersonal skills required for patient-focused communication that employers value in pharmacy graduates.

Methods: In 2011, the Faculty of Pharmacy at Monash University introduced Objective Structured Clinical Examinations (OSCEs) into a second year core unit. OSCEs are performance based assessments that test students' interpersonal skills and clinical knowledge in presenting them with a short clinical scenario. This involved the use of simulated patients to develop and assess interpersonal and communication skills.

Initially, our small Library team made up of librarians and learning skills advisers, was approached to participate in TOSCEs (Teaching OSCEs) as observers. Our input was well-received, as simulations are known to be resource intensive, requiring staffing and time. Since then, our role has expanded to include teaching in TOSCEs as well as acting as simulated patients in workshops throughout the semester and in the exams. Acting as a simulated patient involves using a script and prompts to simulate a real-life interaction between a pharmacist and a patient in a community or clinical setting. This also includes interprofessional communications between pharmacists and other health professionals such as general practitioners.

Results: This collaboration between faculty and library has resulted in increased engagement in the curriculum, spanning across the whole degree. As a result, we now find ourselves partnering with content specialists to teach, deliver and assess patient-focused communication skills to first year, second year and third year undergraduate students. This new role has been very successful ensuring our continued involvement and expansion into new curriculum to be introduced this year (M.Pharm).

Conclusion: This novel approach of engaging librarians and learning skills advisers in the curriculum acknowledges our professional competencies, and offers new opportunities for medical librarians to partner with faculty in preparing and developing work-ready pharmacists.

Keywords: Clinical communication skills, pharmacy students, role-play, pharmacy education, simulated patient, Objective Structured Clinical Examination, professional communication, standardised patients, graduate employability

Poster Number: 57

Time: Sunday, May 28, 2017, 2:00 PM – 2:55 PM

Librarians Dreamed of Participating in a Mock Mass Casualty Incident: Did They Dare to Do It?

Paula Craig, Head Librarian, Library, NSU College of Nursing School of Allied Health, Shreveport, LA; **Tammy Curtis**, Associate Professor/Graduate Faculty, NSU College of Nursing School of Allied Health, NSU College of Nursing School of Allied Health, Shreveport, LA; **Willie Andress**, Designated Regional Coordinator, LSU Health Sciences Center - Shreveport, Louisiana Region 7 Hospital Preparedness Coalition, Shreveport, LA; **Montie' Dobbins**, User Access Services, Head, Health Sciences Library, Health Sciences Library, Shreveport, LA; **David Duggar, AHIP**, Head, Library Liaison Program, Health Sciences Library, LSU Health Shreveport, Shreveport, LA

Objectives: Every three years Region 7 of the state of Louisiana holds a mock mass casualty incident (MMCI). Librarians participated due to their past involvement with the Local Emergency Preparedness Committee (LEPC). This poster presents the roles the librarians had in preparation of the event, activities during, and future needs from the "hot wash" debriefing and follow-up Region 7 meeting afterwards.

Methods: Six months before the scheduled MMCI, librarians attended planning sessions involving Region 7 emergency response teams (ERT) to discuss how their skills might be utilized during the event. Librarian roles for the MMCI were organizational and administrative beforehand, and exploratory of future needs at the conclusion. The nursing librarian’s facility was used for moulage training sessions. Nursing and Radiological Science students participated as the actors/victims of the MMCI. Nursing librarian and faculty created moulage kits for the seven local hospitals and the two on-site disaster scenes at the local airport. On the day of the exercise, the nursing librarian was the Site Actor Controller and the medical librarian was Observer. Site Actor Controller supervised the moulagers and student actors. The Observer role was to photograph and take notes. At the conclusion of the exercise librarians attended a "hot wash" debriefing and distributed a survey by email to Region 7 ERT. In addition, the LEPC held a meeting to report findings of MMCI, and a Region 7 meeting was held to report findings from the MMCI exercise and survey.

Results: The "hot wash" and follow-up meetings noted varied success in learning objectives and difficulties when an MMCI scenario is not scripted. The Information Resources Needs Assessment survey revealed 40% of responders needed information during the MMCI. Eighty percent of responders used text messaging, phone conversations, and 800 MHz portable radios. Twenty percent used an app on mobile devices or asked another responder. While 100% of respondents found the information needed, one response noted communication is always an issue and another noted the need for a map of the airport, gate numbers and instructions on how to access the airport.

Conclusion: Librarian roles were relevant and welcomed in preparation for and during the MMCI. The librarians continue to work with the LEPC to assess, educate, and provide new resources for a successful response to a mock and real MCI.

Keywords: Disaster Preparedness, Mass Casualty Incident, Librarians Role, Moulage

Poster Number: 58

Time: Monday, May 29, 2017, 2:30 PM – 3:25 PM

Partnering with Global Education: Teaching PubMed to Chinese Medical Faculty in an English as a Second Language Program

Irene Machowa Lubker, Research and Education Librarian, Tompkins-McCaw Library for the Health Sciences, Virginia Commonwealth University, Richmond, VA; **Emily Hurst, AHIP**, Head, Research and Education, VCU Libraries, Tompkins- McCaw Library for the Health Sciences, Richmond, VA; **Talicia**

Tarver, Research and Education Librarian, VCU Libraries: Tompkins McCaw Library, Virginia Commonwealth University, Richmond, VA

Objectives: To describe the experiences of medical librarians at a large urban academic health sciences center tasked with teaching database searching to Chinese medical faculty, who were participating in an English as a second language (ESL) program on the university's academic campus.

Methods: The English as a second language program coordinator for the Global Education Office at our university approached the library to fulfill the wishes of their students to learn how to search medical databases. Students came from different medical disciplines and serve as faculty at their home university in Baise, a city west of Guangxi Zhuang Region, China. The students learned English with a certified instructor for a few months before visiting the library. English proficiency ranged from beginners to intermediate. Library sessions included a library tour and instruction on PubMed and a brief discussion of SinoMed. One-on-one consultations with the librarian were also conducted upon request. Language challenges during sessions were resolved through partnering intermediate English learners with beginners.

Results: Students learned the basics of searching PubMed and how to access their citations/articles once they went back to China. Held in 2015, the first class was successful. By request, librarians presented two classes on PubMed and conducted an extended tour of the health sciences campus in 2016.

Conclusion: The coursework and tour offered a good way to facilitate practicing the English language while dealing with issues familiar to medical professionals, as well as increased the library's visibility and role in global engagement. This program created a new relationship between the health sciences library and the Global Education office which continues with plans for future faculty groups from China and other countries.

Keywords: global engagement, China, medical education, ESL, global education, Instruction, PubMed

Poster Number: 59

Time: Tuesday, May 30, 2017, 1:00 PM – 1:55 PM

How Do Health Care Practitioners Evaluate Library and Information Services in Japan?

Yukiko Sakai, AHIP, Associate Professor, School of Library and Information Science, School of Library and Information Science, Keio University, Saitamashi, Saitama, Japan; **Yoko Sato**, Researcher, National Institute of Public Health, Center for Public Health Informatics, Wako, Saitama, Japan; **Masae Sato**, Librarian, Library, Chibaken Saiseikai Narashino Hospital, Narashino, Chiba, Chiba, Japan; **Makiko Watanabe**, Librarian, Kanagawa Children's Medical Center, Library, Clinical Research Institute, Minamiku, Yokohama, Kanagawa, Japan

Objectives: The outcome of library and information services in patient care has rarely been examined in Japan and is therefore unclear. Thus, the purpose of this research is to clarify the value of library and information services in clinical settings. The results are expected to show the performance of the services and necessary information requirements for improving patient care.

Methods: The research team worked on developing protocols replicating and translating the "Facilitator Handbook" used for the Value Study in North America in 2011. Cultural differences were carefully reflected in the protocols. After the ethical approval of the Institutional Review Board, participating sites were recruited through a public relations tool of an association related to medical libraries. In the pilot, a web-based survey and interviews were undertaken among physicians, residents, and nurses at two sites in February and March, 2016; it requested their information seeking behavior for a specific clinical incident in the last 6 months. The number of survey respondents was 241 and the overall response rate was 11.5%. The main survey was conducted in October and November, 2016, at 5 sites.

Results: The number of responses for the main survey was 382, meaning a response rate of 7.3%. The most sought types of information were: therapy information (63.4%), diagnosis (42.1%), drug information (41.6%), prognosis (36.4%), and clinical guidelines (33.3%). Physicians used mostly PubMed (79.9%), JAMAS Web (a bibliographic database provided by the Japan Medical Abstracts Society) (62.9%), and UpToDate (38.1%), while nurses used books (59.7%), JAMAS Web (47.5%), and electronic books (20.1%). They highly valued the information as a source of new knowledge (92.2%), an answer to clinical questions (86.1%), and as qualified clinical care (83.4%). Furthermore, 72.3% of respondents recognized changes in clinical care as a result of the information. The information highly contributed to positive changes in the selection of therapy (73.3%), diagnosis (63.2%), and selection of drugs (38.0%) among physicians, but contributed less among nurses in terms of advice to patients or family (62.1%) and post hospital care or treatment (37.4%). The information also contributed towards avoiding unwelcome events such as additional tests or procedures (39.7%), adverse drug reactions or interactions (30.7%), and patient misunderstanding of diseases (27.0%). They acknowledged information resources provided by libraries (78.9%) as being as important as discussions with colleagues (77.5%).

Conclusion: The findings demonstrated the effective and detailed use of library and information services in clinical settings in Japan.

Keywords: Information Services, Librarians, Libraries, Medical, Professional Role, Access to Information, Information Seeking Behavior, Patient Care, Patient Outcome Assessment, Comparative Effectiveness Research

Poster Number: 60

Time: Tuesday, May 30, 2017, 2:00 PM – 2:55 PM

Contributing to the International Mission of the University: A Librarian Goes to Madagascar

Michele R. Tennant, AHIP, Interim Fackler Director, Health Science Center Library, Biomedical and Health Information Services, Health Science Center Libraries, Gainesville, FL; **Michael M. Miyamoto**, Professor, Biology, University of Florida–Gainesville; **Jill Ranaivoson**, Senior Study Abroad Advisor, International Center, University of Florida–Gainesville

Objectives: This poster illustrates the integration of a librarian into a university's efforts to increase students' international experiential learning opportunities. The librarian's efforts directly support the institution's educational mission and the service and teaching components of her role as a tenured faculty member.

Methods: University of Florida librarians are tenure-eligible faculty, with responsibilities that include teaching, research, and service. Internationalizing students' educational experiences is a priority at the university. To further this goal the librarian (with a background in biology) co-developed/co-taught a 4-week study abroad course concentrating on Madagascar's biodiversity, conservation, sustainability and culture. Madagascar provides a unique learning experience, given its staggering poverty and poor infrastructure, extraordinary but threatened biodiversity and endemic species, and its rich cultural heritage. Organizing this challenging course and attracting students who were "good fits" required skills and attributes similar to those needed to be a successful librarian - information seeking, project management, budgeting, and marketing skills; flexibility, innovative thinking, and the willingness to try something new.

Results: Twelve students enrolled in the course first offered in the summer of 2016. Five pre-departure sessions were taught, ranging in scope from biodiversity to staying healthy in Madagascar. While in Madagascar, students experienced numerous ecosystems (rainforest, spiny forest, dry forest, coral reef); participated in conservation efforts such as reforestation and sea cucumber farming; visited local institutions (orphanage, volunteer hospital, sapphire mine, tourist park); took part in cultural activities; and learned from researchers, guides, craftsmen, educators and students, many of whom are local

Malagasy. Students kept a daily field notebook/diary to reflect on their experiences. The librarian participated in all activities and acted as co-leader of the trip. Student feedback indicated that the study abroad was a positive learning experience for the students and had great impact on their future career plans. The librarian found the experience to be transformational and rewarding. The library built a new collaboration with an important campus partner; the International Center has asked that the course be repeated in 2017.

Conclusion: Many librarians have skill sets and interests beyond those that are required for their specific job. Whether gained through advanced degree, life experience or other means, finding a way to match these skills and interests in concrete ways to the priorities of the institution can result in impactful opportunities for the institution and its students, rewarding experiences for the librarian, and the development of new and significant partners for the library.

Keywords: Internationalization, Faculty, Madagascar, Educational Mission, Institutional Goals

Poster Number: 61

Time: Sunday, May 28, 2017, 2:00 PM – 2:55 PM

Impacting Reproductive Health Training in Ethiopia: Building Information Skills Capacity in an International Setting

Gurpreet K. Rana, Global Health Coordinator, Taubman Health Sciences Library, Taubman Health Sciences Library, Ann Arbor, MI; **Lia Tadesse**, Program Director, Center for International Reproductive Health Training, University of Michigan Medical School, Ann Arbor, MI; **Berhanu Gebremeskel**, Senior Project Manager, University of Michigan, Center for International Reproductive Health Training, Ann Arbor, MI

Objectives: University of Michigan's Center for International Reproductive Health Training (CIRHT) and the Taubman Health Sciences Library (THL) are collaborating to build information skills capacity in obstetrics and gynecology training and research within a network of Ethiopian medical schools. The overall objective of the interdisciplinary collaboration is to enhance information literacy and bring awareness of information resources in reproductive health research and clinical care in developing country setting.

Methods: THL's global health informationist began working with CIRHT in 2016 to plan on-site and distance information skills training; identify development opportunities for faculty and students; and consult on integrated, evidence-based information skills curricula to enhance medical education in reproductive health training. Additionally, the informationist is working with CIRHT leadership to develop research protocols and expert searching strategies to evaluate reproductive health literature.

Results: Collaborative activities have included assessment of information skills perception, behavior and needs in cultural context; investigation of integration of information seeking skills into existing educational competencies in preclinical service in Ethiopia; leveraging information skills training strategies and resources; and educating faculty and clinicians on quality online information resources openly accessible in the educational, research and clinical settings.

Conclusion: Informationists and librarians are not integrated in the clinical or research settings as commonly in Ethiopia as in the United States. Due to the recognition of information seeking proficiency as a skill in lifelong learning of the medical professional, there is a need to build capacity and consider increased integration of informationists and librarians in health care settings and research in Ethiopian health sciences schools. The ongoing collaboration between CIRHT and THL has been a successful one as CIRHT's pilot site in Ethiopia continues to actively work towards empowering women through improvement in reproductive health education. Future activities include development of digital learning objects and collaboration with information professionals in Ethiopia.

Keywords: reproductive health, Ethiopia, global health, international, education, curriculum

Poster Number: 62

Time: Monday, May 29, 2017, 2:30 PM – 3:25 PM

Designing Comic Books to Promote Environmental Health Information to Adolescents

Alla Keselman, Senior Social Science Analyst, Specialized Information Services, Bethesda, MD; **Brock Eastman**, Science Teacher, Walter Johnson High School, Science Education, Bethesda, MD; **Karen Matzkin**, Outreach Specialist for NLM, National Library of Medicine, Specialized Information Services, Bethesda, MD; **Judy Kramer**, Public Health Specialist, National Library of Medicine, SIS, Specialized Information Services, Bethesda, MD; **Janice E. Kelly, FMLA**, Acting Deputy Associate Director, Specialized Information Services, Specialized Information Services, Bethesda, MD

Objectives: This work describes the process of designing comics about environmental health impact of climate change. The six-chapter comics is a story of planet Wüb's science expedition to "Urf" (Earth). The goal of the expedition is to understand similar trends in the warming of the two planets. The chapters can be used as part of high school science curriculum.

Methods: Division of Specialized Information Services of the National Library of Medicine collaborated with a local high school science teacher who also had an arts background on developing the comics. The development involved the following phases: 1) Background research; 2) Storyboarding; 3) Art design; 4) User testing; and 5) Final design. The background research was conducted via the institution's environmental health education resources for high school students and the general public. The story aimed to provide an engaging, entertaining plotline and graphics, while also explaining complex science issues around the formation of Earth's atmosphere, the effect of human activity on its composition and temperature, and the impact of climate change on ecosystems and human health. The information is aligned with the Next Generation Science Standards. Each chapter is supplemented with a teacher guide and student activity materials.

Results: Classroom evaluation pending.

Conclusion: Comics and graphic novels are a potentially effective way of engaging adolescents (and the public) with complex, emotionally charged health information.

Keywords: adolescents, K-12, health education, science education, environmental health, comic books

Poster Number: 63

Time: Monday, May 29, 2017, 2:30 PM – 3:25 PM

Comic Creation as an Innovative Library Role: Process and Resources

Patricia F. Anderson, Emerging Technologies Informationist, Taubman Health Sciences Library, Taubman Health Sciences Library, Ann Arbor, MI; **Elise Wescom**, Media Assistant/Graphic Artist, University of Michigan, Taubman Health Sciencs Library, Ann Arbor, MI; **Kai Donovan**, Media Developer, Taubman Health Sciences Library, University of Michigan, Ann Arbor, MI; **Ruth Carlos**, Professor, University of Michigan-Ann Arbor, Radiology, Ann Arbor, MI

Objectives: The library has been providing graphic medicine support within the medical school in a variety of ways. This led to a partnership with clinical research faculty and a major medical research journal, through which library created a five-page comic to illustrate communication challenges in the clinician-patient encounter, with a goal of creating empathy on both sides.

Methods: To support the comic creation process, the library designed a workshop on rapid-prototyping of comics, created tools to facilitate and support visual and story-line aspects, evaluated comics creation software and hardware for purchase. The library took lead on selecting and managing staff

involved in the comic creation, including a graphic artist. The team involved in the project also co-authored supporting content, resource selection, and other supporting and creative aspects. As the first comic designed as an article for a peer-reviewed research journal, there were special aspects required for the peer-review process. The poster will provide describe the resourcing, content development, collaboration management, project management, peer-review, and actual creative process for this innovative project.

Keywords: graphic medicine, comics, health literacy, professional communication, Twitter, social media, visual literacy

Poster Number: 64

Time: Tuesday, May 30, 2017, 2:00 PM – 2:55 PM

Graphic Medicine in the Library: An Educational Outreach Program

Matthew N. Noe, Library Fellow, Lamar Soutter Library, Worcester, MA

Objectives: Graphic medicine refers to the discourse of healthcare by way of the medium of comics and is a growing field with far-reaching impact. The objective of this outreach program was to provide educational opportunity to librarians on building graphic medicine collections and the creation of related programming.

Methods: The outreach program proceeded on several fronts over the course of a six-month period, beginning with a two-part webinar series targeted to librarians both in-and-out of the region. These webinars sought to provide background information on graphic medicine, materials to aid with collection development, including key title lists, and serve as brainstorming opportunities for programming, including potential partners. At the same time, outreach was conducted with libraries in the local region, seeking to build community ties and support for the creation of new collections and programming. The program, in this iteration, concluded by creating a series of book club kits, that target specific medical conditions and include graphic novels, suggested questions, further readings, and more. These kits are available to regional groups for borrowing, including libraries who may find them valuable as a trial before building a new collection.

Results: Will be made available on the completed poster.

Conclusion: Will be made available on the completed poster.

Keywords: Graphic Medicine, Outreach, Book Club, Collection Development

Poster Number: 65

Time: Sunday, May 28, 2017, 2:00 PM – 2:55 PM

"What Are Mammograms and Breast Cancer?" Developing a Culturally Tailored Narrated Slideshow on Mammograms and Breast Cancer for Somali Women

Rekha Ravindran, Program Coordinator, Interpreter Services, University of Washington Health Sciences Library, Seattle, WA; **Carolyn Martin, AHIP**, Consumer Health Coordinator, University of Washington, NN/LM Pacific Northwest Region, Seattle, WA

Objectives: Develop content for a video slideshow narrated in the Somali language and an associated provider guide, which considers the major barriers to engaging in mammograms with goal to increase overall rates of mammograms among Somali women.

Methods: To determine major barriers, information was collected through literature review, focus groups, and interviews with key community members, advisers, and clinicians. Qualitative data from

these responses were assessed for major themes. This data was integrated into the script of the slideshow. The provider guide was developed based on major barriers and content in the slideshow. Once drafted, the slideshow narrative and provider guide were reviewed by physicians and Somali community members, then culturally tailored based on feedback. The final products are an 18-minute narrative slideshow called “What are Mammograms and Breast Cancer?” and an associated “Provider Guide.”

Results: Formative research showed a variety of major barriers, for example low health literacy, lack of knowledge about cancer, and religion.

Conclusion: This tool can be shown in clinics, at the discretion of the provider. We recommend the slideshow play while a patient is waiting in the exam room with the provider present to answer questions or in the community to women-only audiences, to respect Somali women’s modesty.

Keywords: health education, multicultural, mammograms, breast cancer, Somali language

Poster Number: 66

Time: Monday, May 29, 2017, 2:30 PM – 3:25 PM

My Doctor Said What!? Identifying and Assessing Online Health Information Resources

Nicole Theis-Mahon, Liaison to the School of Dentisty & HSL Collections Coordinator, Health Sciences Libraries, University of Minnesota–Minneapolis; **Shanda Hunt**, Public Health Library Liaison & Data Curation Specialist, University of Minnesota, Health Sciences Libraries, Minneapolis, MN

Objectives: Health information consumers look to the Internet to find answers to questions about their health or that of a loved one. We conducted a study to identify where individuals find online health information, how they use it, and what they think is missing. Results from this study are being used to make recommendations of how to improve services to this population.

Methods: The University of Minnesota Health Sciences Libraries conducted a cross-sectional study of adults in August 2016. The survey instrument was adapted from the eHealth Literacy Scale (eHEALS) and the Patient Activation Measure (PAM-13), administered electronically on tablets at the Minnesota State Fair, and took approximately six minutes to complete. Convenience sampling yielded a total of 281 participants. Analysis of descriptive statistics and statistics to explore relationships between variables were conducted using R, and a qualitative analysis of one survey item was conducted using NVivo.

Results/Conclusion: Preliminary results show that a majority of participants use a search engine, such as Google, WebMD, or the Mayo Clinic website, to locate online health information. While most respondents were confident in their ability to evaluate the health resources they find online, only half identified indicators of quality health information. This result was confounded by the high number of participants who were health providers. Participants identified personalization of and interactivity with health websites as highly desirable.

Keywords: Health Information, Health Literacy, Internet, Decision Making

Poster Number: 67

Time: Tuesday, May 30, 2017, 1:00 PM – 1:55 PM

Health-Related Website Usage by Persons with Serious Mental Illness: Design and Use of a Heath Literacy Survey Tool

Len Levin, AHIP, Head, Education and Clinical Services, Lamar Soutter Library, University of Massachusetts Medical School, Worcester, MA; **Zlatina Kostova**, PHD, University of Massachusetts Medical School, Department of Psychiatry, Worcester, MA; **Joanne Nicholson**, Principal Investigator, Geisel School of Medicine at Dartmouth, Department of Psychiatry, Lebanon, NH; **Kathleen Biebel**, Investigator, University of Massachusetts Medical School, Department of Psychiatry, Worcester, MA; **Elaine Russo Martin**, Director and Chief Administrative Officer, Harvard Medical School, Francis A. Countway Medical Library, Shrewsbury, MA

Objectives: A health literacy focused web site review survey was designed as an initial step in a multi-institutional project to build a website for persons with serious mental illness to help them better understand issues relating to their physical health. This presentation will describe the design, testing, implementation and results of this survey tool.

Methods: The literature shows that persons with serious mental illness (SMI) approach the use of online health information differently than the general population. In 2015, the University of Massachusetts, in collaboration with other academic medical institutions, received a grant to build a new website for persons with SMI that will teach them how to find high quality online health information and will specifically guide them to information about their physical health. As a first step, the project team created a health literacy based survey tool to evaluate current health websites for their utility with an SMI audience. The survey was designed using and building upon an existing validated instrument. It was administered to experts on mental and physical disease. Results will be used to determine quality indicators of the new site and to selected sites to which it will link.

Results: 13 reviewers were identified to complete the survey. Four of the identified participants did not complete the task and others were identified to take their places. Ten participants ultimately completed the surveys. Participants were asked to review between four and five websites focusing on four different topics - cardiovascular health, diabetes, obesity and smoking - all comorbidities with prevalence in the SMI community. The websites were chosen based on Google searches that were performed using examples of layperson searches observed in preliminary focus group activities. The top five non-advertiser-supported sites were included. The survey consisted of 61 questions. The questions were developed using existing open access survey tools (e.g., the DISCERN instrument) and findings on website usage by people with SMI that were discovered in the existing literature. Questions focused on format, navigation, usability and credibility of the sites. Questions were also asked about any etiologic, diagnostic, therapeutic or prognostic information contained in the sites. 65 responses were received.

Conclusion: Results of the survey demonstrated a sampling of health websites that met the criteria for effective use with an SMI population. The authors believe that this survey could also be adapted and used as a general comprehensive health website evaluation tool. It will be made available as an open access document.

Keywords: Health Literacy, Consumer Health Information, Mental Health

Poster Number: 68

Time: Tuesday, May 30, 2017, 2:00 PM – 2:55 PM

Pilot Project to Teach Current and Future Health Care Professionals How to Address Patients with Health Literacy in Mind

Christine A. Willis, Director of Knowledge Management & Learning Resources, Noble Learning Resource Center, Shepherd Center, Atlanta, GA; **Skye Bickett, AHIP**, Assistant Director of Library Services, Library, Georgia Campus - PCOM, Suwanee, GA; **Carolann Curry**, Library Assistant Professor, Reference & Outreach Librarian, Mercer University, Mercer Medical Library, Macon, GA; **Tara Douglas-Williams**, Division Head for Information Services/Library Manager, M. Delmar Edwards, M.D. Library, Morehouse School of Medicine, Atlanta, GA

Objectives: To develop a program that will improve the ability to identify and communicate effectively with patients at the appropriate level of health literacy.

Methods: A pre-test, presentation, post-test, and online resources intended for health science students, researchers, and clinicians was developed. The pre- and post-tests were the same and were based on content. The presentation consisted of lecture points and active learning to fully engage participants. Small focus groups consisting of researchers and clinicians were held to provide feedback on the assessments, presentation, and online resources.

Results: Focus groups stated that the presentation should have more focus on patient-centered care but that the information would be a useful reminder for practitioners. Suggestions were made to include more images on slides and provide additional videos about methods mentioned in the presentation. Including stories about the benefits of health literacy was recommended as a way to show relevance to everyday practice. Focus group members also provided feedback on active learning that would help improve an attendee's application the information. Revisions were made to all portions of the project so that it can be presented in medical schools and health care settings.

Conclusion: The focus groups stated the need for introductory and refresher sessions on health literacy. Training classes will be offered in person for students, researchers, and practicing clinicians at medical schools and in health care settings. Online options will be presented for reference for those who are unable to attend in person training. It is anticipated that the presentation and online resources will help improve communication with patients and increase patient satisfaction.

Keywords: health literacy, clinicians, students

Poster Number: 69

Time: Sunday, May 28, 2017, 2:00 PM – 2:55 PM

Taking the Pulse of Health Literacy Knowledge at an Academic Medical Center

Kelsey Leonard, AHIP, Health Information Services Librarian / Assistant Professor; **Cynthia J. Beeler, AHIP**, Associate Professor / Clinical Information Librarian; **Martha Earl, AHIP**, Assistant Director / Associate Professor; Preston Medical Library / Health Information Center, Knoxville, TN

Objectives: To evaluate health literacy knowledge among medical center staff as well as increase awareness of health literacy throughout the medical center.

Methods: The Clinical Librarian and Health Information Services Librarian received an award from the National Network of Libraries of Medicine to assess the medical center's health literacy knowledge. Surveys were distributed to nurses, the Patient Education Committee, and hospital administration. The IRB-approved survey asked about their knowledge of health literacy and if they were interested in further training. Flash drives that contained health literacy documents were ordered and distributed. Bookmarks with health literacy information were created with the marketing department and distributed with the flash drives. A health literacy banner was designed and displayed in the library. Three health literacy classes were offered and promoted to the medical center staff. Nurse managers were contacted for those floors that wanted more information on health literacy and meetings were scheduled. A follow up survey was sent to nurse managers to distribute.

Results: 193 surveys were completed and returned. 96% of respondents indicated understanding of the concept of health literacy and how it impacts patient outcomes. However, only 78% indicated that health literacy understanding is important to their department. 91% of respondents wanted to learn more about health literacy. Almost every floor in the hospital had a least one staff member requesting further training. Librarians attended three meetings with nurse managers, 10 nursing unit council meetings, and three staff huddles. Two people attended the health literacy classes. Overall, librarians talked with 242 nurses about health literacy. Twenty-one nurses completed the follow up survey.

Conclusion: Librarians were able to evaluate the health literacy knowledge amongst medical center staff through a survey. They were then able to increase awareness of its importance through classes,

meetings, and staff huddles. Efforts are ongoing to further increase awareness and knowledge, such as a medical center-sponsored health literacy forum and a health literacy education “pop up” in the medical center lobby.

Keywords: Health literacy, Nurses, Award, Academic Medical Center, Survey

Poster Number: 70

Time: Monday, May 29, 2017, 2:30 PM – 3:25 PM

Students Dare to Dream with Health Video Productions

John Sanchez, MD-PhD Candidate, University of Utah, School of Medicine, Salt Lake City, UT; **Jean P. Shipman, AHIP, FMLA**, Executive Director, Knowledge Management & Eccles Health Sciences Library, Spencer S. Eccles Health Sciences Library, Spencer S. Eccles Health Sciences Library University of Utah, Salt Lake City, UT; **Tallie Casucci**, Innovation Librarian, Spencer S. Eccles Health Sciences Library, Eccles Health Sciences Library, Salt Lake City, UT; **Anita Leopardi**, Instructor, University of Utah, Health Promotion and Education, Salt Lake City, UT

Objectives: To offer a student health video competition to encourage interdisciplinary students within a university to create video health messages for peers and patients that will be incorporated into personal electronic health records, hospital patient television, as well as university and library websites. The videos address a need for another medium for patient education and multi-lingual education.

Methods: A student leader worked with a university-wide faculty judging team to conduct a second annual student health video competition. Details include: 1) competition requirements, 2) awards and judging rubrics, 3) promotion, and 4) social events. A large publicity campaign, including social media, promoted the competition. Students were incentivized to form interdisciplinary teams and produce multi-lingual videos. Health topics of particular need were identified via community clinics and public health agencies. Five awards were announced at a capstone event. Foundation funding supported the awards and the social events.

Results: Competition details include: a snappy competition name and logo; team registration, formation and membership guidelines; award instructions and criteria; and how to submit the videos. Winning videos are aired on the university’s television station and on the hospital patient education TV station. All videos are on the library’s website.

Conclusion: Student and faculty participation was rewarding as it acquainted faculty and students across the university. Student ambassadors welcomed the opportunity to encourage the creation of health videos. Collaboration with community organizations identified health topics in need of educational videos. Publicity helped to highlight how a health sciences library can bring together many individuals to create another successful collaborative student competition.

Keywords: health literacy, videos, patient education, students, peer instruction, messaging

Poster Number: 71

Time: Tuesday, May 30, 2017, 1:00 PM – 1:55 PM

SHARE 2.0: Spreading Health Awareness with Resources and Education

Yini Zhu, Managing Librarian, Head of Access Services; **Mina Ghajar**, Information and Education Librarian; George F. Smith Library of the Health Sciences, Newark, NJ

Objectives: Following the success of our NLM-funded SHARE program last year, we are now launching SHARE 2.0, a deep dive effort to provide further health literacy resources and education to

patients and physicians. In this effort, we intend to specialize our targeting of the two groups so that our approach and messaging is tailored to the needs of each.

Methods: A key insight we gained from our previous SHARE program (NN/LM MAR grant funded) was that target audiences have different learning needs. Patients and families are first and foremost concerned with their health diagnoses when in a hospital setting and therefore respond best to outreach on their own time. Meanwhile, physicians are often juggling busy schedules and respond best to short programs that are targeted to specific subjects. To address this, we plan to launch SHARE 2.0: Spreading Health Awareness with Resources and Education. We plan to create streaming videos to be run on digital signage in high traffic areas to provide health literacy education:

The literacy program in streaming videos formats will cover NLM MedlinePlus, Drug information, and consumer health information resources at Rutgers University Libraries. This streaming video literacy program will be constantly available, providing visitors the flexibility to review at their convenience.

Streaming videos will be in English, but we will explore the possibility of providing content and recording in non-English languages.

Results: A detailed program results report will be submitted and included on the poster for MLA 2017.

Conclusion: All collected data will be analyzed prior to MLA 2017, and a conclusion will be drawn by then.

Keywords: Patient literacy, consumer health, patient education, MedlinePlus, health information resources, library outreach

Poster Number: 72

Time: Tuesday, May 30, 2017, 2:00 PM – 2:55 PM

Medical Library Support for Peer Language Navigators in Anchorage, Alaska: Partnering to Help Individuals with Limited English Proficiency Find Reliable, Culturally Relevant Health Information

Sigrid Brudie, Medical Librarian, Alaska Medical Library, University of Alaska Anchorage, Anchorage, AK

Objectives: This poster will provide an overview of the “Outreach to Peer Language Navigators” project and its goal to increase peer language navigators’ (PLNs) knowledge and ability to access and share high-quality health information.

Methods: Setting/participants/resources: The Anchorage Health Literacy Collaborative (TAHLC)—a partnership between the Alaska Literacy Program (ALP), University of Alaska Anchorage’s public health, nursing, and psychology departments, Providence Hospital, YWCA, and Anchorage Neighborhood Health Center—has been providing health outreach to the city’s limited-English-proficiency population since 2007. In 2013, UAA’s Alaska Medical Library (AML) joined the Collaborative after they procured an outreach award from the National Network of Libraries of Medicine Pacific Northwest Region to support training of peer language navigators (PLNs) to identify credible and educationally and culturally appropriate health information to share with community members in their native languages.

Results: The first cohort was deemed so successful that a second proposal was submitted and funded. Since then Providence Medical Center in Anchorage, AK has paid a stipend to each PLN for the next two cohorts.

Conclusion: Surveys of PLNs are performed after each training session and concluding survey followed by interviews are conducted at the end of the four sessions to determine (1) number of PLNs who reported increased confidence in finding health information online, (2) number of PLNs who could give an example of what they learned, and (3) number of PLNs who could name attributes of a reliable

online health resource. The first three cohorts have continued to work in their communities under the guidance of an outreach nurse from Providence Medical Center.

Keywords: peer language navigators health literacy community outreach immigrants

Poster Number: 73

Time: Sunday, May 28, 2017, 2:00 PM – 2:55 PM

Coloring Your "Art" Out: Outcomes of Offering Coloring Materials in Targeted Hospital Staff Areas

Tara J. Brigham, Medical Librarian, Mayo Clinic Libraries, Winn-Dixie Foundation Medical Library, Jacksonville, FL; **Chrysanthe M. Yates**, Program Director, Mayo Clinic, Lyndra P. Daniel Center for Humanities in Medicine, Jacksonville, FL

Objectives: Healthcare staff burnout is a widespread national issue that has been reported extensively and the need for stress relief activities is apparent. Coloring books for adults have grown in popularity due to the promise of such benefits as relaxation. A pilot program was started to determine if placing coloring materials in the library could help reduce stress or anxiety.

Methods: The librarian and Humanities in Medicine program director at an academic medical organization devised a plan to offer color-in sheets and colored pencils to healthcare staff. The coloring materials were located in a hospital library. Although participants were not obligated to do so, a short, online survey focusing on anxiety was available for participants to complete after they finished using the coloring supplies. Those who completed the survey were eligible to win a $25 Apple gift card. While coloring therapy does not incorporate all elements of art or relaxation therapy, it may help promote a state of engagement brought about through artistic expression and thus may help to decrease people's experiences of anxiety.

Results: There were four different categories of the color-in sheets: Anatomy, Animals, Mandalas and Modern Art. Out of the 111 coloring sheets available, 84 were taken or colored during a period of six months. Even with the incentive of winning a gift card, none of the participants filled out the short survey. Therefore, is no demographic information of the user population or feedback on levels of anxiety before or after the coloring exercise.

Conclusion: Future considerations include adding a paper version of the survey for the participants to complete and also reduce the number of questions asked in the survey. While a library can serve as a location for relaxation purposes, it is important to consider other high-traffic areas in a hospital, such as break rooms or lunchrooms as well. It is also important to consider other hospital staff, such as art or humanities coordinators, whom librarians can team up with to reduce anxiety and burnout in healthcare staff.

Keywords: art, coloring, healthcare staff, anxiety, library services

Poster Number: 74

Time: Monday, May 29, 2017, 2:30 PM – 3:25 PM

Celebrate the History of Your Library for National Medical Librarians Month

Louisa Verma, Electronic Content & Medical Reference Librarian, Huntington Hospital Health Sciences Library, Huntington Memorial Hospital, Pasadena, CA

Objectives: After assisting with a hospital historical timeline project for the public relations department, the librarians wondered if there was enough information for a similar historical timeline to be created for

the library. Our objective was to see if our archival collections of hospital publications and annual reports would provide any insight into the history of the medical library.

Methods: The librarians and library volunteers scavenged the hospital's old annual reports, internal newsletters and other hospital publications in the library's collections for any photo or mention of the medical library. Further, an Internet search was conducted on each of the former library managers that yielded some unexpected additional information about the library and its librarians. Any news or narrative about the library was saved and documented along with the publication title and year. Information of significant interest such as photos of the library/librarians, articles on and other accounts of the library were compiled to form a better picture of the history of the medical library and how it has evolved. Photos and text of interest were scanned in high resolution to be used on a bulletin board and in an online slide presentation.

Results: This project began with the expectation of finding a few historical photos of the library. The end result was a more complete picture of the history of the hospital's medical library over the past 65 years. We discovered many previously forgotten facts about the library, including: year established; the volunteer guilds fundraising efforts; past librarians/staff; how collections evolved; and photos of past librarians/staff. We used the findings to create an automated slide presentation for display during National Medical Librarians Month. A bulletin board display was also created from the slides to exhibit in the hallway near the library.

Conclusion: In our fast-paced society, taking the time to look back upon those who have come before us provides inspiration and acknowledges the work of past librarians. National Medical Librarians Month provides an opportunity to honor and celebrate the contributions of every day librarians to our own institutions.

Keywords: history, national medical librarians month, hospital librarianship, archives

Poster Number: 75

Time: Tuesday, May 30, 2017, 1:00 PM – 1:55 PM

Patient Family and Hospital Staff Information Needs at a Pediatric Hospital: An (Abbreviated) Analysis of Information Requests Received by the Family Resource Libraries

Hannah Rutledge, AHIP, Head of Clinical Informationist Services, Woodruff Health Sciences Center Library, Emory University, Atlanta, GA

Objectives: To better understand the information needs of patient families and hospital staff at a pediatric hospital by addressing two research questions: 1. To what extent have patient families used the Family Resource Libraries to meet their information needs? 2. To what extent have hospital staff used the Family Resource Libraries to meet their information needs?

Methods: Data was collected from January 2011 to December 2013 by five librarians in four libraries within a pediatric hospital system in Texas. It was collected as daily library usage statistics and compiled into weekly, monthly, and yearly library statistics for hospital administration. During that time, the libraries received a total of 102,439 visitors. There were 1,406 requests for information, 848 from patient families and 558 from hospital staff. This research focused on the 1,406 information requests. Eight variables were analyzed for patient family information requests: year, month, classification, library, request type, specific document, resources used, time spent. The hospital staff information requests shared those eight variables in addition to three more: department, job title, reason for request. The data for the two groups were analyzed separately. The raw data was then compiled and the total data was analyzed by individual library.

Results: Of 58,615 patient family visits to the libraries, 848 (1.45%) involved information requests. Patient families sought primarily medical information in formats that they could take home. The Internet was the most used resource and information was typically printed out for them to take at no charge. A

majority of their requests were answered in five minutes or less. Of 10,473 hospital staff visits, 558 (5.32%) were requests for information. Hospital staff sought primarily clinical information for the purposes of EBP and Research. The Internet was the most used resource, followed by Subscription Databases. A majority of their requests were answered within 30 minutes.

Conclusion: The total number of information requests steadily declined between 2011 and 2013. Patient family information requests declined, and Hospital Staff information requests increased. Hospital Staff requested primarily clinical information and received their requested information in 30 minutes or less, while Patient Families requested mainly medical information and received information in 5 minutes or less. The Internet was the most used resource for both groups. The most requested topics included Nutrition, Diet, Exercise; Autism; Asthma; Diabetes; and School Issues. This study can help the Family Resource Libraries better identify and understand the information needs of their users. It also contributes to the greater understanding of patient family and hospital staff information needs and can be replicated for other types of hospitals. By working to further understand the information needs of patient families and hospital staff, hospital libraries continue to care for patients in numerous information-centric ways.

Keywords: hospital library, medical librarianship, information needs, patient family, hospital staff, library statistics.

Poster Number: 76

Time: Tuesday, May 30, 2017, 2:00 PM – 2:55 PM

From Many to One: Streamlining Library Services across Regions

Joy Rodriguez, AHIP, Assistant Medical Librarian, Kaiser Permanente, Health Sciences Library, Fresno, CA; **Ana M. Macias**, Manager of Library Services SAC/CVA, Kaiser Permanente, Health Sciences Library, Sacramento, CA; **Marie Beckermann**, Assistant Medical Librarian, Kaiser Permanente, Health Sciences Library, Harbor City, CA; **David Keddle**, Director - Medical Library Services, Kaiser Permanente, Health Sciences Library, Woodland Hills, CA; **Peggy Makie**, Library Manager, Kaiser Permanente, Health Sciences Library, Roseville, CA; **Jeffrey Prock**, Assistant Medical Librarian, Kaiser Permanente, Health Sciences Library, Los Angeles, CA

Objective: To organize the inter-regional health sciences libraries of Kaiser Permanente into an integrated library and information network by creating standardized policies governing all library functions regardless of geographical location.

Setting/Participants: Eight committee members, two of which served as Chair and Advisor. Committee members represented 31 hospital libraries across three regions in Northern California, Southern California and the Pacific Northwest.

Methods: The Health Sciences Libraries' Standards and Policies Committee was formed in July 2013. Over the course of a year, a series of monthly meetings were held which consisted of small group work, discussions, and review of the commonalities and differences in existing library policies. Over 70 library policies from all three regions were reviewed and the committee concluded that the existing policies fell into one of four functional areas. These were identified as: Resource Management, Library Space Management, Library Technology, and Education and Information Services. Separate policies were drafted for each of those four functions, as well as an umbrella policy of our Scope of Services. Individual librarians had an opportunity to comment on proposed revisions, regardless of committee involvement, promoting inclusion and adaptation at the user level.

Results: After creating an Unauthorized User Policy that defined library patrons, focus turned to individual library policies**.** By late 2015 a writing, editing and revision process was put in place, and in early 2016 all Kaiser Permanente Health Sciences libraries were operating in unison under one set of policies.

Discussion/Conclusion: Representatives from all three regions reviewed aspects of each of these functional areas and fleshed out areas of agreement. Differences in policies were discussed by the committee and areas of concern were taken back to the peer group at large (all Kaiser Permanente librarians) by the designated regional representative on the committee. Once the peer group approved the function, the policy was presented to the National Library for review. Any concerns were brought back to the Standards and Policies Committee and addressed accordingly. Final policy versions were approved at the National Library level and accepted for signature and implementation. Kaiser Permanente Health Sciences Libraries now have a single policy which governs all core functional areas identified by committee members. Our library services are streamlined and we function as one team, regardless of geographical location or funding source. The shared policies have brought consistency in library services across the various regions in Kaiser Permanente exemplifying One KP.

Keywords: hospital library/libraries, standards & policies, library policies, hospital librarians, medical librarians, health sciences library/libraries

Poster Number: 77

Time: Sunday, May 28, 2017, 2:00 PM – 2:55 PM

Merging Metrics: Meeting the Challenge

Mary Wittenbreer, Head Librarian, Medical Library, Regions Hospital Medical Library, St. Paul, MN; **Jennifer Feeken**, Librarian, Medical Library, Regions Hospital Medical Library, St. Paul, MN; **Patricia Saari**, Librarian, Regions Hospital, Regions Hospital Hospital Library, St. Paul, MN

Objectives: In preparation for a healthcare system merger two existing medical libraries attempted to standardize librarian activity and resource usage reporting. This is an overview of the process of how existing staff and software can be used to provide measurable documentation that demonstrates staff work and resources needed for daily operations, ROI, resource allocation, and annual budgeting process for the departments.

Methods: Based on the review of current practices of the two libraries, a negotiated definition of complexity levels (based on time and the number of databases searched) for librarian patron searches was developed. These complexity levels are incorporated into a newly developed Excel spreadsheet tracking system that also includes other patron characteristics such as discipline and rationale for the search. The tracking system serves multiple purposes for internal tracking - who requests, what is requested, the purpose of the search, who is working on the request, how long it takes to respond to the request and, if appropriate, how many resources are not locally available. This spreadsheet is easily maintained by the staff. The data has been created and formatted to enable monthly and annual charting that documents usage patterns and provides information for decision-making regarding staffing and resource purchases.

Keywords: Hospital Merger, Metrics, Data, Literature Searches, Document Requests, Tracking, Definitions

Poster Number: 78

Time: Monday, May 29, 2017, 2:30 PM – 3:25 PM

Solo Librarians: Demographics, Duties, Needs, and Challenges

Angela Spencer, Manager - Medical Library, C. Alan McAfee MD Medical Library, Chesterfield, MO; **Elizabeth Laera, AHIP**, Medical Librarian, McMahon-Sibley Medical Library, Brookwood Baptist Health, Birmingham, AL; **Halyna A. Liszczynskyj**, Director, Library Services, Medical Library, St. Elizabeth Medical Center, Utica, NY; **Louise McLaughlin**, Information Specialist, Health Sciences Library,

Woman's Health Sciences Library, Baton Rouge, LA; **Kathy Zeblisky, AHIP**, Medical Library Manager, Medical Library, Phoenix Children's Hospital, Phoenix, AZ

Objective: To obtain data on how many librarians classify themselves as solo librarians within a medical/hospital setting. Solo librarians constantly face challenges to maintain and expand services vital to their users. By quantifying their number and needs, a stronger voice can be developed.

Methods: A ten question survey using SurveyMonkey was sent to various medical library related listservs of interest to solo librarians.

Results: 383 surveys were returned, the majority from hospital and academic librarians. Other settings include clinics, organizations, research institutions and Veteran’s institutions. Duties showed the variety of hats a solo can wear. Duties included: reference, interlibrary loan, teaching, committee work, website development, marketing, creating policies/procedures, writing grants, archives, informatics and other work. The “best challenges” question was the most insightful into what the needs are for solos. Major challenges included: funding/budget, awareness/visibility, time management, value/ROI/proving your worth, staffing, space, promotion/marking/outreach, professional development, technology and organizational mergers.

Discussion: The full survey results quantify the size of the solo librarian population, and the contributions and challenges they face working in solo settings. This data can contribute useful information to discussions on best ways to support, educate, inform and advocate for this population.

Conclusion/Next Steps: Solo Librarians are faced with similar financial, marketing and operational challenges regardless of setting. We hope to encourage peers to share their challenges and concerns and work with NN/LM and MLA to educate them about solo librarians’ needs and concerns so that we can sustain our future.

Keywords: solo librarians, hospital librarians, duties, challenges, visibility, funding, value

Poster Number: 79

Time: Tuesday, May 30, 2017, 1:00 PM – 1:55 PM

Daring to Dive into Health Informatics Initiatives

Gail Kouame, Chair, Research & Education Services, Robert B. Greenblatt, M.D. Library, Augusta University, Augusta, GA; **Lindsay Blake, AHIP**, Clinical Services Librarian, UAMS Library, University of Arkansas for the Medical Sciences, Little Rock, AR; **Kathy J. Davies**, Associate Director of Research, Augusta University, Greenblatt Library, Augusta, GA

Objectives: Expand librarian roles in health informatics initiatives at an academic medical center by assimilating best practices, best evidence and guidelines into physician order sets. The overarching goal is to integrate patient education resources to meet meaningful use requirements, and to improve the evidence base for order sets and clinical decision-making.

Methods: The Director of Libraries and the Clinical Librarian approached the Chief Information Officer (CIO) of the university health system about librarian integration into hospital informatics initiatives. The CIO suggested that the Clinical Librarian begin attending the Clinical Informatics Advisory Committee (CIAC), which oversees all electronic and informatics activities in the health system. The librarian's role on this committee is to assist in adding library resources and perspectives to the projects reviewed by CIAC. Additionally, the CIO suggested that librarians join the Orders Management Team (OMT), a multidisciplinary group responsible for vetting and updating clinician order sets. All order sets will be reviewed, cataloged, and prioritized during a two-year update cycle. Many order sets did not originally include links to evidence resources when they were created. The need to link evidence to practice provided a logical starting point for librarian involvement.

Results: The librarian is now a regular member of the Clinical Informatics Advisory Committee, meeting every other week. The multidisciplinary nature of this group allows for insight into the information needs

and issues encountered in the clinical setting from many perspectives and a platform to expand patient education resources. A librarian now serves as the facilitator for the Orders Management Team and sets the agenda for the weekly meetings, in collaboration with the Information Technology support group and other team members. The librarian also adds expertise regarding the evidence links in order sets. The backlog of out of date orders has been reduced, and order sets are being processed more efficiently. After several months, the librarian is now recognized as a peer member of both groups.

Conclusion: Librarians are important members of multidisciplinary teams involving informatics activities in clinical settings. Once the librarian becomes familiar with team processes and terminology, they are equipped to fully contribute to team decisions and prioritization of projects.

Keywords: Clinical Informatics, Electronic Medical Records, Physician Order Sets, Meaningful Use

Poster Number: 80

Time: Tuesday, May 30, 2017, 2:00 PM – 2:55 PM

Are We Dreaming or Doing? How Health Sciences Graduates Access Information in Their Professional Lives

Valeria Long, Liaison Librarian to the Kirkhof College of Nursing, Grand Valley State University, Frey Foundation Learning Center, Grand Rapids, MI; **Betsy Williams**, Health Professions Liaison Librarian, Grand Valley State University, Frey Foundation Learning Center, Grand Rapids, MI; **Barbara Harvey**, Science Liaison Librarian, Grand Valley State University, Mary Idema Pew Library, Allendale, MI

Objectives: Most accreditation standards stipulate that students acquire skills to prepare them for using information in the workplace. However, the library literature suggests many graduates struggle to locate, analyze, and apply information. This study explores which resources health sciences alumnae utilize following graduation, and if respondents felt they acquired sufficient research skills during their coursework to be effective searchers and users of information.

Methods: Graduates of the Grand Valley State University (GVSU) physician assistant studies, physical therapy, nursing, and athletic training programs (n=3506) were invited to complete an anonymous online survey. Participants were asked for the date they received their most recent health sciences related degree from GVSU and if they were working in a field related to that degree. Participants indicated which resources they use most often on the job and why, how those resources are accessed, and whether they were adequately prepared while students at GVSU to effectively search health sciences literature. The study received exempt status from GVSU's IRB.

Results: 363 valid responses were received. Preliminary results indicate that PubMed was the most frequently used resource by respondents, followed by UpToDate, CINAHL, Epocrates, and Medscape. The primary reasons respondents selected these resource were currency, ease of use, and accuracy. 90.5% of respondents indicated their GVSU experiences prepared them to find information, and 89.2% felt confident evaluating information. 86.8% felt confident applying information.

Conclusions: Conclusions will be made available in the MLA '17 poster onsite and in the ePoster gallery.

Keywords: alumni, information literacy, information seeking skills, lifelong learning

Poster Number: 81

Time: Sunday, May 28, 2017, 2:00 PM – 2:55 PM

The Clinical Information Quest: A Survey of Specialists and Primary Care Providers

Bridget Gunn, AHIP, Information & Knowledge Services Librarian, Health Sciences Library, Baystate Health, Springfield, MA; **Ellen Brassil, AHIP**, Director, Health Sciences Library, Baystate Health, Health Sciences Library, Springfield, MA; **Anant M. Shenoy**, Neurologist, Baystate Health, Neurology, Springfield, MA; **Rebecca Blanchard**, Director of Healthcare Education and Assistant Professor, Baystate Health, Academic Affairs, Springfield, MA

Objectives: With the myriad of cases presented to clinicians every day at our integrated academic health system, clinical questions are bound to arise. Our goal was to investigate the rationale and thought process behind the handling of these questions among specialists and primary care providers and to identify ways to promote the pursuit of answers for more informed clinical decision making.

Methods: This study was designed and completed in two phases. In phase one, a series of focus groups was conducted with residents, attending physicians, and advanced practitioners. Data from these focus groups were coded into four themes: identifying and responding to information gaps, background knowledge of search tools and strategies, technology, and interdisciplinary factors. Twenty-seven questions were created from these themes and included in a pilot survey to gain feedback on the clarity of each item. The final tool included 35 items organized into the aforementioned themes. In phase two of the study, the survey was distributed to 1,639 residents, attending physicians, and advanced practitioners via email. Data were collected anonymously via REDCap and analyzed with Stata software.

Results: A total of 292 persons responded to the survey (18% response rate) and most were physician specialists that spent more than 80% of their time taking care of patients. The majority of respondents (84%) encountered clinical questions at least a few times per week, and while most of them often or always pursued answers, time was the biggest barrier for not following through on questions. Most respondents did not have any formal training in searching databases, were unaware of many digital resources, and indicated a need for resources and services that could be provided at the point of care.

Conclusion: This diverse group of practitioners indicated similar information needs, from tracking clinical questions to investigating the answers. Therefore, any intervention to support their needs could be universally beneficial and may not need to be tailored to individual provider types. Since there is no single favorite approach to capturing and pursuing questions, libraries and clinical training programs should explore multiple, interactive strategies and interventions. A combination of educational programs, embedded librarian services, and technology applications could be useful in helping providers pursue answers to their clinical questions.

Keywords: Information Literacy, Decision Making, Evidence-Based Practice, Surveys and Questionnaires

Poster Number: 82

Time: Monday, May 29, 2017, 2:30 PM – 3:25 PM

Implicit Association Between Epidemiological Evidence and Patient Centeredness: An Exploratory Study of Clinical Information-Seeking Expectations of Family Medicine Residents

Katherine Eastman, Clinical Information & Technical Services Librarian, Health Sciences Library, University of Alabama - CCHS Health Library, Tuscaloosa, AL; **Nelle Williams**, Library Director, Health Sciences Library, University of Alabama–Tuscaloosa

Objectives: Determine if clinicians and residents view patient-centered care and evidence-based medicine as thoroughly separate concepts.

Methods: An initial focus group to gauge perceptions of Evidence-Based Medicine in practice at a Regional University Medical Center revealed a tendency to separate the mechanisms of patient-centered care from evidence-based medicine and the general concept of medicine. A cross-sectional

survey was developed that exploded the concepts and asked clinicians, residents and third-year medical students to rank the elements according to their importance for providing the best possible care. Follow-up survey questions asked respondents to provide open text responses for the circumstances in which they seek information, how they maintain awareness of current trends and evidence in medicine, and for words that describe patient-centeredness, medical practice and evidence based medicine. Answers from the survey were used to create an Implicit Association Test to determine if the majority of clinicians and residents divide the idea of evidence-based medicine from medicine in general, and patient-centered medicine specifically.

Results: Will be made available during poster presentation.

Conclusion: Will be made available during poster presentation.

Keywords: Implicit Association Test, Evidence-Based Medicine, Student Perception, Clinician Perception, Dunning-Kruger, Confidence

Poster Number: 83

Time: Tuesday, May 30, 2017, 1:00 PM – 1:55 PM

Organized Acquisition of Cancer Research Skills through Experimental Learning and Tailored Library Instruction

Robert Campbell, Associate Professor of Pharmaceutical Sciences, MCPHS University, Department of Pharmaceutical Sciences, Worcester, MA; **Irena Dryankova-Bond**, Associate Professor and Library Manager, Blais Family Library, Massachusetts College of Pharmacy and Health Sciences, Worcester, MA

Objectives: This poster describes an experimental faculty/librarian co-instructed course designed to teach cancer research skills to first year Pharmacy students. Specifically, it identifies successful strategies used to address common challenges such as motivation, time, feedback, coaching, mentoring, resourcefulness. This course was offered for the past 5 years and has undergone continuous improvement based on results, course evaluations and students' feedback.

Methods: This course uses lab and library instruction to teach didactic and hands on pre-clinical and clinical cancer research skill set in a supportive, guiding environment. The faculty and the librarian are available to provide instruction and support, guidance, review, feedback, and mentoring to students throughout the course. Students (n=9) are organized into groups (n=3); each team investigates a specific cancer disease (i.e., lung, breast or prostate cancer) with a focus on a main theme (i.e., multidrug resistance). This course takes students through the following stages of the research process: problem identification, literature review, data analysis and presentation, research management, conclusions and results presentation, dissemination of results. Students use Cancer.gov, Medline, Embase, and Scopus to identify the evidence. They utilize RefWorks to manage their research and formal tables to synthesize and analyze the evidence, identify trends, and present conclusions. Students disseminate results in a final poster presentation and optional manuscript publication.

Results: Students' learning was assessed using a written exam to evaluate understanding of biological and physiological basis of cancer disease and specific rubrics to evaluate oral presentations, poster presentation and final paper and to provide direction and feedback. Class average for in-class written exam was 94 ± 4.86 (n=9). For presentations the combined scores ranged from 3.5 to 4.0 (n = 6) mean, 3.75 ± 0.75. Final scores ranged from 80% to 96% (n=3) mean, 93 ± 5.7. Course evaluations were overwhelmingly positive ranging between 4.8/5.0 to 5.0/50 on a 5.0 scale with 6/9 67% student response. All students reached 100% competency.

Conclusion: Organized acquisition of research skills in a supportive, guiding environment contributes to mastery of competencies. Continuous instructor's support through on-time guidance, feedback, coaching, and enthusiastic, competent mentorship are fundamental for students' motivation and

success. Blending theoretical foundation with experimental learning encourages students' learning while fostering students' motivation.

Keywords: Cancer Research, Pharmacy students, Educational Methods, Library instruction, Experiential Learning

Poster Number: 86

Time: Sunday, May 28, 2017, 2:00 PM – 2:55 PM

Cultivating Slackers: Using "Slack" to Create a Collaborative Information Literacy Learning Environment in a Longitudinal Integrated Clerkship

Katherine Eastman, Clinical Information & Technical Services Librarian, Health Sciences Library, University of Alabama - CCHS Health Library, Tuscaloosa, AL; **Nelle Williams**, Library Director, Health Sciences Library, University of Alabama–Tuscaloosa;

Objectives: Develop a method to replicate the experience of rounding with a clinical information librarian for students enrolled in a longitudinal integrated clerkship.

Methods: Two Slack Communities were created to provide Preceptors and third-year medical students with the opportunity to form communities of learning. With access to both communities, the librarian hoped to help students develop their information retrieval skills and tailor learning materials based on direct feedback gained from the private preceptor community.

Results: Preceptor feedback in the monitored community provided little insight for the development of learning materials for students, and resident engagement in the student slack community resulted in family medicine residents utilizing the slack community at a greater rate than medical students.

Conclusion: Further outreach and training may be necessary on the part of the library and the evaluation team to make Slack a viable method for creating an analogous experience for students who spend most of their clerkship away from the medical center and affiliated hospital.

Keywords: collaborative learning, clinical queries, information seeking, education, longitudinal integrated clerkship, mobile applications

Poster Number: 87

Time: Tuesday, May 30, 2017, 1:00 PM – 1:55 PM

Reimagining a Research Assignment in Performing Arts Medicine: Librarian-Faculty Collaboration to Increase Information Literacy Skills and Student Success

Hanna L. Schmillen, Health Sciences Subject Librarian, Ohio University, Alden Library, Reference & Instruction Department, Athens, OH; **Jeff Russell**, Assistant Professor, Athletic Training, and Director, Science and Health in Artistic Performance (SHAPe), Ohio University, College of Health Sciences and Professions, Athens, OH; **Sherri Saines**, Reference & Instruction Librarian, Ohio University, Alden Library-Reference & Instruction Department, Athens, OH

Objectives: This librarian-faculty collaboration aspired to enhance a specific research assignment to empower students enrolled in the Health and Injury in Performing Artists course to embrace learning about scientific research and how it pertains to their work as artists, and to further understand the research process and evaluate scientific articles related to performing arts medicine.

Methods: Our librarian-faculty collaboration launched from a library-wide initiative called Reimagining the Research Assignment. The purpose was to evaluate and improve students' research skills through partnerships between faculty and librarians. The two-part workshop allowed us to work one-on-one to incorporate a research background into the course lesson plan and develop an assignment rubric. Phase one contained general brainstorming with a group of faculty and librarians, and phase two focused solely on our specific objectives. To implement, an interactive, library information session, taught by the librarian, was included in the lesson plan before the students were given the research assignment. In addition, the instructions were enhanced to be more specific and include background information on the basics of primary, scientific research that matched the information session. Finally, a rubric was designed to assist with grading guidance and promote consistency in student expectations.

Results: The 2017 spring semester pre- and post- library instruction session survey demonstrated that the session significantly boasted student confidence in all research outcomes: getting full-text (1.9 points); knowing where to search based on topic (1.62 points); evaluating resources (1.57 points); finding credible resources (1.46 points); and searching library's databases (1.62 points). The entire class rated the librarian session as above average. From Fall 2016, several students mentioned the library session as extremely important and helpful for their class success. Overall, the assignment's results greatly improved by several points in comparison to previous classes prior to librarian-faculty collaboration.

Conclusion: The collaboration is a success in both qualitative and quantitative measures used. Evaluation is still in-process, but preliminary results show a significant increase in assignment success, students' confidence with research skills, and faculty assessment of the course overall in comparison to courses before faculty-librarian collaboration.

Keywords: Faculty-librarian collaboration, information literacy, instruction, assessment, performing arts medicine

Poster Number: 88

Time: Tuesday, May 30, 2017, 2:00 PM – 2:55 PM

Promoting Behavior Change Communication with Information Design and Health Information Literacy Strategies

Bethany S. McGowan, Assistant Professor of Library Science and Health Sciences Information Specialist, Purdue University Libraries, Purdue University, West Lafayette, IN

Interventions with a behavioral focus and that use innovative multimedia technology work well to achieve healthy behavior change by quickly and reliably providing efficient, standardized, and personalized information. This study explores the potential of digital interfaces as mediators between information and users by examining patterns of user behavior and defines the design principles needed by librarians to create such interventions.

Health sciences librarians help provide access to health information and improve people's capacity to use health information. As digital interfaces and more personalized forms of health communication are expected, librarians can utilize information design strategies to empower individuals with the health information they need to overcome barriers to health care and health care access and make better-informed health care decisions. This exploratory literature review investigates design-based strategies for providing eHealth literacy and illustrates how librarians can combine information design strategies with information literacy strategies to promote user empowerment and democratization.

Keywords: Information Design, Behavior Change Communication, eHealth, Health Literacy, Digital Divide

Poster Number: 89

Time: Sunday, May 28, 2017, 2:00 PM – 2:55 PM

Flipped Instruction in an Interprofessional Education Course

Charlotte Beyer, AHIP, Instruction and Reference Librarian, Boxer Library, Boxer Library, Rosalind Franklin University of Medicine and Science, North Chicago , IL

Objectives: The core of interprofessionlism is effective communication between members of different healthcare professions. If each member of the interprofessional team cannot find evidence needed to make strong decisions, care may be impacted. This poster outlines how a librarian introduced students from a variety of professions to strategies for locating evidence to support interprofessional practice in a flipped format.

Methods: All first year students must take a foundations course in interprofessionalism which spans the majority of the first year. Since 2010, students were required to view a series of videos on how to access information resources in the library and then take a quiz in the first few weeks of the course. In 2016, a new format was introduced where students could attend an optional session on a symposium day. The library used it as the opportunity to create a session demonstrating how to utilize advanced searching strategies to support their clinical activities. Students had remarked in various programs they wished they had advanced strategies earlier. Since this course was in the first year, this appeared to be a perfect spot to present this information. Since the students already knew the basics of accessing the full text because of the quiz, the librarian was able to create hands on activities instead of lecturing.

Results: Six students chose the library session for part of their symposium day. During the hands on session, students were challenged to use advanced searching techniques. At the end, students remarked informally that every student should have to learn the information, and it was very applicable to their fields of study.

Conclusion: Even though there was a low turnout, the students who did attend gave the session positive marks on the evaluation form. These positive marks caught the attention of the course director, and the librarians are being included in a mandatory session next year.

Keywords: interprofessional education, information literacy, literature searching, students, instruction, flipped learning

Poster Number: 90

Time: Monday, May 29, 2017, 2:30 PM – 3:25 PM

How Does Pretesting for PubMed Knowledge Spark Student Learning?

Rebecca Abromitis, Research & Instruction Librarian, University of Pittsburgh, Falk Library of the Health Sciences, Pittsburgh, PA

Objectives: Predoctoral students enter dental school with varying skill levels for searching biomedical literature databases. However, student self-perception of their ability is generally inflated. A pretest/intervention/post-test approach was developed for use in a PubMed instruction session within an evidence-based dentistry course to: help students authenticate actual ability; spark learning during the instruction session; and elicit measurable improvement in search skills.

Methods: A pretest/intervention/post-test was developed to replace a graded assignment used previously to assess PubMed knowledge and search skill within an evidence-based dentistry course. In order to objectively self-assess genuine PubMed abilities, 80 predoctoral first year dental students completed a non-graded pretest (12 knowledge questions; 8 hands-on searching tasks). While pretesting is typically used for assessment, it is also a research-based technique shown to successfully increase subsequent test scores. Pretesting was immediately followed by the intervention - a 90 minute lecture and hands-on PubMed training session. The expectation was that student motivation for learning PubMed skills during the intervention would increase, if the pretest objectively showed a difference

between students' self-perceived and actual search abilities. The session concluded with a graded (pass/fail) post-test, identical to the pretest, to determine the efficacy of the instruction session for student learning.

Results: A pretest/post-test analysis was performed to measure changes in students' PubMed knowledge and search skills. The overall class average improved on the post-test. The difference between the class average pretest and post-test scores (15.1%) was statistically significant using a two-tailed paired t-test: $t(79)$, $p<0.001$.

Conclusion: These results suggest that a pretest/intervention/post-test approach enabled students to recognize shortfalls in their PubMed knowledge and search skills, and sparked learning that was focused toward those deficiencies. Also, gains in post-test scores provided immediate gratification for both students and the instructor. However, several limitations should be considered. Since pretest/post-test analyses have no control group, they tend to have lower validities, so inferences about changes in class performance must be made with caution. Developing test questions is labor intensive; pre-post testing consumes class time; and although students' short-term retention of knowledge and skills was ascertained, long-term retention would need to be evaluated through subsequent post-testing.

Keywords: Library instruction, PubMed, Information literacy, Educational measurement, Dental students

Poster Number: 91

Time: Tuesday, May 30, 2017, 1:00 PM – 1:55 PM

MEDLIB Q-Bank Anyone? Creating a Question Bank of Medical Informatics Information Literacy Test Questions for Librarians

Lori Fitterling, Instructor of Medical Informatics/Reference Librarian, D'Angelo Library, D'Angelo Library, Kansas City, MO; **Diane Sue. Garber**, Director of Medical Library, Liberty University College of Osteopathic Medicine, Center for Medical and Health Sciences, Lynchburg, VA; **Erin Palazzolo**, Library Director and Professor of Medical Informatics, Burrell College of Osteopathic Medicine at New Mexico State University, Library, Las Cruces, NM

Objectives: As medical librarians assume more formalized roles within the curriculum and with an increase in librarians writing test questions for assessment of instruction classes, this project highlights the need for a question bank of test questions on medical informatics and information literacy as taught by librarians.

Methods: This project began as librarians started sharing test questions from courses taught on medical informatics and information literacy. With the inclusion of medical informatics and information literacy skills in required core competencies, medical librarians are teaching courses in these topics that require a formal assessment. Librarians from three osteopathic universities surveyed osteopathic medical libraries to find out how many librarians are teaching formalized courses in the curriculum, how many librarians are writing formal test questions on medical informatics and/or information literacy topics, and whether there is any interest in creating a shared bank of medical library test questions. Information about how librarians write learning objectives for lectures and relate these to exam questions was also obtained.

Results: 100% of responders indicated that their library provides information literacy sessions or classes at their universities, with 57% giving exams or tests in their courses. Of the librarians writing test questions, 62.5% indicated collaboration with other librarians, bioscientists, and clinicians for at least some of their questions, while the other 37.5% indicated that they only write questions independently. Additionally, 87.5% of the libraries that wrote test questions indicated that at least some of their questions have an osteopathic and/or clinical element. Finally, 57% of all respondents, indicated that they would use a database of library instruction test questions.

Conclusion: This project validates an interest in developing a shared database of test questions for medical informatics/information literacy as taught by academic osteopathic librarians. All of the libraries that answered the survey offer some form of instruction and the majority of librarians in our study are currently writing test questions. This data highlights the interest in working collaboratively as librarians to share exam questions for instruction classes. Additionally, the survey revealed that 43% of the responding libraries reported that they were “not welcome” or “not very welcome” within the formal curriculum with little to no time allotted for information literacy instruction. Further inquiry into the medical informatics/information literacy experiences of librarians would benefit the library instruction community.

Keywords: Medical, Informatics, Information, Literacy, Question, Bank, Test, Questions, Assessment

Poster Number: 92

Time: Tuesday, May 30, 2017, 2:00 PM – 2:55 PM

Measuring the Impact of Library Tutorials in an Allied Health Evidence-Based Practice Course

April J. Schweikhard, AHIP, Reference and Instructional Services Librarian, Schusterman Library, University of Oklahoma-Tulsa, Tulsa, OK; **Ken Randall**, Associate Professor and Clinical Director of OU Physical Therapy and Assistant Dean for Academic Affairs for the College of Allied Health, University of Oklahoma Health Sciences Center, College of Allied Health, Tulsa, OK; **Toni Hoberecht, AHIP**, Technical Services Librarian, Schusterman Library, University of Oklahoma–Tulsa; **Alyssa K. Peterson**, Medical Librarian, Schusterman Library at OU-Tulsa, Tulsa, OK

Objectives: This presentation measures how online library instructional tutorials implemented into an evidence-based practice (EBP) course have impacted the information literacy skills of occupational and physical therapy graduate students. Through the assessment of their final course papers, this study compares differences in the quality of students’ search strategies and sources pre- and post-implementation of the library instructional tutorials.

Methods: In 2014, faculty members in a College of Allied Health and an academic health sciences library developed online library instructional tutorials for occupational and physical therapy students enrolled in a graduate level EBP course. These tutorials were embedded in the EBP online course platform and supplemented learning by guiding students through search processes for different types of sources and levels of evidence.

Three student cohorts have completed the Allied Health EBP course since implementing the tutorials. To measure their impact, the authors evaluated the quality of search strategies and sources of evidence within the students’ final written course papers requiring them to apply the five steps of EBP to a clinical case. Using a rubric to assess characteristics of their search strategies and sources, the authors compared these components within papers written before and after the implementation of the tutorials.

Results: Results of this assessment will be made available in the MLA '17 poster onsite and in the ePoster gallery approximately two weeks before the meeting.

Conclusion: The Conclusion will be made available in the MLA '17 poster onsite and in the ePoster gallery approximately two weeks before the meeting.

Keywords: Online tutorials, Information literacy, Evidence-based practice, Allied Health, Instructional assessment

Poster Number: 93

Time: Sunday, May 28, 2017, 2:00 PM – 2:55 PM

Evaluation of Project SHARE, a Youth Health Information Literacy and Leadership Curriculum, Implementation at Six Locations across the Country

Alla Keselman, Senior Social Science Analyst, Specialized Information Services, Bethesda, MD; **Janice E. Kelly, FMLA**, Acting Deputy Associate Director, Specialized Information Services, Specialized Information Services, Bethesda, MD; **Rachel Chase**, National Coordinator, National AHEC Organization, National Training Center, Gainesville, FL; **John C. Scott**, President, Center for Public Service Communications, Center for Public Service Communications, Claiborne, MD; **Gale Dutcher, AHIP**, Retired Acting Associate Director, Specialized Information Services, Specialized Information Services, Bethesda, MD

Objectives: This work tests the implementation of a youth health information literacy curriculum, Project SHARE, developed by health sciences librarians and enacted at six diverse locations across the country. The objective is to evaluate the impact of the curriculum on the youth' understanding of health disparities affecting their community, information evaluation criteria, familiarity with quality health information resources, and leadership skills.

Methods: Project Student Health Advocates Redefining Empowerment (SHARE), sponsored by NLM and developed by University of Maryland Health Sciences and Human Services Library, is a semester-long curriculum teaching youth to use health information for health advocacy. The federal institution partnered with a national health education association to implement the curriculum at six locations, including high-poverty inner city and tribal areas. Each site enrolled 10-25 adolescents ages 13-18. Youth groups met weekly. Throughout the project, site leaders participated in bi-weekly update calls with one another, the national association, and the federal institution. Program evaluation, conducted by the federal sponsor, involved a quantitative and a qualitative components. The quantitative component included pre- and posttests surveys of health information literacy and knowledge. The qualitative component involved talking circles with participants and focused on leadership skills and personal growth.

Results: Overall, participating in the program improved students' understanding of health disparities, health information literacy, and interest in pursuing health careers, as well as communication and leadership skills, but the improvement varied by site.

Conclusion: Health information literacy programs have the potential to improve youth' knowledge, information and communication skills, and interest in community action and health careers. In-depth program evaluation is challenging, but essential.

Keywords: outreach, special populations, health information literacy, community health, youth

Poster Number: 94

Time: Monday, May 29, 2017, 2:30 PM – 3:25 PM

Building Capacity: Information Skills across the Research Lifecycle

Jaime Blanck, AHIP, Lead Informationist; **Maria Truskey**, Informationist; **Anne K. Seymour**, Director; Welch Medical Library, Baltimore, MD

Objectives: As researchers move through the research life cycle they need skills in managing information and data at every stage of the process: planning, implementing, publishing, dissemination/impact, preservation, and re-use. Our objective is to provide training in these skills and aim to broaden our audience to include trainees in developing countries in order to build research capacity.

Methods: We consulted with training program directors to develop a multi-day seminar for our partner institutions in Uganda to provide emerging researchers with the skills to: 1) maximize the value of individual research portfolios to improve funding opportunities, 2) improve literature searching skills 3)

learn to manage literature citations, 4) apply best practices to increase the quality of the systematic review process, 5) develop awareness and understanding of the scientific publishing environment, and 6) increase understanding of the research lifecycle. A secondary goal is to develop a reproducible training program that can be applied at our other partner institutions as well as adopted by health sciences librarians and other professionals involved in health care at other institutions. Initial outcomes of the training program and evaluations by participants will be presented. Future plans to extend the program will be outlined.

Results: Twenty-three individuals participated in the 3.5 day, seven-module course. At the end of each module, course attendees completed a brief evaluation. The composite results show that 54.7% of attendees rated the course content as Extremely Relevant to them, and 44.59% rated the content Very Relevant. The attendees rated the hands-on activities as 66.2% Very Effective and 17.57% as Extremely Effective. 58.11% said they would be Extremely Likely and 37.84% said they would be Very Likely to recommend the training to a professional colleague.

Keywords: research, global health, capacity building, training

Poster Number: 95

Time: Tuesday, May 30, 2017, 1:00 PM – 1:55 PM

Teach New Pharmacy Students a Drug Literature Searching and Evaluation Course Using Online Instruction, Mini Lecture, and Active Learning Techniques

Wendy Wu, Information Services Librarian, Shiffman Medical Library, Shiffman Medical Library, Detroit, MI; **Veronica Bielat**, Education Librarian, Wayne State University, Purdy Kresge Library, DETROIT, MI; **Steven Remenapp**, Graduate Student Assistant, Wayne State University, Purdy Kresge Library, Detroit, MI

Objectives: This poster will discuss the efforts of liaison librarians to re-design and deliver a flipped class on drug literature searching and evaluation collaboratively with faculty so as to strengthen students' ability to answer drug related inquiries and evaluate literature in a systematic manner, to develop core information competencies, and to actively engage students in classroom for better learning outcomes

Methods: A lecture-based Medical Informatics course to 90+ new pharmacy students was switched to a flipped class based on students' course evaluation and preference. The lecture that focused on PICO method and effective search skills was converted into mobile friendly videos and LibGuides. The clinical-scenario-based homework became in-class group activities. Students' comprehension of video content was evaluated through an online quiz completed prior to class. A mini lecture on literature searching and drug resources was given prior to class activities to reinforce important concepts. Students applied the skills obtained to scaffolded class activities. Finding answers to clinical inquiries in scientific literature fosters students' critical thinking and encourages them to apply their knowledge at higher levels of Bloom's Taxonomy. Retention of knowledge was examined and effectiveness of the course assessed using university assessment tools and an online survey.

Results: The result offlipped classroom on the literature searching and evaluation was assessed by comparison of examination and quiz scores, post-course online attitudinal and PubMed video surveys. Comparing to traditional lecture, the students' exam and quiz score demonstrated 9.1 % improvement on finding drug literature to answer clinical scenario questions. 59.8% of 93 students who participated in attitudinal survey indicated they preferred the flipped course and thought active- and team-based class activities combining with mini-lectures helped them put the skills learned prior to the class into action and improved their learning. Average of 70% of 29 students participated in PubMed video survey agreed the videos helped them learn how to formulate search queries for clinical questions effectively, gave them confidence in PubMed searching and assisted their engagement in the related class activities. Students also suggested that LibGuides on drug information and apps were helpful and pressed desire of having access to instructional materials in other formats such as slides or word

documents. Observation of their' performance during class activities confirmed us that they had basic understanding of PubMed features and learned searching skill and strategies, and retained this knowledge and skill through their course activities in winter semester.

Conclusion: Liaison librarians with different expertise in either drug literature searching or education, in collaboration with pharmacy faculty, integrated active learning methods into a pharmacy literature searching and evaluation course and helped enhance students' learning experience and achieve their learning objectives. The outcome of the flipped classroom course encourages librarians to continue seeking collaborative opportunities to provide active learning information literacy instruction to students in academic sittings.

Keywords: Instruction, active learning, flipped class, instruction collaboration, online instruction

Poster Number: 96

Time: Tuesday, May 30, 2017, 2:00 PM – 2:55 PM

Information Skills Training for Health Equity Leadership Pipeline Collaborative

Judith Smith, Informationist, Taubman Health Sciences Library, University of Michigan–Ann Arbor; **Carol Shannon**, Informationist, Taubman Health Sciences Library, Taubman Health Sciences Library, Ann Arbor, MI; ;

Title: Information Skills Training for Health Equity Leadership Pipeline Collaborative

Objective: Student research assistants often look for advanced information-seeking skills to prepare for research. Informationists at the University of Michigan, Taubman Health Sciences Library (THL), partnered with its School of Public Health's "Health Equity Leadership Pipeline Collaborative" (The Collaborative) to create an information training program to prepare student researchers for roles in health equity and eliminating health disparities within healthcare administration.

Methods: THL informationists used a survey approach to curricular design to inform the process of curricular development and implementation. Based on the Collaborative's mission-guided request "to provide innovative, relevant, and scholarly research on health professions and health education training methods to increase the impact of healthcare leadership on addressing health equity", THL informationists began planning for a sustainable information-seeking and management training series for recently hired student research assistants. The program aims were to create a set of training practices that included: 1) *interdisciplinary resource awareness*, 2) *searching subject- focused databases and grey literature*, and *3) use of citation management tools*. As a supplement to the trainings, THL informationists provided hands-on support with using RefWorks, the citation management tool used by research assistants to organize literature sources.

Results: THL informationists developed a multi-part, scaffolded instructional program for research students that incorporates the desired health equity perspective. The four competencies were based on ACRL's Information Literacy Competency Standards for Higher Education, as well as their Framework for Information Literacy for Higher Education: Information Management and Ethical Use, Searching and Documentation, Evaluating Information, and Scholarly Writing and Publishing. For each component, there are three mastery levels with specific learning objectives, which provide demonstrable skills for evaluation. It is designed with enough flexibility to allow students to begin with any component if needed.

Conclusion: In discussions with both the Collaborative and THL, three overarching training goals have been identified: preparation for research, publishing, and grant-writing. To achieve these goals, our program will focus on information skills education related to those tasks for its student research assistants. THL informationists will develop and implement these training tools and will evaluate their impact.

Keywords: Training, collaboration, partnership, health equity, health disparities, leadership, diversity

Poster Number: 97

Time: Sunday, May 28, 2017, 2:00 PM – 2:55 PM

Two Nursing Liaisons-One-Team: A Collaboration Experience

Marita Malone, AHIP, Medical Librarian, SOM Library, Rowan University School of Osteopathic Medicine, Stratford, NJ; **Samantha Kennedy**, Librarian, Rowan University, Campbell Library, Glassboro, NJ

Objectives: In an evolving academic landscape, universities are being combined with other schools and institutions, creating unique situations. In an effort to ensure nursing library patrons were aware of library resources during the merger of Rowan University and Cooper Medical School of rowan University (CMSRU), nursing liaisons at each campus collaborated during the first year to provide a seamless transition.

Methods: To ensure collaboration and effective communication, the two liaisons met monthly and maintained regular email contact to discuss new and ongoing issues. Areas of concern included: weeding, purchasing new resources (print and electronic), an accreditation committee site visit, transition of workloads and duties and the addition of a third nursing program to the libraries.

Results: Recognizing the need for collaborative efforts and the importance of professional development opportunities, the nursing liaisons enrolled in a MOOC, shared resource articles and information and attended professional meetings together. Because of their constant communication, the liaisons were immediately aware of confusion to nurses and nursing students regarding available resources in their programs and libraries. Separate but similar LibGuides were created and placed in each institutions learning management systems and websites.

Conclusion: The template created by the nursing liaisons enabled each campus or nursing program a cohesive LibGuide base which provided the option for campus customization. Collaboration success was measured by positive faculty and student/nurse feed back, usage statistics, and a deeper appreciation for new colleagues on different campuses.

Keywords: Collaboration, Nursing, Transitions, Communication, Merger, Liaison, Teamwork

Poster Number: 98

Time: Monday, May 29, 2017, 2:30 PM – 3:25 PM

American Association of Colleges of Pharmacy (AACP): Value of Participation for Academic Pharmacy Liaison Librarians

Rae Jesano, AHIP, Reference and Liaison Librarian, Health Science Center Libraries, Health Science Center Libraries, Gainesville, FL

Objectives: The purpose of this poster is to examine the value of membership in the American Association of Colleges of Pharmacy (AACP) and annual meeting attendance for librarians who liaise to colleges or schools of pharmacy. It will discuss the results of a survey of academic pharmacy librarians and related pharmacy faculty on the value of librarian membership in this organization.

Methods: A survey created on Qualtrics and approved by the Institutional Review Board will be sent to members of Pharm-Lib and AACP’s Library and Information Science Section email distribution lists. The survey will solicit opinions from librarians who work with schools of pharmacy on the effect of attending AACP’s meeting and participating in the Association on their professional practice and their interaction with pharmacy students and faculty members. It will also ask about what effects, if any, it has had on their careers. Since these lists also include pharmacy professionals who are interested in knowledge-

based information, the survey will use branch logic to question them specifically on their opinion of librarians attending this pharmacy meeting and its perceived effect on academic pharmacy.

Results: My results will be made available onsite in my poster and in the ePoster gallery

Conclusion: My conclusions will be made available onsite in my poster and in the ePoster gallery

Keywords: Liaison librarians, academic librarianship, subject specialist, association membership, pharmacy librarianship

Poster Number: 99

Time: Tuesday, May 30, 2017, 1:00 PM – 1:55 PM

Why Are Liaison Librarians so Busy?

Chun-Ching Liang, Director, NTU Medical Library, National Taiwan University Medical Library, Taipei, Taipei, Taiwan (Republic of China); **Chris Hwang**, Librarian, National Taiwan University, National Taiwan University Medical Library, Taipei, Taipei, Taiwan (Republic of China); **Yi-Hsin Lu**, Librarian, National Taiwan University Hospital, National Taiwan University Medical Library, Taipei, Taipei, Taiwan (Republic of China); **Ying-Fong Jheng**, librarian, National Taiwan University Medical Library, Taipei, N/A, Taiwan (Republic of China); **Chen-jung Huang**, Librarian., National Taiwan University Medical Library, Taipei, Taipei, Taiwan (Republic of China)

Objectives: This research aims to explore how the library formed the organization of liaison librarians and the target community. Our purposes:

1. To increase the visibility of library.
2. To build and enrich the communication platform, Academic Hub.
3. To strengthen the connection between library and academic departments
4. To help library and librarian fit in the scholar community

Methods: Data collection based on qualitative research method is completed through in-depth interview. Through the interview, this research tries to present the transformation of NTU Medical Library service, the change of liaison librarians' service strategy and mental state, and the current state of Academic Hub system with the promotion of subject service.

1. Team work: working as full-time librarians and sparing some of their time to proceed subject services, each member has his/her own expertise. With the strategical service, group discussion, sharing experiences, and teachers' feedback, the liaison librarians can become multi-dimensional librarians.
2. Walk out of the library and walk into the clinical office and study room: on-site service.
3. Make good use of impact of opinion leaders.
4. Find clinical staff and researchers' need and provide flexible, personal and customized services.

Results: The results show that after more than one year of effort by the liaison librarians,

1. teachers' participation rate of Academic Hub rises to 90%.
2. liaison librarians provide more customized services and on-site services than ever to all departments and clinical offices.
3. library gains highly affirmation and evaluation in the accreditation of parent organization.
4. the subject services substantially enhance image and visibility of library.

Conclusion: Through the subject services, librarians are able to host multiple promotional activities by walking out of library, practically participating in the academic research of parent organization, and making a face-to-face introduction to the library services and resources instead of merely promoting activities through posters and Internet. Although it causes an increase in the workload, librarians have

the chance to build a close connection and deepen the interaction with teachers and medical staffs in the aspect of teaching and research. Also, librarians gain a lot of practical support and spiritual encouragement. For example, some teachers and medical staffs take development of library very seriously and help fundraise for the library. Through this reciprocal process, liaison librarians build self-confidence, increase their passion, have more smiles, and make library services more accessible to the patrons.

Poster Number: S1

Time: Sunday, May 28, 2017, 2:00 PM – 2:55 PM

Cancer Librarians Section Poster

Section Chapter ePosters are not staffed during the meeting, and will be viewable in the Registration Area throughout the Meeting, and available in the online ePoster gallery. Look for the kiosk in the MLA Connection Area located within the Registration Center to view the posters onsite.

Poster Number: S2

Time: Sunday, May 28, 2017, 2:00 PM – 2:55 PM

Collection Development Section Poster

Section Chapter ePosters will be viewable in the Registration Area throughout the Meeting, and available in the online ePoster gallery. Look for the kiosk in the MLA Connection Area located within the Registration Center.

Poster Number: S3

Time: Sunday, May 28, 2017, 2:00 PM – 2:55 PM

Consumer and Patient Health Information Section Poster

Robin O'Hanlon, Assistant Library Director, Outreach and Public Services, CAPHIS, Chair-Elect, Levy Library, Icahn School of Medicine at Mount Sinai, New York, NY

Section Chapter ePosters will be viewable in the Registration Area throughout the Meeting, and available in the online ePoster gallery. Look for the kiosk in the MLA Connection Area located within the Registration Center.

Poster Number: S4

Time: Sunday, May 28, 2017, 2:00 PM – 2:55 PM

Dental Section Poster

Dental Section, Dental Section, MLA Dental Section, Los Angeles, CA

Section Chapter ePosters will be viewable in the Registration Area throughout the Meeting, and available in the online ePoster gallery. Look for the kiosk in the MLA Connection Area located within the Registration Center.

Poster Number: S5

Time: Sunday, May 28, 2017, 2:00 PM – 2:55 PM

Educational Media and Technologies Section Poster

Section Chapter ePosters will be viewable in the Registration Area throughout the Meeting, and available in the online ePoster gallery. Look for the kiosk in the MLA Connection Area located within the Registration Center.

Poster Number: S6

Time: Sunday, May 28, 2017, 2:00 PM – 2:55 PM

Health Association & Corporate Libraries Section Poster

Section Chapter ePosters will be viewable in the Registration Area throughout the Meeting, and available in the online ePoster gallery. Look for the kiosk in the MLA Connection Area located within the Registration Center.

Poster Number: S7

Time: Sunday, May 28, 2017, 2:00 PM – 2:55 PM

History of the Health Sciences Section Poster

History of the Health Sciences Section, Melissa Nasea, Chair, Mary Helms, Chair-Elect, NA, N/A

Section Chapter ePosters will be viewable in the Registration Area throughout the Meeting, and available in the online ePoster gallery. Look for the kiosk in the MLA Connection Area located within the Registration Center.

Poster Number: S8

Time: Sunday, May 28, 2017, 2:00 PM – 2:55 PM

International Cooperation Section Poster

Section Chapter ePosters will be viewable in the Registration Area throughout the Meeting, and available in the online ePoster gallery. Look for the kiosk in the MLA Connection Area located within the Registration Center.

Poster Number: S9

Time: Sunday, May 28, 2017, 2:00 PM – 2:55 PM

Medical Informatics Section Poster

Medical Informatics Section, Medical Informatics Section (MIS), Medical Library Association, Chicago, IL

Section Chapter ePosters will be viewable in the Registration Area throughout the Meeting, and available in the online ePoster gallery. Look for the kiosk in the MLA Connection Area located within the Registration Center.

Poster Number: 100

Time: Tuesday, May 30, 2017, 1:00 PM – 1:55 PM

Mapping Liaison Librarian Areas of Responsibility in Four Professional Schools and Professional Competencies to Support Research Consultations

Xan Goodman, AHIP, Health & Life Sciences Librarian, Assistant Professor, University Libraries, University Libraries, UNLV, Henderson, NV

Objectives: This poster will describe two mapping processes. First mapping the various degree programs, certificates and other specialized units for four professional schools: School of Nursing, School of Allied Health Sciences, School of Community Health Sciences and School of Dental Medicine. Secondly mapping skills required to support research consultations for each professional school is explored.

Methods: This poster will describe multiple liaison areas of responsibility using a visual mapping tool to map degree programs, certificates, and other affiliated departments including specialized units in each area of responsibility. This poster will analyze themes of research consults with visual mapping to describe topics covered during research consults conducted from 2015-2016. Also skills or competencies required to support these research consults will be explored with visual mapping. The Medical Library Association areas of Professional Competency will used to demonstrate areas of librarian competency mapped to research consult themes. Librarians who are responsible for multiple liaison areas coupled with emphasis on in-depth research support might benefit from an overview of the effectiveness of recommended Professional Competencies and how they aid in meeting the research support needs of the schools represented in this poster.

Results: Final results will be available at the annual meeting.

Conclusion: Final results will be available at the annual meeting.

Keywords: MLA professional competencies, research consults, consultations, skills, themes, students, faculty

Poster Number: 101

Time: Sunday, May 28, 2017, 2:00 PM – 2:55 PM

The Librarian in the Lunch Room: A Pilot Project for Office Hours in the Department of Anesthesiology

Ricardo Andrade, Assistant Director for Information Resources, Columbia University Augustus C Long Health Sciences Library, New York, NY

Objectives: The objective of this presentation is to discuss how to implement office hours, marketing methods, and the types of potential questions received. The purpose of the pilot project was to implement office hours in the physician lounge of the Department of Anesthesiology in order to promote library resources, and to make the department aware of support that librarians could provide.

Methods: The presentation will discuss the results of the pilot project. The project, which lasted from November 2016 through June 2016, was a collaboration between a librarian and the chair of the department of Anesthesiology and Critical Care. The project consisted of having office hours in the

physician lounge where Anesthesiology residents and physicians ate lunch and took breaks. The goal of the office hours was to promote the librarian as the librarian for the department, put a face to the name, and to make the department aware of support that the librarian could provide. The presentation will talk about the types of questions received, what discussions occurred during the office hours, marketing messages used, steps that were taken to implement the office hours and discuss who were the highest user of the service.

Results:

Keywords: Office hours; liaison; anesthesiology; residents; outreach

Poster Number: 102

Time: Monday, May 29, 2017, 2:30 PM – 3:25 PM

Librarians: Part of the Physician Assistant (PA) Education Team?

Brandi Tuttle, AHIP, Research & Education Librarian, Medical Center Library & Archives, Duke University, Durham, NC

Objectives: In conjunction with the University Physician Assistant (PA) Program's Program Director, the PA liaison librarian conducted a survey sent to both PA faculty and PA liaison librarians across the nation to learn more about the typical types of library support received from and collaborations embarked with their institution's library and librarians.

Methods: Survey results gave insight on the many ways PA faculty currently collaborate with librarians and use library services. PA faculty and librarians slightly differed in how they thought librarians were used and many reported not using a librarian/library to the fullest extent. New ways to incorporate the librarian into a PA program include: course embedding, technology assistance, grant writing support, faculty research and impact tracking, publishing and scholarship guidance, committee service, and accreditation assistance. At the Physician's Assistant Education Association (PAEA) 2016 Education Forum, this topic was further explored with PA faculty. More data was collected in real time through a survey and audience comments on the many ways PA educators can partner with a librarian to build faculty professional capacity and expertise as well as save time and improve scholarly efficiency.

Results: Survey results and associated comments from both PA faculty and liaison librarians across the nation will be shared as well as next steps and suggestions. The insightful PA faculty perceptions of librarian contributions as well as areas where librarians could move into will be highlighted as well as the many challenges and opportunities that exist for many PA programs and librarians.

Conclusion: While institutions differ greatly in how a PA program is structured and supported by the library, there is ample room for liaison librarians, or those supporting PA programs, to increase the level of involvement based on student and faculty needs.

Keywords: PA, physician assistant, liaisons, embedded

Poster Number: 103

Time: Tuesday, May 30, 2017, 1:00 PM – 1:55 PM

Serving Community Faculty through a Dedicated Library Services Liaison

Katie Jefferson, Library Services Liaison, University of Nevada, Reno School of Medicine, Savitt Medical Library, Reno, NV; **April Heiselt**, Director, Office for Community Faculty, University of Nevada, Reno School of Medicine, Office of Academic Affairs, Reno, NV; **Mary Shultz**, Director, University of Nevada, Reno School of Medicine, Savitt Medical Library, Reno, NV

Objectives: Community-based medical schools often do not have an integrated teaching hospital and instead partner with community hospitals. These schools rely on community faculty, sometimes referred to as volunteer or adjunct faculty, to provide lectures, preceptorships, clinical training, and more. This program details one library's embedded service for these important faculty members.

Methods: Community faculty are clinicians whose main role is patient care. Some have limited experience with instruction and other aspects of their faculty role. Many are unfamiliar with the multitude of university resources and services available to them.

To address this unmet need, the Savitt Medical Library at the University of Nevada, Reno (UNR), partnered with the UNR School of Medicine's Office for Community Faculty to provide dedicated and embedded services to the School's growing ranks of community faculty. In 2016, the position of Library Services Liaison was created to serve these faculty from a central office within the city's largest hospital. This program began by providing literature searching, reference, and instruction. Other faculty needs were quickly discovered and the types of support were expanded. This service has been successful and may be extended to other community hospitals.

Results: The Library Services Liaison to the community faculty has provided reference, instruction, literature searching, and other traditional services. Additionally, she has supported some faculty in their development of lecture materials and in engagement through support of community faculty events. During a sixth month period (July-December 2016), she answered approximately 70 questions per month which included directional, ready reference, in-depth, and research consultations. Most were in-person during her scheduled hours at the hospital, but roughly 18% were by email.

Conclusion: Having a liaison that specifically serves community faculty has extended the library's service to this important group. Her presence and support has also aided in the marketing of library resources and services. This effort has resulted in a further partnership and creation of an academic library space within the hospital to support community faculty, residents, fellows, and students.

Keywords: Volunteer, adjunct, faculty, Nevada, hospital, clinical library, liaison, library services

Poster Number: 105

Time: Sunday, May 28, 2017, 2:00 PM – 2:55 PM

The MLA Voter: Results of a Survey Exploring MLA Voter Attitudes, Perceptions, and Voting Practices

James Shedlock, AHIP, FMLA, Consultant/retiree, MLA Fellow; Distinguished Member, AHIP, Chicago, IL

Objective: The survey's research goal seeks insight into the MLA voting process by measuring opinions regarding elections for President, Board of Directors, and Nominating Committee. This survey measures voting issues such as the qualities of candidates; reasons for non-voting; preferences for a single slate; campaigning; the nomination process; etc.

Methods: Survey Monkey is the instrument of choice and email distribution was used exclusively. Invitations were sent to 2,671 MLA members eligible to vote and 676 surveys were received, a 25% return rate.

Results: Survey demographics include: 20% of respondents came from the Midwest Chapter; 83% of respondents are individual MLA members; respondents are in the 45-64 age bracket (53%); 40% of respondents have 26 or more years of work experience; 85% of respondents are female; 90% work fulltime; and 60% are employed in an academic work environment.

Respondents said the qualities they look for in a MLA presidential candidate are vision for MLA (83%) and the amount and kind of MLA experience (78%). For Board of Directors candidates, the qualities sought are amount and kind of MLA experience (83%) followed by a vision for MLA (73%). Qualities sought for members of the Nominating Committee are amount and kind of MLA experience (76%) and amount of library experience (53%).

Personal qualities such as gender (47%), race (58%), sexual orientation (81%) or marital status (95%) are almost never considered. Professional qualities such as institutional affiliation (43%) or regional affiliation (35%) are sometimes considered.

In terms of voting issues such as the use of a single slate for national elections, respondents indicated yes by 20%; 35% polled no and 44% said not sure. 67% of respondents say they always vote in MLA national elections. Only 16 respondents indicated that they never vote in MLA elections. Almost all respondents indicated that voting was both a member benefit (77%) and a member responsibility (93%).

In terms of campaigning for MLA national elections, over 82% say they do not campaign. 52% agree with the MLA tradition of no campaigning but 39% are not sure. 54% are not sure whether campaigning would have a positive or negative effect on MLA national elections. 48% of respondents say the MLA Board should define controls for campaigning and 41% are not sure.

Conclusion: Understanding the MLA voter's perceptions has the potential for revealing new insights into association elections and will be useful to future Nominating Committees and future candidates.

Keywords: MLA voting; voting attitudes; voting perceptions; voting practices; elections; voting process; candidates; slates

Poster Number: 106

Time: Monday, May 29, 2017, 2:30 PM – 3:25 PM

DARE to CHANGE: One MLA Chapter's Dream of Evolution

Connie K. Machado, AHIP, Associate Director / Associate Professor, Rowland Medical Library, Rowland Medical Library/Univ. of Mississippi Med. Cntr., Jackson, MS; **Elizabeth G. Hinton, AHIP**, Reference Librarian, Rowland Medical Library, University of Mississippi Medical Center, Jackson, MS; **Tara Douglas-Williams**, Division Head for Information Services/Library Manager, M. Delmar Edwards, M.D. Library, Morehouse School of Medicine, Atlanta, GA

Objectives: A chapter of the Medical Library Association (Chapter) continues to struggle with annual meeting expenses without utilizing Chapter funds. The goal of this initiative is to incorporate cost-effective changes into the Chapter's annual meeting planning, schedule, and objectives; provide the best return on investment for attendance by members; and determine new management practices and dues structures for the Chapter.

Methods: The Chapter will survey all other MLA Chapters to determine the following: Annual Meeting format; website functionality (specifically, questions about membership directories, dues payment process and annual meeting registration); dues structures; and questions concerning Chapter leadership/management. Survey data will be analyzed and resulting report will be distributed to the Chapter's Executive Committee to facilitate discussion and aid in evaluation of current Chapter practices. Results will aid the Chapter in making decisions toward redesigning the annual meeting format, content, and costs to better serve membership needs, while also decreasing the overall annual meeting expenses.

Keywords: fiscal responsibility; chapter expenses; chapter annual meetings; budget; survey

Poster Number: 107

Time: Sunday, May 28, 2017, 2:00 PM – 2:55 PM

Dreaming to Learn the Socious Platform, the Hospital Libraries Section Dares to Follow the Educational Media and Technologies Section

Ellen Aaronson, AHIP, Medical Librarian, Medical Library, West Hills Hospital & Medical Center, West Hills, CA; **Lisa Marks, AHIP**, Director of Libraries, Mayo Clinic, Staff Library, Scottsdale, AZ; **David Duggar, AHIP**, Head, Library Liaison Program, Health Sciences Library, LSU Health Shreveport, Shreveport, LA

Objectives: Describe the process undertaken by a team of librarians conducting a website marketing campaign designed to make section members more aware of features of the website. Goals include providing an engaging "game" for members to learn more about their professional community, obtaining feedback about how they feel about the section, and offering a forum for discussion to encourage section involvement.

Methods: The team of librarians created a sequence of daily activities based on a model designed by another section. Permission to use the model was obtained from that section. Daily instructions for the website activities were posted on the section website and emailed to the listserv. Those completing the daily activity could enter to win a gift card by answering an online survey. Activities included asking members to add a photo and create a bio for their profile, post a message to the section Forum, and update their subscriptions indicating how they would like to receive section mail. A brief online survey will be utilized to collect feedback on how members feel about the section, its value, ease of navigation on the website, preferred methods of communication, and how the section can best meet members' needs.

Results: LimeSurvey was used to administer and analyze the survey data. Approximately 550 HLS members received the daily email with a total of 30 participating over the 10 days and completing the survey. One-third of those performed at least one activity while two-thirds did two or more activities. 10 winners were selected by an independent party at the end of the time period, and they each received a $25 Amazon gift card.

Conclusion: The librarian team was surprised at the low participation however the feedback received is quite valuable. Participants reported learning a lot about the HLS website and MLAnet. Members enjoyed the "game" format and specifically found activities relating to receiving messages, connecting with other members and changing their profiles very relevant. One even identified some revisions that were needed on a form!

Keywords: Socious, MLAnet, Hospital, Libraries, Section, Educational, Media and Technologies, Section, Professional, Community, Games

Poster Number: 108

Time: Tuesday, May 30, 2017, 2:00 PM – 2:55 PM

Development of a Mentoring Program for 21st Century Library Leaders within New and Developing Medical Schools: Process and Outcome

Joanne M. Muellenbach, AHIP, Founding Director, Health Sciences Library, UNLV Libraries, University of Nevada, Las Vegas , Las Vegas, NV; **Nadine Dexter, AHIP**, Director Harriet F. Ginsburg Health Sciences Library, Harriet F. Ginsburg Health Sciences Library, Harriet F. Ginsburg Health Sciences Library, Orlando, FL; **Debra Rand, AHIP**, Associate Dean for Library Services, Health Sciences Library, Hofstra Northwell School of Medicine, Hempstead, NY

Objectives: The Association of Academic Health Sciences Libraries (AAHSL) New and Developing Academic Health Sciences Libraries - Mentoring Program has been established to provide a method of introducing and encouraging library directors and other library leaders within new and developing medical schools to work together and network within the community. Mentors support library leaders in learning about their new roles and guide mentees in their future goals.

Methods: The Association of Academic Health Sciences Libraries (AAHSL) New and Developing Academic Health Sciences Libraries Committee receives numerous requests for consultations from

library leaders within new and developing medical schools. To learn more about their needs, a needs assessment was distributed to library directors and leaders within 21st century medical schools. Results indicated that library leaders seek support in the areas of accreditation, budget, collections, information literacy / evidence-based medicine instruction, space planning, and staffing. To address these needs, the Committee recommended the development of an AAHSL Mentoring Program. Following AAHSL Board review and approval, the AAHSL Mentoring Program was established. An overview of the AAHSL Mentoring Program, including application forms, links to the New and Developing Health Sciences Libraries LibGuide, and to a series of articles on "Born Digital" libraries, published in MLANews, may be found on the AAHSL website, located at: www.aahsl.org.

Results: they will be made available during my poster session at MLA '17.

Conclusion: they will be made available during my poster session at MLA '17.

Keywords: Association of Academic Health Sciences Libraries (AAHSL), Born Digital Libraries, Leadership, Mentoring, New Medical Schools

Poster Number: 109

Time: Sunday, May 28, 2017, 2:00 PM – 2:55 PM

User-Centered Approach to an Advanced Information Literacy Programme in the E. Latunde Odeku Medical Library

Toluwase V. Asubiaro, Librarian II, Systems Unit, E. Latunde Odeku Medical Library, College of Medicine, University of Ibadan, Nigeria, London, ON, Canada

Objectives: This paper describes the user-centered approach used at the E. Latunde Odeku Medical Library (ELOML), University of Ibadan, Nigeria in delivering information literacy (IL) programme and other library services to the library patrons during the renovations of the library building when there was no alternative building for delivery of library services.

The main objective of the user-centered programme was to provide a way of providing library services to the library patrons despite the fact there was no library building. Achieving this aim was important because of the importance of ELOML to medical education in Nigeria.

Methods: Information literacy programme tagged user-centered librarianship was executed during the renovations of the ELOML library building between May 2014 and May 2016. The user-centered librarianship programme was executed by taking the library services to the library patrons in the comfort of their offices and classrooms through training and workshops for patrons on electronic services and products. Emphasis on electronic services and resources became important because the print resources were not accessible. The first sets of trainings were based on topics that were identified from patrons' inquiries from the library over a period of time. The user-centered programme was marketed using flyers, banners and word of mouth. Contents were developed for the identified topics with the aim of empowering patrons to use e-resources and services of the library effectively. The heads of departments were written formal letters with a request from the library to deliver training for staff and graduate students on the identified topics at the chosen time and venue by each department. The IL programme targeted lecturers, researchers graduate students and resident doctors. Participants in the first user-centered library programme were asked to list topics of interest that subsequent trainings on information literacy should address. The list of topics of interest was used for developing topics for subsequent trainings. The social media platform (Facebook) was meant to complement the face-to-face classes. Some of the topics covered included information search strategies, plagiarism, reference management using software, e-resources in ELOML and library orientation.

Results: First, the library was active and able to remain viable even though there was no building for delivery of library services. Second,

Conclusion: User-centered librarianship is a new paradigm which offers dynamism in librarianship and makes it possible to customise solutions to challenges faced in delivery of library services.

Keywords: Advanced Information Literacy, Medical Library, user-centered librarianship

Poster Number: 110

Time: Monday, May 29, 2017, 2:30 PM – 3:25 PM

Promoting Professionalism within a Health System Library Department

Heather J. Martin, AHIP, Director, System Library Services, Providence Health & Services, Portland, OR; **Isaac Huffman, AHIP**, Library Director, City of Mount Vernon, WA, Olympia, WA

Objectives: To increase the level of professionalism within a large health system library department and encourage a culture of continual professional development.

Methods: Hospital library staff often lack the traditional avenues to support professional growth. Providence library leadership aimed to increase professionalism within the department through hiring, promotion, and support of Academy of Health Information Professionals (AHIP) credentialing. Hiring and promotion initiatives included standardizing job descriptions across the health system, transferring vacant library paraprofessional positions into librarian positions, and promotion of staff members who had or were pursuing masters degrees. Support of becoming or moving up levels of AHIP membership included providing at work time for applications, publication support, finding and prompting continuing education as well as providing dedicated funds for application fees. The program was appraised via employee feedback, publication, and credential tracking.

Results: In 2016, 6 out of 7 eligible staff held, received or renewed Academy of Health Information Professional Status. Library staff published 1 scholarly paper, and presented 1 paper, and 3 posters at national and regional conferences. In 2017 Library staff were accepted to present 4 posters, 1 paper, and 1 lightening talk at the 2017 MLA Annual Meeting and 2 Staff members are serving in leadership roles in regional MLA chapters.

Staff gave strong feedback that the library was doing a better job of supporting their professional development and the library benefited from the work completed as part of these professional development exercises including new pathways, tools, and approaches to library services. These changes also played a part in growing library service by nearly 27% over the past year (2015 to 2016).

Conclusion: Working for a non-academic or small education institution there are often few organizational structures that support the development of staff. Therefore, internal department structures, peer support, and leading by example become important motivators in developing a professional staff. Our experience indicates that with a few changes including achieving personal development milestones, internal praise, allocating time and resources continuing education, and clearly placing staff development in departmental strategic priorities that a non-academic employer can maintain a high standard of professional practice and participation.

These professional practice standards, in turn, benefit the department in a number of ways most notably in practice advancement and service growth. These investments also provided a good return for the organization costing only 0.3% of the total library budget while netting a service growth that would have cost much more utilizing traditional marketing outlets.

Keywords: AHIP, professionalism, management

Poster Number: 111

Time: Tuesday, May 30, 2017, 1:00 PM – 1:55 PM

Back to the Basics! Career Development for Early Career Librarians through Subject-Intensive Conferences

Natalie Logue, Access Services Librarian; **Ansley Stuart, AHIP**, Allied Health Sciences Information Librarian; Augusta University, Robert B. Greenblatt, M.D. Library, Augusta, GA

Objectives: To assess a subject intensive conference for career development benefits among librarians with less than 5 years of professional experience.

Methods: Two early-career librarians who attended the Science Bootcamp for Librarians Southeast 2016 assembled an overview of the conference content, professional opportunities, and the impact it had on their careers. To further investigate the outcomes of the conference, they developed a survey for conference attendees asking them to analyze if they had a better understanding of topics, methodology, critical thinking, and professional connections after conference than they did before and sent it out via email four months after the conference. This data was grouped into three career stages for comparison: Early career, mid-career, and late career.

Results: Will be made available during the poster presentation at MLA '17.

Conclusion: Will be made available during the poster presentation at MLA '17.

Keywords: continuing education, early-career librarians, professional development, science bootcamp

Poster Number: 112

Time: Tuesday, May 30, 2017, 2:00 PM – 2:55 PM

Mapping Health Professions Education Program Standards to Library Competencies

Megan G. Van Noord, Research & Education Librarian, Duke University Medical Center Library & Archives, Durham, NC; **Leila Ledbetter**, Librarian, Medical Center Library & Archives, Duke University Medical Center Library, Durham, NC; **Emily Mazure, AHIP**, Biomedical Research Liaison Librarian, Medical Center Library & Archives, Duke University, Durham, NC; **Brandi Tuttle, AHIP**, Research & Education Librarian, Medical Center Library & Archives, Duke University, Durham, NC; **Megan von Isenburg, AHIP**, Associate Director, Research and Education, Medical Center Library & Archives, Medical Center Library & Archives, Durham, NC; **Jamie Conklin**, Research & Education Librarian, Duke University, Duke Medical Center Library, Durham, NC

Objectives: Our objective was to assess the role of librarians in meeting programmatic competencies required for our university's health education programs' standards and accreditation. We also sought to identify additional elements in curricula that could benefit from library involvement and to discover core themes that we could translate into specific competencies for students to drive future library instruction and assessment.

Methods: Health professions students need to navigate their information needs and to be persistent, curious, comfortable with ambiguity, critical and open minded when doing so. Individual program standards (e.g., LCME Accreditation Standards and Elements) recognize these student needs and include statements that address them. Librarians, working as subject liaisons to specific disciplines and engaged in health professions educational programs, are equipped to teach students in these areas. We collected specific program objectives, accreditation requirements, and professional society frameworks under which each program operates, noting all program standards in which our liaisons either currently play or potentially could play a role. We compared standards across programs, identified the major focus areas for library instruction and services, and created a set of core information resource competencies for all health professions students at our university.

Results: Health profession educational standards relate to library support in four key areas: 1. lifelong learning, 2. research and scholarly communication, 3. evidence-based practice, and 4. the domain of the library. All programs have standards that explicitly stated evidence-based practice competencies, indicating the potential for interprofessional curricular efforts addressing students' abilities to locate, appraise, and apply the best evidence.

Conclusion: Comparing established library instruction and services to existing program standards and goals enabled us to identify current and future key contributions of the library for meeting student learning goals. Next steps include exploring more instruction on scholarly communication issues like open access, metrics, and copyright, as well as using drafted competencies for assessment purposes.

Keywords: Curriculum, Health Professions Education, Accreditation, Liaisons, Library Instruction & Services

Poster Number: 113

Time: Sunday, May 28, 2017, 2:00 PM – 2:55 PM

Recounting the Successful Pilot of the MLA International Cooperation Section International Visiting Health Information Professionals Program

Sandra McKeown, Health Sciences Librarian, Health Sciences Library, Queen's University, Kingston, ON, Canada; **Chao Yu Wu**, Librarian, National Taiwan University, Medical Library, Taipei, Taipei, Taiwan (Republic of China)

Objectives: The International Visiting Health Information Professionals Program was developed to "…foster international cooperation, communication, awareness, exchange of ideas, and professional excellence" [1]. This poster describes the activities, outcomes and evaluation of the program's first match from the perspectives of both the visiting librarian and host institution.

Methods: Bracken Health Sciences Library at Queen's University in Kingston, Ontario, Canada hosted a librarian from the National Taiwan University Medical Library over a 4-week period in September and October of 2016. After the applicants were officially matched, the host institution helped arrange living and travel accommodations. The host preceptor and international librarian communicated via email and Skype prior to the visit to introduce themselves and coordinate details of the visit. The preceptor and visiting librarian worked together to develop a schedule of meaningful activities that would be beneficial for the visiting librarian and host institution.

Results: The visiting librarian met with each of the health sciences librarians and staff to learn about their individual and shared roles and activities. A number of health sciences library activities were observed by the visiting librarian, including: team meetings, librarian reference shifts, individual and group consultations with a librarian, library tours, library sessions for undergraduate and graduate students in medicine, nursing, and rehabilitation therapy, and the marking of information literacy assignments. The visiting librarian met and heard from international colleagues during the Library Day portion of the Queen's Joanna Briggs Collaboration Conference of the Americas that took place in Kingston. Outside of the health sciences library setting, tours were arranged for the Anatomy Museum at Queen's and the Museum of Health Care. The visiting librarian also met with non-health sciences librarians to tour other libraries at Queen's University in order to understand the broader university library system. Highlights of the visit were prepared by the visiting librarian and broadly communicated via weekly posts to the library's Facebook page.

Outcomes: The visiting librarian and host preceptor provided evaluation feedback to the head of their respective libraries and to the Chair of the MLA/ICS IVIP International Visiting Health Information Professionals Program Task Force. The international librarian and the host institution's librarians exchanged information and ideas to gain valuable insight into the medical library profession from an international viewpoint. The international librarian was able to share their experience and ideas with colleagues back home to help enhance library services at the National Taiwan University Medical Library.

Poster Number: 114

Time: Monday, May 29, 2017, 2:30 PM – 3:25 PM

Veterans Affairs Librarian Chief/Manager Mentoring Program: Creating a Support System for Our New Leaders

Dorothy Pamer. Sinha, AHIP, Chief, Library Service, Minneapolis VA Healthcare System, Minneapolis VA Medical Center, Minneapolis, MN; **Nancy A. Clark**, Director, Library Network Office, Library Network Office, Department of Veterans Affairs, Dallas, TX

Objectives: The mentorship program is chaired by an Associate Chief of Staff for Education in collaboration with the Library Network Director. There are six experienced Library Managers across the country that mentor new VALNET Chiefs/Managers . Mentoring sessions typically last 2 1/2 days and take place at the site of the mentee.

Methods: VALNET includes library staff at over 120 medical centers throughout the U.S. In order to maintain a strong network of Library staff nationwide, we depend on Librarian Chiefs/Managers having the knowledge and tools necessary to competently direct their programs. The mentoring program provides these new leaders the training and guidance needed to be successful in their new roles. This poster describes the objectives, curriculum, processes, challenges, evaluation and outcomes of the program.

Results: Mentees find the program extremely helpful in learning about the VA Healthcare System, getting up to speed on the knowledge and tools required to operate a quality program, and networking with key individuals in VALNET. Mentors benefit by meeting new Librarian Chiefs/Managers and their supervisors and seeing a variety of network libraries. The mentor/mentee relationship continues after the initial 2 1/2 day mentoring session ensuring that new Chiefs/Managers continue learning and have a trusted colleague to assist with questions and issues as they arise. The program strengthens VALNET overall by establishing and maintaining strong relationships among library staff throughout the VA.

Conclusion: Mentors have informal conversations before the mentoring sessions to assess mentee background and learning needs. Post-mentoring surveys assess the mentee's satisfaction with the mentoring session. The survey includes satisfaction ratings using both a Likert scale and free text which allows the mentee to provide feedback about the mentor and the program in general. Results are reviewed by the Program Chair and the Library Network Director. Suggestions for changes are brought to the group for discussion and possible implementation.

Keywords: Mentoring, Library management

Poster Number: 115

Time: Tuesday, May 30, 2017, 1:00 PM – 1:55 PM

Paraprofessional to Professional: Dreaming, Daring, Doing

Sydni L. Abrahamsen, AHIP, Librarian, Patient and Health Education Library, Mayo Clinic, Scottsdale, AZ

Objectives: This poster discusses one librarian's experience making the transition from paraprofessional staff at an academic research library to librarian at a consumer health library in a medical center.

Methods: The poster will include information on the similarities and differences in academic and consumer health library work, my experiences in transitioning from one role to another, some challenges faced when making this type of career move, and describe some of the advantages an academic library background brings to consumer health librarianship. It will also include some preparatory steps one can take to prepare for successfully transitioning from paraprofessional to professional and/or from an academic to medical setting.

Conclusion: At the time of MLA ’17, the presenter will have been in this position for nine months and will be able to share some of the successes and missteps in navigating this new role.

Keywords: job searching, consumer health, academic, transition, preparation

Poster Number: 116

Time: Tuesday, May 30, 2017, 2:00 PM – 2:55 PM

Utilization of Intentional Reflective Practice among Health Sciences Librarians

Jolene M. Miller, AHIP, Director, Mulford Health Sciences Library, The University of Toledo, Toledo, OH

Objectives: Intentional reflective practice (IRP) is common among nurses and other professions with high levels of interpersonal interactions. There is little information about the use of IRP among librarians, and no studies specifically on medical librarians found in the published literature. This study examined the role of IRP among health science librarians, perceived benefits, and perceived barriers to use.

Methods: This cross-sectional study replicated the 2014 study by Greenall and Sen, who surveyed librarians and information professionals in Britain to determine if and how they used reflective practice/writing in their professional work. This research used a modified version of their anonymous questionnaire (tailoring it to health sciences librarians), consisting of primarily close-ended questions, with several open-ended questions. The research population in this study is health science librarians, primarily working in the US, who are members of MEDLIB-L, MLA Chapter, and/or MLA Section email lists. An email was sent to these lists, inviting health science librarians to complete the online questionnaire.

The results and conclusion will be made available at the poster presentation.

Keywords: reflective practice, reflective writing, reflection, professional development

Poster Number: 117

Time: Sunday, May 28, 2017, 2:00 PM – 2:55 PM

Promoting Research across the Library through an Inclusive Interest Group

John Cyrus, Research & Education Librarian, Tompkins-McCaw Library, Tompkins-McCaw Library / Research and Education, Richmond, VA; **Patricia Sobczak**, Collections Librarian and Assistant Professor, Virginia Commonwealth University, Cabell Library, Richmond, VA

Objectives: To create an intralibrary interest group to promote research to library faculty and staff by providing training and resources as well as leveraging organizational knowledge.

Methods: In spring 2016, the authors discussed forming an informal interest group to explore all aspects of the research process from research questions to dissemination. The group would capitalize on expertise in the library and draw from university experts to increase interest in research across the library, provide exposure to skills and knowledge, and assist with projects in development. The authors also seek to explore and encourage research collaborations between library departments. Open to library faculty and staff, the group launched in fall 2016 and meets semiannually. First year objectives include: creating library-wide inventory of ongoing research projects, generating at least one research project to implement in 2017, and providing at least one training session on on core research topic. The first year will focus special attention on progress made towards objectives, feedback, lessons learned, and future goals.

Results: The research interest group held three meetings in its first nine months. After the first meeting, a research project registry was created using Google Forms, allowing individuals to submit ongoing projects or ideas in development. Entries in the registry also indicate whether the project is open for collaboration. Despite creation of a registry, the group has yet to generate a research project on its own. There was significant interest in learning more about obtaining funding for research and other scholarly projects within the library, which prompted the group to offer two training sessions, a webinar on COS Pivot and a Reference and User Services Association (ALA) webinar on grant-funding and grant support services. Feedback from the meetings has been largely positive, and it has given the group helpful input that will guide its further development.

Conclusion: In the first nine months of existence, the research interest group has held meetings that regularly attract librarians from a variety of departments within the library. This demonstrated interest in topics related to all facets of research has created what seems to be a heightened awareness to research related topics especially in terms of learning opportunities. As the group continues to find its footing, we are looking at ways to increase our reach and voice within the library. This includes promoting practical research related to day-to-day functions as well as highlighting university level opportunities for training and funding.

Keywords: research, collaboration, training, education

Poster Number: 118

Time: Monday, May 29, 2017, 2:30 PM – 3:25 PM

Inpatient Attitudes Toward Mobile Device Use Among Health Care Professionals: A Collaborative Study

Lori Giles-Smith, Hospital Librarian, University of Manitoba, Bill Larson Library, Grace, Hospital, La Salle, MB, Canada; **Andrea Spencer**, Manager of Patient Care, Health Sciences Centre, Vascular Access Team, Central Lines and Nutrition Support Services, Winnipeg, MB, Canada

Objectives: As mobile devices become increasingly popular in healthcare and hospital librarians promote application features, understanding how patients feel about mobile device use is crucial to developing relevant hospital policies and educational initiatives. This study explores patient attitudes towards mobile device use among healthcare professionals and seeks to determine whether patients understand why mobiles devices might be utilized at the bedside.

Methods: This study employed an interview design guided by an investigator-developed descriptive survey. A hospital librarian and nurse interviewed a convenience sample of adult inpatients in medical and surgical units at a community hospital to explore current patient attitudes towards healthcare professional's usage of mobile devices and mobile applications in support of direct patient care. A focus group discussion with the hospital's Patient and Family Advisory Council garnered additional insights into concerns, expectations, and feelings related to device utilization. Recordings of the patient interviews and focus group were transcribed and coded by the researchers so that trends could be identified. Descriptive statistics were analyzed to describe the characteristics of the patients interviewed and determine the representativeness of the study's sample.

Results: Study analysis is ongoing. The results will be available during the poster presentation at MLA '17.

Conclusion: Study analysis is ongoing. The conclusions will be available during the poster presentation at MLA '17.

Keywords: survey design

mobile applications

mobile communication devices

interprofessional collaboration

patient-centred care
hospital setting
point of care resources

Poster Number: 120

Time: Tuesday, May 30, 2017, 2:00 PM – 2:55 PM

Daring to Influence Higher Education: Two Health Sciences Librarians' Experiences with Non-Librarian Leadership Programs

Annie M. Thompson, Director, University of Southern California, Wilson Dental Library, Los Angeles, CA; **Elizabeth C. Whipple, AHIP**, Research Librarian, Ruth Lilly Medical Library, IU School of Medicine, Indianapolis, IN

Objectives: This poster will provide an overview of the authors' experiences attending non-librarian related leadership programs, specifically two women in leadership programs offered through the HERS institute.

Methods: Health sciences librarians have the opportunity to take advantage of a variety of library leadership-related intensive programs, including the NLM/AAHSL Fellowship, Harvard Leadership Institute for Academic Librarians (LIAL) and MLA-offered Continuing Education courses. While it is logical for those in supervisory and leadership positions to take advantage of courses aimed at librarians, it is also advantageous to attend leadership programs that provide a wide-ranging view of issues in higher education, as well as navigating the broader world in which many of us operate. The authors will provide an overview of their experiences participating in two unique programs offered through the HERS Institute as well as discuss strengths, weaknesses, and lessons learned for health sciences librarians.

Results/Conclusions:

Attending a non-librarian leadership program helps to provide a broader understanding of issues and challenges in higher education. We have also broadened our professional circles within higher education, and were able to bring our librarian perspective and experience to higher education circles. This leadership program could be improved through incorporating more active learning sessions and more time for discussing and talking with colleagues. Our interactions with attendees was as valuable, if not more, than the "official" program.

Keywords: leadership, women, higher education, continuing education, leadership programs, women in leadership

Poster Number: 121

Time: Sunday, May 28, 2017, 2:00 PM – 2:55 PM

Reengaging Faculty and Students with the Library: Creating Outreach Programs and Departmental Collaborations That Work

Nicole S. Dettmar, AHIP, Curriculum Design Librarian, Health Sciences Library, Seattle, WA; **Tania Bardyn, AHIP**, Associate Dean and Director, Health Sciences Library, University of Washington–Seattle

Objectives: With the rise of online resources and remote services in academic health sciences libraries, librarians find it harder to engage faculty in library services to support teaching and learning.

Positive collaboration with faculty is essential to effective library instruction programs and to fulfilling our teaching mission. Non-traditional services can re-engage faculty and students with the library.

Methods: Through faculty surveys, participation on faculty and departmental committees, embedded librarian activities, and involving faculty in library space planning activities, we identified and implemented multiple new services and programs to meet the information needs of faculty and re-engage them with the library. The new programs include partnering with academic departments on teaching and learning teams to assist faculty with designing online courses and integrating resources within them, assessing research impact by partnering with departments in faculty promotional activities, creating a testing/assessment center in the library computer lab, providing space in the library for professional writing support, and building out new library spaces with technology to promote active learning and collaboration. The library can increase faculty engagement and therefore improve services to students as faculty become more aware of new library services and resources and see librarians as full partners in teaching and learning.

Results: Preliminary results of the new programs, including increased library resources and space usage in support of teaching and learning, will be made available during the poster presentation.

Keywords: Faculty, students, outreach, collaboration, engagement

Poster Number: 122

Time: Tuesday, May 30, 2017, 1:00 PM – 1:55 PM

The Science and Magic of Marketing Harry Potter's World at a Medical Library

Kelsey Leonard, AHIP, Health Information Services Librarian / Assistant Professor, Preston Medical Library / Health Information Center, Preston Medical Library / Health Information Center, Knoxville, TN; **Donna Doyle**, Communications & Programs Coordinator, University of Tennessee Graduate School of Medicine / University of Tennessee Medical Center, Health Information Center - Preston Medical Library, Knoxville, TN; **Martha Earl, AHIP**, Assistant Director / Associate Professor, Preston Medical Library, Preston Medical Library / Health Information Center, Knoxville, TN

Objectives: To demonstrate how the use of a National Library of Medicine (NLM) Traveling Exhibit can increase awareness and usage of the medical library.

Methods: A medical library hosted the NLM's Traveling Exhibit, "Harry Potter's World: Renaissance Science, Magic, and Medicine." A librarian and a communications/programs coordinator developed marketing plans for the exhibit prior to its arrival and a schedule of events during the display period. Two speakers were scheduled for the opening of the exhibit. Hospital marketing created and distributed a press release. Flyers were sent to surrounding colleges and public libraries. A local news station interviewed librarians, dressed as wizards. The related library display included information on the exhibit speakers, postcards created using images from NLM's website, a guest book, and the Harry Potter books. Participants could enter a drawing to win the books by completing an exhibit quiz. Opening day, the local news station did several three minute, early morning segments on the exhibit.

Results: Members of the community learned about the event and came to the medical center specifically to view the exhibit. 11 people called wanting more information after seeing the event on the local news. 201 people signed the guest book, and 114 people took the quiz to be entered to win the Harry Potter book series. The door count increased by 171 people during the exhibit. Over 600 postcards were printed throughout the event and all were taken. 33 people attended the first speaker's presentation, and 14 attended the second speaker's presentation.

Conclusion: During the National Library of Medicine's traveling exhibit, library usage increased. Members from the community attended programs at the medical library while also learning about the connection between Harry Potter and Renaissance science, magic, and medicine. The event was a success, the library is planning to host another traveling exhibit in fall 2017.

Keywords: National Library of Medicine, Marketing, Traveling Exhibits, Harry Potter, Community Outreach, Literature, Pop Culture

Poster Number: 123

Time: Tuesday, May 30, 2017, 2:00 PM – 2:55 PM

Voices in the Choir: Librarians Helping to Move Osteopathic Research Forward

Lori Fitterling, Instructor of Medical Informatics/Reference Librarian, D'Angelo Library, D'Angelo Library, Kansas City, MO; **Jean L. Sidwell**, Library Director - Missouri Branch, A.T. Still Memorial Library , A.T. Still Memorial Library, Kirksville, MO

Objectives: Medical research continues to frame clinical practice. With the unified accreditation system, evidence-based research that demonstrates a relevance to osteopathic medicine is finding a place in medical literature. Previous research gathered information about osteopathic librarians' research experiences, highlighting a gap in osteopathic research in the medical literature. The current study poses the question, "What can osteopathic librarians do to increase osteopathic research?" By critically looking at osteopathic library collections and searching challenges, this project offers ways to encourage osteopathic research.

Methods: Three methods were used to gather information. Using a three-item survey designed for this study, osteopathic libraries were surveyed about how much of their total collection is considered osteopathic, how much of that collection is used, and how much of their total materials budget supports osteopathic medicine resources. Second, PubMed and the Clinical Trials databases were searched for osteopathic research topics for the past 20 years, systematic reviews of osteopathic topics, and the number of clinical trials involving osteopathic manipulative medicine or treatment. Third, osteopathic library websites were reviewed for osteopathic search guides and helpful finding aides.

Results: Most osteopathic libraries indicated that less than ¼ of their total library collection was considered osteopathic, 30% indicated that their osteopathic collection was used on a daily basis and 45% reported that about ¼ of their total materials budget supports osteopathic medicine resources. 65% reported that less than half of their osteopathic collection is in electronic form and 85% reported they are not digitizing osteopathic materials. 45% of osteopathic libraries that responded have conducted an osteopathic literature review and 55% of libraries have consulted international osteopathic resources.

Conclusion: Results suggest that while osteopathic libraries have osteopathic resources, very little of their total materials budget supports their osteopathic collection and very few are adding to their collection by digitizing print materials. Most osteopathic library websites have osteopathic resources search guides and finding aides, and osteopathic librarians contribute to osteopathic research at their institutions by performing osteopathic literature and systematic reviews. As such, these results may be used to help librarians add their voice to the osteopathic research community.

Keywords: Osteopathic medicine, osteopathic research, literature reviews, osteopathic collections

Poster Number: 124

Time: Tuesday, May 30, 2017, 2:00 PM – 2:55 PM

Daring to Reexamine and Reimagine a Reference Service: Is It Needed?

Jonquil D. Feldman, AHIP, Associate Library Director, Briscoe Library, UT Health San Antonio, San Antonio, TX; **Luis F. Barcenes, III**, Supervisor, of Circulation Services, University of Texas Health Science Center at San Antonio, Briscoe Library, San Antonio, TX; **Karen D. Barton**, Liaison to the School of Health Professions, Briscoe Library, UT Health San Antonio, San Antonio, TX; **Christine S. Gaspard**, Liaison to the School of Medicine, Briscoe Library, Briscoe Library, San Antonio, TX; **Emme**

Lopez, Liaison to the School of Nursing, University of Texas Health Science Center at San Antonio, Briscoe Library, San Antonio, TX

Objectives: Evidence shows that the number of reference transactions in academic health sciences libraries is trending downward. As a result, many libraries have changed how they provide reference services. The purpose of this paper is to discuss how UT Health San Antonio librarians identified problems, weaknesses and strengths, and transformed our reference services from a scheduled "on call" to an unscheduled "as needed" service.

Methods: After the Reference Desk was removed eight years ago, Circulation staff served as the frontline, referring in-depth questions to "on-call" librarians, who were scheduled 4-6 hours per week. The number of questions averaged 0-2 during a 2-hour shift and the majority of questions were about room reservations. Many librarians did not enjoy being on call and it became difficult to fill open shifts. With a new director came the creation of a liaison librarian program to work with each of our five schools. The liaison librarians were tasked with re-designing reference services with Circulation staff as the first point of contact. They reviewed the questions typically received and created a question flow chart. A training plan was developed for Circulation staff to manage the question flow. Online forms and guides were created for specific tasks or resources. The last daring move was to eliminate the on call schedule.

Results: Questions referred by Circulation staff were responded to by an available librarian. Liaisons responded to questions from faculty or students from their assigned school. We will discuss how this unscheduled model has worked out for both librarians and Circulation staff.

Conclusion: Re-imagining the reference service required hours of introspection to determine what was wrong with it, dream up an alternative, and dare to implement a change. With appropriate training for Circulation staff, and with liaisons also deployed on campus to provide personalized research support and instruction, the need for traditional scheduled reference services has been eliminated.

Keywords: Reference re-design, Reference Question flow chart, Liaisons, Circulation staff training, Daring to Change

Poster Number: 125

Time: Sunday, May 28, 2017, 2:00 PM – 2:55 PM

Library Resources Role in Specialty Rehabilitation Services at Model System Centers

Christine A. Willis, Director of Knowledge Management & Learning Resources, Noble Learning Resource Center, Shepherd Center, Atlanta, GA

Objectives: To determine what library resources and services are available to the Spinal Cord Injury Model System Centers (SCIMSC) & Traumatic Brain Injury Model System Centers (TBIMSC) and how those resources are utilized to further research dissemination.

Methods: An electronic survey was sent to each of the 14 SCIMSC and 16 TBIMSC using the email on the Model Systems Knowledge Translation Center (MSKTC) website. The survey sought to gather general information on the available library services, including professional librarians, electronic database access and most used journal subscriptions. The survey asked about academic affiliation, number of years as a model system, number of research staff, funding sources for library resources and library staffing.

Results: Of the 22 unique Model System Centers in survey group, ten Centers responded and 2 email addresses on the MSKTC were returned as undeliverable. Attempts were made to find correct emails but did not result in a working email to use. Half of the respondents are both a SCIMSC & TBIMSC, and the majority are associated with both an academic institution and hospital. Research staffs are generally small with most having 10 or fewer research staff supported by the Model System. All Centers have

access to library resources and most reported having access to a professional librarian. Eight Centers responded that funding for library resources and staff is through the institution/hospital or they were not sure. No library services were supported with Model System grant funding. The most common subscription databases that Centers confirmed to have access are: EBSCO, ClinicalKey/Elsevier, and Wolters Kluwer. Of the 19 general rehabilitation/discipline specific journals, the most common ones accessed included: American Journal of Physical Medicine & Rehabilitation, Archives of Physical Medicine & Rehabilitation, Assistive Technology, Disability & Rehabilitation, Physical Therapy, PM&R, and Rehabilitation Psychology. Nine brain injury related journals were listed for Centers to select and all but 1 were utilized by the majority of TBIMSCs respondents. Similarly, of the 4 spinal cord injury related journals 3 were utilized by the SCIMSCs who responded.

Conclusion: Model System Centers are well supported with library resources by their institutions and it may be worth the librarians associated with these institutions looking into a Docline group for these specialty resources. Collaborations to update rehabilitation journal lists and bibliometric analysis projects using papers produced by these Centers can be considered to insure the appropriate resources are accessible by Model System researchers.

Keywords: rehabilitation, spinal cord injury, traumatic brain injury, library services, model system

Poster Number: 126

Time: Monday, May 29, 2017, 2:30 PM – 3:25 PM

Library Outreach to Administrative Professionals in a Biomedical Research Institution

Benjamin D. Hoover, Access Services and Instruction Librarian, Harrell Health Sciences Library: Research and Learning Commons, Penn State University College of Medicine/Hershey Medical Center, Hershey, PA; **Marie C. Cirelli**, Collection Access and Support Services Librarian, Penn State University College of Medicine/Hershey Medical Center, Harrell Health Sciences Library: Research and Learning Commons, Hershey, PA; **Robyn B. Reed, AHIP**, Biomedical Informatics and Emerging Technologies Librarian, Harrell Health Sciences Library, Harrell Health Sciences Library: Research and Learning Commons, Hershey, PA; **Lori B. Snyder**, Collection Development and Digital Resources Management Librarian, Penn State University College of Medicine/Hershey Medical Center, Harrell Health Sciences Library: Research and Learning Commons, Hershey, PA

Objectives: Administrative professionals employed by biomedical researchers are charged with a variety of library-related research responsibilities. To increase their knowledge and proficiency in these tasks, the library developed a series of workshops and a resource guide aimed specifically at assisting this population.

Methods: Using data from reference tracking software as well as known challenges, workshops and an accompanying guide were designed covering topics including using library resources, citation management software, MyNCBI, calculating h-indexes, and determining article citation counts. The institution employs approximately 90 administrative professionals across clinical and academic research departments. This target audience received emails advertising the sessions. The workshops have been offered annually in recent years due to attendance, participation, and survey feedback. The goals of the initiative are to increase knowledge regarding library resources as well as providing a point of contact within the library for future questions.

Results: The workshops were offered in the Spring of 2015 and the Spring of 2016. In 2015 aggregated attendance over 3 workshops (Endnote, Library Overview, and Author Support) was 52 people. Attendance dropped to 16 people over the same number of workshops (Endnote, Library Overview, My NCBI) in 2016. Usage of the LibGuide created in preparation for the workshops generated more than 3,000 views in a two year period. In 2015 there were 710 page views and in 2016 there were 2,228 page views. Results of the post-workshops surveys indicated that the workshops were generally well-received.

Conclusion: The decrease in workshop attendance and increased LibGuide use may be attributed to a small pool of candidates and repeated workshop topics, or a preference for a self-service online resource. In 2017 attendance and LibGuide use will be tracked and compared to prior years. Some challenges include teaching a group of individuals with varied technology levels, maintaining interest in the workshops by offering relevant topics in a timely manner, and communicating the value . Moving forward in 2017 five workshops will be offered including new and updated content. In addition, the LibGuides will be updated to reflect content changes.

Keywords: Library Outreach, Workshop, Library Instruction, Administrative Support Staff, Research Assistance, Administrative Professional, Biomedical Research

Poster Number: 127

Time: Tuesday, May 30, 2017, 1:00 PM – 1:55 PM

How to Merge Libraries: A 3-D Guide (Dream, Dare, and Do)

Michael S. Fitts, ASST. DEAN FOR DIVERSITY; **Sylvia McAphee**, CHAIR OF ACQUISITIONS; UAB LIBRARIES, BIRMINGHAM, AL

Objectives: Merging the two major libraries at the University of Alabama at Birmingham (The single largest employer in the state of Alabama) has enabled us to better serve our users. While several previous attempts had been made to encourage more collaboration between the Mervyn H. Sterne and Lister Hill Library of the Health Sciences (an academic and a medical library) only moderate success was seen. Users however now are singing the praises of UAB libraries and librarians.

Methods: Combining resources allows the library staff to provide more specialized services to faculty and students. In addition to creating an organization with more effective management of the libraries' resources and increased efficiencies allows the libraries to shift more resources toward its collections and new user services. Together, the libraries have more than 2 million volumes with over 500,000 titles.

Results: The creation of the new library structure, while still transitioning, has already increased the number of as well as the quality of services the libraries provide to the university and the community at large. With an individual at the helm of the libraries such as Dean John Meador who brought with him a wealth of knowledge in library science, exemplary administrative leadership and a strong commitment to community outreach, makes it beneficial for everyone involved. Throughout this process, some "best practices" have emerged.

Conclusion: Admittedly change can often times be difficult for people; and while some still may be against the idea of merging. The realization is that there are likely to be more mergers in the not too distant future. We must realize that in today's society of interdisciplinary research and collaboration the fact remains that combining efforts just makes sense. It's about pulling together and providing resources to our users as seamlessly as possible. Libraries should continuously review processes and services as our users' needs change. We must adjust to meet those needs.

Keywords: merger diversity academic health sciences collaboration seamless users best practices

Poster Number: 128

Time: Tuesday, May 30, 2017, 2:00 PM – 2:55 PM

HappyOrNot: Investigating an Innovative Way to Evaluate Services and Assess User Satisfaction

Nadine Dexter, AHIP, Director Harriet F. Ginsburg Health Sciences Library, Harriet F. Ginsburg Health Sciences Library, Harriet F. Ginsburg Health Sciences Library, Orlando, FL; **Shalu Gillum, AHIP**, Head of Public Services, Harriet F. Ginsburg Health Sciences Library, Harriet F. Ginsburg Health Sciences Library, Orlando, FL; **Deedra Walton, AHIP**, Head of Electronic Resources, Harriet F. Ginsburg Health Sciences Library, University of Central Florida College of Medicine, Orlando, FL; **Natasha Williams**, User Services Librarian, University of Central Florida College of Medicine, Harriet F. Ginsburg Health Sciences Library, Orlando, FL; **Pamela Herring, AHIP**, Electronic Resources Librarian, Harriet F. Ginsburg Health Sciences Library, Harriet F. Ginsburg Health Sciences Library, Orlando, FL

Objectives: To investigate whether the "HappyOrNot" terminal is an effective method to collect meaningful user feedback about library resources, services, and events. In the past the health sciences library has gathered student and faculty feedback via email surveys and focus groups, however response rates have been low. In an effort to obtain just-in-time user experience information, the library decided to purchase a "HappyOrNot" system.

Methods: The library purchased a HappyOrNot standalone “smiley terminal,” commonly seen in airports. The terminal displays a question, which can be changed as often as needed, and prompts users to respond to the question by pressing a button corresponding to their level of satisfaction. The buttons depict smiley faces ranging from happy to mad. The smiley terminal will be displayed in various locations within the library in conjunction with library events and will display different questions in order to gather user experience information about library resources, services, and events. Data is gathered and sent to the Cloud, and reports can be generated by the hour, day, and more, allowing for more specific user feedback data. Data collected from the smiley terminal will be compared to responses collected from emailed surveys asking the same question. We would like to compare the response rates of the two types of assessments, as well as the actual responses.

Results: The library is continuing to collect data from the smiley terminal. To date we have had 17 unique surveys, including: “How was your stop at the library today?”, “How helpful was today’s library orientation session,” and “How satisfied were you with the topic of today’s lunch & learn session?” During one particular Monday-Sunday period, we received 141 responses to “How was your stop at the library today?” with 79.4% of users answering “very positive” and 17% answering “positive,” for a total of 96.4% of users having positive experiences with the library.

Conclusion: The HappyOrNot smiley terminal provides the library with important data about library users’ level of satisfaction at a given moment in time. While the survey responses are not as robust and detailed as those collected through email surveys, the terminal allows us to easily and quickly perform micro-assessments about a variety of topics. We are still evaluating whether this system is a good way to collect meaningful user data.

Keywords: assessment, survey, evaluation, feedback, Micro Assessment Tool

Poster Number: 130

Time: Monday, May 29, 2017, 2:30 PM – 3:25 PM

Derring Do Survey Data: Exploring Health Sciences Library Collaboration with Evaluation Experts

Marian G. Taliaferro, AHIP, Director, AAMC Reference Center & Archives, Washington, DC

Objectives: To investigate the value in consulting and collaborating with evaluation experts for the purpose of gaining richer data on health sciences special library services. Does working with a third party offer added benefits for richer data on new opportunities for the library?

Methods: A small health sciences special library setting, offering research and both library and archives resources to approximately 650 staff and to the public, partnered with an expert evaluation team to implement a follow-up study. Library staff invited participation from the evaluation team to act as a third

party such that data collected would be highly constructive and to further ensure anonymity. Through this effort, earlier online survey data on user awareness of the health sciences library's services and research support was supplemented with data obtained from an intervention via 3 staff focus groups. These staff responded to a series of in-person questions to provoke conversation for the purpose of offering input on new opportunities for the library as well as on user's preferences for receiving information from the library.

Keywords: evaluation, collaboration, assessment, research services, special libraries, health association libraries, new services, new roles

Poster Number: 131

Time: Tuesday, May 30, 2017, 1:00 PM – 1:55 PM

Capturing Data to Explore the Use of a Library's Public Spaces

Shenita Peterson, Life Sciences Informationist; **Amy Allison, AHIP**, Associate Director; Woodruff Health Sciences Center Library, Woodruff Health Sciences Center Library, Atlanta, GA

Objective: Recently, a university's library system began the process of planning future facility renovations. The health sciences library decided to conduct a seat survey to assess utilization of the library's public study spaces. The goal was to provide decision-makers with data about the types of spaces and seating preferred by users in order to be considered in the renovation planning process.

Methods: A map was created to identify and label each seat in the public library spaces. Library staff collected data five times each day during two separate 7-day periods in a single semester. Each data collector marked on the map each occupied seat. Data for each collection period was entered into an online form using Google Docs. Data was organized and aggregated for analysis; individual seats into groups by type of seating (carrels, tables, computer workstations, and soft seating). Results were reported to the health sciences library's administration.

Results: The maps represented 290 seats. Seating groups included 20 groups of adjacent carrels (105 seats), 30 tables (96 seats), and 15 groups of soft seating (40 seats). There were also 49 seats at computer workstations. Staff recorded seat occupancy data during 78 observations over the two weeks. Occupancy rates were calculated for each observation period. Occupancy rates for all seats ranged from 9.7% to 44.8%. Carrels had the highest occupancy rate in a single period at 68.6%, followed by seats at computers (57.1%), seats at tables (41.7%), and soft seating (15%). Comfortable occupancy rates for seating groups were also calculated and were defined for tables (half of the seats at the table were occupied), for carrels (half of adjacent carrels occupied), and for soft seating (at least one seat occupied). Carrel groups had the highest comfortable occupancy rates (ranged 0 to 90%) with over half of the carrel groups reaching comfortable occupancy during ten of the fourteen days measured.

Conclusion: During this survey period, carrels were the preferred type of seating, and soft seating the least preferred. The seat survey is useful for assessing user preferences and patterns for occupying public seating spaces. Next steps should include investigating why users utilize the carrels and tables more frequently than other types of seating, as well as why some groups of seating are utilized more frequently than others.

Keywords: assessment, facility, planning, data

Poster Number: 132

Time: Tuesday, May 30, 2017, 2:00 PM – 2:55 PM

Doing More with Library Space: Assessment and Planning through a Space Utilization Study

Katie A. Prentice, AHIP, Associate Director for User Experience and Assessment, Schusterman Library, University of Oklahoma-Tulsa, Tulsa, OK; **Erica K. Argyropoulos**, Graduate Research Assistant, University of Oklahoma-Tulsa, Schusterman Library, Tulsa, OK

Objectives: The objective of this study was to assess the space and furniture utilization at the Schusterman Library, a small academic library, through direct observation.

Methods: The initial direct observation project took place over two long semesters in a small academic health sciences library. With a detailed floor plan and legend of activities to identify, the first floor was observed in the Fall of 2015 and the second floor in Spring of 2016. Library graduate assistants conducted the observations and marked a floor plan map every two hours from opening to track the ebb and flow of space use. Movement of furniture was also noted as well as the study activity and use of laptops. The two semesters of data was used to request funding for electrical improvements for open spaces. In the Spring of 2017 data will be collected about table and power use after installation of upgrades to determine use-changes of the newly powered spaces.

Results: Extensive use details were collected about where library users sit during what part of the day and certain areas of the library were identified as much more active than others. Entire furniture groups were identified as wholly under-used. Inadvertent information was collected about snacks, food, and personal items left unattended. The strong need for additional electrical plugs was identified by table movement toward walls.

Conclusion: Full and active use of library space is important for planning. The data has already been used to justify funding for electrical improvements to open spaces; additional solutions are being considered for other under-utilized spaces and detailed space use information is available for consultation. As the physical footprint of the Schusterman Library is small, knowing how space is used is important for any expansion plans and the information will be valuable to all future physical improvements.

Poster Number: 133

Time: Sunday, May 28, 2017, 2:00 PM – 2:55 PM

Creating Community: How Our Library Dreamed Big, Dared to Take Chances, and Did Something New

Melanie J. Norton, Head of Access and Delivery Services, Cushing/Whitney Medical Library, Cushing/Whitney Medical Library, New Haven, CT; **Melissa Funaro**, Clinical Librarian, Cushing/Whitney Medical Library, Yale University, New Haven, CT

Objectives: Those in the medical profession, suffer from stress, anxiety, and burnout as demands of work and/or study can create negative impacts on their physical and mental health. Close to 10% of fourth year medical students report having suicidal thoughts, while 400 doctors commit suicide every year. Our library is offering space and programs to encourage community and promote resiliency.

Methods: Recognizing the benefits of community to promote resiliency and mental wellbeing, our library "hired" a therapy dog in 2014 to give our students and healthcare professionals an opportunity to relax, bring them general happiness and encourage conversation with colleagues.

We incorporated board games into the collection for check-out; providing our patrons a chance to enjoy the games and take their minds off of their studies, while interacting with their peers.

We partnered with students from the Wellness Center to create a non-denominational wellness space within the library where individuals can meditate, quietly reflect, or practice yoga.

The library has traditionally been a place of learning and study, but we believe that the library can also create a sense of community while supporting the wellbeing of our healthcare population.

Keywords: Community, Resiliency, Wellness, Library Space, Mental Health, Anxiety, Space

Poster Number: 135

Time: Sunday, May 28, 2017, 2:00 PM – 2:55 PM

Exemplary Practices to Inspire Library Accessibility: A Toolkit

Patricia F. Anderson, Emerging Technologies Informationist, Taubman Health Sciences Library, Taubman Health Sciences Library, Ann Arbor, MI; **Lynne Cutler**, Branch Manager, City of Oakland, Montclair Library, Oakland, CA; **L. Scott Lissner**, ADA Coordinator, Ohio State University, University ADA Coordinator, Columbus, OH; **JJ Pionke**, Assistant Professor and Applied Health Sciences Librarian, Social Science, Health, and Education Library at the University of Illinois Urbana Champaign, Social Sciences, Health, and Education Library at UIUC, Champaign, IL; **Stephanie Rosen**, Accessibility Specialist, University of Michigan-Ann Arbor, Hatcher Graduate Library, Ann Arbor, MI; **Anna Ercoli Schnitzer**, Informationist, University of Michigan, Taubman Health Sciences Library, Ann Arbor, MI; **Jane Vincent**, Assistive Technology Manager, University of Michigan-Ann Arbor, Shapiro Library / Knox Ctr, Ann Arbor, MI

Objectives: Libraries have a proud history of increasing access for the public to collections and resources not readily available. Online environments and increased awareness of special needs populations raise the need for libraries to take leadership roles in accessibility of environments and services. In this poster we collect exemplary practices in creating accessible library environments as a toolkit for other libraries.

Methods: There are many possible roles for librarians in supporting disabilities and special needs. Our team utilizes surveys, focus groups, and searches to identify needs in our own institution as well as leaders and best practices from other institutions. These efforts have resulted in new collaborations, task force, official reports, working documents, changes in services and resources, new practices in negotiating with vendors, outreach to new special populations, and more. This is an iterative process, allowing us to build on past initiatives while planning future directions. Outcomes and products to date include tools to facilitate communication with special needs patrons, training programs for staff, guidelines for inclusive meeting planning, service animal events, adaptive technology services, use of new technologies to extend access to new populations, revised contract negotiation practices, and more.

Results: Will be provided at the MLA Annual Meeting.

Conclusion: Will be provided at the MLA Annual Meeting.

Keywords: disability, accessibility, library services, special needs, facilities, library design, innovation

Poster Number: 136

Time: Tuesday, May 30, 2017, 2:00 PM – 2:55 PM

Creating the Dream: Reimaging a Special Collections Space

Esther E. Carrigan, AHIP, Associate Dean and Director, Medical Sciences Library, Texas A&M University, College Station, TX; **Nancy G. Burford**, Veterinary Collections Curator, Medical Sciences Library, Medical Sciences Library, College Station, TX

Objectives: To plan the renovation of a closed, limited-use special collections suite into an area that would allow a variety of activities: a space for increased student day use, a place to reserve for special events, a visible space with optimal physical environment for collections, reading room, and an exhibit space to showcase and encourage use of historical collections.

Methods: Brainstorming sessions were held to generate ideas about source s of ideas for the space. Feedback from LibQUAL+ service satisfaction dealing with library physical spaces and experience

gained from previous library user space renovation projects was incorporated. Visits to other libraries and interviews with colleagues were employed. An outline of the goals for the space transformation were developed and shared with a library interior design firm. Concept drawings and a project budget estimate were developed, and a specialized renovation project team created. Detailed planning meetings were held during winter-spring 2016 and construction began late spring 2016.

Results: Renovations were completed and use of the space underway in spring 2017. Usage of the space is monitored through counter devices and logs of events/tours. Feedback is gathered from students using the space for study and visitors to exhibits. Data on use of the space will be provided.

Conclusion: The renovated spaces provide a versatile and useful space and a foundation for further activities. Planning is underway to promote use of the space and the historical collections. Discussions are planned with library departmental liaisons and university faculty to brainstorm projects and assignments that will use the historical collections both for their subject contact and as examples of printing history.

Keywords: historical materials, special collections, archives, transforming library spaces

Poster Number: 137

Time: Sunday, May 28, 2017, 2:00 PM – 2:55 PM

Physical Space Utilization in a Health Sciences Library: An Ithaka Case Study

Jean P. Shipman, AHIP, FMLA, Executive Director, Knowledge Management & Eccles Health Sciences Library, Spencer S. Eccles Health Sciences Library, Spencer S. Eccles Health Sciences Library University of Utah, Salt Lake City, UT; **Darell Schmick, AHIP**, Research Librarian, University of Utah, Spencer S. Eccles Health Sciences Library, Salt Lake City, UT; **Mellanye Lackey**, Associate Director for Education and Research, University of Utah, Eccles Health Sciences Library, Salt Lake City, UT; **Nancy Foster**, Senior Anthropologist, Ithaka S+R, Libraries and Scholarly Communication, New York, NY

Objectives: To better understand how the physical space of the Spencer S. Eccles Health Sciences Library (EHSL) is being used, why non-users don't come to the physical library, and how to improve communications with users.

Methods: The EHSL hired a consultant from Ithaka S+R to develop a space study. Three instruments were applied to gather data - question reply cards for distribution inside the library's physical space, direct observations of designated spaces inside the building, and consultant-conducted structured interviews regarding use of the space and communications about the library. A project team of library faculty and staff performed the data collection. The consultant supported analysis and interpretation of the data.

Results: Data was collected over the course of a week at the end of March 2017. The consultant presented findings to library personnel to better understand user physical space utilization and communication preferences. These results will be highlighted on the poster.

Conclusion: The inclusion of Ithaka S+R provided a novel opportunity to examine health sciences library user behavior. The poster highlights comparisons between the EHSL findings and those of other academic libraries. Insights were shared with campus administration as a large health sciences campus transformation project is underway at the University of Utah. Project findings will assist with determining the long-term future of the EHSL building in light of this transformation. Further study will be conducted via campus surveys and focus groups to validate the project findings.

Keywords: Ithaka S+R, Space Utilization, Physical Library, Usage, Library Usage

Poster Number: 138

Time: Monday, May 29, 2017, 2:30 PM – 3:25 PM

Making a Case That Physical Library Space Relates Positively to Student Learning

Lori Fitterling, Instructor of Medical Informatics/Reference Librarian; **Marilyn J. DeGeus**, Director of the Library; D'Angelo Library, Kansas City, MO

Objectives This project addresses the question of how physical space in the library impacts medical students' *perception* of success. Furniture design, quiet and collaborative group study areas, adequate data ports and charging stations, and a general relaxing of library policies contributes to overall student success.

Methods: The D'Angelo library has made significant changes with the physical space of the library, and this project is an assessment of the impact these changes have had on first and second year medical student learning. Two methods were used to gather data. First, a learning space assessment was completed which tracked the usage of various spaces in the library at different times of the day for a six-month period. Second, a focus group of 50 first and second year students was conducted to gather information about their experience, perceptions and recommendations of the physical space in the library. This baseline data will be used for an annual assessment tool of the library's physical space and how it relates to student success.

Results: The learning space assessment determined that library usage is consistently high at the noon hour and that quiet areas are the most used spaces in the library, with group study rooms second. Focus group results had positive feedback for the new furniture design, the addition of Brody chairs and study booths that have provided flexibility, comfort, and added technology enhancements to individual study. Students prefer tables with data ports or carrels with rolling chairs and a relaxed atmosphere in the library. Students also prefer the natural light from the various windows, as it fosters academic success as well as decreases mental fatigue. Positive feedback for the art work and historical displays was also noted. Students suggested the purchase of small, portable white boards and floor-level flip-out chairs for relaxation purposes, and would like to be able to check out laptops. Overall results support positive perceptions of success from students when studying in the library.

Conclusions: This study shows that in the D'Angelo library the spaces provided are compatible with successful completion of student study goals. It is important to revisit space utilization to adequately meet the diversity of student learning, and this study will serve as a tool going forward to assess student perception of the D'Angelo library as it relates to their medical education.

Keywords: physical space student learning design assessment

Poster Number: 139

Time: Tuesday, May 30, 2017, 1:00 PM – 1:55 PM

Surprise and Delight: The Library as Cultural Space

Alexa Mayo, AHIP, Associate Director for Services, Health Sciences and Human Services Library, Health Sciences and Human Services Library, Baltimore, MD; **Thom Pinho**, Instructional Technology Specialist, University of Maryland, Health Sciences and Human Services Library, Baltimore, MD; **Erin D. Latta**, Coordinator, National DOCLINE Coordination Office, NNLM, SE/A RML - HS/HSL, UMD, Baltimore, MD; **Anna-Marie Epps**, Coordinator, University of Maryland, Health Sciences and Human Services Library, Baltimore, MD; **Persia Drummond**, Library Supervisor, University of Maryland, Health Sciences and Human Services Library, Baltimore, MD

Objectives: An academic health sciences library with a large gallery located prominently near its entrance, is committed to creating a multidimensional cultural space for the University. This poster

describes the successes in designing innovative and exciting wrap-around programming, displays, and activities that complement and add depth to the core gallery exhibits.

Methods: The library's Exhibits, Display and Promotion committee (EDP) is comprised of creative, artistically-minded staff from each division within the library. These volunteer members are responsible for designing wrap-around programming, displays, and activities that add value to the gallery's main attraction. The library hosts about five exhibits each year, including National Library of Medicine travelling exhibits, art from emerging and established artists, children's art from local schools, and more. The EDP team works to share focus with special collections and University resources - highlighting hidden gems within the campus community. The team uses resources in creative ways - transforming old-style glass display cases into curio cabinets, for instance - or by turning deaccessioned books into creative tables and sculptures. This poster will highlight effects the EDP committee's fresh and experimental approach has had on the success of exhibits in the gallery.

Results: Innovative wrap-around activities surprise and delight visitors, increase foot-traffic in the gallery, and position the Library as a cultural destination within the University.

Conclusion: Designing innovative and exciting wrap-around programming, displays, and activities adds depth to the Library's core gallery exhibits. A volunteer committee of creative staff encourages community-building and camaraderie within the Library.

Keywords: space; cultural space; art; art exhibits; collaboration

Poster Number: 140

Time: Tuesday, May 30, 2017, 2:00 PM – 2:55 PM

Incorporating Active Learning Library Sessions for Dental Students

Rebecca Davis, Information Services Librarian, Wilson Dental Library, Los Angeles, CA

Objectives: Library instruction sessions enable first year Doctor of Dental Surgery (DDS) students in Problem Based Learning (PBL) to familiarize themselves with library resources to resolve their learning needs. In the Herman Ostrow School of Dentistry of USC, first year students attend four library sessions. The goal of each session being to teach students about library resources through active learning activities.

Methods: Current literature does not address incorporating active learning in library instruction for dental students. The incoming DDS class of 2020 recently concluded four session of library instruction sessions which are part of the curriculum. Three sessions involve active learning activities which enables students to familiarize themselves with library resources to resolve learning needs for PBL. The three sessions are taught four times for 90 minutes with 36 students in each session. Sessions begins with a 15-20-minute lecture then students are divided into nine groups of four to complete an activity. During the resource selection session students search for library resources. The citing resources session enables students to practice citing various resources. For the PubMed Flipped Classroom students are sent PubMed tutorials and quizzes to before the session and then complete an activity conducting various searches in PubMed.

Results: Based on the evaluations, the majority of students agreed or strongly agreed that for the Resource Selection session they learned about library resources to use to resolve their learning needs, how to search for library information to resolve their learning needs and know where to go for help to resolve their learning needs. For the Citing Resources session, the majority of students agreed or strongly agreed that they learned about different citation styles and citation generators, how to cite using APA style, and where to go for assistance with citations. Lastly, for the PubMed Flipped Classroom the majority of students once again agreed or strongly agreed that they know how to do a basic search using Automatic Term Mapping in PubMed, learned about how to search PubMed using MeSH terms, and learned about the different between searching PubMed and Google Scholar.

Conclusion: By incorporating active learning activities in library sessions this gives students an opportunity to learn about how to search for library information because they are able to search on their own. This gives students hands on experience so that they are able to learn through trial and error. Based on the results from the evaluations, the majority of students agreed or strongly agreed that they had learned more about searching for library resources, how to cite resources and how to search PubMed.

Keywords: Active Learning, Dental Students, Library Sessions

Poster Number: 141

Time: Sunday, May 28, 2017, 2:00 PM – 2:55 PM

Landscaping the Future: How Librarians Contributed to a Medical School's Strategic Planning Process

Laura T. Kane, AHIP, Assistant Director for Information Services; **Felicia Yeh, AHIP**, Deputy Director; **Ruth Riley, AHIP**, Director, Library Services; School of Medicine Library, Columbia, SC

Objectives: To demonstrate how embedded librarians contributed to the success of a medical school's strategic planning process.

Methods: INTRODUCTION: In early 2016, the University of South Carolina School of Medicine formed a school-wide Strategic Planning Team charged with adopting and implementing a five-year strategic plan for the institution. The Team was comprised of a Steering Committee and four focused subcommittees in the areas of: (1) Education, (2) Research, (3) Clinical Care, and (4) Diversity and Inclusion. Faculty librarians were invited to serve as information resource consultants on each subcommittee.

METHODS:

The following data was collected:

• The number of committee meetings attended by the librarians

• The specific tasks completed by librarians within each subcommittee

• The number and types of literature searches completed

• Methods of information distribution by librarians to subcommittee members

Results: The committee work was completed in February of 2017 and the "USC School of Medicine Strategic Five-Year Plan" was shared with the School of Medicine's Executive Committee in March 2017.

Conclusion: The inclusion of librarians as consultants in the school-wide Strategic Planning Process proved to be a successful endeavor. Literature searches and fulltext documents provided by librarians were beneficial to committee discussions. The project provided an avenue for librarian participation in future administrative and planning processes within the academic medical center.

Poster Number: 142

Time: Monday, May 29, 2017, 2:30 PM – 3:25 PM

Shaping up Medical Students for Residency: Librarian Participation in Pediatric Bootcamp

Pamela Hargwood, AHIP, Clinical Librarian, Rutgers, The State University of New Jersey, Robert Wood Johnson Library of the Health Sciences, New Brunswick, NJ; **Elizabeth Goodman**, Assistant

Professor of Pediatrics/Pediatric Sub-Internship Director, Rutgers, The State University of New Jersey, Robert Wood Johnson Medical School, New Brunswick, NJ

Objectives: To describe the successful collaboration between a medical librarian and a Pediatric Sub-Internship Director in teaching evidence based medicine skills during a two week course to medical students in order to prepare the students for a pediatric residency.

Methods: The Association of American Medical Colleges (AAMC) has proposed "Entrustable Professional Activities" (EPAs) for medical students to ensure readiness for residency. The broad categories that these EPAs encompass include patient care, medical knowledge, practice based learning and improvement, interpersonal and communication skills, professionalism, and systems-based practice. In preparation for a pediatric residency, fourth year medical students take a two-week course, Pediatric Boot Camp. The medical librarian was asked to help teach the evidence based medicine session of this boot camp. The medical librarian taught four 45 minute EBM sessions on one day each week of the boot camp. During the 45 minute session, students were given a clinical scenario. As a group, the students formulated clinical questions based on the scenario. The students were each assigned a specific databases to answer the question and discussed their findings as a group.

Results: Will be made available during poster presentation and in the ePoster gallery.

Conclusion: Will be made available during poster presentation and in the ePoster gallery.

Keywords: entrustable professional activities, medical students, residency, evidence based medicine

Poster Number: 144

Time: Tuesday, May 30, 2017, 2:00 PM – 2:55 PM

Integrating Library Resources with Curriculums: A Case Report of Taipei Medical University Library

Shu-Yuan Siao, Head, Knowledge Services Sec, Main Library, Taipei Medical University Library, Taipei, Taipei, Taiwan (Republic of China); **Hsiao-Fen Yu**, Reference Librarian, Knowledge Services Sec., Main Library, Taipei Medical University Library, Taipei, Taipei, Taiwan (Republic of China); **Tzu-Heng Chiu**, Professor, Center of General Education/International Cooperation Division, National Central Library, Center for General Education, Taipei, Taipei, Taiwan (Republic of China)

Objectives: In order to integrate curriculums with library resources and to strengthen students' information retrieval skills, the Taipei Medical University Library (TMUL) cooperated with teachers to establish an information resources website for curriculums. The website contains syllabus, teaching materials, and related library resources of each curriculum, to facilitate the direct use of students, and provide a feedback channel for users.

Methods: This project was conducted in the following steps: (1) announced and accepted applications; (2) the reference librarian visited applicants (teachers) to collect the needs of the courses; (3) bibliographies of assigned journal articles, textbooks, monographs, electronic resources, and multimedia (DVDs) about each curriculum were collected; (4) created a website of this project; (5) the reference librarian went to the classroom to teach how to use the library resources ; (6) Launched the project website; (7) collecting the Feedback from faculty and students; and (8) evaluating the performance and impact of this project.

Results: In the fall semester of 2016, there were 11 curriculums with 10 teachers from 9 collages attended this project with 303 students in total registered to those classes. TMU library allocated 1 full time staff to organize related library materials, electronic resources and FAQs of those classes. And reference librarians went to the classroom for library instruction education (EndNote, PubMed, Medline, Scopus, and Interlibrary Loan…etc,). The project website (http://203.71.86.69/) includes lists of assigned articles, textbooks, monographs, electronic resources, atlas and multimedia about each curriculum and hyperlink to the e-fulltext while they are available. Teachers generally believed that the

library instruction education was useful to students. However, in order to improve the visibility of project website, it is better to be linked to My TMU2, the e-platform of learning & teaching in our university.

Conclusion: This project integrates curriculums with library resources and strengthens the relationship among faculty, students and librarians. Teachers participating in this project express their appreciation for in-class library instruction and feel it is very useful to students' learning. In the future, TMUL will try to link the project website to My TMU2 to enhance the visibility and usage rate of the resources we organized for those curriculums.

Keywords: library resources, curriculums, Taipei Medical University Library

Poster Number: 145

Time: Sunday, May 28, 2017, 2:00 PM – 2:55 PM

A Comprehensive Summary of Services and Resources Provided by Librarians in Support of Dental Education Programs in the United States and Canada

Elizabeth Stellrecht, Liaison, School of Dental Medicine, Health Sciences Library, Health Sciences Library, Buffalo, NY; **Richard McGowan**, Assistant Curator: Research Librarian, New York University School of Medicine, NYU Health Sciences Library, New York University Health Sciences Library, New York, NY; **Irene Machowa Lubker**, Research and Education Librarian, Tompkins-McCaw Library for the Health Sciences, Virginia Commonwealth University, Richmond, VA; **Nena Schvaneveldt, AHIP**, Head Librarian/Reference and Instruction Librarian, University Library, Roseman University Library, South Jordan, UT; **Susan Arnold, AHIP**, Director, WVU Health Sciences Library, Health Sciences Library, Health Sciences Library, Morgantown, WV; **Rebecca Davis**, Information Services Librarian, Wilson Dental Library, Los Angeles, CA; **Nicole Theis-Mahon**, Liaison to the School of Dentisty & HSL Collections Coordinator, Health Sciences Libraries, University of Minnesota–Minneapolis; **Elisa Cortez**, Liaison Librarian to the Schools of Dentistry and Allied Health, University Libraries, University Libraries, Loma Linda, CA; **Michael Kronenfeld**, University Librarian, A.T. Still Memorial Library, AT Still Memorial Library, Mesa, AZ

Objectives: This unique environmental scan will provide MLA members with a summary of library services and resources that support dental education and research. We will describe trends in dental librarianship as well as services and resources that can be adopted to serve the needs of health sciences education programs.

Methods: A survey will be conducted to take an inventory of services and resources that dental librarians provide. The study population consists of librarians who work in dental libraries or college / university libraries that serve dental programs. The librarians that will be surveyed are from institutions with a Commission on Dental Accreditation (CODA) accredited DMD/DDS pre-doctoral program. Currently, there are 76 such programs within the United States and Canada. Follow up phone interviews will be conducted as necessary. The data collected will be analyzed and summarized.

Keywords: library services, liaison services, dental education, trends

Poster Number: 146

Time: Monday, May 29, 2017, 2:30 PM – 3:25 PM

Bring-Your-Own-Device: Issues and Interventions in Medical Schools and Health Care Facilities

Khutsafalo K. Kadimo, Librarian, University of Botswana, University of Botswana Library, Gaborone, South-East, Botswana; **Dineo Ketshogileng**, Senior Librarian, University of Botswana Library, Department of Library Services, Gaborone, South-East, Botswana; **Ryan Littman-Quinn**, In-country

Director, Health Informatics, Botswana-UPenn Partnership, Health Informatics, Gaborone, South-East, Botswana; **Kagiso Sebina**, Data Manager, University of Botswana, Faculty of medicine, Medical Education Partnership Initiative, Gaborone, South-East, Botswana; **Kutlo Balotlegi**, Mobile Learning Technical Assistant, University of Botswana, Faculty of Medicine, Medical Education Partnership Initiative, Gaborone, South-East, Botswana; **Masego B. Kebaetse**, Lecturer, University Of Botswana, Department of Medical Education, Gaborone, South-East, Botswana

Objectives: The purpose of the study was to explore the literature to identify issues, guidelines, and or interventions related to the use of personal devices in medical education and healthcare settings.

Methods: A review of the literature was conducted using PubMed, Web of Science, and Ebscohost (Academic Search Premier, ERIC, CINAHL, and MEDLINE) with the following keywords: healthcare, mobile devices, bring-your-own-device, confidentiality, health records. Due to limited peer-reviewed journal articles, grey literature was also considered eligible for inclusion. Only literature written in English was considered for inclusion. The search yielded 1593 articles including 11 hand-searched articles. Titles and abstracts of all articles were reviewed two times, eliminating non-eligible articles and resulting in 19 articles. The fulltext review resulted in 15 eligible articles articulating issues, interventions or guidelines relating to the use of personal mobile devices (e.g. tablets, smartphones, and personal digital assistants). A review matrix was developed to record the main elements of each of the 15 articles. Once the review matrix was completed, it was reviewed for emerging themes.

Results: Several themes emerged from the analysis related to BYOD issues and interventions: device management, data management, medical applications, information technology, education and curriculum, and policy. The findings suggest that a comprehensive BYOD policy is fundamental in addressing emerging issues by providing guidance on appropriate interventions. Elements such as password protection, encryption, and applications regulation have been constantly highlighted as critical to patient health information security. Fostering appropriate mobile device use is important in reducing incidents of distracted doctoring, cross-infection, and unsecured transmission of patient health information.

Conclusion: Since the use of personal mobile devices in medical education and healthcare settings is expected to continue trending upwards, disallowing the use of personal mobile devices is close to impossible, and could limit the potential benefits to medical education and patient care. A more feasible approach discussed in the literature is developing BYOD policies and or guidelines that balance leveraging technological advances with organisational security and patient privacy. The paucity in peer reviewed literature calls for research in the development and effectiveness of policies and guidelines to pave way for a robust conversation regarding BYOD in medical education and healthcare settings.

Keywords: Medical education, healthcare facilities, bring-your-own-device, BYOD policy, mobile devices

Poster Number: 148

Time: Tuesday, May 30, 2017, 2:00 PM – 2:55 PM

Dare to Make the Dream Come True: Developing a Curriculum Mapping Tool with the School of Medicine

Erin N. Wimmer, AHIP, Teaching and Learning Librarian, Spencer S. Eccles Health Sciences Library, Eccles Health Sciences Library, Salt Lake City, UT; **Kerri Shaffer**, Director of Curriculum and Faculty Support, University of Utah, School of Medicine, Salt Lake City, UT

Objectives: Develop a curriculum mapping tool that maps to MeSH terms and SNOWMED concepts, for faculty and administrators to identify where content is being taught, and where gaps exist.

Methods: Working with School of Medicine (SOM) staff, this Library received an LSTA grant to build a natural language processor (NLP). This tool will contribute to a searchable database of curriculum

content found in the School of Medicine. Content will be tagged to medical subject headings to make searching more efficient, and to help administrators, faculty, and students identify where specific topics and competencies are being taught in the curriculum. The Department of Biomedical Informatics at this institution will create the tool, which will then integrate with a front-end interface being created by SOM IT. Once completed, the NLP will be stored on this Library's servers, and SOM faculty and administrators given access through their front-end queries.

Results: This project is ongoing, and will not be completed until July. Key advice for those interested in undertaking this kind of project in the future will be shared.

Conclusion: Careful planning and communication with the team are critical for the development of a useable NLP. Clearly defined objectives and timelines help the team stay on track, though roadblocks (both anticipated and unforeseen) are likely.

Keywords: natural language processor; curriculum map; LSTA grant; collaboration

Poster Number: 149

Time: Sunday, May 28, 2017, 2:00 PM – 2:55 PM

Broadening the Scope: Integrating an Information Science Perspective to an Online Medical Education Journal Club

Saori Wendy Herman, AHIP, Head of Education and Access Services, Health Sciences Library, Hofstra Northwell School of Medicine, Hempstead, NY

Objective: A Hofstra Northwell School of Medicine librarian collaborated with the assistant vice president of faculty development at Northwell Health to enhance an online medical education journal club's scope by integrating an information science perspective.

Methods: In early 2015, the assistant vice president of faculty development at Northwell Health created an online medical education journal club. The club meets once a month, one speaker is invited to discuss two medical education articles based on a specific theme, and the creator facilitates the discussions. Beginning in January 2016, a librarian has been added as a regular contributor to the club to integrate an information science perspective. The librarian is responsible for covering the limitations and bibliometrics of each article. In instances where a systematic review or literature review is being discussed, the librarian provides additional commentary on the search methodology. Formative data is collected from a survey that is distributed to all participants at the end of each meeting. Informal comments pertaining to the librarian's participation are collected in addition to the survey.

Results: There was a total of 205 participants over the 14 sessions conducted between April 2015 and January 2017. The participants were a mix of clinicians, librarians, and educators. The types of articles reviewed, spanned a range of medical education topics such as diversity in academic environments, unprofessional behavior, simulation, and burnout. The survey respondents self-reported that the program met its learning objectives. 59.8% of respondents intended to make changes in their clinical and/or educational environment as a result of the sessions. Informal comments pertaining to the librarian's role from both participants and presenters were positive. The librarian has seen an increase in interest and inquiries pertaining to alternative bibliometrics.

Conclusions: The online medical education journal club has been well received by participants and faculty presenters. The positive feedback as well as the steadily increasing number of participants indicates the club's success. The addition of a librarian allowed for an expanded discussion of the literature and an introduction to traditional and non-traditional bibliometrics. The club provides a valuable opportunity for continuing education, faculty development, and interdisciplinary collaboration.

Keywords: online journal club, bibliometrics, collaboration, information science, medical education

Poster Number: 150

Time: Monday, May 29, 2017, 2:30 PM – 3:25 PM

Draw! Using Art for Student and Faculty Engagement

Mickel Paris, Health Sciences Librarian, University of Pacific, Rite Aid Information, Stockton, CA; **Edward L. Rogan**, Assistant Professor, University of the Pacific, School of Pharmacy, Stockton, CA; **Cassie Etter**, Access Services Specialist, University of the Pacific, Rite Aid Information Commons, Stockton, CA; **Mary Kate Finnegan Dopkins**, Access Service Specialist, University of the Pacific, Rite Aid Information Commons, Stockton, CA; **Rachel K. Stark, AHIP**, Health Sciences Librarian, Library, California State University, Sacramento, Sacramento, CA

Introduction: The Health Sciences Library hosted an art event with the goals of increasing engagement and improving library programming. Faculty, students and staff were solicited to submit artwork incorporating topics related to the pharmacy sciences. The event, PharmArt, sought to stimulate discussion among artists and participants. The exhibit also provided pharmacy students an opportunity to interact with students in other Health Sciences disciplines and exchange cross-curricular ideas in a neutral space. Previous events have shown poor attendance, even with prizes and social media marketing. The resulting lack of engagement hindered many goals of library events, which generated the research to create PharmArt.

The objective of PharmArt was to increase student and faculty engagement with Health Sciences Library programming. The rationale for its creation was to increase participation in library programs that encourage and support students. The purpose was to provide students, faculty, and staff with new opportunities to improve creative skills, increase awareness of library programming, and allow for greater collaboration between pharmacy faculty and library staff.

Methods: A call for art submissions went out through social media accounts and print advertising displayed across campus for the PharmArt exhibit. Artists submitted information about their works through a Google submission form. Art pieces were displayed with a small card describing the art work's details. Raffle tickets were given to PharmArt exhibit attendees for a door prize and also used as voting ballot for favorite art piece. The day of the exhibit, prizes were awarded and ribbons placed. Event metrics for exhibit attendance and social media engagement were compared to the same metrics taken during the "Better Hearing and Speech" event in May 2015.

Results: Library program attendance for the PharmArt event compared against the Better Hearing & Speech event showed an overall increase of 347%. A total of 11 works of art were submitted with 44% submitted by faculty, 36% by students, and 18% by staff. Social media engagement with the library showed an increase in all metrics between the PharmArt and Better Hearing & Speech events including Blogger post hits, Facebook page shares/reactions, Facebook cumulative reach, and Facebook click-throughs.

Conclusions: Incorporating student, staff, and faculty art works into a library-hosted event significantly increased participation in a library program. Faculty, librarians and students came together and shared a common creative interest. The event increased student attendance for library programming, and increased the library's social media presence.

Keywords: Pharmacy, Faculty, Student, Engagement, Art

Poster Number: 151

Time: Tuesday, May 30, 2017, 1:00 PM – 1:55 PM

Guiding Residents for Literature Searches and Reference Management for Their Theses: Showcasing the Urgent Need for Training in a Developing Country

Vasumathi Sriganesh, Founder & Hon CEO, QMed Knowledge Foundation, Mumbai, Maharashtra, India

Objectives: Can training in literature searching and reference management, supplemented by individual guidance, help residents gain these skills for researching for their theses? In a developing country where very few residents are exposed to such learning, this study assessed the potential for such training becoming a priority to help residents acquire these skills for evidence based practice.

Methods: Population: Residents in a city of a developing nation where minimal or no training is conducted for literature searches and reference management

Methodology: Funds for this project were first raised through crowd sourcing. Heads of institutions with residency programs were contacted to ask residents to apply. Participants were chosen based on their levels of interest, and willingness to share knowledge gained, with at least one more resident. One-day workshops were conducted for small groups, to train them to search PubMed and use a Reference Manager (Mendeley). Each resident was then given additional help for his/her thesis topic, either personally, over the phone, email or short Skype sessions. Residents completed a post program feedback assessing knowledge gained and satisfaction. Their guides also were invited to fill a short survey indicating their satisfaction with the resident's references and referencing.

Keywords: Residency programs, Theses, PubMed, Reference Managers, Workshops, Training, Librarians roles

Poster Number: 152

Time: Tuesday, May 30, 2017, 2:00 PM – 2:55 PM

Librarians Flip for Students: Developing a Self-Directed and Lifelong Learning Skills Module for First- and Second-Year Medical Students

Nancy R. Glassman, AHIP, Assistant Director for Informatics; **Racheline G. Habousha**, Director; **Winifred S. King**, Web Services Librarian; **Aurelia Minuti**, Head of Reference and Educational Services; **Rachel Schwartz**, Reference Librarian; **Karen Sorensen**, Reference Librarian; D. Samuel Gottesman Library, Bronx, NY

Objectives: To develop and present training modules embedded in the first and second year curriculum for teaching medical students how to effectively search the literature and also find evidence-based articles. Using a flipped classroom approach, these modules introduce students to the process of self-directed learning to acquire skills they can use throughout their careers.

Methods: The session for first year students focused on developing fundamental searching skills. The session for second year students also covered fundamental skills but focused on searching for evidence-based medical literature. Both sessions followed a flipped classroom format. Librarians developed an interactive, multi-media, web-based tutorial for students to complete prior to the classroom session. In the tutorial, students were presented with a clinical case (via text and audio) and guided in formulating a clinical question, searching the biomedical literature with PubMed, finding relevant articles, and evaluating the results. Hands-on practice using embedded quizzes and exercises reinforced important points. Librarians reviewed quiz results and identified knowledge gaps to focus on during the hands-on classroom sessions. Students were assigned two new cases during the session. Working in groups of six, they demonstrated their new searching knowledge and skills and received feedback from their peers and facilitators.

Results: The online exercises were completed by 75% of first year students and 93% of second year students. Based on the results, librarians identified gaps in students' knowledge, such as formulating the clinical question and selecting appropriate MeSH headings. Some challenges encountered by librarians and students included problems with technology and the large dimensions and layout of the facility. A post-workshop survey was completed by 59% of students of which 93% found the online

tutorials to be “satisfactory,” “very good” or “excellent.” Notably, most students found the in-class sessions to be redundant.

Conclusion: To reduce redundancy in future classroom sessions, librarians will focus on filling in knowledge gaps detected from online exercise results and discussing searching techniques in depth. Additionally, librarians will encourage student participation by using audience response systems and introducing a gaming component. Because both groups of students received training in basic searching skills this year, librarians need to develop new content for online and classroom instruction for rising second-year students. To determine if training was effective, librarians will develop and implement an assessment tool to be administered a year later when teaching students more advanced techniques. The assessment will indicate whether students can recall and implement what they learned a year earlier.

Keywords: self-directed learning, lifelong learners, flipped classroom, EBM, medical education

Poster Number: 153

Time: Monday, May 29, 2017, 2:30 PM – 3:25 PM

Libraries Using Innovative Programming to Reach Students: Promoting Theater to Teach Medical Students about the Stigma of HIV/AIDS

Nina C. Stoyan-Rosenzweig, Senior Associate in Libraries, Health Science Center Libraries, Health Science Center Library, Gainesville, FL

Objectives: This project was part of a larger University of Florida Health Science Center Library program providing reliable information about HIV/AIDS using a variety of artistic media. This portion focused on investigating the use of theater- the play "Patient A"- to address stigma associated with the disease and especially the conflict arising from the nature of the way the disease is contracted.

Methods: This project involved a librarian working with medical students and medical school alumni who created a theater program at the medical school and staged the play twice to investigate the impact of this media and play choice on attitudes of theater goers. The librarian recruited medical students and acquired funding to cover the price of scripts and performance fees and supervised development of the student's research project. The student worked with other current students to stage the play and conduct surveys and focus groups for one staging while the other staging of the play was done with graduates of the University of Florida College of Medicine who had first staged the play to address questions of stigma and disease. This session covers the programmatic development aspect rather than the results of the research.

Results: The library staged two performances of the play "Patient A" working with medical students and medical alumni both to involve students in research projects- for a medical humanities pathway in the medical school- that complemented the Health Science Center Library's involvement in a complex project to provide reliable information on HIV/AIDS and help encourage individuals to know their status by finding ways to reduce stigma and increase knowledge. The library used a number of media including film and print materials as well as training sessions as part of this project and especially innovative use of theater to address stigma.

Conclusion: This presentation will address the work of developing and carrying out a plan to stage the play and successfully develop a plan to involve students and engage the medical school community- including medical students and graduates from the College of Medicine in production of the play that included recruiting actors for rehearsal as well as research on the impact of the play on the viewing audience.

Keywords: Theater Stigma HIV/AIDS Medical student research Library involvement medical education

Poster Number: 154

Time: Monday, May 29, 2017, 2:30 PM – 3:25 PM

Book, Brush, and Stethoscope: Introducing Art and Literature Elements within Evidence-Based Medicine Training Sessions

Alexander Lyubechansky, Clinical Librarian and Assistant Professor, Savitt Medical Library, University of Nevada, Reno School of Medicine, Las Vegas, NV; **Joanne M. Muellenbach, AHIP**, Founding Director, Health Sciences Library, UNLV Libraries, University of Nevada, Las Vegas , Las Vegas, NV

Objectives: Asking a focused, clinical question is a key element in clinical decision-making, and the first step in practicing evidence-based medicine. To help medical students, residents and health practitioners develop this skill, elements of art and literature are introduced into evidence-based medicine instruction sessions. These sessions also enhance their overall knowledge of evidence-based medicine, and how to acquire the necessary evidence.

Methods: Art, literature and medicine are intrinsically linked; the essence of all three is discovery. To this end, evidence-based medicine instruction sessions begin with the introduction of a painting or a short story. This is followed by the instructor's questions, with the purpose of stimulating an in-depth analysis and discussion. The instructor's questions are always fast-paced, prompting a brain-storming approach. Learners are encouraged to observe and analyze a work of art, or a story, and to think about the author's intentions, colors, mood, narrative content, methods of expression, style, and theme. Conclusions are reached through a shared questioning process, that allows for ambiguity, and for differences of opinion. Session evaluations and feedback shows that introducing art and literature within evidence based medicine are effective and engaging methods for learning how to ask a focused clinical questions, and for furthering clinical decision-making.

Results: *Results and Conclusions will be given in my poster at MLA '17.*

Keywords: asking questions, art, clinical decision-making, evidence-based medicine, information literacy, learning, literature, observation, teaching methods

Poster Number: 155

Time: Tuesday, May 30, 2017, 1:00 PM – 1:55 PM

Using the Association of College and Research Libraries (ACRL) Framework to Integrate Information Literacy and Evidence-Based Medicine Content within a New, Undergraduate Medical Education Curriculum

Joanne M. Muellenbach, AHIP, Founding Director, Health Sciences Library, UNLV Libraries, University of Nevada, Las Vegas , Las Vegas, NV; **Dana Thimons, AHIP**, Health Sciences Librarian, University of Las Vegas, Nevada, Las Vegas, NV; **Bredny Rodriguez, AHIP**, Health Sciences Librarian, Health Sciences Library, University of Nevada, Las Vegas, Las Vegas, NV

Objectives: Asking an answerable clinical question and acquiring the necessary information are the first two steps in the practice of evidence-based medicine, and it is important that these skills be reinforced within the medical education curricula over time. This study aims to identify the best approach for utilizing the ACRL Framework or other standards for integrating information literacy and evidence-based medicine content, within all four years of a new, undergraduate medical education curriculum.

Methods: A systematic review of the literature was conducted, searching CINAHL, EMBASE, PubMed, Scopus, Web of Science and other relevant databases. Searching was completed through February 2017. Hand searching of the Medical Library Association annual meeting abstracts through 2016 was also completed. Studies of interest focused on use of the ACRL Framework, and information literacy

and evidence-based medicine (EBM) instruction within a problem-based, undergraduate medical education curriculum. Studies of particular interest were those that discuss the ACRL Framework or Standards, information literacy and EBM instruction, and MD programs. The results of this study will inform our approach for the integration of librarian-led information literacy and evidence-based medicine instruction within a new, problem-based learning, undergraduate medical education curriculum.

Results: the results will be made available during my poster presentation at MLA '17.

Conclusion: the conclusion will be made available during my poster presentation at MLA '17.

Keywords: Association of College and Research Libraries (ACRL) framework, evidence-based medicine (EBM), information literacy, problem-based medicine (PBL), undergraduate medical education (UME)

Poster Number: 156

Time: Tuesday, May 30, 2017, 2:00 PM – 2:55 PM

Designing and Integrating Evidence-Based Medicine Tools into a Medical School Curriculum

Abraham Wheeler, AHIP, Health Sciences Librarian, Health Sciences Group, Michigan State University Libraries, East Lansing, MI

Objectives: As our medical school expanded its EBM teaching across the 4 year curriculum, we recognized the need to develop tools to teach EBM principles and practice. We developed two worksheets that meet the student at appropriate points of need in the curriculum. These tools compliment the curriculum by building critical reasoning skills that help students internalize the EBM assessment process.

Methods: Critical appraisal tools are a key learning component when teaching evidence based medicine. Previous tools we tried were too superficial, or too complex and time consuming to use. Because of this dissatisfaction, we designed two worksheets that support students' longitudinal curriculum journey from EBM novice to expert. The tools are built to compliment and reinforce their course work and emphasize different aspects of critical reasoning scaffolded to instructor and curriculum expectations. By the end of medical school the students are trained in a “Quick and Dirty” method to review a paper. Combining course instruction and EBM practice, this method equips students to evaluate a paper's relevancy to patient care and physician practice in only 5-10 minutes. We will present the various iterations of the resources and an overview of how they are implemented into the curriculum.

Keywords: EBM, critical, appraisal, evidence, curriculum, pedagogy

Poster Number: 157

Time: Sunday, May 28, 2017, 2:00 PM – 2:55 PM

Clinical Apps: Gateway to Accessing and Assessing Health Information

Margaret A. Hoogland, AHIP, Clinical Medical Librarian, Mulford Health Sciences Library, The University of Toledo, Temperance, MI

Background: Evidence Based Practice (EBP) is a major component of education in the health sciences. Despite the incorporation of tablets, apps, and discovery tools, however, the transition to using current technology and trends within EBP has not yet happened. In 2015, a Minnesota Medicine article discussed the incorporation of tablets, phones, and electronic devices into daily routine and

practice of health care professionals. Also discussed was the impact of technological changes, while still in the early stages of adoption among currently practicing health care professionals, and how these changes allow for better care of the patient.[1] If health professional students are being exposed to the Electronic Health Record, Simulation Centers, and other technology during Orientation, it seems that our focus, when discussing and teaching EBP, should include these newer technologies. Earlier exposure will decrease the anxiety that students face later in the programs and it might increase the engagement of the student with EBP principles from the outset.

[1]Baker NJ, Beattie J. How your smartphone or tablet can make you a smarter doc. Minnesota Medicine, 2015; 98(8):31-34.

Objectives: This study has two objectives. First, discover what Clinical Apps are available to students in health professional programs. Secondly, determine what (if any) activities within Evidence-Based Practice instruction mention Clinical Apps or provide activities involving Clinical Apps.

Methods: A six question survey, which was created in Survey Monkey, will be distributed electronically to members of a group interested in EBP Instruction (approximately 100 members) and active members of the Midwest Region of the Medical Library Association (approximately 300 members) during the month of March 2017.

Results: Collected Date will be analyzed and the results will be posted by mid April 2017.

Conclusion: These will be posted once the results have been analyzed.

Keywords: Evidence Based Practice, Clinical Apps, Instruction

Poster Number: 159

Time: Tuesday, May 30, 2017, 1:00 PM – 1:55 PM

Combining Research Results and Dental Accreditation Requirements to Create Instruction Opportunities

Peter Shipman, Dental Medicine and Cancer Librarian, Research and Education, Robert B. Greenblatt, MD Library, Augusta, GA

Objectives: The aim of this program is to expand librarian instructional opportunities and improve student performance in evidence-based dentistry (EBD) competencies.

Methods: A recent librarian-led internal study of EBD behaviors of fourth-year dental students at external clerkships indicated poor recall of the EBD process (question, find, appraise, act, evaluate). A dental school curriculum subcommittee, including the librarian, is currently meeting to evaluate the presence of the EBD competency in the curriculum in preparation for an accreditation review. Preliminary screening of course syllabi identified five possible courses and two EBD process rubrics supporting the EBD competency accreditation standard. A need for further EBD process training by the librarian may be indicated by the low number of rubrics which correlates to poor student recall of the EBD process in the librarian's study. Performing the study and having membership on the subcommittee gives the librarian a platform to advocate for instructional opportunities that improve student performance in the EBD competency standard prior to accreditation review.

Results: The evidence-based dentistry subcommittee of the dental school curriculum committee was able to identify three more additional courses supporting the EBD accreditation standard. Assistance in EBD supplied by the librarian for first semester, second-year dental students confirms continued poor recall of the EBD process. This cohort of dental students was not in the original research study. The taskforce could not identify any fourth-year course work that includes support of the EBD accreditation standard. In the lockstep curriculum, there are gaps in semesters when students are not being tested in the EBD process. The dental librarian added two new instructional sessions after the research study (one undergraduate, one advanced education).

Conclusion: Students are receiving exposure to EBD principles to satisfy the accreditation standard, but the lack of awareness of the EBD process indicates it may be difficult for dental schools to determine if new graduates can effectively perform evidence-based dentistry in future dental practice. The EBD taskforce believes more faculty development in EBD is necessary. The dental librarian will have a role in training faculty in evidence-based dentistry.

Keywords: Evidence-based dentistry, Dental school accreditation, Dental school curriculum revision

Poster Number: 161

Time: Sunday, May 28, 2017, 2:00 PM – 2:55 PM

Preparing for Practice: Strengthening Third-Year Medical Students' Awareness of Point-of-Care Resources

Tamara M. Nelson, Instructor/Reference Librarian, Rowland Medical Library, Jackson, MS

Objectives: To create a responsive third-year library course that prepares students for their clerkship. This course is part of a four year evidence-based medicine curriculum and was designed to strengthen students' awareness of resources available through the library that would prove useful at the point-of-care.

Methods: The library plays an intricate role in the School of Medicine's curriculum through a four-year series on evidence-based practice that is presented in conjunction with existing courses. The third-year course was embedded in the students' clerkship and an online course utilizing Canvas was created to facilitate instruction. The course consisted of two modules which covered an overview of evidence-based medicine and a hands-on activity that required students to explore the resources available through the library. Students were also given an end of course evaluation that was used to determine the overall effectiveness of the course.

Results: The end of course evaluation was used to determine the overall effectiveness of the course. There were 149 students enrolled in the course and 127 responded to the evaluation. Evaluation results showed that 87% of respondents believed the information learned through the course would be beneficial in their clerkship and 98% felt the modules were easy to navigate.

Conclusion: The third-year library course proved to be effective in preparing students for their clerkship. Student feedback were mostly favorable and constructive and provided suggestions for improvement.

Keywords: evidence-based medicine, point-of-care resources, instruction, medicine

Poster Number: 162

Time: Monday, May 29, 2017, 2:30 PM – 3:25 PM

An Inventory of Professional Nursing Association Evidence-Based Practice (EBP) Continuing Education Training and Instruction

Kerry Dhakal, Assistant Professor/Research and Education Librarian, Research and Education Department, Health Sciences Library, The Ohio State University, Columbus, OH

Objectives: To explore which professional nursing associations offer evidence-based practice (EBP) continuing education (CE) courses or other EBP training activities, and to determine if librarians are involved in instructional roles for EBP CE courses or EBP training activities offered by professional nursing associations.

Methods: Professional nursing associations' websites and conference repositories were reviewed and inventoried to identify evidence-based practice (EBP) continuing education (CE) courses or EBP

training activities. Librarian participation in these courses or activities as instructors or co-instructors was also recorded. A descriptive survey will be used to gather additional information directly from health sciences librarians to elicit further if librarians are participating in the instruction of EBP CE courses or EBP training activities offered by professional nursing associations.

Results: Will be provided on my poster at the conference.

Conclusion: Will be provided on my poster at the conference.

Keywords: evidence-based practice, EBP, continuing education, CE, training, professional nursing associations, instruction, health sciences librarians

Poster Number: 163

Time: Tuesday, May 30, 2017, 1:00 PM – 1:55 PM

Effect of Librarian Involvement on Use of Evidence-Based Resources in Small Group Case-Based Learning

Lindsay Blake, AHIP, Clinical Services Librarian, UAMS Library, University of Arkansas for the Medical Sciences, Little Rock, AR; **Julie K. Gaines**, Associate Professor; Head of the Medical Partnership Library, Augusta Univ/Univ of Georgia Medical Partnership, Augusta Univ/Univ of Georgia Medical Partnership, Athens, GA; **Elena A. Wood**, Assistant Professor, Medical College of Georgia at Augusta University, Medicine/Academic Affairs, Augusta, GA; **Thom Gaddy**, Associate Professor, Cellular Biology and Histology, Augusta University/University of Georgia Medical Partnership, Biomedical Sciences, Athens, GA; **Michael Russell**, Associate Professor, Physiology, Augusta University/University of Georgia Medical Partnership, Biomedical Sciences, Athens, GA; **Eve Gallman**, Associate Prof - Neuroscience, AU/UGA Medical Partnership, Basic Sciences, Athens, GA; **Darra Ballance**, Director, Retention Programming and Technology, Statewide AHEC Network, Augusta University, Augusta, GA

Objectives: The purpose of this project is to analyze the quality of resources to which first and second year medical students refer when giving presentations during case-based small group learning activities. The findings will inform investigators as to whether there needs to be increased emphasis on critical appraisal of resources consulted.

Methods: A team of five librarians and four medical educators from the medical college solicited over 600 student presentations medical students on two campuses, one in Augusta, Georgia, and one in Athens, Georgia, from the fall 2016 and spring 2017 semesters. Librarians in Augusta provided a two hour overview of resources at the beginning of the year, while the librarian in Athens is embedded in classes and works with the students throughout the year. The team then provided students with formal feedback on the use of resources and image in individual presentations through a review of citations. In addition, the librarians attended small group sessions throughout the year to provide informal feedback. By reviewing the cited resources students use in support of their case-based presentations, investigators will determine if students cite resources at the most appropriate level of evidence as they progress through the phase 1 and phase 2 curriculums. Exempt status was granted by the university's Institutional Review Board.

Results: Students responded well to feedback on presentations and welcomed librarians into their small group sessions. On average second year medical students used a greater range of resource types and had a higher number of citations. The use of image resources also increased in the second year, but correct citation of images did not show as great of an increase. Overall use of clinical journals and textbooks was high for all student presentations. Consumer websites were also frequently used. The clinical point of care tool UpToDate, basic science journals and textbooks, and .edu or .gov sites were heavily used in individual presentations.

Conclusion: Librarian classes, formal feedback, and presence in PBL classes helped to improve student use of evidence-based resources and correct citations of resources and images in both the first

and second years. Students were receptive to feedback and making changes to assist in improving their presentations.

Keywords: problem based learning, evidence-based, citation analysis, case-based learning, small group learning

Poster Number: 164

Time: Tuesday, May 30, 2017, 2:00 PM – 2:55 PM

Standardized Patient Encounters for First- and Second-Year Medical Students Written by Librarians, Students, and Clinicians

Lindsay Blake, AHIP, Clinical Services Librarian, UAMS Library, University of Arkansas for the Medical Sciences, Little Rock, AR; **Frances M. Yang**, Director of Evidence-Based Medicine Curriculum, Assistant Professor of Epidemiology, Medical College of Georgia, Biostatistics and Epidemiology, Augusta, GA; **Christos Hatzigeorgiou**, Chief, General Internal Medicine, Augusta University / Medical College of Georgia, University Library, Augusta, GA; **Hutton Brandon**, Second year medical student, Augusta University, Medical College of Georgia, Augusta, GA; **Benjamin Wilson**, Second Year Medical Student, Augusta University, Medical College of Georgia, Augusta, GA; **Renee Page**, Associate Dean of Curriculum, Augusta University, Medical College of Georgia, Augusta, GA

Objectives: To create realistic patient encounters for first and second year medical students that incorporate evidence based medicine (EBM) principles and steps, patient communication, research skills, and clinically relevant information resources. These exercises were presented in the first and second year evidence based medicine modules and built on the students' knowledge and practice of EBM.

Methods: The Director of the first and second year Evidence-Based Medicine module requested the Clinical Librarian expand her role in the second year EBM curriculum by creating inpatient standardized patient encounters based on her clinical rounding experience. This exercise introduces second year medical students to the clinical resources they need for their third year clerkship rotations. The clinical librarian then broadened the scope of the project into the first year and developed outpatient patient-physician encounters that walked students through the basic steps of using EBM while incorporating the three main tenets of evidence based medicine and health literacy principles. The Chief of Internal Medicine, the Associate Dean of Curriculum, and two second year medical students assisted with the formation of new scenarios. The outpatient exercises called for the students to role-play a scenario where a clinician and patient may have differing viewpoints, requiring the clinician to research and communicate suggested therapies.

Results: Overall, both the first and second year students appreciated the clinical scenarios. The EBM modules both improved overall scores from previous years. Many second year students noted that the standardized patient interaction helped them to better understand the purpose of EBM and introduce them to resources they will use in their clinical rotations. First year students felt that the role-playing gave them a concrete way to see how they would integrate EBM, biostatistics and reading the primary literature into workflows.

Conclusion: The standardized patient encounter incorporating literature for second year medical students was a successful way to introduce students to clinical databases in a real world setting. Likewise, first year medical students enjoyed patient-physician role-playing and learned about EBM principles and actions as well as health literacy principles along the way. Students felt that both exercises were enjoyable and effective learning tools. The students appreciated the interaction and hands on nature of the exercises and working closely with a clinician. The exercises will be updated and expanded with the assistance of specialty physicians in upcoming EBM modules.

Keywords: simulation, patient care, evidence-based medicine, patient communication, health literacy

Poster Number: 165

Time: Sunday, May 28, 2017, 2:00 PM – 2:55 PM

Exploring the Role of Tacit Knowledge in Evidence-Based Practice

Margaret (Peg) H. Burnette, Biomedical Sciences Librarian and Assistant Professor, University of Illinois at Urbana-Champaign, Urbana, IL

Objectives: Tacit knowledge represents an amalgam of training, skill, experience, expertise, emotions and intuition of an individual. Evidence-based practice purports to consider the evidence in equal measures with the practitioner's experience and expertise (tacit knowledge), and the patient's circumstances. The objective of this exploratory study is to investigate the literature on the role of tacit knowledge in evidence based practice.

Methods: This research will entail a review of the medical literature on the role of tacit knowledge (expertise and experience) in evidence-based practice and clinical decision making. Search terms include tacit knowledge, experience, expertise, evidence-based practice, decision making, judgment, and clinical practice. Databases will include PubMed, Scopus, Web of Science and Google Scholar. Inclusion criteria will be publications back to 1970s that address the relationship between experience/expertise/tacit knowledge to evidence-based decision making or practice. The resulting body of literature will be analyzed according to types of publication (formal as with report of research or informal as with editorial commentary), the time frame (to assess any trends that might emerge), and how tacit knowledge as expertise or experience was treated, discussed or overlooked in clinical decision making.

Results: It is expected that this review will reveal a gap in the literature of EBM that reflects the widespread lack of attention to the important role of experience and expertise in the clinical decision making.

Conclusion: Evidence-based practice has been a standard for clinical decision making that impacts patient outcomes and physician performance, and influences insurance coverage. While it is important to consider the evidence when making health care decisions, the role of tacit knowledge as reflected in individual expertise cannot be overlooked.

Keywords: tacit knowledge, experience, expertise, evidence-based medicine, evidence-based practice, decision making, judgment, clinical practice

Poster Number: 166

Time: Monday, May 29, 2017, 2:30 PM – 3:25 PM

Soothing the Nightmare: A Practice Approach to Introducing Students to American Psychological Association (APA) Style

Jill Deaver, Reference Librarian/ Assistant Professor, Lister Hill Library of the Health Sciences/ Reference Department, Birmingham, AL

Objectives: To determine if an APA activity, which asks Master's of Science in Nursing (MSN) students to engage their APA manuals during their program orientation, is an effective strategy for teaching students the practice of APA style.

Methods: A retrospective analysis was conducted to determine the most frequently asked APA questions between January 2014-May 2016. APA questions included in the analysis generated from the Ask A Librarian chat and email service, as well as the MSN online course discussion boards monitored by the nursing liaison during this time. Based on the overall analysis, a 45-minute game-driven presentation was developed and led by the nursing liaison during the Summer 2016 MSN Orientation,

held in an auditorium for roughly 600 students. Students used Post-its, provided by the liaison, to tag the most frequently used example pages based on APA questions from past semesters. Students who attended the orientation will be surveyed in February 2016 as to the usefulness of their tagged manuals. A follow-up analysis will cover Fall 2016 APA questions sent in via chat, email, and course discussion boards.

Results & Conclusions: Results and Conclusions will be made available during the presentation of this poster at MLA '17.

Keywords: APA Style; Graduate Nursing Education; Reference Librarians; Teaching Methods

Poster Number: 167

Time: Tuesday, May 30, 2017, 1:00 PM – 1:55 PM

Distance Doesn't Have to Mean Distant: A Literature Review of Embedded Librarianship in Nursing Distance Education Programs

Hannah Schilperoort, Information Services Librarian, Norris Medical Library, University of Southern California , Los Angeles, CA

Objectives: The two primary objectives of this literature review are to:

1. Determine the services, tools, and strategies that librarians have used to embed services, resources, and instruction in nursing distance education programs.

2. Provide a foundation for current and future librarians in the process of developing new services for existing and new nursing distance education programs.

Methods: Step 1: Conduct a comprehensive search in library and information databases and journals as well as medical, nursing, and allied health databases and journals for peer-reviewed articles on the subject of library services, resources, and instruction for academic nursing distance education programs. Only articles that discuss entirely online programs are included.

Step 2: Organize the findings according to types of library services, tools, and strategies used to implement services, resources, and instruction into learning management systems.

Step 3: Describe the challenges and successes librarians experience, as well as the value these experiences offer to current and future librarians who are in the process of developing new services for existing and new nursing distance education programs. This documentation is important because distance education programs are a growing trend in allied health education.

Results: Will be made available during poster presentation and MLA'17.

Conclusion: Will be made available during poster presentation and MLA'17.

Keywords: nursing, distance education, embedded librarianship, academic libraries

Poster Number: 168

Time: Tuesday, May 30, 2017, 2:00 PM – 2:55 PM

Facilitating an Evidence-Based Nursing Research Fellowship Program

Karen Wells, Manager, Libraries and Knowledge Services, SCLHS Saint Joseph Hospital, Denver, CO

Objectives: This poster details the facilitation of a new Evidence-Based Nursing Research Fellowship Program.

Methods: A team of hospital associates, comprised of a Clinical Research Librarian, Nurse Administrator, Research Director, Quality Director, Statistician, Pharmacist, Nursing Educators and Managers, and a HIPAA Compliance Officer partnered together to develop an EBN Research Fellowship Program. This partnership allowed for Librarian participation in a curriculum that taught 8 skills, one of which was Librarian-led instruction on systematic reviews and literature searching. The Research Librarian enabled Nursing associates to perform EBN systematic reviews and impact patient care at the bedside by providing a venue for nurses to answer burning clinical questions. This paper describes faculty participation, curriculum development, Library skills taught and the evaluation and subsequent intercession.

Results: The partnership improved the research fellows understanding of the systematic review process. Following proper assessment and appraisal of the evidence, nurses were able to translate the research directly to patient care and this allowed promotion of clinical EBN at the bedside. Nurses also investigated submittals of research proposals through the IRB, initiating further Nursing Research, pending results of their findings.

Conclusion: Evaluations of this program showed the fellows were overwhelmed and the 6 month instructional period was too brief. As a result, more classes were added and the program is now a 1-year commitment, with more hands-on activities, particularly in literature searching.

Keywords: Nursing Research Fellowship, Evidence Based Nursing, Hospitals, Bedside Patient Care, Systematic Review, Literature Searches, Librarian participation

Poster Number: 169

Time: Sunday, May 28, 2017, 2:00 PM – 2:55 PM

Development and Implementation of Library Modules in a 100% Online Nursing Program

Kevin Pardon, Health Sciences Librarian, Arizona State University, Dpc Library, Phoenix, AZ

Objectives: With more and more of our students enrolling in online degree programs and attending class virtually we as librarians must ensure that we are providing the same level of education, content, and service to these online students as we do to our in-person students. This poster will describe the development and implementation of multiple library modules across a 100% online RN-BSN nursing program at a large public institution.

Methods: The librarian met with and worked with instructors across three courses in the online RN-BSN program to discuss and examine current library content and instruction that already existed in these classes, as well as the need for new content and modules. An instructional scaffolding approach was settled on, where new content would be introduced progressively to students over the course of three semesters in three separate consecutive courses. In previous semesters, many faculty simply chose their own library content, linked only to the general tutorials page, or lacked any library content at all, making a new structured approach even more necessary. This poster will describe the development of these library modules in more detail, including software used and best practices, and will also focus on the implementation and lessons learned.

Results: A total of five new modules were implemented in the first two classes, while current library tutorials were kept in the third class in the sequence. The modules focused on teaching the students information literacy and database searching skills.

Conclusion: Sequencing library modules over the course of multiple semesters allowed students to build upon core knowledge that is necessary to successfully complete increasingly advanced assignments and gain research skills that can be applied in their future careers as nurses.

Keywords: Online Education, Instruction Scaffolding, Information Literacy Training

Poster Number: 170

Time: Monday, May 29, 2017, 2:30 PM – 3:25 PM

A Work in Progress (or "What Happened to the Library Orientation?"): Implementing Embedded Library Services During the First Semester of a New Online Graduate Nursing Program

Hannah Schilperoort, Information Services Librarian, Norris Medical Library, University of Southern California , Los Angeles, CA

Objectives: The purpose of this case study is to provide an overview of the process of developing embedded library services and resources within the learning management system of an online clinical graduate nursing program. The results of this study should prove useful for nursing and allied health librarians developing embedded library services in new academic online programs.

Methods: The liaison librarian for a new online graduate nursing program begins working in the new position after the first semester of the program is underway. As a result, the students and faculty begin the program without an official orientation to library services and without embedded access to library services within the course management system. This case study follows the librarian through the process of collaborating with nursing faculty and staff, learning management system staff, and other librarians and university staff to implement embedded library services within the learning management system. This study examines and reflects upon the development of embedded library services and resources during the first semester of the program and how the results shape the implementation of embedded library services, including library orientation, at the start of the second semester.

Results: Will be made available during poster presentation and MLA'17.

Conclusion: Will be made available during poster presentation and MLA'17.

Keywords: nursing, distance education, embedded librarianship, academic libraries

Poster Number: 172

Time: Tuesday, May 30, 2017, 2:00 PM – 2:55 PM

Using Reflection and Peer Discussion to Increase Engagement of Master's in Nursing Students During Library Orientation

Adelia Grabowsky, Health Sciences Librarian, Ralph Brown Draughon Library, Auburn University LIbraries, Auburn, AL

Objectives: It can be difficult to engage students at a general library orientation since they are not at point of need for using resources and opportunities for hands on learning are limited. This study seeks to discover if adding a reflective worksheet and short peer discussions during an orientation are effective in increasing student engagement with the session.

Methods: Setting/Participants: 55 newly admitted online MSN (Masters in Nursing) students attending an on-campus orientation.

Methodology: Students were provided a worksheet, asked to reflect on the last time they worked on a paper or project and write down the biggest problem/frustration they encountered. The orientation lecture included three short pauses during which students were asked to write down on the worksheet something that confused them or that might help with their previous frustration and then discuss one of the things they wrote down with a peer. The reflection worksheets were collected and responses were analyzed to establish themes among comments and questions. Themes were compared to feedback

from earlier orientations, which did not include a reflection worksheet, to determine if students were more engaged with orientation content.

Results: Fifty-four students completed the reflection worksheet. Eighty-five percent (n=46) of students were able to list something which would help with their previously stated problem/frustration. In the final section asking for comments/questions/suggestions, the majority of students (n=35) said the presentation was helpful/informative/useful but 7% (n=4) asked a question and 6% (n=3) made a suggestion. The three suggestions and one of the questions requested a recorded version of the presentation for later review. In previous MSN orientations, students were asked to list questions or suggestions on index cards at the end of the presentation. This technique resulted in very few responses (n<5) and all of the responses were variations of “it was helpful/useful/great”; there were no suggestions or questions.

Conclusions: Having students reflect on and document previous problems/frustrations experienced in the research process, then asking them to look for solutions to their problem within the presentation and to discuss what they learned with one another seemed to increase engagement among students. The collected worksheets provided evidence that students were not merely listening to the presentation but were looking for solutions to actual problems they had previously encountered. Suggestions from the worksheets also lead to the production of nine short videos covering different aspects of the presentation that students can view at point of need.

Keywords: Library Orientation, nursing students, reflection, peer discussions, MSN students

Poster Number: 173

Time: Sunday, May 28, 2017, 2:00 PM – 2:55 PM

Back to the Basics: Designing a Research Skills Course for Nurses

Christopher P. Duffy, Director, Library Services and Continuing Education, Medical Library, Robert Wood Johnson University Hospital Somerset, Somerville, NJ; **Kyle Downey**, Assistant Medical Librarian, Robert Wood Johnson University Hospital Somerset, Medical Library, Somerville, NJ

Objectives: To describe the successful creation of a educational program for staff nurses at a multi-campus, academic teaching hospital that was designed to teach them the basic skills that they need to be successful when conducting research. This program was jointly coordinated between the medical library and the nursing education department at an academic teaching hospital.

Methods: In June of 2015, an educational needs assessment was conducted by the Center for Professional Development, Innovation, and Research at an urban academic teaching hospital. One of the needs that was described by staff nurses was the need for basic education in how to conduct a literature search, how to write an abstract, and to better understand the basics of research.

With that in mind, the Director of Library Services coordinated with two Clinical Nurse Educators to design a unique half day seminar called "Research Skills for Nurses". This program taught staff nurses the basics in literature searching, appraisal of the literature, how to write an abstract, and how to navigate the IRB.

This program was so successful that it was brought to various other campuses through the hospital system.

Results: The Research Skillscourse has proven to be highly successful in teaching staff nurses at a multi-campus academic medical center. Data gained from course evaluations showed that over 86% of the nurses educated felt that the education they received would improve their skills in researching topics for their practice. There have been numerous requests for additional courses throughout the healthcare system.

Conclusion: A course designed to teach staff nurses basic research skills at a multi-campus, academic medical center, was highly successful in improving competency with regards to literature searching, literature apprasal, and basic library skills.

Keywords: Educational Seminar, Research Skills, Library and Nursing Collaboration, Nursing Education, Educational Needs Assessment

Poster Number: 174

Time: Monday, May 29, 2017, 2:30 PM – 3:25 PM

Dreaming to Extend Services, Daring to Expand Our Roles: Embedded Librarians on a Nursing Research Subcommittee

Diana Almader-Douglas, Librarian, Mayo Clinic, Hospital Library, Phoenix, AZ; **Lisa Marks, AHIP**, Director of Libraries, Mayo Clinic, Staff Library, Scottsdale, AZ

Objectives: Librarians at our institution are members of the Nursing Research Subcommittee. However, our role has evolved from just membership to partnership, as we now function as embedded librarians. Our objective is to highlight a committee-based embedded librarian program from the perspective of hospital librarians.

Methods: Embracing an opportunity to provide library services outside of library walls, we hoped to increase awareness of our libraries and services to increase library usage. Initially, we planned to contribute high quality information through personalized 'meet you where you are' reference services and literature searches. Our commitment led us to participate in monthly subcommittee meetings, volunteer opportunities with subcommittee events or tasks, and improved communication with nursing staff. Over time, our role expanded from library service providers to team partners. We proactively engage in the team work, and contribute beyond our initial goal. We also participate beyond scheduled meetings having presented posters at subcommittee-sponsored events, further strengthening our working relationships with nursing staff while improving our visibility.

Results: Our reach has expanded; we are thriving outside of the library walls. We have presented at nursing courses, workshops and work groups. We participate in Evidence Based Practice mentor meetings, and are teaching other groups how to research the evidence. We have earned the trust of the subcommittee and colleagues. We share the goals of the subcommittee as well as the mission and vision of our institution, and these shared values contribute to our success. We plan to expand embedded practice to other groups at our institution, and present a model for hospital libraries considering implementing embedded services.

Keywords: embedded librarian, library services, nursing research, subcommittee

Poster Number: 175

Time: Tuesday, May 30, 2017, 1:00 PM – 1:55 PM

Information Literacy Training to Improve Nursing Care at the Bedside: Librarian Participation in an Evidence-Based Practice Pilot and Beyond

Basia Delawska-Elliott, AHIP, Medical Librarian, Providence Health & Services, System Library Services, Portland, OR

Objectives: To support hospital-based nursing education programs, teaching information literacy to enhance evidence-based practice and promote nursing research.

Methods: Health sciences librarians are increasingly involved in educational programs designed to introduce evidence-based practice concepts to nurses. When a pilot Evidence-Based Boot Camp was proposed at Providence St. Vincent, a 4-time Magnet-designated hospital, the librarian was asked to join the instructor and mentor panel. The librarian's contribution consisted of co-presenting the

introduction to EBP, searching literature and appraising the evidence; providing tailored hands-on small-group search training; and helping nurse teams apply the evidence to EBP projects. At the conclusion of the pilot, a plus/delta analysis was conducted to evaluate the program. As a result training modules, including LIS instruction, were revamped. The pilot was a success, leading to an annual EBP Boot Camp and to a launch of a nursing research boot camp pilot. The librarian has continued involvement in the EBP Boot Camp and has been invited to co-teach in the research pilot.

Results: The first Evidence-Based Boot Camp for nurses was launched in 2015. A study conducted by the organizers showed significantly greater understanding of evidence-based practice concepts at the conclusion of the Boot Camp and a recommendation was made to continue the Boot Camp model. At the Boot Camp celebration ceremony, which included poster presentation of the projects and the discussion of challenges and lessons learned, searching and understanding the literature were identified as both the most challenging concepts and the most valuable lessons learned. Based on the instructor observation and feedback from the nurses, the process of teaching to search for evidence was expanded both conceptually, to address a preliminary scoping review, and practically, by expanding the hands-on sessions from 1 hour to 1.5 hours. The new teaching model has since been employed in the 2 following cohorts of the Evidence-Based Boot Camp and in the inaugural Nursing Research Boot Camp.

Conclusion: Librarian involvement in hospital-based nursing education programs promoted nursing information literacy and the advancement of new nursing knowledge. The evidence-based practice projects developed in the course of the Boot Camp directly impacted and improved patient care.

Keywords: information literacy instruction, evidence-based nursing, evidence-based practice, nursing research, hospital libraries

Poster Number: 176

Time: Tuesday, May 30, 2017, 2:00 PM – 2:55 PM

Does the Intention Match the Outcome? Completion Rates in a Massively Open Online Course

Molly Knapp, AHIP, Training Development Specialist, University of Utah, NNLM Training Office, Houston, TX; **Jessi Van Der Volgen, AHIP**, Assistant Director , NNLM Training Office, Eccles Health Sciences Library, Salt Lake City, UT; **Rebecca Brown**, Training Development Specialist, Spencer S. Eccles Health Sciences Library, Spencer S. Eccles Health Sciences Library, Salt Lake City, UT; **Matthew D. Steadman**, Web Software Engineer, University of Utah, Eccles Health Sciences Library - NN/LM Training Office, Taylorsville, UT; **Sarah Dickey**, Program Manager / NN/LM Training Office, University of Utah, Eccles Health Sciences Library, Salt Lake City, UT

Objectives: What do people intend to do when registering for an online class? Research suggests not everyone who signs up for a massively open online course (MOOC) actually intends to complete the course. Sometimes they just want to look at the content. We surveyed learners enrolled in a MOOC, and will analyze to what extent their intention matches their behavior.

Methods: 116 participants enrolled in a free, accredited, asynchronous, online class were required to answer one multiple-choice question prior to viewing course content. The required question: what is your intention for this course? Response options were: 1) I intend to complete all of the modules 2) I intend to complete some of the modules 3) I intend to browse the content, but not complete the modules for credit or 4) Unsure. We collected answers in the content management system. Participants completed modules in the order of their choice and received credit based on the number of modules completed. We cross-referenced data from responses to the required question with actual course completion rates and analyzed for trends.

Results: 91 participants completed the survey. Of these, 52% intended to complete all of the modules, 27% intended some, 1% intended to browse, and 20% were unsure. Actual completion rates: 19% completed all the modules, 59% some, 20% none. Completion rates drop when participants who did not

complete the required question are included (16% All; 47% Some; 38% None). Those with the intention to complete some modules had the highest rate of matching intention (68%). 57% who intended to complete all modules completed some, and half of respondents who were unsure what they would do completed some modules. On average, participants completed 5 of 13 modules.

Conclusion: Overall, completion rates for this MOOC were higher than traditional MOOC completion rates. 38% of registrants did not complete any modules. Reasons for this include time constraints and content not relevant. Future research will compare completion rates from this MOOC to a previous iteration where the intention question is excluded in order to investigate unintended causality. Does the intention match the outcome? It depends. Knowing that not all participants intend to complete the course is useful for interpreting completion rates. Those that intended to do some and actually did some and thus met their goal, would not likely be counted if strictly counting at all or none completion rates.

Keywords: “Distance learning”

instruction

MOOC

NN/LM

Assessment

Poster Number: 177

Time: Sunday, May 28, 2017, 2:00 PM – 2:55 PM

Library Interventions: Recurring Subject-Focused Webinar Instruction

Nita Mailander, AHIP, Director of Library Services, GCU Library, Grand Canyon University, Phoenix, AZ

Objectives: To describe a successful program of live, recurring, subject focused library instructional webinars, including nursing and health sciences, in an academic library.

Methods: In 2010, the Library began offering weekly library introduction webinars to complement in-person group instruction and the Ask-A-Librarian in-person and virtual services. In 2011, the first monthly subject focused webinar was developed specifically for nursing students. With attendance growing in the weekly introduction webinars and the monthly nursing webinars, additional subject areas were identified and requests for additional webinar instruction were reviewed from faculty, students, and staff. The subject focused webinar model has now expanded to include a total of ten recurring subject focused and course specific webinars. Twelve Reference Librarians identify desired learning outcomes, develop instructional content, and share teaching responsibilities for both virtual and in-person group instruction. In addition to the regular recurring library webinar instructional sessions, Reference Librarians provide customized, focused webinar sessions on specific disciplines and research skills at the request of faculty.

Results: Since 2010, the Library has offered over 850 webinar sessions with over 15,000 attendees. In addition to general subject focused webinars, in 2015, the Library began offering recurring course specific webinars that include courses on communication and information literacy, environmental science, and Christian worldview. With the collaboration and support from faculty, webinar attendance continues to grow.

Conclusion: An established recurring webinar schedule can be an effective method of providing instruction on library research and information literacy skills. Virtual delivery of group instruction extends the learning opportunities for students and faculty beyond the walls of the Library.

Keywords: Virtual learning, Distance education, Webinars, Information literacy, Instruction and teaching, Nursing education, Online learning, Academic libraries

Poster Number: 178

Time: Monday, May 29, 2017, 2:30 PM – 3:25 PM

Beyond Open Access: A Health Information Access Strategy for Africa

Toluwase V. Asubiaro, Librarian II, Systems Unit, E. Latunde Odeku Medical Library, College of Medicine, University of Ibadan, Nigeria, London, ON, Canada

Objectives: The aim of this research is first, to assess the role of open access (OA) model in health information access in Africa. Secondly, to identify the barriers to health information access in Africa under the OA model. Lastly, to recommend a health information access strategy that will employ language and media technologies and is well balanced between globalisation and localisation.

Methods: The methodology employed is desk research. Literature that was collected and reviewed included government and institutional policies on open access for health information and research articles on how OA policies have been implemented in Africa. Further, research articles on how OA has been employed for health information access were also collected and reviewed. The former set of articles were collected and reviewed in order to assess and identify barriers to health information access through OA in Africa. The new strategy of information access is situated on the premise of localization of internet, its content and information technologies for health information access in conformity with the standards and culture of Africans.

Keywords: Open access model, Health information, Africa, language technology, media technology, health information access

Poster Number: 179

Time: Tuesday, May 30, 2017, 1:00 PM – 1:55 PM

Promoting Open Access Opportunities on a University Campus: An Educational Approach

Stephanie Swanberg, AHIP, Assistant Professor, Information Literacy & eLearning Librarian, Oakland University William Beaumont School of Medicine, Rochester, MI; **Julia Rodriguez**, Associate Professor, Scholarly Communications Librarian, Oakland University, Oakland University Libraries, Rochester, MI

Objectives: With increasing pressure from their institutions, faculty are constantly seeking new ways of contributing to their fields, particularly in science and medicine. Open access has been shown to increase scholar visibility and impact, yet faculty remain uncertain about pursuing open access opportunities. Libraries are perfectly situated to educate and support open access in publishing, in the classroom, and in research.

Methods: To begin discussions of open access across the entire university, an emerging medical library and university library partnered to co-sponsor a series of educational workshops targeting faculty and tied to Open Access (OA) Week. For the last four years, an annual Open Access Week Celebration has been hosted to educate faculty, students, and librarians. Each year, an average of three educational sessions have been offered on a variety of topics linked to that year's OA Week theme. Topics included: introduction to open access, author's rights, copyright issues and fair use in the classroom, open data, impact metrics, and open educational resources. Featured speakers were librarians, clinicians, and science faculty based on their roles and expertise. This poster will describe the educational events, discuss successes and challenges, and provide strategies for other libraries to implement similar education programs on open access.

Results: Since 2013, an OA Week Celebration has been sponsored each year with a total of 12 events and 118 attendees. All events have taken place in the library and attendees have been university faculty

or staff with a large percentage being from the library. Successes have included: 1) increased awareness and knowledge of OA policies and resources by library faculty and staff who are now better equipped to handle OA questions; and 2) shared learning opportunities for faculty from different departments and a space to discuss their research and publishing activities. Challenges have included fluctuating attendance numbers, addressing faculty concerns about publishing in open access journals, and marketing these sessions to students. Beyond education, the libraries are tackling new OA initiatives including piloting open access publishing funds and adopting an OA resolution -- a pledge to publish and promote OA.

Conclusion: Libraries are truly taking the lead in promoting OA at our institutions. Starting with an educational approach can be the first step in increasing awareness and acceptance of OA on university campuses.

Keywords: open access, publishing, instruction methods, outreach, faculty, health care professionals, graduate students, medical students

Poster Number: 180

Time: Tuesday, May 30, 2017, 2:00 PM – 2:55 PM

Explorations in Scholarly Publishing: The Liaison Librarian's Role in Developing Open-Access Journals

Xan Goodman, AHIP, Health & Life Sciences Librarian, Assistant Professor, University Libraries, University Libraries, UNLV, Henderson, NV; **Samantha Godbey**, Education and Psychology Librarian, Assistant Professor, University Libraries, University of Nevada, Las Vegas, Las Vegas, NV

Objectives: Liaison librarian roles in scholarly publishing will be examined. Details about aspects of journal creation, design, indexing, schools as publishers, and journal hosting will be provided in order to empower attendees in decision-making about engaging in open-access publications with disciplinary faculty.

Methods: This presentation describes two librarians' experiences collaborating with faculty on open-access journals. The education and psychology librarian will describe her role as co-editor of a new journal with a disciplinary faculty member and share details about the development of that journal from concept to publishing. The health sciences librarian will describe facilitating the move of an established nursing journal from a well-known traditional publishing platform to the open-access bepress platform. She will describe her role in providing support for copyright questions, indexing, and authoring open-access language for the journal. The librarians will also share findings regarding indexing requirements for platforms such as PubMed, Google Scholar, Scopus, and Directory of Open Access Journals. A tool will be shared that documents the rules for these indices to enable strategic indexing of journal content.

Keywords: Open Access, Scholarly Communications, Publishing, Journal Indexing, Institutional Repositories, Collaboration

Poster Number: 181

Time: Sunday, May 28, 2017, 2:00 PM – 2:55 PM

Dare You Look the Predator in the Eye? How Librarians Are Addressing the Nightmare of Questionable Publication Practices

Kristen A. Sakmar, Research/Instruction Librarian, Shimberg Health Sciences Library, University of South Florida, Tampa, FL; **Krystal Bullers, AHIP**, Emerging Tech Librarian & Pharmacy Liaison, Shimberg Health Sciences Library, University of South Florida, Tampa, FL; **Ardis Hanson**, Assistant Director, Research & Education, Shimberg Health Sciences Library, University of South Florida, Tampa,

FL; **Allison M. Howard, AHIP**, Research and Education Librarian, Shimberg Health Sciences Library, University of South Florida, Tampa, FL; **John J. Orriola**, Assistant Librarian, Shimberg Health Sciences Library, Shimberg Health Sciences Library, Tampa, FL; **Randall L. Polo**, Research/Instruction Librarian, Shimberg Health Sciences Library, University of South Florida, Tampa, FL

Objectives: The objectives of this study were to determine awareness of predatory and other questionable publishing practices (QPPs) among health science librarians; to discover how they educate their patrons about this growing threat to scholarship; and to identify tools and strategies used to support journal validation and selection.

Methods: An online, confidential, voluntary survey was distributed via professional listservs to health science librarians. The survey instrument sought to (1) identify awareness of QPPs; (2) explore personal and institutional experiences with predatory and other unscrupulous publishing activities; and (3) examine the methods, tools, and strategies used to educate and support scholars, such as providing guidelines for evaluating journals or other support services for authors.

Results: Of the 130 respondents who completed the survey, the most commonly reported roles included research, instruction, and collection development. The top questions received from patrons about QPPs were: (1) the legitimacy of a publication and (2) the inability to find citations to a journal title in standard indexes. Between 2010 and 2014, the percentage of respondents who had heard about the issue of QPPs increased from 32% to 88%. Discussions or communications with colleagues were the most common means of learning about QPPs.

The top strategies used to promote and support legitimate publishing practices were identified as: (1) presentations and classes, (2) online guides and tutorials, and (3) appointing a scholarly communications librarian. Almost a quarter of respondents reported that their libraries were not, as yet, taking steps to counter the problem. The most commonly used tools were Beall's lists of predatory publishers and standalone journals, followed closely by the Directory of Open Access Journals.

Open-ended comments indicated the need for an authoritative single source to verify credible publications. Respondents also identified: (1) the proliferation of publications, (2) the pressure to publish for faculty promotion and tenure, and (3) the desire for rapid publication of research results as factors driving faculty to publish in predatory journals.

Conclusions: Most respondents were aware of QPPs. The majority of health science libraries are taking steps to educate their patrons and to promote legitimate publishing practices. Librarians reported using a broad spectrum of tools and strategies, although the primary tools (Beall's Lists) are no longer being maintained. Further research is needed to determine the efficacy of patron education efforts.

Keywords: Predatory journals, predatory publishers, questionable publishing practices, publication ethics, scholarly communications

Poster Number: 182

Time: Monday, May 29, 2017, 2:30 PM – 3:25 PM

Dueling Access Policies: Complying with Public and Open Access Policies on a University Campus

Bethany Myers, AHIP, Research Informationist; **Rikke S. Ogawa, AHIP**, Team Leader for Research, Instruction, and Collection Services; Louise M. Darling Biomedical Library, Los Angeles, CA

Objectives: In a large university, faculty and staff have competing priorities when publishing articles. The goal of acceptance to a prestigious journal (often defined by popularity in a field, publisher, impact factor, etc.) may be in conflict with the desire to make more research freely accessible. Institutions and funding organizations have developed policies to ensure public accessibility to published research findings. Not all policies are easily interpreted. Not all policies are enforced. To assist in providing public access to research publications from their institutions, the University of California has developed a local

repository, invested in publication harvesting software, and adopted open access policies which apply to faculty and staff in varying degrees.

Methods: Using data from the UC Publication Management and NIH Public Access Compliance Monitor, the authors compared the rate of compliance among sample UCLA departments with the UC Open Access policy and the NIH Public Access policy. Publication data was analyzed for 1) completeness of compliance with local and federal policies, 2) overlap between policies, 3) opportunities for procedural or technological improvements in compliance rates, and 4) outreach opportunities in order to increase compliance.

Results & Conclusion: Results and Conclusions will be presented at the meeting.

Keywords: public access, open access, institutional repository, scholarly communication

Poster Number: 183

Time: Sunday, May 28, 2017, 2:00 PM – 2:55 PM

Librarian Contributions to a Revamped Open-Access Public Health Journal

Darra Ballance, Director, Retention Programming and Technology, Statewide AHEC Network, Augusta University, Augusta, GA; **Kim Mears**, Scholarly Communications Librarian, Robert B. Greenblatt, MD Library, Robert B. Greenblatt, M.D. Library, Augusta, GA

Objectives: :To improve the quality of a recently revived online journal, publishing original research in public health.

Methods: A health sciences librarian who is embedded in an academic public health institute was asked to assist in the preparation of a recently revived journal for application for review by NLM and the DOAJ. The public health institute serves as the academic partner to a state public health association, which published the journal from 2006-2009. The journal was revived by the institute and association as an open access publication in 2015. The institute, and the association's board of directors, were very interested in increasing the scholarly value and impact of their publication.

Results: Reviewing NLM's application for indexing in MEDLINE led the librarian (and a colleague) to contribute to additional enhancements: the establishment of a secure archival site, selecting a Creative Commons license, adhering to Open Access ideals, and obtaining a CrossRef account for DOIs for each article. The librarian applied to Thomson/Reuters for the journal's inclusion in the Science Citation Index as a "regional journal." The journal is in final consideration for inclusion in the DOAJ; applications to NLM and Thomson are awaiting decisions. The librarian works closely with administrative staff as each issue is published to ensure DOIs are assigned correctly.

Conclusion: This ongoing project has enhanced the embedded librarian's value to the public health institute and assisted in the scholarly development of the journal. Design improvements to the web site are being suggested by the librarian and will be submitted to the institute's director. The institute has begun a research study on state public health associations and barriers to publishing state-level public health research; the librarian is included on the research team for this project as well.

Keywords: publishing, open access journals, embedded librarians, public health, online journals

Poster Number: 184

Time: Tuesday, May 30, 2017, 2:00 PM – 2:55 PM

Being a Part of the Big Picture: The Use of a Published Authors' Recognition Project by a Medical Library System to Demonstrate Value and Strengthen Its Presence within Its Institution

Ene Belleh, AHIP, Medical Librarian, Christiana Care Health System, Lewis B. Flinn Clinical Library, Newark, DE

Objectives: In the ever changing and challenging world of hospital librarianship, the need to be a part of the institution's big picture is essential for hospital libraries to demonstrate their value. To achieve this, the medical library department reinstated its published authors' recognition project in 2012 after a brief hiatus as part of research and education celebration.

Methods: The process of keeping track of institutional authors and their publications began in 2002. Published authors were tracked each calendar year and were featured the following spring in a poster listing all publications, book chapters, abstracts, and poster presentations. This poster listed all authors by name and department. In 2012, the reference librarian who serves as project manager enhanced the project by adding large individual posters highlighting 26 authors. These individual posters featured the first page of the selected article, along with the author's photograph, name, department, and abstract from their published work.

Results: The posters were displayed in a heavily used hallway during research and education month and garnered a great deal of attention. During this period the library experienced an increase in visitors to the libraries, literature search requests from current and future authors, journal article requests, and an overall use of resources and services. This project provided additional visibility for the libraries which contributed to efforts to strengthen the library's position within the institution.

Conclusion: Will be made available during poster presentation

Keywords: Publication, authors, institution, teaching, hospital, recognition, value, publishing, journal article, abstract, book chapter, presentation

Poster Number: 185

Time: Sunday, May 28, 2017, 2:00 PM – 2:55 PM

'The Publisher and Editor Regretfully Retract This Article Because...' : Teaching Retraction

Marianne Galati, Liaison Librarian, Client Relationship Management, Texas Medical Center Library, Houston, TX; **Beatriz Varman, AHIP**, Manager, Texas Medical Center Library, Client Relationship Management Department, Houston, TX

Objectives: Concerns about retractions in the biomedical literature and a bewildering array of terms and definitions have prompted consideration of what librarians need to know about retraction. Librarians are aware of this as a worrisome aspect of the business of publishing medical and scientific literature, but what do we need to know to help our faculty and students about this issue?

This poster examines the major issues around retraction of medical and scientific publications, and addresses learning objectives when planning to teach faculty or students essential facts about retraction of scientific publications.

Librarians need to

- Understand the various types of indications in the medical literature, including "retraction," "errata," "corrigenda," and "expressions of concern."
- Know how to locate the sources of policy statements on retraction as written by the medical editorial community, and individual journal editors
- Teach their patrons how to check their bibliography against current lists of retracted articles in order to avoid citing a retracted publication
- Identify resources available to learn more about scientific misconduct

Methods: Methodology: A survey of the literature on retractions will be conducted and a brief history of changes and developments will be plotted on a timeline

A survey of the written policies of PubMed and Embase has been conducted and will be summarized

Search strategies to identify retracted, errata and "expressions of concern" will be demonstrated

Conclusion: The professional librarian may help their researchers by flagging articles found with an indication of retraction, error or expression of concern. In this manner, the researcher will know which articles might need closer attention, and will have the final word as to inclusion or exclusion. Particular attention should be paid to articles with “expressions of concern,” which may progress to a full retraction, or may be removed once the concern has been addressed and resolved. When evaluating the medical literature, standards of evaluation for good evidence always apply.

Keywords: Retractions, publishing, withdrawn publications, errata, "expression of concern"

Poster Number: 186

Time: Monday, May 29, 2017, 2:30 PM – 3:25 PM

Reading for Resilience: One Way to "DO" Bibliotherapy

Sandy Iverson, Manager, Health Information & Knowledge Mobilization, Health Sciences Library, St. Michael's Hospital, Toronto, ON, Canada; **Sharon Bailey, AHIP**, Librarian, Centre for Addiction and Mental Health, CAMH Library, Toronto, ON, Canada; **Carolyn Ziegler**, Information Specialist, St. Michael's Hospital, Health Sciences Library, Toronto, ON, Canada

Objectives: Bibliotherapy can be defined as the use of literature to help deal with the challenges of life. This paper will explore the use of a particular form of bibliotherapy with two different groups of mental health patients and will include descriptions of the structure of the reading sessions, group dynamics, and the materials used to address specific topics

Methods: Librarians at an academic hospital partnered with their psychiatry department to deliver a read- aloud bibliotherapy program to mental health patients. Programs were delivered to both in-patients and members of a community based recovery program based at the hospital. Each program ran for a minimum of 6 weeks and engaged between 3 and 8 clients in each group. Peer-support workers also participated in the group sessions. Readings from literature (poetry, fiction and non-fiction) were selected and used to introduce and discuss topics such as loneliness, compassion, forgiveness, gratitude, etc. The discussions were facilitated by a librarian and a librarian/psychotherapist. Basic written evaluations were collected from participants and interviews were conducted with the peer support workers that also attended the groups. The authors will situate their paper within the greater body of literature on bibliotherapy, providing an overview of the practice.

Results: Will be made available in the MLA '17 poster onsite and in the ePoster gallery

Conclusion: Will be made available in the MLA '17 poster onsite and in the ePoster gallery

Keywords: Bibliotherapy, Mental Health, Psychiatric patients, Reading, Resilience, Consumer health

Poster Number: 187

Time: Tuesday, May 30, 2017, 1:00 PM – 1:55 PM

A Collaborative Development of an Internet-Based eHealth Intervention for Perinatal Depression

Nicole C. Strayhorn, Recent MLIS Graduate, Florida State University, Tallahassee, FL; **Gabriella Williams**, Digitization Grant Project Manager, Otto G. Richter Library, University of Miami, Miami, FL; **Sila Lott**, Faculty Librarian, Tallahassee Community College, Tallahassee, FL

Objectives: Objective: To create an internet-based ehealth intervention to increase awareness about perinatal depression and improve depressive symptoms among women in the perinatal period.

Main Purpose: Mom@Gram is an internet-based intervention tailored to share health information on perinatal depression, coping mechanisms for depression, and breastfeeding.

Research Question: What is an effective design for a perinatal depression ehealth intervention?

Methods: Methods: An extensive literature review was conducted to understand the information needs of those affected by perinatal depression and to understand effective design of ehealth interventions.

Exposure: This ehealth intervention gives medical and library professionals the opportunity to meet an unidentified information need and break treatment barriers for women by providing access to resources via website, mobile, virtual chat, and text messaging to share health information on perinatal depression, coping mechanisms, and breastfeeding consultations.

Population: The target audience are women in the perinatal period: a woman who experiences depressive symptoms and anxiety during pregnancy and after childbirth

Method of Evaluation: A qualitative survey will be conducted in the future to provide necessary feedback for the development and impact of this e-service on libraries. When live, this service can include quantitative ratings at the end of chat services for direct user feedback.

Results: The literature review indicates there is a need for improved patient knowledge and engagement to reduce depressive symptoms of women in the perinatal period. Studies have shown eHealth interventions can help reduce depressive symptoms and encourage women to seek the help they need. Therefore the goals of Mom@Gram are: (1) to reduce depressive symptoms, (2) increase screening, (3) build awareness of maternal depression, (4) improve access to mental health services, and (5) provide support. Mom@Gram was created with a public and private interface for users, leading to various technologies that may help in that capacity: namely, a patient profile, self assessment, e-diary, live chat, and resource libraries on various topics including nutrition and lactation information.

Conclusion: Our research shows the importance of understanding the development process of an ehealth intervention for the prevention and reduction of perinatal depression: (1) it clarifies the needs that have to be met for the intervention to be of use to the target population; and (2) it provides design components that could apply to the target population. In the next phase of our research, we will survey people to gauge the perceived usefulness of an ehealth intervention for perinatal depression.

Keywords: perinatal; depression; eHealth; intervention; maternal health

Poster Number: 188

Time: Tuesday, May 30, 2017, 2:00 PM – 2:55 PM

Library Guides on Disabilities: Building a Robust Resource for All

JJ Pionke, Assistant Professor and Applied Health Sciences Librarian, Social Science, Health, and Education Library at the University of Illinois Urbana Champaign, Social Sciences, Health, and Education Library at UIUC, Champaign, IL; **Jaena Manson**, Graduate Assistant, University of Illinois at Urbana-Champaign, University Library, Urbana, IL

Objectives: Utilizing the American Library Association Carnegie Whitney Grant, this project created a set of library guides around disabilities that are most commonly encountered in libraries, including Autism, Post Traumatic Stress Disorder, and physical disabilities like hearing loss and visual impairment. The guides were then assessed for impact through usage statistics and a survey.

Methods: To create the guides, a series of best practices were implemented so that the guides were accessible and minimally confusing/cognitively overloading. To assess impact and usage of the library guides on disability, usage statistics were gathered as well as responses from a short survey to determine use of the guides but also desired improvements to the guides.

Results: From the middle of October 2016 to early January 2017, the library guides were viewed about 2900 times. Eight people took the survey that was attached to each guide and provided valuable feedback in regards to not only amendments to the current guides but suggestions for new ones. There was also email feedback that reflected much of what the survey responses revealed.

Conclusion: Based on the response to the library guides from this project, there is a clear need for easily accessible and relatable information about disabilities, especially disabilities that are rarer and/or more stigmatized.

Keywords: library guides, grant, disability, assessment, outreach

Poster Number: 189

Time: Sunday, May 28, 2017, 2:00 PM – 2:55 PM

Searching for Animal Research Methodology: The Potential Impact of Differences Between the PubMed Record and Full-Text Methods Section

Emily Mazure, AHIP, Biomedical Research Liaison Librarian, Medical Center Library & Archives, Duke University, Durham, NC; **Melissa A. Ratajeski**, Coordinator of Data Management Services, Health Sciences Library System, Health Sciences Library System, Pittsburgh, PA; **Karen Gau**, Research and Education Librarian, Virginia Commonwealth University, Tompkins-McCaw Library for the Health Sciences, Richmond, VA; **Brandi Tuttle, AHIP**, Research & Education Librarian, Medical Center Library & Archives, Duke University, Durham, NC; **Erica R. Brody**, MSLS Student, Research & Education, School of Information & Library Science, Durham, NC

Objectives: Locating descriptions of methodology is challenging since PubMed does not search full-text. The objectives of this pilot study were to determine how well the title, abstract, and MeSH terms cover the content of the Methods section in in-vivo animal research articles, particularly with respect to procedures, drugs, and animal species used, and to describe how this might impact a search.

Methods: To identify a reasonable number of articles describing a limited breadth of procedures to review; a PubMed search was conducted to identify articles mentioning the animal model rhesus macaque and commonly abused substances. Other limits included: English, published since 2010, indexed in Medline, and "has abstract." From the results, a set of articles were randomly selected for inclusion. The title/abstract, MeSH terms, and Methods section of each article were reviewed and details such as: animal species, number of animals, description of housing, description of experimental procedures used, and analgesia/anesthesia were extracted. Data was analyzed using both quantitative and qualitative methods.

Keywords: Animals, abstract, methods, MeSH, subject headings, alternatives, IACUC, bibliometrics

Poster Number: 190

Time: Monday, May 29, 2017, 2:30 PM – 3:25 PM

Using Medical Subject Headings (MeSH) to Enrich Curriculum Topic Data for Curriculum Integration

Keith Engwall, AHIP, Assistant Professor, Web & Emerging Technologies Librarian, Medical Library, Oakland University William Beaumont School of Medicine, Rochester, MI; **Stephanie Swanberg, AHIP**, Assistant Professor, Information Literacy & eLearning Librarian, Oakland University William Beaumont School of Medicine, Rochester, MI; **Robin Rivest**, Director of Curriculum Data Management, Oakland University William Beaumont School of Medicine, Administration, Rochester, MI

Objectives: As educational partners, librarians have opportunities beyond instruction to engage in the development of the medical school curriculum. One such opportunity is a librarian-led project to map existing instructional topic keywords to Medical Subject Headings (MeSH) terms for the four-year curriculum. This can disambiguate content and identify related instructional sessions in the effort to locate curriculum gaps and redundancies.

Methods: The MeSH mapping project builds upon prior efforts at the medical school to accurately represent session content using topic keywords. A report containing an alphabetical list of keywords was obtained from the administrator of the curriculum management system. Librarians used these keywords to search the online MeSH thesaurus, select equivalent terms, and record them in a delimited list alongside the keyword within a spreadsheet. A simple Python script was used to parse the delimited lists and reorder the spreadsheet by MeSH term. There were numerous instances where multiple keywords were mapped to the same or closely related MeSH terms. The MeSH-keyword mapping will be applied to individual sessions, which will then be submitted for review by course directors, the committee, and faculty. This project reveals a simple way for librarians to contribute to curriculum development and analysis at their institutions.

Results: We successfully mapped MeSH terms to curriculum keywords and subsequently to individual sessions and have thus produced a dataset with enriched conceptual linkages.

Conclusion: By finding conceptual linkages between related keywords (via MeSH term overlap), we have the opportunity to identify potential relationships between sessions across the curriculum. These linkages must be vetted to ensure they are accurate, but they may allow us to determine whether these are redundancies that can be optimized or whether they are opportunities for integration between sessions and courses.

Keywords: engagement, curriculum, integration, analysis, MeSH

Poster Number: 191

Time: Tuesday, May 30, 2017, 1:00 PM – 1:55 PM

When the Doctor Is Stumped: Google and PubMed as Diagnostic Aids

Elizabeth Laera, AHIP, Medical Librarian, McMahon-Sibley Medical Library, Brookwood Baptist Health, Birmingham, AL; **D. Denise Boykin**, Physician, Brookwood Baptist Health, Department of Internal Medicine, Alabaster, AL

Objectives: To study how often Google searches performed by a PGY-3 internal medicine resident lead to the correct diagnosis in comparison to PubMed literature searches conducted by a hospital librarian.

Methods: Using 28 diagnostic cases published in the “Case Reports of the Massachusetts General Hospital” section of 2015 issues of New England Journal of Medicine, the resident used key terms to search Google while the medical librarian used a combination of key terms and controlled vocabulary, coupled with Boolean logic, to search PubMed. Both resident and librarian were blind to the differential and final diagnosis sections of the published papers. Differential diagnosis lists were separately compiled based on search results. The final published diagnosis was compared to the list and the results were analyzed for statistical and clinical significance.

Results: The main outcome measure is the percentage of correctly diagnosed cases by each researcher. Google searches led to the correct diagnosis in 14 (50%, 95% confidence interval 32% to 67%) cases while PubMed searches yielded the correct diagnosis in 19 (67.8%, 95% confidence interval 49% to 82%) cases. The results were not statistically significant, but the researchers believe them to be clinically meaningful.

Conclusion: Google searches are most helpful when trying to obtain the diagnosis of conditions with uncommon symptoms. Due to the natural limits provided by PubMed and the experience of a trained medical librarian, PubMed searches are more likely to produce a correct answer.

Keywords: literature searching, PubMed, differential diagnosis, decision support, hospital librarianship

Poster Number: 192

Time: Tuesday, May 30, 2017, 2:00 PM – 2:55 PM

Bridging the Gap: Dare to Incorporate Citation Analysis in Searching for Literature

Q. Eileen Wafford, Research Librarian, Northwestern University, Galter Health Sciences Library, Northwestern University, Chicago, IL

Objectives: Librarians participating in comprehensive literature searches are entrusted to capture all relevant literature on specific topics, which leaves many librarians wondering if their results reflect the best balance of precision and recall. Citation analysis adds objectivity to searches that will reduce uncertainties. We examine the practical application of a citation analysis methodology to enhance search strategies and identify relevant literature.

Methods: Citation analysis of academic papers is often used to compute metrics related to research impact. However, citation analysis can also be employed for comprehensive literature searches. To illustrate the value and usability of this methodology for librarians who regularly conduct literature searches but are unfamiliar with citation analysis, we explore a recent methodology introduced by Belter. The methodology is explained with a step-by-step rendering that provides clear descriptions and visualizations of complex topics, such as citation networks and bibliometric relationships. We also highlight the tools needed to successfully perform the citation analysis. We present a comparison of results retrieved using traditional searching techniques with those generated using citation analysis. This comparison highlights the benefits and challenges of this methodology and how citation analysis can be a helpful companion in comprehensive literature searches, especially for systematic reviews.

Results:

The tools required to perform the citation analysis are available to many medical librarians. The step-by-step rendering offered a practical guide that non-technical librarians can follow to manage their citations and conduct the analysis. In a comparison of the 670 citations that were retrieved using traditional searching techniques for the title and abstract screening of a systematic review with the 159 citations rendered through citation analysis, we discovered that both sets shared 25 citations. When applied to a scoping review, 152 citations were produced by traditional searching techniques compared with 210 generated from the citation analysis—there were five matching citations.

Conclusion: Incorporating this method of citation analysis will challenge many librarians to explore new ways of understanding citations and using technology. Even with low citation matches, librarians can use citation analysis to identify additional citations or complement existing search strategies. The uniqueness of each search gives librarians the opportunity to determine how to apply citation analysis results to enhance their searches.

Keywords: Literature searching, systematic reviews, citation analysis, research methodology, bibliometric relationships

Poster Number: 194

Time: Monday, May 29, 2017, 2:30 PM – 3:25 PM

Expedition Discovery: A Qualitative Voyage into Mediated Search Data

Tara R. Malone, Assistant Professor and Librarian, Reference and Instructional Services, University of Oklahoma Health Sciences Center Robert M. Bird Library, Oklahoma City, OK; **Phill Jo**, Assistant

Professor and Senior Reference Librarian, Robert M. Bird Health Sciences Library, Reference and Instructional Services, Robert M. Bird Library, Oklahoma City, OK; **Shari C. Clifton, AHIP**, Professor, Associate Director, and Head of Reference, Robert M. Bird Health Sciences Library, Reference and Instructional Services, Robert M. Bird Library, Oklahoma City, OK

Objectives: Qualitative analysis provides a range of methods with which to examine mediated search data in a manner that applies structure without losing the contextual richness of interactions. This project explores mediated search data while simultaneously evaluating qualitative analysis tools. Librarians sought to discover themes and patterns, individual stories, and areas for improvement.

Methods: Librarians in the reference and instructional services department at an academic health sciences library used three qualitative analysis tools—NVivo, Dedoose, and Voyant—to investigate mediated search data. Qualitative data for reference transactions were recorded using Springshare's LibAnswers/Reference Analytics platform. Librarians used a grounded theory methodology to guide data analysis via such strategies as thematic coding, word and phrase frequencies, and concept mapping. In addition to exploring mediated search data, each tool was evaluated and compared according to such criteria as functionality, user interface, and support/documentation.

Results: Results will be made available in the poster onsite and in the ePoster gallery

Conclusion: Conclusions will be made available in the poster onsite and in the ePoster gallery

Keywords: qualitative research, reference, mediated searching, qualitative analysis tools

Poster Number: 195

Time: Tuesday, May 30, 2017, 1:00 PM – 1:55 PM

Daring to Teach Ourselves: Self-Directed Peer Education as a Way to Increase Knowledge and Use of Databases

Rachel K. Stark, AHIP, Health Sciences Librarian, Library, California State University, Sacramento, Sacramento, CA; **Marina T. Aiello**, Mgr of Library Services, Tech and Instr Design, NCAL Libraries, Kaiser Permanente, Stockton, CA

Objectives: Librarians need to stay up to date with the new features and tools offered in databases, and for librarians new to an institution, learning how to use databases that are unfamiliar to them is a must. This poster examines the benefits of librarians teaching librarians, and explores whether peer led instruction improved the use of subscribed databases.

Methods: In 2015, in order to improve service to patrons, each hospital librarian in the group was assigned a database to become familiar with over a five month period, as well as was given a list of questions to answer regarding the features of each database with the goal of building a matrix of database features. After growing their knowledge of the assigned databases, the librarians each delivered a 30-minute presentation to the group about the uses, benefits, and offerings of their respective database, and a pre/post survey was administered to determine if the presentations increased the groups' knowledge of the assigned databases. Hospital librarians are often solo librarians with limited opportunity to learn from peers; however, a group of librarians who have access to the same databases can train each other and expand librarian knowledge of the available databases.

Keywords: Training Database Instruction Peer Education Hospital Librarian Customer Service

Poster Number: 196

Time: Tuesday, May 30, 2017, 2:00 PM – 2:55 PM

Using Major Thesaurus Terms or Title and Abstract Only Terms in Search Strategies for Systematic Reviews: The Probability of Losing Relevant References

Wichor M. Bramer, Biomedical Information Specialist, Medical Library, Medical Library, Rotterdam, Zuid-Holland, Netherlands; **Jos Kleijnen**, Director, Kleijnen Systematic Reviews Ltd, -, York, England, United Kingdom; **Oscar Franco**, Professor, Erasmus MC, University Medical Center Rotterdam, department of Epidemiology, Rotterdam, Zuid-Holland, Netherlands

Objectives: Researchers performing systematic reviews have to read many, mostly irrelevant, articles. To reduce the number of hits, thesaurus terms can be limited to major terms, or titles and abstracts alone can be searched. However, its effects on reviews are unknown. This research shows limited searches can reduce the number of hits, but still retrieve all relevant references in most reviews.

Methods: We analyzed 54 finished systematic reviews for which we had designed the searches. We adapted the original Embase and Medline searches to use major thesaurus terms, or title and abstract terms alone. Per review, we calculated the changes in the total number of articles retrieved. For each review, we checked whether all included references were retrieved by the limited searches. We did this for Embase only, and in both Embase and Medline. Thus, we determined the probability that a review would lose important references because of these search limitations. We asked the authors of the reviews about the effect missing these included references would have had on the review's quality. Would they have preferred the reduction in articles to screen knowing the cost in missed relevant references?

Results: Limiting Embase searches to major terms reduced the total number of hits by 8%. For 44 reviews all of the included references were retrieved, and for 5 reviews the relative loss was minimal (< 5%). Limiting both Medline and Embase to major terms reduced the number of hits more, and had negative consequences for less reviews than searching Embase using only title or abstract. Searching the title or abstract of both databases reduced the number of hits with 20%. This had no effect on 31 reviews, and a minimal effect on 5.

Conclusion: Limiting Embase alone to major terms does have only a limited influence on included references, but also does not reduce the total number of hits a lot. Searching both embase and Medline using title and/or abstract terms only reduces the number of hits more substantially, but this has negative consequences for the completeness of the reviews. Researchers wanting to reduce the number of hits should decide which probability is acceptable to them. When limiting searches we recommend including exhaustive terms in title or abstract and searching other databases as well.

Keywords: Review Literature as Topic; Databases, Bibliographic; Vocabulary, Controlled; Sensitivity and Specificity

Poster Number: 197

Time: Sunday, May 28, 2017, 2:00 PM – 2:55 PM

The GreyLit Report: Understanding the Challenges of Finding Grey Literature

Danielle Aloia, Collection Management Librarian, New York Medical College, Valhalla, NY; **Robin Naughton**, Head Digital, Library, The New York Academy of Medicine, New York, NY

Objectives: In order to better understand the needs of the health sciences research community, we interviewed GreyLit Report users about their challenges, tools and methods for finding grey literature. Speaking directly to librarians and researchers about their needs can help us to better understanding how researchers search and find grey literature in order to provide effective search reporting techniques.

Methods: Over the course of a week, we conducted 12 online interviews with national and international users of the GreyLit Report. We used Skype for business to hold conference calls with subscribers of the GreyLit Report and identified others by searching authors of LibGuides content for grey literature. Interviews were recorded and manually transcribed. Data was analyzed using a coding method. A literature review was also conducted analyzing the search strategies used for finding grey literature in systematic reviews.

Results: It was found that Google and website searching were the main tools that participants used to find grey literature in their field. A review of the literature found that hand searching, Google and websites were the main tools reported in systematic review research.

Conclusion: We recommend developing a search plan for finding grey literature. Decide on the resources to use before starting your search, indicate search strategies for each resource by providing name of organization or search engine, URL, dates searched, search terms used, analysis of results, how and why results were chosen and time spent. Be prepared to spend time on each step of the process.

Keywords: grey literature, user experience, search techniques, systematic reviews

Poster Number: 198

Time: Monday, May 29, 2017, 2:30 PM – 3:25 PM

Increasing Number of Databases Searched in Systematic Reviews and Meta-Analyses Between 1994 and 2014

Michael T. Lam, Candidate, Bachelor of Medical Sciences, Schulich School of Medicine & Dentistry, Western University, Ontario, Canada, Markham, ON, Canada; **Mary McDiarmid**, Librarian, Ontario Shores Centre for Mental Health Sciences, Research and Academics, Whitby, ON, Canada

Objectives: The purpose of this study was to determine whether the number of bibliographic databases used to search the health sciences literature in systematic reviews (SRs) and meta-analyses (MAs) changed over a 20-year period around the official 1995 launch of the Cochrane Database of Systematic Reviews (CDSR).

Methods: Ovid Medline was searched using a modified version of a strategy developed by The Scottish Intercollegiate Guidelines Network to identify SRs and MAs. Records from three milestone years were searched: the year immediately preceding (1994) and one (2004) and two (2014) decades following the CDSR launch. Records were sorted with randomization software. Abstracts or full texts of the records were examined to identify database usage until 100 relevant records were identified from each of the three years.

Results: The mean and median number of bibliographic databases searched in 1994, 2004, and 2014 were 1.62 and 1, 3.34 and 3, and 3.73 and 4, respectively. Studies that searched only one database decreased over the three milestone years (60% in 1994, 22% in 2004, and 10% in 2014).

Conclusion: The number of bibliographic databases searched in individual SRs and MAs increased from 1994 to 2014.

Keywords: Review Systematic Review Meta-analysis Medline Databases as topic Database, Bibliographic Evidence-based medicine

Poster Number: 199

Time: Tuesday, May 30, 2017, 1:00 PM – 1:55 PM

Snapshot of the Learning Needs, Gaps, and Interests of a Health Science Libraries Consortium

Sarah Bonato, Reference and Research Librarian, CAMH Library Services, Toronto, ON, Canada; **Jessica Babineau**, Information Specialist, Library and Information Services, University Health Network, Toronto, ON, Canada

Objectives: To describe the learning needs, gaps in knowledge and continuing education interests of a multi-institution consortium group comprised of libraries and information centres in teaching and community hospitals, public health units, educational institutions and government and non-government organizations in the health sector.

Methods: Consortium members' adherence to and promotion of professional standards in health science library services is supported by a professional practice subcommittee. The group offers opportunities for professional development and a forum for the support and exchange of resources and knowledge to support good practice to consortium members. Events have included workshops, guest speakers and round table talks on various topics of interested to the membership. Attendees are surveyed after each event. The mandate of the professional practice sub-committee and continuing education activities will be discussed. In addition, we will provide an overview of what our survey data tells us about our membership's learning needs, gaps in knowledge and continuing education interests. Challenges in providing professional development to a large, varied and multi-institutional group will be discussed

Results: results will be made available at the poster presentation

Keywords: Systematic Review Training, Librarians as Learners, Surveys, Continuing Education Interests

Poster Number: 200

Time: Tuesday, May 30, 2017, 2:00 PM – 2:55 PM

Which Information Sources Should Be Used to Identify Studies for Systematic Reviews of Economic Evaluations?

Mick Arber, Senior Information Specialist; **Hannah Wood**, Senior Information Specialist; **Julie Glanville**, Associate Director; York Health Economics Consortium, York, England, United Kingdom

Objectives: Available evidence about which information resources to search to identify economic evaluations (EEs) predates closure of NHS EED and HEED. This study aims to assess which databases are now the best sources of EEs and to identify the most efficient combination of databases.

Methods: A reference set of EEs will be gathered from systematic reviews of EEs undertaken to inform health technology assessments. We will use relative recall methodology to test the performance of information sources by calculating the number of reference set records retrieved / uniquely retrieved from each source. This allows high and low-yield sources to be identified along with degree of overlap. Yield and relative recall for each database, and combination of databases, will be presented. Resource overlap and unique yield will also be reported. We will report the characteristics of studies not included in any database studied and discuss implications for identifying this type of evidence. We will identify the resources, and combinations of resources with the highest yield of EEs. We will consider the impact of the order in which we search databases to identify the most efficient order.

Results and Conclusions: Results and conclusions will be made available during the poster presentation at MLA '17.

Keywords: Systematic review; economic evaluation; databases; search methodology; evidence-based librarianship

Poster Number: 201

Time: Sunday, May 28, 2017, 2:00 PM – 2:55 PM

Effectiveness of Citation Managers in Removing Duplicates for Known Record Discrepancies

Rachael Posey, Pharmacy Librarian, University of North Carolina at Chapel Hill, Health Sciences Library, Raleigh, NC; **Jennifer Walker**, Cancer Information Librarian, University of North Carolina at Chapel Hill, Health Sciences Library, Chapel Hill, NC

Objectives: To determine the effectiveness of different citation managers and other purpose-built duplicate removal tools for de-duplicating records in cases where there is a known problem or discrepancy between records from different bibliographic databases.

Methods: We chose a set of one hundred articles, downloaded from three databases each, for which we were aware of discrepancies among the database records. We included records with variations in titles, author names, digital object identifiers (DOIs), diacritical marks in the titles or authors, page numbers, volumes and issues, publication dates, additional symbols in the titles or abstracts, or a combination of these discrepancies. We then uploaded the records to the following citation managers and purpose-built systematic review platforms that detect duplicates: EndNote, RefWorks, F1000 Workspace, Mendeley, Covidence, and CREBP-SRA. We then compared the duplicate removal processes in each tool to determine the number of true duplicates, false positives, and false negatives identified by each tool, stratified by the type of record discrepancy, to determine which tool scored the best for each type of error and overall.

Results: Results will be made available in our MLA '17 poster onsite and in the ePoster gallery.

Conclusion: Conclusions will be made available in our MLA '17 poster onsite and in the ePoster gallery.

Keywords: systematic reviews, duplicate removal, citation management

Poster Number: 202

Time: Monday, May 29, 2017, 2:30 PM – 3:25 PM

Citation Searching and Relevance Ranking for Systematic Reviews

Christopher W. Belter, Informationist, National Institutes of Health Library, NIH Library, Silver Spring, MD

Objectives: Identifying literature for systematic reviews is a time- and labor-intensive process that requires significant amounts of time and expertise to perform. In this presentation, I summarize an alternative approach that uses advanced citation search techniques to identify literature for a systematic review and rank the search results by their relevance to the review.

Methods: The proposed method starts with one or more key documents known to be relevant to a topic and then retrieves papers (a) cited by the key paper(s), (b) citing the key paper(s), (c) that share references with the key paper(s), and (d) that are cited alongside the key paper(s). Because the same document can be retrieved multiple times in this process, the relevance rank for each document is determined by the number of times it is retrieved. To test the method, I ran a series of simulated searches in which I used the method to attempt to retrieve 401 studies included in 23 published systematic reviews. I then compared the method’s overall precision and recall to that reported in these reviews and measured the precision of the search results at each of the relevance rank levels generated by the method.

Results: The method successfully retrieved 349 (87%) of the studies included in the 23 reviews. Progressively higher relevance ranks were associated with substantially increased precision. Search results with a relevance rank of 2 or higher included 75% of the 401 studies in a total result set less than

a tenth the size of that reported in the reviews. The method was also run in less than a month for all 23 reviews and required no subject knowledge on the part of the searcher.

Conclusion: Although citation searching is unlikely to ever replace text searching for systematic reviews, the proposed method could be a useful supplement to text searching to ensure 100% recall on a particular topic. The relevance ranks it generates could also aid the literature screening process by giving screeners a smaller subset of search results to focus on. It could also be used to generate or facilitate rapid literature reviews in non-systematic review settings.

Keywords: Systematic reviews, literature searching, citation analysis, information retrieval, relevance ranking

Poster Number: 203

Time: Tuesday, May 30, 2017, 1:00 PM – 1:55 PM

Roles for Librarians in Systematic Reviews over Time

Angela Spencer, Manager - Medical Library, C. Alan McAfee MD Medical Library, Chesterfield, MO; **Jonathan D. Eldredge, AHIP**, Associate Professor, Health Sciences Library and Informatics Center/Family & Community Medicine, Medical library, Albuquerque, NM

ABSTRACT

Objective: To find and document roles librarians have played in systematic reviews as a means to project future roles and possible areas of needed research. Health science librarians have been involved with systematic reviews since the 1990s. Two case studies published in the mid-2000s described possible roles for librarians in systematic reviews, but were limited to the authors' circumstances. Librarians are increasingly called upon to be involved in systematic reviews so this inventory of roles should aid in describing their services.

Methods: Scoping review. The authors conducted controlled vocabulary and textword searches in *PubMed*™, *Library, Information Science & Technology Abstracts (LISTA)*, and *Cumulative Index to Nursing and Allied Health Literature (CINAHL)*. They also searched individual article PDFs using the truncated term systema* for each issue of *Journal of the European Association for Health Information and Libraries (JEAHIL.* The authors pulled up each PDF from the years 1997-2016 in *JEAHIL* using the search feature in the PDF viewer and searched the term systematic. The authors also did additional scan of JCHLA due to the way some of the articles were indexed. The authors also reviewed past MLA annual meeting poster and paper abstracts for the years 2001-2016.

Results: The authors identified 19 different roles by librarians and other information professionals in conducting a systematic review. Some roles were well-known such as searching, selecting databases, utilizing technology tools, and documentation. Other roles were less well-known such as question formulation, evaluation, indexing and citation management

Keywords: systematic reviews, roles, librarians

Poster Number: 204

Time: Tuesday, May 30, 2017, 2:00 PM – 2:55 PM

The Effectiveness of a Hands-On Systematic Review Course: Pilot Project Findings

Angela C. Hardi, Clinical Resources Librarian, Bernard Becker Medical Library, Bernard Becker Medical Library, St. Louis, MO; **Lauren H. Yaeger**, Clinical Librarian, Medical Library, St. Louis Children, St. Louis Children's Hospital in partnership with Washington University School of Medicine,

Saint Louis, MO; **Susan A. Fowler**, Library Director, Brown School Social Work and Public Health, Washington University in St. Louis, St. Louis, MO; **Michelle M. Doering**, Clinical Libarian, Washington University School of Medicine in St. Louis, Bernard Becker Medical Library, St. Louis, MO

Objectives: An academic medical librarian team hypothesizes that a hands-on, mixed-pedagogy-model course is an effective way to teach librarians how to design searches for systematic reviews. Included hands-on activities allow attendees to complete a systematic review search strategy by the course's conclusion. Participants will take skills and knowledge-based tests, along with self-efficacy assessments to determine the effectiveness of this training model.

Methods: A pilot version of the course will be offered to a group of local librarians. The course is designed with a mixed model of teaching methods including problem-based learning, team-based learning, and standard lectures. Hands-on activities are emphasized with the end goal that students will feel confident and be capable of independently conducting systematic review searches. To determine the effectiveness of this model, including teaching methods and activities, students will be assessed before and after the course. Students will take pre and post-tests to measure differences in their knowledge, skills, and perceived confidence pertaining to designing a systematic review search. Data will be analyzed using a paired T-test. Finally, attendees will be asked to complete 1-week post and 6-month post assessments to help determine the potential benefits and drawbacks of the course and training model.

Results: Results from our study are still being compiled and will be presented at the MLA conference.

Conclusion: We believe our hands-on course will be an effective tool for teaching other librarians how to conduct systematic review literature searches, but we have not compiled our data yet and will present our final conclusion at the MLA conference.

Keywords: Systematic Reviews, Training, Professional Development, Course Development, Effectiveness Research

Poster Number: 205

Time: Sunday, May 28, 2017, 2:00 PM – 2:55 PM

Research Guide in Your Pocket: Assessing the Usability of a Research Guide Optimized for Mobile Devices

Ariel Pomputius, AHIP, Health Sciences Liaison Librarian, University of Florida, Health Science Center Library, Gainesville, FL; **Hannah F. Norton, AHIP**, Interim Fackler Director, Associate University Librarian, Health Science Center Library, Biomedical and Health Information Services, University of Florida–Gainesville; **Rae Jesano, AHIP**, Reference and Liaison Librarian, Health Science Center Libraries, Health Science Center Libraries, Gainesville, FL; **Mary E. Edwards, AHIP**, Reference & Liaison Librarian, Health Science Center Library, Biomedical and Health Information Services, Health Science Center Library, Gainesville, FL

Objectives: As more library users in the health sciences use smaller devices to access information on the go, Springshare's LibGuides software has developed a more responsive design for viewing research guides on mobile devices. This research project surveyed library users to compare the usability of research guides viewed on a mobile device but optimized according to different standards of best practice.

Methods: While conducting research on best practice guidelines for a research guide on a mobile device, librarians from the University of Florida Health Science Center Library discovered that little research existed on the topic. Best practice guidelines for both desktop-optimized and mobile-optimized research guides were developed from a review of past studies on research guide usability, mobile website design, and recommended practices for responsive design. An existing LibGuide meant for use on a mobile device, Mobile Resources for Health, was duplicated and both copies were adapted

according to these best practice guidelines: one guide was optimized according to the best practices for mobile access and the other was optimized according to the best practices for desktop use. Library users were asked to explore the guides on a mobile device and their collective response indicated ease of use for each guide.

Results: Although this survey is currently active and results have not been finalized, survey respondents have provided interesting feedback on their ability to navigate both the desktop-optimized and mobile-optimized research guides. A few users reported confusion in navigating both guides, but user experience thus far has been more positive with the mobile-optimized guide. According to early results, users had a better experience finding specific apps using the mobile-optimized guide than the desktop-optimized guide. More data will be collected and analyzed in order to clarify these preliminary results.

Conclusion: Based on some confused responses regarding navigation, the research guide may warrant further assessment and usability testing, including heat maps and recorded user interaction, in order to determine what areas specifically proved most challenging. In addition, the survey has raised interesting questions about the content of the Mobile Resources for Health research guide. Write-in answers on the most useful and least useful aspects of the guide pointed to areas of future improvement in what apps were included and how they were presented.

Keywords: LibGuides, Research Guides, Responsive Design, Mobile Devices, Mobile Technology

Poster Number: 207

Time: Tuesday, May 30, 2017, 1:00 PM – 1:55 PM

Where Do They Get Those Wonderful Toys? The Continuing Adventures of a Library Tech Team Implementing New Technologies in a Collaborative Learning Space

Jason Lilly, Library Systems Analyst, Ruth Lilly Medical Library, Indiana University School of Medicine, Indianapolis, IN; **Jennifer Herron**, Emerging Technologies Librarian, Ruth Lilly Medical Library, Ruth Lilly Medical Library, Indianapolis, IN; **Kellie Kaneshiro, AHIP**, Library Technology Team Leader, Ruth Lilly Medical Library, Ruth Lilly Medical Library, Indianapolis, IN; **Gabriel Rios**, Library Director, Indiana University School of Medicine, Ruth Lilly Medical Library, Indianapolis, IN

Objectives: As part of our school's accreditation process, a need for more collaborative learning space on campus was identified. Our library tech team, consisting of our Library Director, Library Tech Team Leader, Library Systems Analyst, and Emerging Technologies Librarian initiated a plan to address this need and finishing touches were recently completed on our new space 'The Nexus: Student Collaborative Learning'.

Methods: The Nexus, Student Collaborative Learning lab is a large space with mobile furniture to allow students to configure the room in ways most conducive to their learning needs. We've not only conceived of the space for collaboration among students but also as a gateway to introduce them to new technologies. Currently we have two mobile media: scapes to encourage group work, a small 3d print lab with three printers , a 98 inch mobile touch display on which we're running a pilot for the University of the 3d anatomy software BodyViz, and our pride and joy, our touch-enabled IQ Visualization Wall. We're looking into VR headsets and how these might be applied to medical education in our environment. It truly has been an adventure and we've enjoyed seeing our students engaged by and engaging with all our new toys.

Results: As the space has been open longer, we have noticed students drawn to it to do group work while utilizing the IQ Wall. We've documented more logins on the wall and more activity in the room based on headcounts taken by student workers. The 3D printing is starting to establish itself as a popular resource and we are sending models to our campuses across the state. We've held multiple tech talks in the space as well as more technology focused library classes. We'll be running a pilot of

our BodyViz implementation with medical students in Anatomy in coordination with their faculty. We've also been engaged with the Technology in Medicine Student Interest Group to push out and hold events in the space.

Conclusion: While The Nexus can be considered a completed space, our team's focus on incorporating new technologies in the area will be a continuing journey.

Keywords: 3d printing, technology, visualization, collaboration

Poster Number: 208

Time: Tuesday, May 30, 2017, 2:00 PM – 2:55 PM

Streamlining the Library System: Preparing for the Next Generation

Natalie Logue, Access Services Librarian; **Sandra L. Bandy, AHIP**, Chair, Content Management; Augusta University, Robert B. Greenblatt, M.D. Library, Augusta, GA

Objectives: This presentation examines challenges the only health sciences library system faced during the migration of a state-wide consortium from Ex Libris Voyager to Alma. Alma is a cloud-based system constructed for print, OER, and digital collections that will allow the institution to grow beyond Voyager, which is nearing the end of its lifecycle.

Methods: A library team of Access Services and Content Management librarians and staff was formed to manage the data cleanup and implementation process. Members of this team were also involved in consortium committees which communicated problems and solutions between the 29 consortium institutions. Ex Libris provided the library with 59 data cleanup tasks and accompanying SQL report queries to systematically evaluate the library's current integrated library system. The implementation team met weekly to review reports and determine best practices for the data to migrate correctly over to Alma. Data cleanup reports indicated that several groups of data were inconsistent with the libraries best practices within patron records and cataloging records including item holdings. Report findings identified instances of human error, opportunities for improved organization, and methods of preservation for NLM classification cataloging records.

Results: Of the 59 data cleanup tasks, 39 were determined to be applicable to the health sciences library. Best practices established include reducing the number of patron groups, item locations, and item types. Some cleanup tasks required little work as others were more time consuming. The library identified over 500 patron records that were duplicate or inactive for three or more years and were deleted from the current system. Over 700 cataloging records with mismatched locations and holding records with missing items were identified and corrected. Two solutions were also identified to insure the health sciences NLM classification subject headings would migrate over to the Alma environment.

Conclusion: The Alma ILS is built to accommodate physical and electronic records and manage patron records externally. The data cleanup tasks most significantly prepared library data to migrate successfully into a new organization and cataloging model, but also established new best practices for Alma and eliminated messy and outdated records.

Preparing to migrate from one integrated library system to another, the library reviewed numerous patron records and cataloging records cleaning them up so unnecessary data would not transfer. Upon completing these tasks, the library is equipped to move to the next step of validating content in the new system.

Keywords: Alma, Integrated Library System, Data Cleanup

Poster Number: 209

Time: Sunday, May 28, 2017, 2:00 PM – 2:55 PM

The Anatomage Table: An Innovative Approach to Anatomy Education

Merle Rosenzweig, Informationist, Taubman Health Sciences Library, Taubman Health Sciences Library, Ann Arbor, MI; **Mark P. MacEachern**, Informationist, Taubman Health Sciences Library, Taubman Health Sciences Library, Ann Arbor, MI; **Chase Masters**, Enabling Technologies Informationist, Taubman Health Sciences Library, University of Michigan–Ann Arbor

Objectives: The Anatomage Table, a virtual dissection resource, is a visualization system of the human body and is being adopted by many leading medical schools and institutions as an innovative approach to teaching anatomy. This poster focuses on how our institution is integrating the Table into the curriculum to facilitate, enhance, and improve anatomy education in the field of anatomy.

Methods: We gathered information from our School of Kinesiology and our School of Dentistry on how they are using The Table in anatomy classes. In addition to the human models—both male and female, The Table has several animal models that can be used by our basic science faculty and students.

Results: Since The Table can be connected to a projector, lectures are more dynamic and the students are more engaged. Our table is setup in the public area of our Health Sciences Library and is available during hours of operation. Unlike using a cadaver or animal model, The Table's virtual models allow for repetition of procedures.

Conclusion: 21st century medical education is embracing technology. Access to journal information and textbooks are delivered to our uses electronically. The Anatomage Table is another tool that fits into this.

Keywords: Anatomage, Anatomy Education

Poster Number: 210

Time: Monday, May 29, 2017, 2:30 PM – 3:25 PM

Dreaming of the Perfect Fit: A Structured Evaluation of Three Reference Management Tools That Support Collaborative Research

Erica R. Brody, MSLS Student, Research & Education, School of Information & Library Science, Durham, NC; **Kathleen McGraw**, Assistant Department Head, User Services and Dentistry Librarian, Health Sciences Library, Health Sciences Library/User Services Department, Chapel Hill, NC; **Barbara Rochen. Renner**, Library Services Evaluation Specialist and Liaison, Allied Health Sciences and Professor, Department of Allied Health Sciences, School of Medicine, Health Sciences Library, University of North Carolina at Chapel Hill, Chapel Hill, NC

Background: F1000 Workspace, Mendeley, EndNote Basic (without Web of Science), and EndNote Desktop/Online are tools that facilitate building shared libraries of references with attached PDFs for groups such as research labs, medical residents and co-authors. This evaluation compares specific functionality of these tools to help librarians match recommendations with user needs.

Methods: The structured evaluation includes these metrics: sharing; access; importing citations, PDFs, and databases; duplicate removal; ability to organize and make notes; ease of installing a word plugin; styles available for bibliography references; ability to upload and track versions of a co-authored article; and product support.

Results: *Sharing*: F1000W and EndNote offer unlimited shared "projects" and "groups," respectively, although whole libraries may not be shared with EndNote Basic. EndNote desktop allows syncing one library with an online account. All the references in this library can be shared from the desktop with other EndNote v.7+ users, or by sharing groups within the library via the online account. Desktop users can accept unlimited library sharing invitations. Free Mendeley accounts offer one private group with 3 members. An institutional license provides unlimited groups with 25 members. *Access*: F1000W and

EndNote Basic are entirely web based. Mendeley and EndNote are desktop based with online interfaces. *Importing*: All programs have browser web importers and import files in a variety of standard formats and, with the exception of EndNote Basic, will create records from PDFs of articles with DOIs. *Deduplicating:* F1000W merges exact duplicates on import. All products have tools to identify and merge duplicates upon verification. *Organizing in shared groups*: Mendeley has shared multi-level folders and tags; other products share just folders. *Notes*: All products, with the exception of EndNote Basic, provide highlighting and notes within stored PDFs. Mendeley and F1000W display PDF notes in the records and notes can be directly added to the records. *Citing tool*: Word plugins are easy to install for all programs. F1000W has a Google Docs add-in. *Styles*: EndNote Basic has 21 styles, while the other programs provide hundreds. Editing styles is available via support request in F1000W, in EndNote Desktop but not in EndNote Basic, and in Mendeley for those with scripting skills. *Manuscripts*: F1000W has a manuscript sharing tool with version tracking. *Support:* Response time to support requests are shortest in F1000W.

Conclusions: The poster details user needs and tool recommendations in 4 cases: medical resident, public health student, systematic review team and research lab.

Keywords: Reference management, citing and writing

Poster Number: 211

Time: Tuesday, May 30, 2017, 2:00 PM – 2:55 PM

Dare to Change: Moving from Internet Protocol (IP) Authentication

Amy Allison, AHIP, Associate Director, Woodruff Health Sciences Center Library, Woodruff Health Sciences Center Library, Atlanta, GA; **Sandra Franklin, AHIP**, Director, Emory University, Woodruff Health Sciences Center Library, Atlanta, GA; **Lisa Macklin**, Director, Emory University, Robert W. Woodruff Library / Scholarly Communications Office, Atlanta, GA

Objective: The libraries at one university identified a need to change the way users access licensed online content, such as databases, eJournals and eBooks, because the current method of IP authentication proved unreliable and unsecure for the network. The objective was to move to a system of authenticating authorized users by unique user network ID both on and off campus.

Methods: The project team surveyed other libraries to identify how they authenticate and authorize users to access online resources. Based on these results and a simultaneous campus IT security initiative, the team selected a combination of Shibboleth and EZProxy. The team developed a timeline for testing and implementing the system and defined user populations authorized to access specific resources. Next, the team created a communication plan with three objectives: 1) obtaining buy-in from key stakeholders, 2) informing users of the impending change to how they will access online resources, and 3) providing service desks with talking points and training to respond to questions. The team revised the policies governing walk-in user access. An inventory of publishers was prepared to inform them of the change from the entire IP address range to the specific proxy server IPs.

Results and Lessons Learned: In November 2014, publishers and vendors were notified of the change. Beginning January 2015 university and health system users were required to authenticate, even when using the university network. Beginning May 2015, health system employees were restricted to selected resources.

The change affected all university and health system users, as well as visitors and non-Emory individuals working on campus. For assistance with the new system, users were directed to two virtual service points, where 145 requests related to the new system were received January-July 2015. Additional requests were received in person or via phone or direct email to library staff.

Although authenticating to access resources is commonplace for most library users, changing the way users authenticate can be monumental. It is critical that the institution's leadership understand and support the change. The project should explore the variety of ways users might access licensed

content, including directly accessing publishers' websites or using third party applications. Communication with all stakeholders before and after implementation is essential and must be consistent. Involving key non-library stakeholders in developing communications facilitates messaging that is clear, acceptable, and distributed through effective channels.

Keywords: Change, leadership, planning, decision-making, technology, electronic resources, authentication

Poster Number: 212

Time: Tuesday, May 30, 2017, 2:00 PM – 2:55 PM

Makerspace Mania! Developing a Makerspace in a Health Sciences Library

Kim Mears, Scholarly Communications Librarian, Robert B. Greenblatt, MD Library, Robert B. Greenblatt, M.D. Library, Augusta, GA; **Natalie Logue**, Access Services Librarian, Augusta University, Robert B. Greenblatt, M.D. Library, Augusta, GA; **Gail Kouame**, Chair, Research & Education Services, Robert B. Greenblatt, M.D. Library, Augusta University, Augusta, GA

Objectives: To advance the clinical and educational objectives of the university and to foster innovation by developing a makerspace in the health sciences library.

Methods: A committee of three librarians investigated models of makerspaces in health sciences libraries and evaluated the current needs of faculty and staff for 3D printing and data visualization. A proposal was developed in three phases: information gathering, in-depth interviews, and cost-benefit analysis of equipment and program development.

Results: During the information gathering stage, the committee reviewed a variety of sources such as websites, white papers, and listserv discussion threads on makerspaces in health sciences and academic libraries. Interviews were conducted with educational technology staff, health sciences faculty, research administration personnel, and a community technology hub. Equipment recommendations were selected during the cost-benefit analysis, which weighed the initial and continuing costs of equipment, the long-term goals of the makerspace, and the needs of the students and faculty. Location and training needs were also included in the proposal recommendations. Implementation of the makerspace is ongoing.

Conclusions: Health sciences libraries are becoming more active in the development and implementation of makerspaces in health sciences libraries. Future considerations for the Greenblatt Library makerspace include outreach and promotion and the development of a sustainable funding model.

Keywords: makerspace; innovation; proposal; technology; 3D printing

Poster Number: 213

Time: Sunday, May 28, 2017, 2:00 PM – 2:55 PM

No Time, No Budget: Facilitating Mapping by Community Health Organizations

Colette Hochstein, Technical Information specialist, National Library of Medicine, Specialized Information Services, Bethesda, MD; **Gale Dutcher, AHIP**, Retired Acting Associate Director, Specialized Information Services, Specialized Information Services, Bethesda, MD; **Janice E. Kelly, FMLA**, Acting Deputy Associate Director, Specialized Information Services, Specialized Information Services, Bethesda, MD; **Alla Keselman**, Senior Social Science Analyst, Specialized Information Services, Bethesda, MD; **Kurt Menke**, Certified GIS Professional (GISP), Bird's Eye View, Bird's Eye View, Albuquerque, NM; **John C. Scott**, President, Center for Public Service Communications, Center

for Public Service Communications, Claiborne, MD; **Jennifer Dong**, Computer Scientist, National Library of Medicine, Division of Specialized Information Services, Bethesda, MD

Objectives: This poster discusses approaches taken by the Community Health Maps blog to improve access to accurate, high quality health information, especially by under-served and special populations. By conveying potential uses of mapping to community groups unfamiliar with the technology and by supporting those already engaged in mapping, the blog enhances community mapping efforts that support access to quality health information.

Methods: A department of a federal institution collaborated with a certified GIS professional and a health-centered organization to develop a blog which simplifies the use of GIS, especially for community organizations, libraries, schools, and non-profits. The blog provides information about free and inexpensive mapping tools, software reviews, best practices, and train-the-trainer workshops. It also relays the experiences of those who have successfully implemented its mapping procedures. The site offers a series of written "lab" exercises, also available via YouTube, which take users through a carefully prescribed workflow covering collecting field data, bringing the data into an open-source desktop GIS package, conducting basic spatial analyses, uploading and styling data, and publishing a map. It provides a vehicle for sharing "real-life" projects that have been successfully undertaken with these tools and the workflow, which in turn bolsters GIS novices' confidence and motivation.

Keywords: map, underserved, GIS, community health, low-cost, no-cost, free, mapping tools

Poster Number: 214

Time: Monday, May 29, 2017, 2:30 PM – 3:25 PM

Dream It, Then Do It in the Media Design and Innovation Lab

Erin N. Wimmer, AHIP, Teaching and Learning Librarian; **Nancy Lombardo, AHIP**, Assoc Director for Digital Collections; Spencer S. Eccles Health Sciences Library, Eccles Health Sciences Library, Salt Lake City, UT

Objectives: Develop a creative technology space, available to faculty and students across the health sciences, in which to design innovative digital learning objects and media for integration in the curriculum.

Methods: There are no freely available spaces on this institution's health sciences campus for faculty and students to create digital learning objects and media.

As an educational technology hub for the health sciences community, this Library created a Media Design and Innovation Lab (MDIL) to fill this need.

Equipment and software appropriate for innovative teaching and learning were identified and evaluated. Faculty across the health sciences campus consulted to determine specific wants and needs for the MDIL. The space was customized to meet as many of these needs as possible.

Library faculty and staff became "experts" on software and web tools that were added to computers in the MDIL. These experts taught their colleagues how to use these programs in order to instruct and support health sciences faculty and staff as they design and create using the MDIL.

Results: This project is ongoing. News of the future availability of the MDIL is met with excitement by stakeholders from across the Health Sciences. The space has been suggested for a variety of future projects for both faculty and students. Key advice for those interested in creating this kind of space in the future will be shared.

Conclusion: Creating a Media Design and Innovation Lab is an exciting way to engage stakeholders in the creation of digital learning objects.

Keywords: media development; curriculum development; digital learning objects; educational technology; innovative design

Poster Number: 215

Time: Tuesday, May 30, 2017, 1:00 PM – 1:55 PM

Media Explorers: Does Your Library Need a Media Development Team?

Kai Donovan, Media Developer, Taubman Health Sciences Library, University of Michigan, Ann Arbor, MI; **Elise Wescom**, Media Assistant/Graphic Artist, University of Michigan, Taubman Health Sciencs Library, Ann Arbor, MI; **Jean Song**, Assistant Director, Academic and Clinical Engagement, Taubman Health Sciences Library, Taubman Health Sciences Library, Ann Arbor, MI

Objectives: This poster charts the birth, growth, and integration of the Library's Media Development team from instructional videos to conference logos and comics in medicine.

Methods: What do you do with technical processing staff and shelving assistants when your library is transitioning to a bookless library? Do they have an interest in multimedia and art? If they do, then you may be able to transform them into a Media Development team! Our library did just that with one staff member and one temporary assistant who learned how to create and incorporate photographs, videos, illustrations and comics into the health sciences curriculum, workshops, and special projects as a Media Developer and Media Assistant/Graphic Artist. The Library's Media Development team has branched out into new and exciting partnerships with internal staff and faculty in the last eight years. We will describe our journey and most exciting projects as an integral unit of the Library.

Results: We will highlight our most successful projects and demonstrate areas where a media development team can benefit academic health sciences libraries.

Conclusion: This poster will be of interest to any academic health sciences library considering increasing their multimedia assets and possibly establishing a media development team.

Keywords: media; media development; video; photography; illustration; comics

Poster Number: 216

Time: Tuesday, May 30, 2017, 2:00 PM – 2:55 PM

MedlinePlus Connect: Analysis of User Needs

Loan Nguyen, Informationist, National Institutes of Health Library, NIH Library, Bethesda, MD; **Katherine Masterton**, Librarian, National Library of Medicine, Public Services Division, Bethesda, MD; **Jennifer Jentsch**, Project Manager, MedlinePlus Connect, National Institutes of Health, National Library of Medicine, Bethesda, MD

Objective: MedlinePlus Connect is a free service linking health IT systems, patient portals and electronic health record (EHR) systems to targeted, authoritative patient health information from the MedlinePlus.gov website. Since its launch in 2010, no regular process exists to collect user feedback. The objective was to understand the needs of the MedlinePlus Connect users to improve and enhance services.

Methods: This study was a qualitative design. Fifty-one users were identified from a comprehensive list of MedlinePlus Connect users, as well as referrals from the MedlinePlus Connect team members. Of the 51 invited to participate in an interview, eleven interviews were completed using a semi-structured questionnaire focusing on implementation, use and areas for growth. Descriptive information and common themes were analyzed from the recorded interviews.

Results: Participants came from a variety of organizations, ranging from international health IT vendors to small specialized companies in behavioral health. Eight of the vendors are using MedlinePlus Connect to meet Meaningful Use requirements. Other uses include an online medical billing code database, an online risk assessment tool, and a Personal Health Record (PHR) application.

Implementation of the API was unanimously an easy and straightforward task, with “no problem at all” as the general consensus. Depending on the skill and number of personnel, time to implement ranged from a couple of hours to four weeks. Seven of the eleven participants (63%) were not on the mailing list or not aware of its existence. However, five of the seven (71%) wanted to receive more information to get on the list. The primary reasons for choosing MedlinePlus Connect were to meet Meaningful Use certification and that it is a free service. Some comments for improvement include adding more languages, expanding content in wellness and alternative medicine, applying clinical decision support rules, adding CPT codes, lower literacy materials, online gaming, and more information geared for the clinicians.

Conclusion: This study is the first formal endeavor to collect user feedback since the launch of MedlinePlus Connect in 2010. Broadly, MedlinePlus Connect is well received by customers; used primarily to meet Meaningful Use certification and easy to implement within an electronic health system. Recommendations for future efforts include a marketing campaign to promote the listserv, produce materials in more languages particularly in French, and conducting a user feedback study similar to this one every two years.

Keywords: MedlinePlus Connect, EHR systems, patient portals, Meaningful Use, patient education resources, user needs, interviews

Poster Number: 217

Time: Sunday, May 28, 2017, 2:00 PM – 2:55 PM

Health Fact or Fiction: Utilizing an iPad Flashcard App to Engage and Educate Fair Attendees

Katherine Chew, Associate Librarian / Research & Outreach Services, Health Sciences Libraries, Health Sciences Libraries / University of Minnesota, Minneapolis, MN; **Anne Beschnett**, Research & Instruction Librarian, St. Catherine University, Minneapolis, MN

Objectives: Attracting fair attendees to stop and interact with library staff about health information resources has always been a timeless problem, especially when you are competing against other, more “flashier” exhibits within the immediate area. The challenge is to come up with a way to captivate people and engage them long enough to enlighten them about reliable health information resources.

Methods: Since the mid-2000s, the Health Sciences Libraries has staffed a booth at their state fair highlighting NLM and library resources. In 2014, the booth staff debuted a new attraction - a health fact or fiction quiz that utilizes an iPad flashcard app. The quiz consists of health related questions, where the quiz taker would guess true or false, and then tap the iPad screen to see the answer or swipe to the next question. All of the answer pages contain the answer, a short explanation about why it was true or false and a url(s) to a health information resource. The initial quiz consisted of seven true / false questions and the following year this was expanded to fourteen questions. The questions were selected to cover a wide variety of health topics designed to provoke discussion about health information

Results: We had very engaged people who had fun trying to guess the medical device and the health true or false quiz had participants actually reading the information as to why a question's answer was true or false with a few friendly discussions about why a certain question was true versus false based on changing health information. We expected most people to try to answer just a few questions, but many cycled through all of the questions and a few even tried both quizzes. Comments received after taking the quizzes included “that was really interesting” or “I learned a lot.”

Conclusion: Fair attendees greatly enjoy participating in games and are very willing to spend the time at an exhibit where their brains are also engaged. The quizzes are a great way to connect with exhibit visitors by providing a setting that encourages shared story-telling and provides the opportunity to discuss a wide variety of health information, both from the aspect of what people think they know to opening up avenues of new knowledge and understanding.

Keywords: Outreach, Technology, Health Literacy, Engagement

Poster Number: 218

Time: Monday, May 29, 2017, 2:30 PM – 3:25 PM

Dreaming up a New Poster Printing Service: One Library's Experience

Ryan Harris, AHIP, Reference Services Manager, Research and Education Librarian, Health Sciences and Human Services Library, Health Sciences and Human Services Library, Baltimore, MD; **Everly Brown**, Head of Information Services, Health Sciences and Human Services Library, Health Sciences and Human Services Library / Information Services Department, Baltimore, MD; **Thom Pinho**, Instructional Technology Specialist, University of Maryland, Health Sciences and Human Services Library, Baltimore, MD

Objectives: Seeing a need to provide poster printing to campus users for professional and educational purposes, the Information Services department management team at a university's health sciences library investigated and designed a new poster printing service.

Methods: The Information Services management team, along with the library's IT department, investigated possible locations for the poster printer, created policies and procedures, and developed a submission form and back end administration portal for poster printing. The administration portal is highly automated; it allows library staff to easily identify what is being printed, notifies users when their print jobs are completed, and sends out a satisfaction survey to those who use the service. This process allowed us to help develop a work flow for printing. It was determined staff at the library's Information Services desk would print posters, as well as assist library patrons with the new service as needed. A subject guide that includes information about the service as well as guidance on how to format posters was created. A pricing model was established that is for cost recovery only.

Results: The poster printing service was launched in July 2016. The printer was put in the library's innovation space which already featured 3D printing and is highly visible on the library's first floor. In the first 6 months of the service, 156 posters were printed. 69% of posters were printed for conference presentations. The service was used by all schools on campus with the majority of users coming from the schools of nursing and pharmacy. Satisfaction surveys indicated that most users are highly satisfied with the promptness of the poster printing and poster quality.

Conclusion: While the poster printing service is relatively new, users seem quite pleased with it. The information services staff is tracking times of heavy use in order to anticipate future work flow and staffing needs to support the service. Based on feedback from the satisfaction survey, we will next investigate tools that can be used to cut posters printed smaller than our recommended dimensions. We will also start offering a Perfecting your Poster Presentation to assist users with designing posters.

Keywords: Poster Printing, collaboration, public services

Poster Number: 219

Time: Tuesday, May 30, 2017, 2:00 PM – 2:55 PM

Try Not to Get Sued!: The Pursuit of Accessibility and a Professional Captioning Service for All Library Videos

Kai Donovan, Media Developer, Taubman Health Sciences Library, University of Michigan, Ann Arbor, MI; **Elise Wescom**, Media Assistant/Graphic Artist, University of Michigan, Taubman Health Sciencs Library, Ann Arbor, MI; **Mark A. Chaffee**, Informationist Associate, Taubman Health Sciences Library, Ann Arbor, MI; **Jean Song**, Assistant Director, Academic and Clinical Engagement, Taubman Health Sciences Library, Taubman Health Sciences Library, Ann Arbor, MI

Objectives: Recently, major institutions such as Harvard and M.I.T. have been sued due to the lack of closed captions on their online educational materials. In light of these lawsuits our Library has undertaken the development of a process to caption all newly produced public online videos. This poster documents that process, as well as the investigation of utilizing a professional captioning service for our videos.

Methods: The Library has invested much time and many resources over the last several years into captioning all of their public videos, but this tedious process consumes staff time that could be better spent on other important projects. We will show the process of how we prepare captions for our videos to meet current accessibility standards and the data that is being collected to justify the expenditure of hiring a professional captioning service over health sciences library staff provided captions.

Results: The Library's video captioning process and captioning data collection procedures will be outlined and shared.

Conclusion: This poster will be useful for any health sciences library interested in making their videos accessible in a cost effective manner.

Keywords: captions; accessibility; video;

Poster Number: 220

Time: Tuesday, May 30, 2017, 2:00 PM – 2:55 PM

Benefits of Participating in a Mobile App Evaluation Project

Alicia Lillich, Kansas/Technology Coordinator, National Network of Libraries of Medicine/MidContinental Region (NN/LM-MCR), The University of Kansas Medical Center, Kansas City, KS; **John Bramble**, Research Enterprise / Outreach, Spencer S. Eccles Health Sciences Library, National Network of Libraries of Medicine, MidContinental Region, Salt Lake City, UT

Objectives: In order to improve confidence of using tablet computers, the NNLM MCR provided librarians with funds to experiment and evaluate paid medical or work appropriate apps and share their experience with the library community. This project examines changes in confidence, quality of evaluations, app selection, amounts spent, and benefits of project participation.

Methods: Two cohorts of participants were asked to review apps over a year long period. Qualified applicants agreed to evaluate at least four paid mobile apps appropriate to their work environment and submit evaluations by established deadlines. Evaluators were provided an online App Evaluation Report Form (AERF) that guided them to develop a systematic and critical evaluation of mobile apps. The AERF focused on: an app's authority/credibility of information sources; accuracy and objectivity; currency of information; organization and usability; and purpose. After completing the project, participants were asked to respond to a brief questionnaire about their perceived benefit and confidence in evaluating apps. The participant evaluations were assessed for quality and thoroughness.

Results: In year 1, 9 (81%) of 11 evaluators reported that being a participant in the project benefited their program. In year 2, 16 (84%) participants either strongly agreed or agreed that they feel more confident in their ability to evaluate mobile apps.

Keywords: mobile apps, evaluation, technology

Poster Number: 221

Time: Sunday, May 28, 2017, 2:00 PM – 2:55 PM

Rural Information Connection: An iPad Mini Lending Program to Rural Student Physicians

Emily M. Johnson, AHIP, Regional Health Sciences Librarian and Assistant Professor; **Carmen Howard**, Regional Health Sciences Librarian & Instructor; Library of the Health Sciences Peoria, Peoria, IL

Objectives: To determine the value of supplying medical students enrolled in a rural student physician longitudinal curriculum rotation with iPad minis that were pre-loaded with high-quality mobile health apps and to increase access to and awareness of mobile health information resources for clinical care in a rural environment.

Methods: Rural student physicians were loaned the iPad mini for a seven-month long rural medicine rotation. A mixed methodology for evaluation included using pre/post surveys and structured learning journal prompts. To analyze the success of this program, pre- and post-surveys were implemented at the beginning and end of the rotation using the validated Technology Acceptance Model (TAM) instrument. The students were asked to participate in structured learning journal prompts sent to them every three weeks during their rural placement allowing the investigators to gain insight of the use of the resources and the iPad device. The structured learning prompts were developed with an action research approach, using previous feedback from the prompts to develop new questions for student feedback. Thematic coding and analysis were carried out by two reviewers to identify resource use, task performance and participant reflection within the structured learning journals (SLJs).

Results: Nine students enrolled in the program with five having completed the rotation with the iPad mini to date; two students dropped out and two students are expected to finish in June 2017. The students reported using the device either daily or several times a week. Data gathered using the thematic coding of the SLJs and pre- and post-surveys show a consistent use of the device on the rural rotation for accessing information, answering clinical questions, sharing information with their rural preceptor, and studying for examinations. The resources or clinical information tools reported by the students prior to the rural rotation were used during the rotation and several students reported exploration and adoption of new evidence-based information resources. Participants also described several opinions on the implementing of the program within the rural environment, which allowed the investigators to modify the program for new participants.

Conclusion: The assessment informed the researchers by showing the information-seeking behavior in a rural environment and acceptance of new technology into the participants' workflow. With use of the iPad minis and resources, students were able to access essential clinical information and test prep material, adding to their educational experience within their rural student physician training.

Keywords: Mobile Devices, Rural Medicine, Medical Students, Surveys, Journaling

Poster Number: 222

Time: Monday, May 29, 2017, 2:30 PM – 3:25 PM

Tracking Tech Trends: Studying Patron Technology Use through Annual Surveying

Hannah F. Norton, AHIP, Interim Fackler Director, Associate University Librarian, Health Science Center Library, Biomedical and Health Information Services, University of Florida–Gainesville; **Mary E. Edwards, AHIP**, Reference & Liaison Librarian, Health Science Center Library, Biomedical and Health Information Services, Health Science Center Library, Gainesville, FL; **Ariel Pomputius, AHIP**, Health Sciences Liaison Librarian, University of Florida, Health Science Center Library, Gainesville, FL; **Michele R. Tennant, AHIP**, Interim Fackler Director, Health Science Center Library, Biomedical and Health Information Services, Health Science Center Libraries, Gainesville, FL

Objectives: At an academic health sciences library serving students, faculty, and staff across a wide variety of disciplines, studying library patrons' technology use, particularly in areas of mobile technology, provides necessary information on intersection points for library services. Administering a similar survey

annually for five years generates a holistic view of patrons' technology needs and preferences over time.

Methods: Beginning in 2012, the University of Florida (UF) Health Science Center Library (HSCL) began administering a 16-question survey designed by the University of Southern California Norris Medical Library to address technology use of health professional students and faculty and their interest in related library services. For three years we participated in a multi-institution implementation of this survey; when the collaboration ended, we continued to administer the survey at UF. While some questions have been modified over time for clarity or changes in available technology, many are consistent across the five years of survey implementation, allowing analysis of trends over time in use of specific technologies and service needs at our institution.

Results: Smartphone ownership among survey respondents is nearly universal (ranging from 87.6% to 95.7% over the past 5 years), and a majority of respondents also own a tablet (from 51.1% to 70.2%). While respondents were likely to check library hours, use medical apps, and use library electronic resources from their smartphone or tablet, they reported being unlikely to friend or follow the library on Facebook or Twitter or send a call number from the catalog. One simple change implemented in response to survey results was to add the library's hours to the "Quick Links" portion of the library's website; while the hours are featured on other parts of the site, the Quick Links are the most prominent portion of the site's mobile version. Likewise, when survey data indicated that respondents were highly interested in training on mobile device apps, the HSCL developed a stand-alone workshop entitled "Mobile Resources for Health." Trends that have not yet been explored further include respondents' preference for print books for both academic (53.1% to 57.3%) and leisure (53.2% to 55.2%) reading, as compared to ebooks.

Conclusions: Annual review of survey results has led to incremental changes in services offered. Reviewing the aggregate data allows for more strategic consideration of future directions, with implications towards marketing the library's resources, training development, and service development.

Keywords: mobile technology, technology trends, surveys and questionnaires, user input

Poster Number: 224

Time: Tuesday, May 30, 2017, 2:00 PM – 2:55 PM

A Healthy #Chat: Twitter Chat Participation to Engage Audiences, Share Health Resources, and Track Impact

Joelle A. Mornini, Analyst, ICF, National Institute of Health, National Library of Medicine/Outreach and Special Populations Branch, Bethesda, MD

Objectives: Twitter chats are a popular social media method for promoting awareness and sharing resources on consumer health topics. This project provides best practices on: how to locate relevant Twitter chats to attend, how to actively participate by sharing quality health resources and engaging participants to discuss and explore your resources, and how to measure the efficacy of the Twitter chats.

Methods: Over a period of eight months in 2016, Twitter chats were attended three or more times per month to share quality consumer health resources with family caregivers. Relevant Twitter chats were located through the Tweet Chats schedule on the website Symplur and through the Twitter feeds of government and non-profit organizations. Participation in the Twitter chats was conducted through HootSuite, a free tool with subscription features. Hootsuite allows the user to view multiple Twitter streams on one screen, which increases response speed and engagement opportunities. The percentage of tweets retweeted (shared by other users) and percentage of tweets favorited (liked by other users) was tracked on a monthly basis using the free online analytics tool Twitonomy (which includes subscription features), to see if attendance of Twitter chats led to higher levels of engagement with users and sharing of library resources.

Results: 34 Twitter chats were attended between May 1 and December 31, 2016, with between three and five chats attended monthly. Frequently attended chats focused on dementia caregiving and provided the opportunity to interact with family caregivers and organizations that work with caregivers and care recipients. Percentage of tweets retweeted ranged from 29% to 49% over the eight month period, and percentage of tweets favorited ranged from 28% to 37%. No positive correlation was found between number of Twitter chats attended and percentage of tweets retweeted or favorited.

Conclusion: Twitter chats provide a unique opportunity to engage with both healthcare professionals and the general public in order to share reliable health resources. Tools like Symplur, HootSuite, and Twitonomy can be used to more effectively identify, participate in, and measure impact of relevant chats. Although no positive correlation was found between number of chats attended and overall engagement, there was still a reasonably high level of Twitter engagement throughout the eight month period of this study. Further research with a larger sample of Twitter accounts is needed to identify if Twitter chat attendance consistently leads to higher levels of engagement.

Keywords: Social media, consumer health, special populations, user engagement

Poster Number: 225

Time: Sunday, May 28, 2017, 2:00 PM – 2:55 PM

Doing More with Facebook Data

Alyssa K. Peterson, Medical Librarian, Schusterman Library at OU-Tulsa, Tulsa, OK; **Katie A. Prentice, AHIP**, Associate Director for User Experience and Assessment, Schusterman Library, University of Oklahoma-Tulsa, Tulsa, OK

Objectives: This poster will describe how data was mined to assess and improve the impact of the Schusterman Library at the University of Oklahoma-Tulsa's Facebook Page.

Methods: At the Schusterman Library, a small academic library, data from Facebook Insights and Google Analytics were used over a several month period to analyze specific marketing initiatives for success. Based on the library's ongoing mission to increase library awareness and utilization, two campaigns, one featuring librarian endorsements of resources and services and a second assessing the utilization of links to the library's website were examined through data assessment to determine their effectiveness.

Results: As part of the library's ongoing mission to improve campus relationship and increase library use, librarians conducted and assessed social media campaigns aimed at improving campus engagement with Facebook posts link to the library web content. The first campaign resulted in increases in Facebook reach, reactions, comments, and shares ranging from 400% to 800%. The second campaign resulted in conflicting data from Facebook Insights and Google Analytics.

Conclusion: Librarians found that posting photographs of library staff and real users, personally endorsing library services, and showcasing librarians as people can improve the reach and impact of Facebook. However, due to inconsistent reporting of data related to increasing traffic to the library website, it is not clear if this success translates to web traffic. Overall, social media can be used successfully to engage campus audiences with library information.

Keywords: Social media, assessment, marketing, quality improvement

Poster Number: 226

Time: Monday, May 29, 2017, 2:30 PM – 3:25 PM

What's Your Brand? Teaching Students to Leverage Social Media to Launch Their Careers

Alexandra Gomes, AHIP, Associate Director for Education, Information, and Technology Services, Himmelfarb Health Sciences Library, The George Washington University, Washington, DC; **Gisela Butera**, Reference and Instruction Librarian, Himmelfarb Health Sciences Library, Himmelfarb Health Sciences Library, Washington, DC; **Anne Linton, AHIP**, Director, Himmelfarb Health Sciences Library, Himmelfarb Health Sciences Library, Washington, DC

Objectives: Despite stories in the press, social media can be a positive force in health care. Today's students are digital natives and should be introduced to professional practices to leverage these platforms for career enhancement. This poster describes the development of a social media instructional session, focused on creating a professional brand and adopting a positive professional approach to social media.

Methods: Since 2012, The George Washington University Himmelfarb librarians have coordinated an instructional session for students designed to raise awareness of e-professionalism as students transition to the role of healthcare professionals. This session has been adapted for a variety of student audiences including medicine, nursing, pharmacy, and physician assistant students. In 2016 the Physical Therapy program approached the library about reworking this session for their capstone students, focusing instead on positive methods to leverage social media to build their careers. A facilitated conversation and panel discussion of faculty mentors and social media communication experts was created, to help promote best practices on branding professional personas online. The session discussed how to create a professional LinkedIn profile, social media networks to join, and the overall development of digital professional identity. The development process, resulting instructional session, and student feedback will be described.

Results: Five panelists participated in the 1.5 hour session: a Physical Therapy (PT) faculty member, a health sciences faculty member, a recent PT program graduate, a fourth year medical student, and media relations staff member. All the panelists shared their experiences in using social media to advance their careers. All (45) graduating students attended. The students were encouraged to explore multiple professional social media platforms including LinkedIn and ResearchGate as well as to review their personal social media presence for material that may put them in a less favorable light with potential employers and/or patients. Stakeholder feedback from the course director and students was positive.

Conclusion: PT students are very interested in learning more about how social media can help them find a job and enhance their careers. Key take home messages to the students were: 1) the power of collaboration to find professional partners, create new networks, and enable people to find you, 2) the importance of being proactive, and 3) the amount of time needed to update and manage a professional identity on these platforms.

Producing this social media panel was very satisfying, but it takes a lot of coordination and lead time to put together.

Keywords: social media, professionalism, instruction, careers, digital identity, branding

Poster Number: 227

Time: Tuesday, May 30, 2017, 2:00 PM – 2:55 PM

Ontologies, Tag Collections, and Folksonomies: Curating Disease Hashtags to Enhance Collaboration and Dialogue

Lynne Frederickson, Informationist; **Patricia F. Anderson**, Emerging Technologies Informationist; Taubman Health Sciences Library, Ann Arbor, MI

Objectives: Hashtags arise spontaneously, creating rich and dynamic folksonomies for tracking online conversations. Traditional user-generated hashtags are effective for narrowcasting events and supporting localized conversations, but the lack of conventions creates confusion and does not scale

well for broader dialogues on complex themes. We intend to promote a better understanding of ontological concepts to facilitate consensus and enhance disease-specific online dialogue.

Methods: In order to support and enhance disease-specific conversations among patients, researchers, and practitioners, we define and clarify the distinctions between user-generated folksonomies and more formal tag collections and ontologies, present general concepts and best practices for tag collections, and introduce the concept of tag curation.

We present best practices for applying ontological concepts to user-generated tag collections, and by doing so explain through examples the concepts of tag length, modularization, stemming and sorting, and hierarchical relationships.

We describe ongoing curatorial activities, including applying content and form criteria and identifying and filling domain-specific gaps in tag collections, and how these activities promote participation and consistency. The benefits of tag curation will be illustrated with examples and antipatterns.

We present the cancer tag collection project as a working model, including examples of specific challenges and how they were resolved.

Keywords: twitter, ontologies, hashtags, social media, tag curation

Poster Number: S10

Time: Sunday, May 28, 2017, 2:00 PM – 2:55 PM

Medical Library Education Section Poster

Section Chapter ePosters are not staffed during the meeting, and will be viewable in the Registration Area throughout the Meeting, and available in the online ePoster gallery. Look for the kiosk in the MLA Connection Area located within the Registration Center to view the posters onsite.

Poster Number: S11

Time: Sunday, May 28, 2017, 2:00 PM – 2:55 PM

Nursing and Allied Health Resources Section Poster

Section Chapter ePosters are not staffed during the meeting, and will be viewable in the Registration Area throughout the Meeting, and available in the online ePoster gallery. Look for the kiosk in the MLA Connection Area located within the Registration Center to view the posters onsite.

Poster Number: S12

Time: Sunday, May 28, 2017, 2:00 PM – 2:55 PM

Pharmacy and Drug Information Section Poster

Pharmacy and Drug Information Section, NA, NA, NA, N/A

Section Chapter ePosters are not staffed during the meeting, and will be viewable in the Registration Area throughout the Meeting, and available in the online ePoster gallery. Look for the kiosk in the MLA Connection Area located within the Registration Center to view the posters onsite.

Poster Number: S13

Time: Sunday, May 28, 2017, 2:00 PM – 2:55 PM

Relevant Issues Section Poster

Relevant Issues Section, Joan Marcotte Gregory, Chair, Jim Anderson, Chair-Elect, Kate Flewelling, Past Chair, Chicago, IL

Section Chapter ePosters are not staffed during the meeting, and will be viewable in the Registration Area throughout the Meeting, and available in the online ePoster gallery. Look for the kiosk in the MLA Connection Area located within the Registration Center to view the posters onsite.

Poster Number: S14

Time: Sunday, May 28, 2017, 2:00 PM – 2:55 PM

Research Section Poster

Research Section, Section , Medical Library Association, Chicago, IL

Section Chapter ePosters are not staffed during the meeting, and will be viewable in the Registration Area throughout the Meeting, and available in the online ePoster gallery. Look for the kiosk in the MLA Connection Area located within the Registration Center to view the posters onsite.

Poster Number: S15

Time: Sunday, May 28, 2017, 2:00 PM – 2:55 PM

Technical Services Section Poster

Section Chapter ePosters are not staffed during the meeting, and will be viewable in the Registration Area throughout the Meeting, and available in the online ePoster gallery. Look for the kiosk in the MLA Connection Area located within the Registration Center to view the posters onsite.

Poster Number: S16

Time: Sunday, May 28, 2017, 2:00 PM – 2:55 PM

Veterinary Medical Libraries Section Poster

Section Chapter ePosters are not staffed during the meeting, and will be viewable in the Registration Area throughout the Meeting, and available in the online ePoster gallery. Look for the kiosk in the MLA Connection Area located within the Registration Center to view the posters onsite.

Poster Number: S17

Time: Sunday, May 28, 2017, 2:00 PM – 2:55 PM

Hawaii-Pacific Chapter Poster

Hawaii-Pacific Chapter, Diane Kunichika, Medical Librarian, Tripler Army Medical Library, Honolulu, HI

Section Chapter ePosters are not staffed during the meeting, and will be viewable in the Registration Area throughout the Meeting, and available in the online ePoster gallery. Look for the kiosk in the MLA Connection Area located within the Registration Center to view the posters onsite.

Poster Number: S18

Time: Sunday, May 28, 2017, 2:00 PM – 2:55 PM

Medical Library Group of Southern California and Arizona (MLGSCA) Chapter Poster

Section Chapter ePosters are not staffed during the meeting, and will be viewable in the Registration Area throughout the Meeting, and available in the online ePoster gallery. Look for the kiosk in the MLA Connection Area located within the Registration Center to view the posters onsite.

Poster Number: S19

Time: Sunday, May 28, 2017, 2:00 PM – 2:55 PM

Mid-Atlantic Chapter (MAC) Poster

Section Chapter ePosters are not staffed during the meeting, and will be viewable in the Registration Area throughout the Meeting, and available in the online ePoster gallery. Look for the kiosk in the MLA Connection Area located within the Registration Center to view the posters onsite.

Poster Number: S20

Time: Sunday, May 28, 2017, 2:00 PM – 2:55 PM

Midcontinental Chapter (MCMLA) Poster

Section Chapter ePosters are not staffed during the meeting, and will be viewable in the Registration Area throughout the Meeting, and available in the online ePoster gallery. Look for the kiosk in the MLA Connection Area located within the Registration Center to view the posters onsite.

Poster Number: S21

Time: Sunday, May 28, 2017, 2:00 PM – 2:55 PM

Midwest Chapter Poster

Midwest Chapter, Membership Secretary, Midwest Chapter of MLA, Columbus, OH

Section Chapter ePosters are not staffed during the meeting, and will be viewable in the Registration Area throughout the Meeting, and available in the online ePoster gallery. Look for the kiosk in the MLA Connection Area located within the Registration Center to view the posters onsite.

Poster Number: S22

Time: Sunday, May 28, 2017, 2:00 PM – 2:55 PM

New York-New Jersey (NY-NJ) Chapter Poster

Section Chapter ePosters are not staffed during the meeting, and will be viewable in the Registration Area throughout the Meeting, and available in the online ePoster gallery. Look for the kiosk in the MLA Connection Area located within the Registration Center to view the posters onsite.

Poster Number: S23

Time: Sunday, May 28, 2017, 2:00 PM – 2:55 PM

North Atlantic Health Sciences Libraries (NAHSL) Chapter Poster

North Atlantic Health Sciences Libraries Chapter, na, na, na, N/A

Section Chapter ePosters are not staffed during the meeting, and will be viewable in the Registration Area throughout the Meeting, and available in the online ePoster gallery. Look for the kiosk in the MLA Connection Area located within the Registration Center to view the posters onsite.

Poster Number: S24

Time: Sunday, May 28, 2017, 2:00 PM – 2:55 PM

Northern California and Nevada Medical Library Group (NCNMLG) Chapter Poster

Section Chapter ePosters are not staffed during the meeting, and will be viewable in the Registration Area throughout the Meeting, and available in the online ePoster gallery. Look for the kiosk in the MLA Connection Area located within the Registration Center to view the posters onsite.

Poster Number: S25

Time: Sunday, May 28, 2017, 2:00 PM – 2:55 PM

Philadelphia Regional Chapter Poster

Philadelphia Regional Chapter, Chapter Council Representative, MLA-Phil, Philadelphia, PA

Section Chapter ePosters are not staffed during the meeting, and will be viewable in the Registration Area throughout the Meeting, and available in the online ePoster gallery. Look for the kiosk in the MLA Connection Area located within the Registration Center to view the posters onsite.

Poster Number: S26

Time: Sunday, May 28, 2017, 2:00 PM – 2:55 PM

South Central Chapter (SCC) Poster

South Central Chapter, Chapter, SCC/MLA, Shreveport, LA

Section Chapter ePosters are not staffed during the meeting, and will be viewable in the Registration Area throughout the Meeting, and available in the online ePoster gallery. Look for the kiosk in the MLA Connection Area located within the Registration Center to view the posters onsite.

Poster Number: S27

Time: Sunday, May 28, 2017, 2:00 PM – 2:55 PM

Southern Chapter (SC) Poster

Section Chapter ePosters are not staffed during the meeting, and will be viewable in the Registration Area throughout the Meeting, and available in the online ePoster gallery. Look for the kiosk in the MLA Connection Area located within the Registration Center to view the posters onsite.
